# Amphoteric chalcogen-bonding and halogen-bonding rotaxanes for anion or cation recognition

**DOI:** 10.1038/s41557-025-01742-x

**Published:** 2025-02-20

**Authors:** Yuen Cheong Tse, Andrew Docker, Igor Marques, Vítor Félix, Paul D. Beer

**Affiliations:** 1 Chemistry Research Laboratory, Department of Chemistry, University of Oxford, Oxford, UK; 2 CICECO – Aveiro Institute of Materials, Department of Chemistry, University of Aveiro, Aveiro, Portugal

**Keywords:** Supramolecular chemistry, Interlocked molecules, Halogen bonding

## Abstract

The ever-increasing demand in the development of host molecules for the recognition of charged species is stimulated by their fundamental roles in numerous biological and environmental processes. Here, capitalizing on the inherent amphoteric nature of anisotropically polarized tellurium or iodine atoms, we demonstrate a proof of concept in charged guest recognition, where the same neutral host structure binds both cations or anions solely through its chalcogen or halogen donor atoms. Through extensive ^1^H nuclear magnetic resonance titration experiments and computational density functional theory studies, a library of chalcogen-bonding (ChB) and halogen-bonding (XB) mechanically interlocked [2]rotaxane molecules, including seminal examples of all-ChB and mixed ChB/XB [2]rotaxanes, are shown to function as either Lewis-acidic or Lewis-basic multidentate hosts for selective halide anion and metal cation binding. Notably, the exploitation of the inherent amphoteric character of an atom for the strategic purpose of either cation or anion recognition constitutes the inception of a previously unexplored area of supramolecular host–guest chemistry.

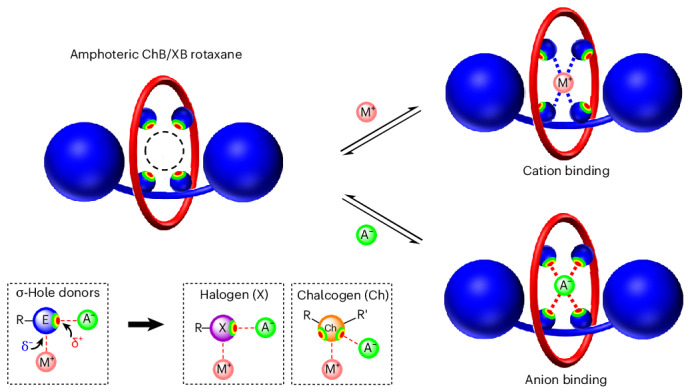

## Abstract (web summary)

The importance of charged species in numerous biological and environmental processes has stimulated the development of host molecules for their selective recognition. Now anisotropically polarized halogen- and chalcogen-bonding [2]rotaxanes are demonstrated to exhibit dual Lewis-acidic and Lewis-basic amphoteric properties for anion or cation recognition via the same donor atoms.

## Main

In recent years, a unique set of non-covalent binding forces in supramolecular chemistry has emerged, in particular, the sigma (σ)-hole interactions chalcogen bonding (ChB) and halogen bonding (XB)^1,2^. Defined as the attractive interaction between an electrophilic region of a group 16 or 17 atom and a Lewis base^3,4^, seminal applications of ChB and XB in crystal engineering^5,6^, self-assembly^7–10^ and organocatalysis^11–17^ have identified these bonding interactions as powerful complements to traditional interactions such as hydrogen bonding (HB). In the context of anion recognition, ChB or XB host systems frequently outperform the anion affinity and selectivity of their HB host analogues^18–27^.

The Lewis-acidic characteristics of ChB/XB originate from localized electronically depleted σ-hole regions on the surface of the chalcogen/halogen atoms anisotropically polarized by electron-withdrawing substitutents^2,28^. However, an implicit corollary to this non-uniform electronic distribution is the preservation of electron-dense regions encompassing the σ-hole, conceivably imparting simultaneous nucleophilic and electrophilic, or ‘amphoteric’, character to ChB^29,30^ and XB^31–36^ donors. The ability of the heavier chalcogens, in particular tellurium, to function as either a Lewis base^37–40^ or Lewis acid^9,41–44^ has been recognized for many decades, and although somewhat less common, organohalogen compounds have also been shown in the solid state to exhibit halogen-based nucleophilicity manifesting as type II halogen–halogen interactions^45–47^ and halogen···metal interactions^48–51^. In charged species recognition, this apparently antithetical behaviour presents a remarkable opportunity for developing motifs capable of binding cationic or anionic species in solution through the same donor atom.

**Fig. 1:**
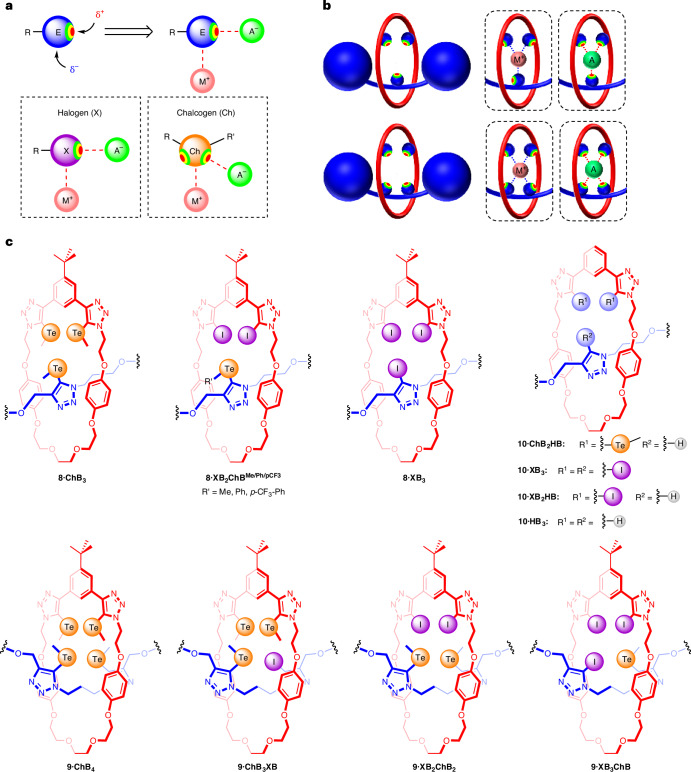
Amphoteric σ-hole [2]rotaxanes for cation or anion recognition. **a**, Anisotropically polarized halogen and chalcogen atoms possess localized electron-deficient σ-holes encompassed by electron-dense regions, endowing them with amphoteric character for anion binding or cation coordination (E = halogen or chalcogen; R, R′ = electron-withdrawing groups). **b**, The design of amphoteric tri- and tetradentate [2]rotaxanes incorporated with σ-hole donors inside the rotaxane cavities for convergent and multidentate cation or anion binding. **c**, Structures of the library of amphoteric tri- and tetradentate ChB/XB/HB σ-hole [2]rotaxanes studied in this work.

To this end, we sought to investigate whether multidentate ChB and XB donor arrays incorporated into mechanically interlocked [2]rotaxane host architectures could capitalize on this potential amphoteric behaviour and function as novel ion receptor systems, in which both anion or cation binding can occur endotopically using the same donor atoms (Fig. 1). We herein report the synthesis of a library of neutral [2]rotaxanes, containing tri- and tetradentate telluro- and iodo-triazole arrays, including the first examples of all-ChB and mixed ChB/XB donor interlocked host structures. Extensive ^1^H nuclear magnetic resonance (NMR) titration experiments and computational investigations reveal how the combination, denticity and nature of the σ-hole donors influence halide anion recognition properties of the rotaxane series. Cation binding studies demonstrate that the chalcogen and halogen donor atoms of the rotaxane hosts function as Lewis bases, facilitating the complexation of transition and post-transition metals with remarkable affinities and selectivity.

## Results and discussion

A copper(I)-catalysed azide–alkyne cycloaddition (CuAAC)-mediated active metal template (AMT) methodology was exploited to construct the target multidentate [2]rotaxane series^52–55^, where the endotopically coordinated macrocycle·Cu(I) species catalyses a [3 + 2] cycloaddition reaction forming the mechanically bonded axle. We have previously established that the ambidentate 1,3-bis(iodo-/proto-triazole) benzene motif is capable of triazole-N···Cu(I) ligation through a rotation of the triazole heterocycle, which facilitates [2]rotaxane synthesis^56,57^. Interestingly, a telluromethyl-triazole-functionalized analogue was also demonstrated to be AMT compliant via direct Cu(I)···Te coordination^56^. These initial observations motivated the development of interlocked host systems featuring multidentate ChB/XB donors, with potential amphoteric character for anion or cation recognition purposes.

### Synthesis of σ-hole [2]rotaxanes

**Fig. 2:**
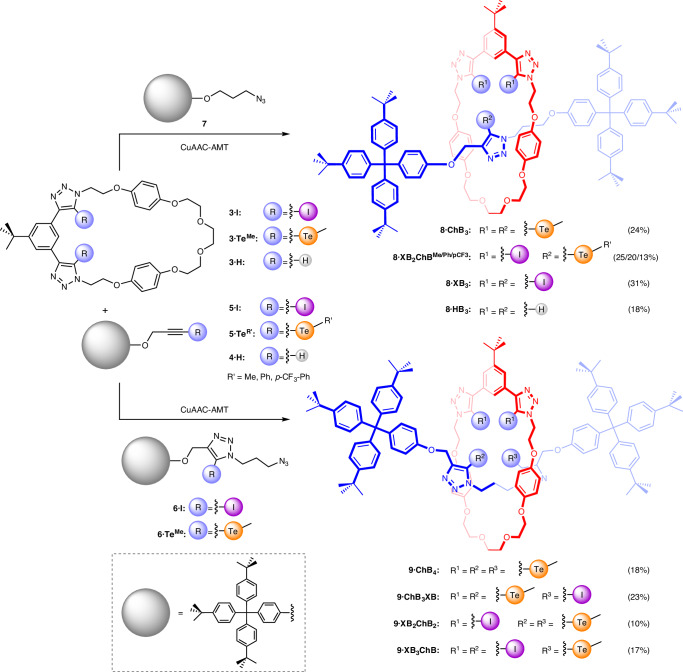
CuAAC-AMT synthesis of amphoteric ChB/XB/HB [2]rotaxane receptors. CuAAC-AMT reaction of a 1:1 mixture of macrocycle **3** and [Cu(CH_3_CN)_4_]PF_6_, in the presence of excess alkynyl **5** and azido **6**/**7** stopper units affords the series of tri- (**8**) and tetradentate (**9**) ChB/XB/HB amphoteric [2]rotaxanes. The binding cavities of the mechanically interlocked receptors are incorporated with amphoteric ChB/XB/HB donors for convergent and multidentate anion or cation recognition. Isolated yields are indicated in parentheses.

The detailed synthesis and characterization of the [2]rotaxane series is described in Supplementary Sections 1 and 2. In a typical CuAAC-AMT rotaxane synthesis (Fig. 2), an equimolar mixture of the macrocycle and [Cu(CH_3_CN)_4_]PF_6_ was stirred at room temperature in CH_2_Cl_2_ with a fivefold excess of the appropriate stopper azide and alkyne precursors. After an EDTA/NH_4_OH aqueous work-up procedure, purification by preparative thin-layer chromatography (TLC) afforded the series of rotaxanes in variable yields. Importantly, the modular AMT approach allowed access to all-ChB and mixed ChB/XB [2]rotaxanes containing up to four σ-hole triazole-based donors. The ChB binding site was further engineered by using aryl-tellurium functionalized stopper precursors, affording [2]rotaxanes **8·XB**_**2**_**ChB**^**Ph**^ and **8·XB**_**2**_**ChB**^**pCF3**^.

### Anion binding properties of σ-hole [2]rotaxanes

The halide anion binding properties of the σ-hole [2]rotaxane series were investigated by ^1^H NMR titration experiments in acetone-*d*_6_ (Supplementary Section 3). Aliquots of halides as their tetrabutylammonium (TBA) salts were titrated against solutions of the interlocked hosts, and changes in chemical shifts in the resulting ^1^H NMR spectra were monitored. Overall, notable perturbations were observed for both the internal benzene proton *a* and TeCH_3_ signals, indicating the convergent binding of anions by the chalcogen/halogen donors near the shielded rotaxane cavity. Bindfit^58,59^ analysis of the anion binding isotherms, generated by monitoring the changes in chemical shifts as a function of anion concentration, determined 1:1 stoichiometric host–guest anion association constants (Table 1).

**Table 1:** Anion association constants (*K*_a_, in M^−1^) for tri- and tetradentate [2]rotaxanes with TBA halide salts in acetone-*d*_6_ or 2% D_2_O/acetone-*d*_6_ determined by ^1^H NMR titrations^a^

	Acetone-*d*_6_			2% D_2_O/acetone-*d*_6_
[2]Rotaxane	Cl^−^	Br^−^	I^−^	Cl^−^
**10·XB**_**3**_	>10^5^	/	/	/
**8·XB**_**2**_**ChB**^**Me**^	4,090 (351)	2,330 (41)	977 (24)	89 (1)
**10·XB**_**2**_**HB**	4,380 (322)	2,140 (68)	902 (57)	/
**8·ChB**_**3**_	130 (1)	80 (1)	46 (<1)	/
**10·ChB**_**2**_**HB**	127 (2)	110 (2)	34 (3)	/
**10·HB**_**3**_	81 (3)	65 (5)	43 (1)	/
**8·XB**_**2**_**ChB**^**Ph**^	/	/	/	69 (1)
**8·XB**_**2**_**ChB**^**pCF3**^	/	/	/	173 (3)
**9·XB**_**3**_**ChB**	>10^5^	>10^5^	3,080 (153)	/
**9·XB**_**2**_**ChB**_**2**_	10,600 (394)	4,220 (137)	1,090 (21)	/
**9·ChB**_**3**_**XB**	557 (13)	253 (4)	99 (2)	/
**9·ChB**_**4**_	172 (2)	100 (1)	56 (1)	/

^a^*K*_a_ values calculated using Bindfit^58,59^ with a 1:1 host–guest binding model. Errors (±) are in parentheses and are all <5%. Solvent: acetone-*d*_6_ or 2% D_2_O/acetone-*d*_6_. *T* = 298 K. The rotaxane concentration was 1.0 mM, except for [**9·ChB**_**4**_] = [**9·XB**_**2**_**ChB**_**2**_] = 0.5 mM. Data for **10·XB**_**3**_, **10·XB**_**2**_**HB**, **10·ChB**_**2**_**HB** and **10·HB**_**3**_ are adopted from refs. ^56,57^.

The solidus indicates that the titration experiment was not performed.

Inspection of Table 1 reveals that all receptors display a general preference for smaller halides (Cl^−^ > Br^−^ > I^−^), rationalized on host–guest size complementarity and anion basicity arguments. Notably, the XB hosts exhibit the largest anion affinities followed by the ChB analogues (XB > ChB > HB). Increasing the number of iodo-triazole XB donor moieties dramatically enhances anion affinity, as demonstrated by the sequential substitution of ChB donors with the XB donor in the tetradentate σ-hole [2]rotaxane series (**9·ChB**_**4**_ < **9·ChB**_**3**_**XB** < **9·XB**_**2**_**ChB**_**2**_ < **9·XB**_**3**_**ChB**). Likewise, the incorporation of telluromethyl-triazole ChB donors into the tridentate HB rotaxane framework elicits a modest but noticeable enhancement in Cl^−^ and Br^−^ affinity (**10·HB**_**3**_ < **10·ChB**_**2**_**HB**). In addition, the replacement of chalcogen with halogen donors substantially influences receptor anion selectivity towards more charge-dense halide anions, as indicated by comparing the *K*_a_(Cl^−^)/*K*_a_(I^−^) values of the tetradentate hosts.

Complementing halide binding strength tuneability of the rotaxanes via σ-hole substitution and variation, the divalent nature of the Te donor also presents a unique opportunity to further finetune anion affinity through the electronic and steric properties of substituents bonded to the ChB donors. To illustrate, anion recognition properties of [2]rotaxanes **8·XB**_**2**_**ChB**^**Ph**^ and **8·XB**_**2**_**ChB**^**pCF3**^, consisting of aryl-functionalized telluro-triazole ChB donors in the axle component, as well as their methyl analogue **8·XB**_**2**_**ChB**^**Me**^, were probed by chloride titration in 2:98 D_2_O/acetone-*d*_*6*_. This solvent mixture was chosen to also potentially exploit the solvent shielding effect of introducing a hydrophobic aromatic substituent around the rotaxane binding domain in this competitive aqueous environment (Supplementary Figs. 61,63). A slight diminution in chloride affinity was observed upon replacing the methyl substituent (*K*_*a*_ = 89 M^−1^) with a phenyl group (*K*_a_ = 69 M^−1^), which may be rationalized by the steric hindrance imposed by the aryl ring. By contrast, a 2.5-fold increase in chloride binding (*K*_a_ = 173 M^−1^) was observed for **8·XB**_**2**_**ChB**^**pCF3**^, suggesting that the more electron-deficient *p*-trifluoromethylphenyl substituent enhances ChB donor potency.

### Cation binding properties of σ-hole [2]rotaxanes

Having investigated the anion recognition capabilities of the σ-hole [2]rotaxanes, our attention turned to studying their cation binding properties. Whilst tellurium is commonly encountered as a donor atom for complexing soft metal cations including Ag(I) (refs. ^37–40^), the observation of Ag(I)···halogen interactions with remarkably short distances is well documented in the solid state^48–51^. Therefore, we sought to establish whether the nucleophilic regions of the ChB tellurium and XB iodine donor atoms could also function as cation binding sites, with initial efforts directed towards chalcogen/halogen-mediated Ag(I) coordination.

**Fig. 3:**
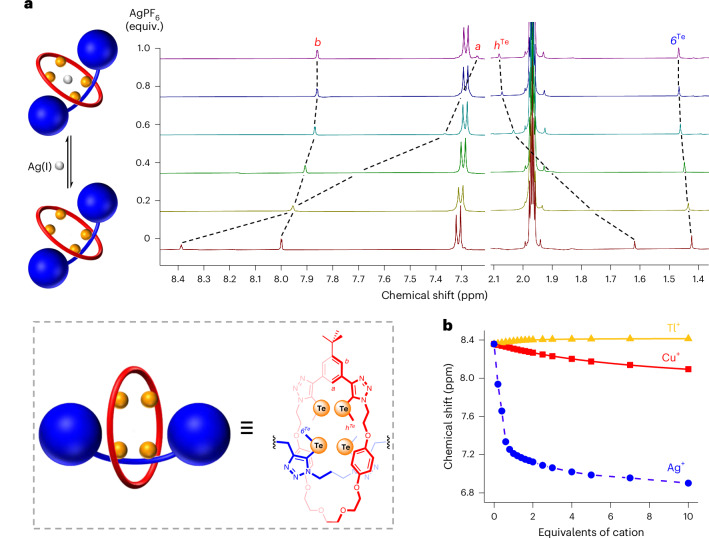
^1^H NMR cation titration of 9·ChB_4_. **a**, Truncated ^1^H NMR titration spectra of **9·ChB**_**4**_ upon addition of 0.2, 0.4, 0.6, 0.8 and 1.0 equivalents of AgPF_6_. **b**, Binding isotherms showing changes in chemical shift of proton *a* with increasing equivalents of metal cation. The filled dots represent the experimental data, the solid lines show the calculated isotherms and the dashed lines are a visual aid ([**9·ChB**_**4**_] = 1.0 mM, 500 MHz, 298 K, CD_3_CN). Source data

Undertaking ^1^H NMR titration investigations in CD_3_CN (for solubilizing both the hosts and cation salts), the addition of AgPF_6_ induced notable perturbations to the proton signals from the triazole-decorated cavities in both the macrocyclic and interlocked ChB or XB receptors (Fig. 3a). The magnitude of these changes was most pronounced for the TeCH_3_ protons of the ChB receptors, suggesting the formation of direct Ag(I)···Te attractive interactions, corroborated by density functional theory (DFT) analysis (vide infra). In the case of both tri- and tetradentate ChB [2]rotaxanes **8·ChB**_**3**_ and **9·ChB**_**4**_, the addition of Ag(I) resulted in considerable line broadening of internal benzene proton *a* and TeCH_3_ signals *h*^Te^ in the vicinity of the interlocked binding cavity until one equivalent of the cation had been administered, after which only minor shifts were observed, suggesting the formation of a highly stable tellurium-mediated Ag(I)·rotaxane complex. Analogous ^1^H NMR cation titrations were also conducted with other soft metal cations, Cu(I) and Tl(I) (Supplementary Section 4), and Bindfit^58,59^ analysis of the metal cation binding isotherms (Fig. 3b) determined 1:1 stoichiometric host–guest association constants (Table 2).

**Table 2:** Cation association constants (*K*_a_, in M^−1^) for [2]rotaxanes and macrocycles with various metal salts in CD_3_CN determined by ^1^H NMR titrations^a^

Receptor	AgPF_6_	[Cu(CH_3_CN)_4_]PF_6_	TlPF_6_
**9·ChB**_**4**_	>10^5^	113 (1)	2,350 (127)
**8·ChB**_**3**_	>10^5^	98 (1)	1,570 (52)
**3·Te**^**Me**^	7,600 (1,001)^b^	22 (1)	84 (3)
**8·XB**_**3**_	125 (1)	40 (2)	318 (7)
**3·I**	n.b.	n.b.	120 (4)
**8·HB**_**3**_	220 (2)	40 (1)	282 (6)

^a^*K*_a_ values calculated using Bindfit^58,59^ with a 1:1 host–guest stoichiometric binding model. Errors (±) are in parentheses and are all <5% unless otherwise stated. Solvent, CD_3_CN. *T* = 298 K. Receptor concentration, 1.0 mM.

^b^Error, 13%.

n.b., no binding.

Table 2 reveals that ChB receptors exhibit a pronounced preference for the Ag(I) cation, wherein a notable mechanical bond enhancement^60^ in metal cation recognition is evident with the silver(I) affinities of the tri- and tetradentate interlocked ChB [2]rotaxanes **8·ChB**_**3**_ and **9·ChB**_**4**_ (*K*_a_ > 10^5^ M^−1^) being at least an order of magnitude greater than macrocycle **3·Te**^**Me**^ (*K*_a_ = 7600 M^−1^). Moreover, all ChB receptors displayed much stronger Ag(I) binding compared with the XB and HB hosts. Although relatively weakly bound, a noticeable mechanical bond enhancement in Cu(I) binding is also observed for rotaxanes **9·ChB**_**4**_/**8·ChB**_**3**_/**8·XB**_**3**_ over their respective macrocycles **3·Te**^**Me**^/**3·I**. This is attributed to both the stronger innate preference of the polarizable tellurium/iodine donor atoms towards the Ag(I) cation and the size complementarity between the rotaxane host binding domain and the metal cation, with the ionic radius of Cu(I) (0.77 Å) being notably smaller than that of Ag(I) (1.15 Å) and Tl(I) (1.50 Å)^61^.

Thallium(I) binding titration experiments also determined that the ChB and XB interlocked systems exhibit larger association constants than their corresponding macrocyclic components alone (**3·Te**^**Me**^/**3·I**), which is especially pronounced in the case of **8·ChB**_**3**_ and **9·ChB**_**4**_. Interestingly, the direction of the chemical shift perturbation observed for the *tert*-butyl phenyl ring protons (*a* and *b*) during the Tl(I) cation titration experiments is opposite to that observed for the other cations, suggesting the formation of cation···π interactions between the aromatic ring and Tl(I), as corroborated by DFT studies (vide infra).

Furthermore, a comparison of the metal cation affinities between **8·XB**_**3**_ and **8·HB**_**3**_ tentatively indicates evidence of Lewis-basic iodine donor contribution in cation binding. Considering the more electron-withdrawing nature of the iodine atom, **8·HB**_**3**_ was predicted to exhibit an overall stronger cation association than **8·XB**_**3**_, due to the more basic nitrogen donors of proto-triazole compared with iodo-triazole. Surprisingly, comparable Cu(I) and stronger Tl(I) associations were observed for **8·XB**_**3**_, potentially suggesting the formation of metal···iodine interactions, which was further supported by the combination of additional metal cation ^1^H NMR titrations, ultraviolet–visible spectroscopic studies and the successful AMT synthesis of a XB rotaxane analogue in which iodo-triazole nitrogen coordination to Cu(I) is sterically not feasible in the XB macrocycle precursor (Supplementary Section 6). Computational DFT studies also support the Lewis-basic iodine donor metal cation binding mode of **8·XB**_**3**_ (vide infra).

To exclude the possibility of spectral perturbations due to non-specific electrostatic interactions or changes in the solvent medium dielectric properties, a ^1^H NMR titration of **8·ChB**_**3**_ with hard K^+^, as its tetrakis(3,5-bis(trifluoromethyl)phenyl)borate (BAr^F^_4_) salt, was conducted (Supplementary Fig. 92). Crucially, no binding was observed between the ChB rotaxane and the alkali metal cation, despite the same +1 charge as Ag(I) and Tl(I), and the latter being of comparable ionic radius (*r*(K^+^) = 1.38 Å)^61^.

### Modelling studies

Computational insights into the amphoteric nature of ChB/XB σ-hole binding sites were obtained through Gaussian 16 (ref. ^62^) DFT calculations on the halide and metal cation complexes of **8·ChB**_**3**_**′** and **8·XB**_**3**_**′**, respective prototypes of **8·ChB**_**3**_ and **8·XB**_**3**_ where the bulky axle stoppers were replaced by methyl groups (Supplementary Section 5).

**Fig. 4:**
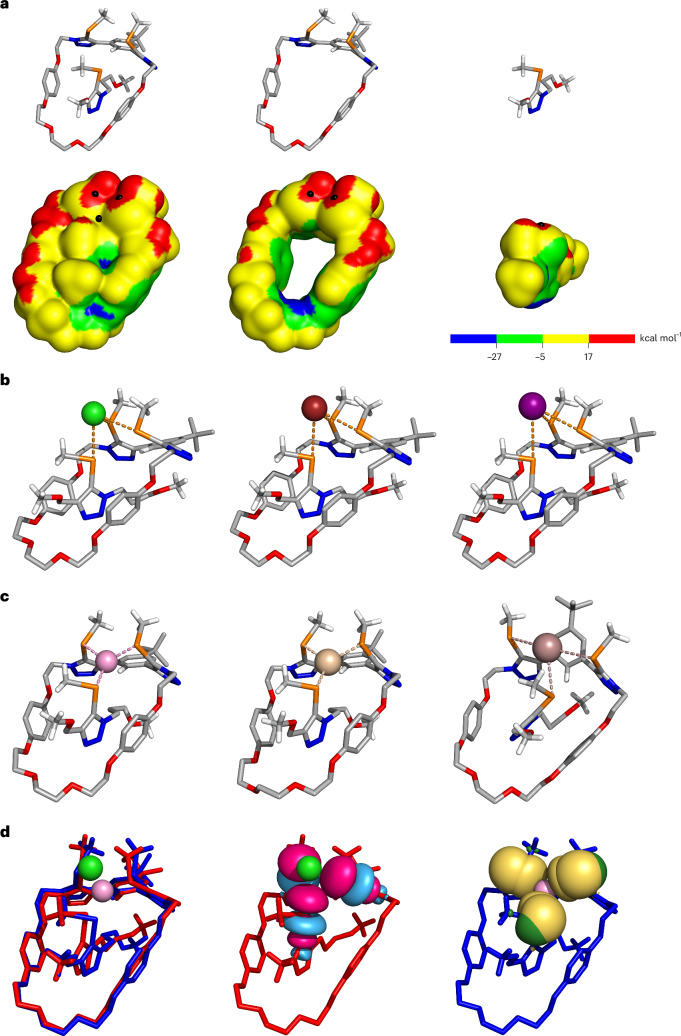
DFT calculations on 8·ChB_3_′. **a**, PBE0-D3(BJ)/def2-TZVP gas-phase optimized structure of **8·ChB**_**3**_**′**, together with its isolated macrocyclic and axle components and their corresponding MEPs (0.001 e Bohr^−3^). The most positive values of each TeMe binding unit are identified with black hemispheres. **b**, M06-2X/def2-TZVP(D)/acetone optimized structures of Cl^−^, Br^−^ and I^−^ associations with **8·ChB**_**3**_**′**. **c**, PBE0-D3BJ/def2-TZVP/acetonitrile optimized structures of **8·ChB**_**3**_**′** metal complexes, showing Cu(I) (left), Ag(I) (centre) or Tl(I) (right) coordinated to the TeMe binding sites of the pseudo-[2]rotaxane host. **d**, Optimized structures of **8·ChB**_**3**_**′** complexes with Cl^−^ (red) or Cu(I) (blue) overlapped (left), along with the NBO Ω*_Ctrz–Te_ orbitals (pink and light blue, centre) and the NBO *n*_Te_ orbitals (yellow and green, right), drawn at the 0.03 e Bohr^−3^ contour. In **b** and **c**, the intermolecular interactions are represented as dashed lines, regardless of their nature.

The amphoteric character of the tellurium binding sites was initially investigated with the gas-phase evaluation of the molecular electrostatic potential (MEP) of **8·ChB**_**3**_**′** and its isolated components (Fig. 4a). Generally, the most positive values of electrostatic potential (*V*_s,max_) were observed on tellurium centres in both the **3·Te**^**Me**^ macrocycle and the prototypic axle, ascribed to σ-holes induced by the C_trz_–Te bonds (trz = triazole). Concomitantly, the calculated negative MEP regions, almost perpendicular to the C_trz_–Te bonds, are consistent with their Lewis-basic property. Pseudo-[2]rotaxane **8·ChB**_**3**_**′** is characterized by a well-defined positive MEP region that encloses the three tellurium donors, but no negative *V*_S_ points are discernible on the chalcogen surfaces owing to their proximity in the interlocked structure.

Following the experimental ^1^H NMR titration insights in acetone-*d*_*6*_ and the MEP of **8·ChB**_**3**_**′**, the halides were hosted in the pseudo-[2]rotaxane cavity, forming three putative convergent ChB interactions with the tellurium donor atoms of **8·ChB**_**3**_**′**. These binding arrangements were investigated in acetone (CPCM^63,64^) using the def2-TZVP(D)^65–67^ basis sets and the M06-2X (refs. ^68,69^) functional (Supplementary Section 5). Naturally, the ChB interactions in the DFT-optimized structures (Fig. 4b) lengthen with the guest anion sizes (Supplementary Table 2). The ChB interactions were further characterized through natural bond orbital (NBO) analysis^70,71^, with the assessment of the second-order perturbation theory energy (*E*^2^) values from the interactions between the lone-pair orbitals of the halides (*n*_X_) and the antibonding orbitals of the C_trz_–Te bonds (Ω*_Ctrz–Te_). In each halide complex, the *E*^2^ energies of the individual ChB bonds (Supplementary Table 2) decrease with the increasing of the corresponding Te···X^−^ distance.

The cation binding properties of the σ-hole-based receptors were studied at the PBE0^72^-D3^73^(BJ)^74^/def2-TZVP^66,75,76^/acetonitrile(SMD^77^) theory level^78^. These studies started with the evaluation of the binding mode preference of **3·Te**^**Me**^ for metal cations (Supplementary Section 5). This preliminary analysis confirmed that the two macrocyclic chalcogen sites, functioning as Lewis bases, can concertedly bind Ag(I), Cu(I) or Tl(I) (Supplementary Fig. 109). Furthermore, in Tl(I)·**3·Te**^**Me**^ the metal repositioned itself over the *tert*-butylphenyl group. Based on these structural features and ^1^H NMR titration evidence, our computational investigation ensued with **8·ChB**_**3**_**′** complexes, having each cation hosted within the ChB pseudo-[2]rotaxane telluromethyl-triazole-decorated cavity. The computed structures of Ag(I) and Cu(I) pseudo-[2]rotaxane complexes display a trigonal coordination environment (Fig. 4c), with the cation interacting with the macrocycle’s and the axle’s TeMe motifs through three equivalent dative covalent bonds. Their distances naturally increase with the cation sizes, with average values of 2.58 Å and 2.74 Å for the Cu(I)···Te and Ag(I)···Te interactions, respectively. Moreover, the C_trz_–Te···M calculated angles around 100° are consistent with the tellurium negative MEP regions being nearly perpendicular to C_trz_–Te bonds (Supplementary Table 9). In Tl(I)·**8·ChB**_**3**_**′** the cation is also bound by the three TeMe units, with an average Tl(I)···Te distance of 3.69 Å. The Tl(I) cation embedded into the **8·ChB**_**3**_**′** binding pocket moved to the *tert*-butylphenyl ring at short Tl(I)···C_ar_ distances of 3.31–3.39 Å, indicating the existence of cation···π interactions (Fig. 4c), as observed in Tl(I)·**3·Te**^**Me**^. This distinct binding mode of Tl(I) is in line with the ^1^H NMR titration results (vide supra).

The M···Te interactions were further characterized through NBO analysis, using *E*^2^ energies derived from the interactions between the lone pair orbitals of the tellurium centres (*n*_Te_) and the lone-vacancy orbitals of the metal cations (LV_M+_) (Supplementary Section 5). The Ag(I) complex of **8·ChB**_**3**_**′** shows a higher average *E*^2^ energy (83.7 kcal mol^−1^) compared with the Cu(I) complex (68.9 kcal mol^−1^) (Supplementary Table 9), in line with the remarkable selectivity experimentally observed for Ag(I). Tl(I) binding of **8·ChB**_**3**_**′** is stabilized by orbital overlapping of LV_Tl+_ with both the *n*_Te_ orbitals of the two Te atoms (average *E*^2^ = 19.1 kcal mol^−1^) and the six ${\rm{C}} {...\over}{\rm{C}}$ aromatic bonds (${\Omega_{{\rm{C}}{...\over}{\rm{C}}}}$) (average *E*^2^ = 2.5 kcal mol^−1^).

The DFT-computed structures for the **8·XB**_**3**_**′** complexes (Supplementary Fig. 112) show the Ag(I) and Cu(I) metal centres coordinated in a trigonal fashion to the three iodine donor atoms of the pseudo-[2]rotaxane, with the average M···I distances increasing with the metal cation’s size (Supplementary Section 5 and Supplementary Table 11). The interaction between the LV_M+_ and the *n*_I_ orbitals results in lower overall *E*^2^ energies for Cu(I) than for Ag(I) (Supplementary Table 11). The M···I interactions are weaker than those of M···Te interactions in **8·ChB**_**3**_**′** (Supplementary Table 9 versus Supplementary Table 11), suggesting that the superior cation affinity of ChB receptors is due to the stronger Lewis-basic character of their telluro-triazole binding units (Supplementary Fig. 114). The DFT-computed structure of Tl(I)·**8·XB**_**3**_**′** also displays three convergent Tl(I)···I bonds and Tl(I)···π interactions (Supplementary Fig. 112).

Overall, the computational studies support the amphoteric character of **8·ChB**_**3**_ and **8·XB**_**3**_ as receptors capable of coordinating either cations or anions through the chalcogen/halogen binding sites. This dual binding ability requires only a subtle spatial reorganization of the σ-hole binding units, as illustrated in Fig. 4d with the overlapping of the chloride and copper(I) **8·ChB**_**3**_**′** complexes, together with the interaction between appropriate NBOs (Ω* or *n* orbitals of the TeMe binding units) and the guest ions’ orbitals (*n* for halides or LV for metal cations). Consistently, the M···Te interactions lead to a more pronounced decrease in the natural population analysis (NPA) charges of the guest metal cations than the M···I interactions (Supplementary Section 5). Furthermore, anion recognition also leads to a variation of the NPA charges of the halides, but to a lesser extent and in the opposite direction (Supplementary Table 14). The nature of the Te···X^−^ and M···Te (**8·ChB**_**3**_**′**) and M···I (**8·XB**_**3**_**′**) interactions was further elucidated through fuzzy bond order (FBO) analysis (Supplementary Table 15). Low covalent character was estimated for the Te···X^−^ interactions in **8·ChB**_**3**_**′**·X^−^ (FBO ≤0.34), whereas the Ag(I) and Cu(I) complexes of **8·ChB**_**3**_**′** and **8·XB**_**3**_**′** afford average FBO values of 1.04 (Ag(I)·**8·ChB**_**3**_**′**), 0.99 (Cu(I)·**8·ChB**_**3**_**′**), 0.94 (Ag(I)·**8·XB**_**3**_**′**) and 0.90 (Cu(I)·**8·XB**_**3**_**′**), more consistent with dative covalent interactions. The Tl(I) complexes feature much weaker Te/I···Tl(I) interactions (FBO ≤0.38).

## Conclusions

This work experimentally and theoretically demonstrates a fundamental proof of concept in host–guest chemistry, wherein anion or cation recognition can be mediated by the same donor atoms in a host molecule. This was achieved by judicious incorporation of amphoteric σ-hole donor motifs into the unique topologically shielded, three-dimensional binding domains of mechanically interlocked rotaxane host systems. Facilitated by utilizing a CuAAC-AMT synthetic strategy, a library of neutral tri- and tetradentate σ-hole donor [2]rotaxane hosts featuring an array of anisotropically polarized tellurium (ChB) and iodine (XB) donor atoms lining their interlocked binding pockets was constructed, including unprecedented all-ChB and mixed ChB/XB [2]rotaxanes for the purpose of demonstrating unrivalled amphoteric charged guest binding behaviour. Comprehensive ^1^H NMR titration and computational DFT studies reveal the respective rotaxane’s Lewis-acidic character of halide anion binding through the formation of ChB/XB σ-hole interactions at the tellurium/iodine donor sites, and concomitant Lewis basic multidentate coordinating capability for selective metal cation chelation via the same chalcogen/halogen donor atoms. Furthermore, charged guest binding strengths and selectivity can be fine-tuned by strategically varying the number, nature and spatial arrangement of the σ-hole donors. This research presents a design principle in coordination and supramolecular host–guest chemistry, where σ-hole donor motifs can be strategically exploited to fabricate novel host structures exhibiting dual Lewis-acidic and Lewis-basic amphoteric properties for charged guest molecular recognition.

## Online content

Any methods, additional references, Nature Portfolio reporting summaries, source data, extended data, supplementary information, acknowledgements, peer review information; details of author contributions and competing interests; and statements of data and code availability are available at 10.1038/s41557-025-01742-x.

## Supplementary information

Supplementary Information Supplementary information.

Supplementary Data 1 DFT-optimized geometries and *Ɛ*_0_ energies.

## Source data

Source Data Fig. 3 Plot data for Fig. 3b.

## Data Availability

All data supporting the findings of this study are available within the article and its Supplementary Information. Source data are provided with this paper.
