# Publisher Correction: Amphoteric chalcogen-bonding and halogen-bonding rotaxanes for anion or cation recognition

**DOI:** 10.1038/s41557-025-01799-8

**Published:** 2025-03-12

**Authors:** Yuen Cheong Tse, Andrew Docker, Igor Marques, Vítor Félix, Paul D. Beer

**Affiliations:** 1 Chemistry Research Laboratory, Department of Chemistry, University of Oxford, Oxford, UK; 2 CICECO – Aveiro Institute of Materials, Department of Chemistry, University of Aveiro, Aveiro, Portugal

**Keywords:** Supramolecular chemistry, Interlocked molecules, Halogen bonding

Correction to: *Nature Chemistry* 10.1038/s41557-025-01742-x, published online 20 February 2025.

In the version of the article initially published, in the fifth paragraph of the “Modelling studies” section, the text “…and the six C ⎓ C aromatic bonds (Ω_C_ ⎓ b_C_)” was incorrect and has now been amended to “and the six ${\rm{C}} {...\over}{\rm{C}}$ aromatic bonds (${\Omega_{{\rm{C}}{...\over}{\rm{C}}}}$)” in the HTML and PDF versions of the article.

