# Supplementary material for: Amphoteric chalcogen-bonding and halogen-bonding rotaxanes for anion or cation recognition
Source: Nat Chem. 2025 Feb 20;17(3):373–81. doi: 10.1038/s41557-025-01742-x (PMC11882458; doi:10.1038/s41557-025-01742-x)
Supplement: Supplementary file 1 — Supplementary information. [file 41557_2025_1742_MOESM1_ESM.pdf]

nature chemistry

Supplementary information https://doi.org/10.1038/s41557-025-01742-x

Amphoteric chalcogen-bonding and halogen-bonding rotaxanes for anion or cation recognition

In the format provided by the authors and unedited

Electronic Supplementary Information (ESI) for

Amphoteric Chalcogen Bonding and Halogen Bonding Rotaxanes for Anion or Cation Recognition

Yuen Cheong Tse[a†], Andrew Docker[a†], Igor Marques[b], Vítor Félix[b], and Paul D. Beer[a*]

[a] Chemistry Research Laboratory, Department of Chemistry, University of Oxford, Mansfield Road, Oxford, UK

[b] CICECO – Aveiro Institute of Materials, Department of Chemistry, University of Aveiro, 3810-193, Aveiro, Portugal

Email: *paul.beer@chem.ox.ac.uk*

[†] These authors contributed equally to the work.

Contents

S1 Synthesis and Characterisation 2

S2 Spectral Characterisation of Novel Compounds 27

S3 ^{1}H NMR Anion Binding Studies 76

S4 ^{1}H NMR Cation Binding Studies 102

S5 DFT Computational Modelling 125

S6 Further Evidence for Iodine···Cation Interaction 143

S7 Acknowledgement 154

S8 References 154

S1 Synthesis and Characterisation

General Information

All solvents and reagents were purchased from commercial suppliers and used as received unless otherwise stated. Dry solvents were obtained by purging with nitrogen and then passing through an MBraun MPSP-800 column. H_2O was de-ionized and micro filtered using a Milli-Q ® Millipore machine. Column chromatography was carried out on Merck® silica gel 60 under a positive pressure of nitrogen. Routine NMR spectra were recorded on either a Varian Mercury 300, a Bruker AVIII 400 or a Bruker AVIII 500 spectrometer with ^{1}H NMR titrations recorded on a Bruker AVIII 500 spectrometer. TBA salts were stored in a vacuum desiccator containing phosphorus pentoxide prior to use. Where mixtures of solvents were used, ratios are reported by volume. Chemical shifts are quoted in parts per million relative to the residual solvent peak. Mass spectra were recorded on a Bruker µTOF spectrometer. Triethylamine was distilled from and stored over potassium hydroxide. Tris[(1-benzyl-1H-1,2,3-triazol-4-yl)methyl]amine (TBTA),[1] **1·I/Te^{Me}/H**,[2] **2,**[3] **5·I**[4] and **6·I**[5] were prepared according to literature procedures.

Synthetic Procedures and Characterisation

Synthesis of Macrocycle Precursors

The target XB/ChB sigma(σ)-hole macrocycles **3·I/Te^{Me}**, and the proto analogue **3·H**, were prepared via a CuAAC-mediated macrocyclisation strategy, in which equimolar quantities of appropriately appended bis-alkyne **1·I/Te^{Me}/H**[2] and bis-azide **2**[3] were reacted in the presence of $[Cu(CH_3CN)_4]PF_6$ and TBTA in anhydrous CH_2Cl_2 over the period of 24–48 hours (Fig. 1). Subsequent aqueous workup with basic EDTA/NH_4OH and purification by column chromatography afforded the target macrocycles in yields in the range of 21–43%.

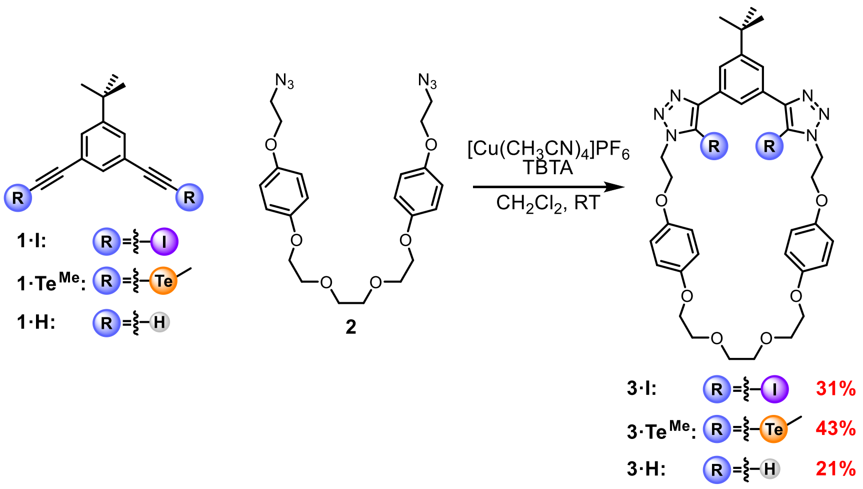

Fig. 1 | Synthetic routes to target macrocycles **3·I/Te^{Me}/H**.

XB Macrocycle (3·I)

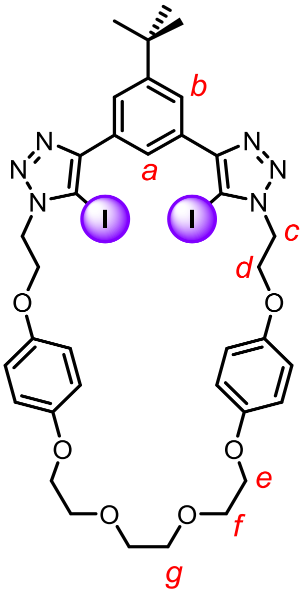

<u>*General Procedure for CuAAC-mediated Macrocyclisation*</u>

$[Cu(CH_3CN)_4]PF_6$ (86 mg, 0.23 mmol, 0.5 equiv) and TBTA (61 mg, 0.12 mmol, 0.25 equiv) were added to dry and degassed CH_2Cl_2 (130 mL), and allowed to stir at room temperature for 15 minutes under N_2. Bis-azide **2** (218 mg, 0.46 mmol, 1 equiv) dissolved in dry and degassed CH_2Cl_2 (10 mL) was added to the Cu(I)-TBTA mixture, followed by a solution of bis(iodoalkyne) **1·I** (200 mg, 0.46 mmol, 1 equiv) in dry and degassed CH_2Cl_2 (10 mL). The reaction mixture was allowed to stir at room temperature in dark overnight. The solvent was removed *in vacuo* and the solid residue was redissolved in CH_2Cl_2 (100 mL) and washed with aqueous basic EDTA (30 mL x 2). The organic layer was dried over $MgSO_4$, filtered and concentrated on the rotary evaporator. Further purification with silica gel column chromatography (60% EtOAc in CH_2Cl_2) afforded the target macrocycle **3·I** as white powder (129 mg, 31%).

^{1}H NMR (400 MHz, $CDCl_3$) δ (ppm): 8.12 (t, J = 1.6 Hz, 1H; *a*), 7.96 (d, J = 1.6 Hz, 2H; *b*), 6.78 (d, J = 9.2 Hz, 4H; *hydroquinone ArH*), 6.71 (d, J = 9.2 Hz, 4H; *hydroquinone ArH*), 4.86 (t, J = 4.9 Hz, 4H; *c*), 4.43 (t, J = 4.9 Hz, 4H; *d*), 4.02 (t, J = 4.8 Hz, 4H; *e*), 3.78 (t, J = 4.8 Hz, 4H; *f*), 3.67 (s, 4H; *g*), 1.43 (s, 9H; *tert*-butyl *H*).

^{13}C NMR (126 MHz, $CDCl_3$) δ (ppm): 153.68, 152.41, 152.23, 150.53, 130.29, 125.61, 124.42, 78.73, 70.97, 69.85, 68.35, 67.74, 50.16, 35.30, 31.52, 29.85, 1.17.

HRMS (ESI +ve) m/z: 907.1168 ($[M+H]^+$, $C_{36}H_{41}O_6N_6I_2$ requires 907.1171)

ChB Macrocycle (3·Te^{Me})

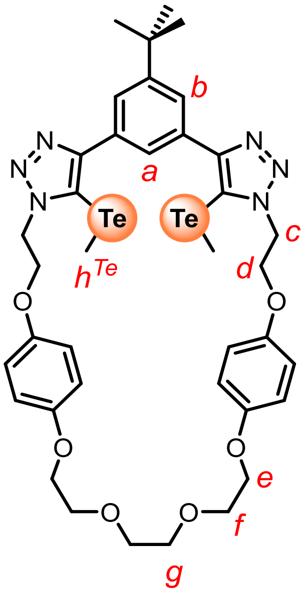

Following the general procedure for CuAAC-mediated macrocyclisation between bis(telluromethylalkyne) **1·Te^{Me}** (250 mg, 0.54 mmol, 1 equiv) and bis-azide **2** (254 mg, 0.54 mmol, 1 equiv), the crude was purified by silica gel column chromatography (60% EtOAc in CH_2Cl_2) to give the target macrocycle **3·Te^{Me}** as white powder (218 mg, 43%).

^{1}H NMR (400 MHz, $CDCl_3$) δ (ppm): 8.41 (t, J = 1.6 Hz, 1H; *a*), 7.92 (d, J = 1.6 Hz, 2H; *b*), 6.80 (d, J = 9.2 Hz, 4H; *hydroquinone ArH*), 6.74 (d, J = 9.2 Hz, 4H; *hydroquinone ArH*), 5.00 (t, J = 4.8 Hz, 4H; *c*), 4.36 (t, J = 4.8 Hz, 4H; *d*), 4.03 (t, J = 4.8 Hz, 4H; *e*), 3.81 (t, J = 4.8 Hz, 4H; *f*), 3.70 (s, 4H; *g*), 1.85 (s, 6H; h^{Te}), 1.42 (s, 9H, *tert*-butyl *H*).

^{13}C NMR (126 MHz, $CDCl_3$) δ (ppm): 153.80, 153.46, 152.31, 152.02, 131.58, 125.78, 124.78, 115.95, 115.74, 102.35, 71.00, 69.83, 68.38, 68.15, 50.44, 35.20, 31.52, -12.69.

HRMS (ESI +ve) m/z: 941.1650 ($[M+H]^+$, $C_{38}H_{47}O_6N_6Te_2$ requires 941.1658)

HB Macrocycle (3·H)

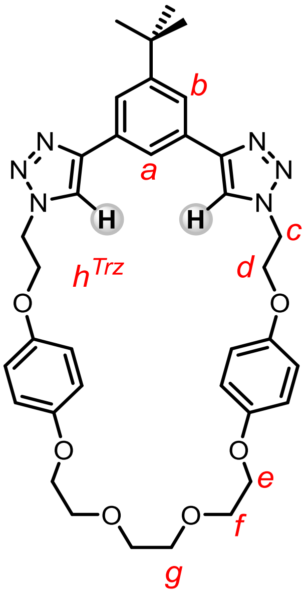

Following the general procedure for CuAAC-mediated macrocyclisation between bis(iodoalkyne) **1·H** (200 mg, 1.10 mmol, 1 equiv) and bis-azide **2** (518 mg, 1.10 mmol, 1 equiv), the crude was purified by silica gel column chromatography (15:15:70 EtOAc/Acetone/CH_2Cl_2) to give the target macrocycle **3·H** as white powder (154 mg, 21%).

^{1}H NMR (400 MHz, acetone-d_6) δ (ppm): 8.53 (s, 2H; h^{Trz}), 8.09 (d, J = 1.6 Hz, 2H; *b*), 8.00 (t, J = 1.6 Hz, 1H; *a*), 6.89 (d, J = 9.2 Hz, 4H; *hydroquinone ArH*), 6.83 (d, J = 9.2 Hz, 4H; *hydroquinone ArH*), 4.86 (t, J = 4.6 Hz, 4H; *c*), 4.42 (t, J = 4.6 Hz, 4H; *d*), 4.03 (t, J = 4.6 Hz, 4H; *e*), 3.77 (t, J = 4.6 Hz, 4H; *f*), 3.63 (s, 4H; *g*), 1.42 (s, 9H; *tert*-butyl *H*).

^{13}C NMR (126 MHz, acetone-d_6) δ (ppm): 154.69, 153.45, 153.07, 148.16, 132.59, 122.51, 121.23, 116.64, 116.48, 71.54, 70.34, 69.02, 68.33, 50.69, 35.53, 31.66, 26.22.

HRMS (ESI +ve) m/z: 655.3234 ($[M+H]^+$, $C_{36}H_{43}O_6N_6$ requires 655.3239).

Synthesis of Alkynyl Axle Precursors

The iodo-alkynyl stopper **5·I**[4] was obtained by iodination of proto-alkyne **4·H** using *N*-iodomorpholine hydroiodide and CuI in THF, followed by aqueous work-up (Fig. 2). The novel tellurium functionalised alkyne coupling partners **5·Te**$^{\mathbf{R'}}$ (R' = Me, Ph, pCF3 (*para*-CF_3-Ph)) utilised in the AMT methodology were synthesised via treatment of a 1:1 CH_2Cl_2/CH_3CN solution of stopper alkyne **4·H** with freshly pulverised $AgNO_3$. Excluding light, the addition of a catalytic amount of triethylamine immediately induced the precipitation of the stopper Ag(I) acetylide. A THF suspension of this isolable organometallic stopper derivative was reacted with either an alkyl or aromatic tellurium(II) bromide species (R'TeBr), generated by exposing the parent diorgano ditelluride (R'_2Te_2) to a CH_2Cl_2 solution of Br_2, to afford the respective organotellurium appended stopper derivative.[2,6–8] Notably, this procedure provides facile access to a wide variety tellurium appended alkyne stopper precursors, limited only by accessibility of the corresponding ditelluride precursor.

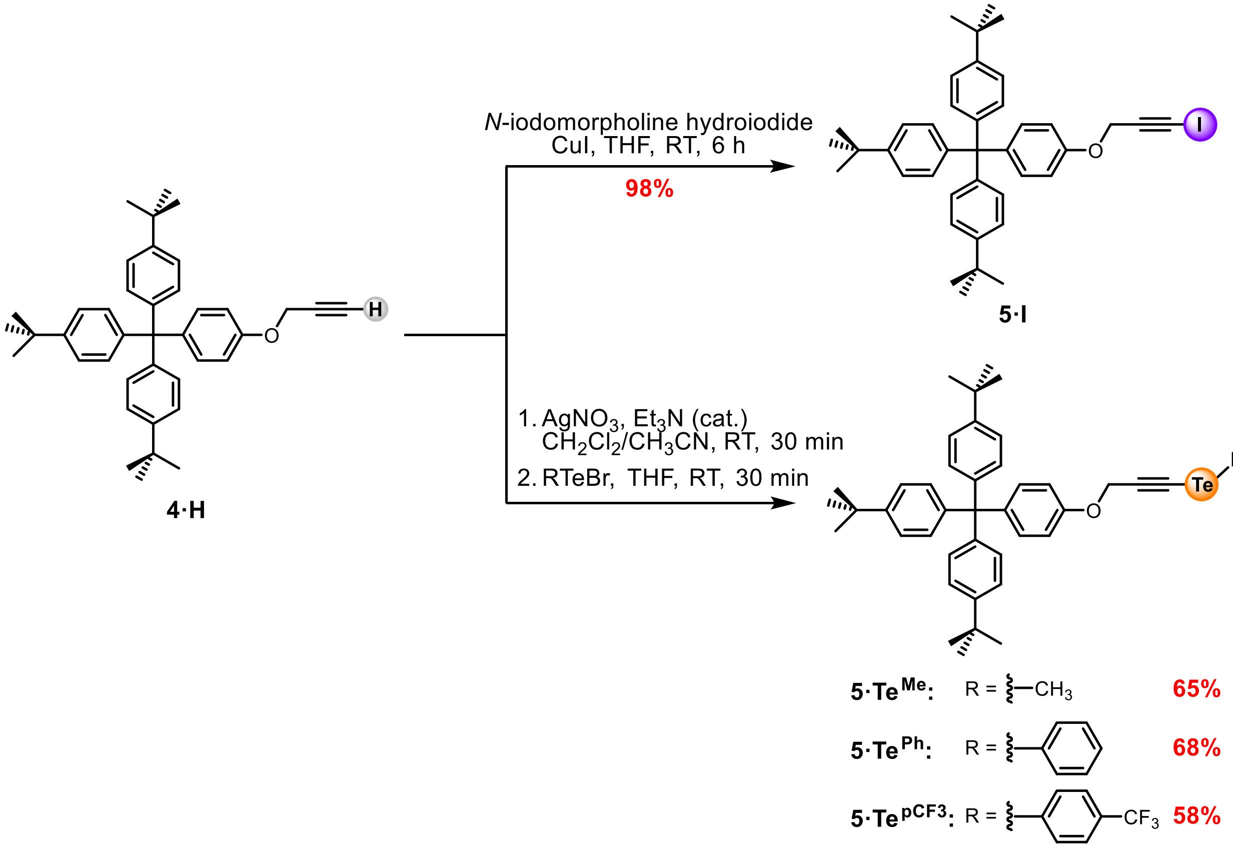

Fig. 2 | Synthetic route to target alkynyl axle precursors **5·I/Te**$^{\mathbf{Me/Ph/pCF3}}$.

Stopper telluromethyl-alkyne (5·Te^{Me})

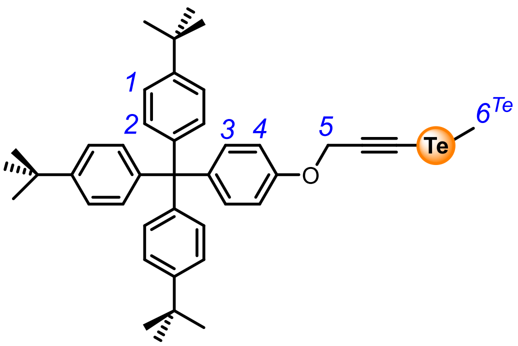

<u>*General Procedure for Stopper Telluroalkyne Synthesis*</u>

$AgNO_3$ (158 mg, 0.98 mmol, 1.01 equiv) and catalytic amount of freshly distilled Et_3N (0.1 mL) were added to a solution of stopper proto-alkyne **4·H** (500 mg, 0.92 mmol, 1 equiv) dissolved in 1:1 CH_2Cl_2:CH_3CN (40 mL). The mixture was stirred at room temperature for 30 minutes in dark to generate the corresponding stopper silver acetylide. The solvent was removed *in vacuo* and the isolated acetylide was suspended in dry THF (18 mL). In a separate flask, $(CH_3)_2Te_2$ (263 mg, 0.92 mmol, 1 equiv) was dissolved in dry THF (6 mL), to which was added 1.0 M Br_2 solution in CH_2Cl_2 (0.92 mL) at 0 °C to generate the corresponding CH_3TeBr. After warming to room temperature, the CH_3TeBr solution was added to the stopper silver acetylide suspension dropwise and the reaction mixture was stirred for 30 minutes. The crude was then filtered through celite, and concentrated on rotary evaporator. Product was purified by silica gel column chromatography (20% CH_2Cl_2 in *n*-hexane) to give the product as white powder (410 mg, 65%).

^{1}H NMR (400 MHz, acetone-d_6) δ (ppm): 7.32 (d, J = 8.6 Hz, 6H; *2*), 7.12 (d, J = 8.6 Hz, 6H; *1*), 7.11 (d, J = 9.0 Hz, 2H; *3*), 6.90 (d, J = 9.0 Hz, 2H; *4*), 4.94 (s, 2H; *5*), 2.11 (s, 3H; 6^{Te}), 1.30 (s, 27H; *tert*-butyl H).

^{13}C NMR (126 MHz, $CDCl_3$) δ (ppm): 155.78, 148.48, 144.22, 140.50, 132.37, 130.89, 124.19, 113.61, 106.44, 63.23, 57.34, 43.28, 34.44, 31.53, -14.92.

Stopper Phenyl Telluroalkyne (5·Te^{Ph})

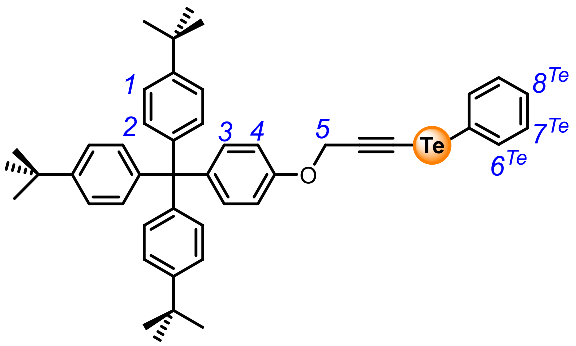

Following the general procedure for stopper telluroalkyne synthesis using stopper proto-alkyne **4·H** (500 mg, 0.92 mmol, 1 equiv) and Ph_2Te_2 (377 mg, 0.92 mmol, 1 equiv) as starting materials, the crude was purified with silica gel column chromatography (20% CH_2Cl_2 in *n*-hexane) to give the target **5·Te^{Ph}** as white powder (468 mg, 68%).

^{1}H NMR (400 MHz, acetone-d_6) δ (ppm): 7.76 – 7.70 (m, 2H; 6^{Te}), 7.32 (d, J = 8.6 Hz, 6H; *2*), 7.29 – 7.24 (m, 1H; 8^{Te}), 7.24 – 7.17 (m, 2H; 7^{Te}), 7.16 – 7.08 (m, 8H; *3* + *1*), 6.94 (d, J = 9.0 Hz, 2H; *4*), 5.05 (s, 2H; *5*), 1.30 (s, 27H; *tert*-butyl H).

^{13}C NMR (126 MHz, $CDCl_3$) δ (ppm): 155.66, 148.51, 144.21, 140.57, 135.64, 132.39, 130.91, 129.88, 128.20, 124.20, 113.66, 112.58, 110.27, 63.24, 57.28, 46.76, 34.44, 31.54.

Stopper *para*-Trifluoromethyl Telluroalkyne (5·Te^{pCF3})

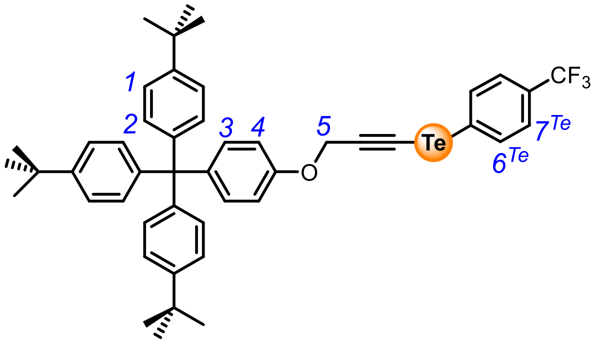

Following the general procedure for stopper telluroalkyne synthesis using stopper proto-alkyne **4·H** (500 mg, 0.92 mmol, 1 equiv) and (*para*-CF_3-Ph)$_2$Te$_2$ (502 mg, 0.92 mmol, 1 equiv) as starting materials, the crude was purified with silica gel column chromatography (10% CH_2Cl_2 in *n*-hexane) to give the target **5·Te^{pCF3}** as white powder (420 mg, 56%).

^{1}H NMR (400 MHz, acetone-d_6) δ (ppm): 7.98 (d, J = 8.0 Hz, 2H; 7^{Te}), 7.55 (d, J = 8.0 Hz, 2H; 6^{Te}), 7.31 (d, J = 8.7 Hz, 6H; *2*), 7.17 – 7.08 (m, 8H; *3* + *1*), 6.96 (d, J = 9.0 Hz, 2H; *4*), 5.10 (s, 2H; *5*), 1.30 (s, 27H; *tert*-butyl H).

^{13}C NMR (126 MHz, $CDCl_3$) δ (ppm): 155.62, 148.57, 144.17, 140.82, 134.81, 132.46, 130.89, 126.34 (q, $^4J_{C-F}$ = 3.9 Hz), 124.22, 124.05 (q, $^1J_{C-F}$ = 273 Hz), 118.47, 113.62, 111.65, 63.26, 57.26, 45.86, 34.45, 31.52, 22.49.

^{19}F NMR (377 MHz, $CDCl_3$) δ (ppm): −62.77.

Synthesis of Azido Axle Precursors

To facilitate access to target higher-order tetradentate XB and ChB [2]rotaxanes, azido precursors **6·I/Te^{Me}** consisting of a pre-formed iodo-/telluromethyl-triazole, were prepared by reacting stopper alkynes **5·I/Te^{Me}** with excess 1,3-diazidopropane[9] under standard CuAAC conditions in yields of 85–90% (Fig. 3).[5]

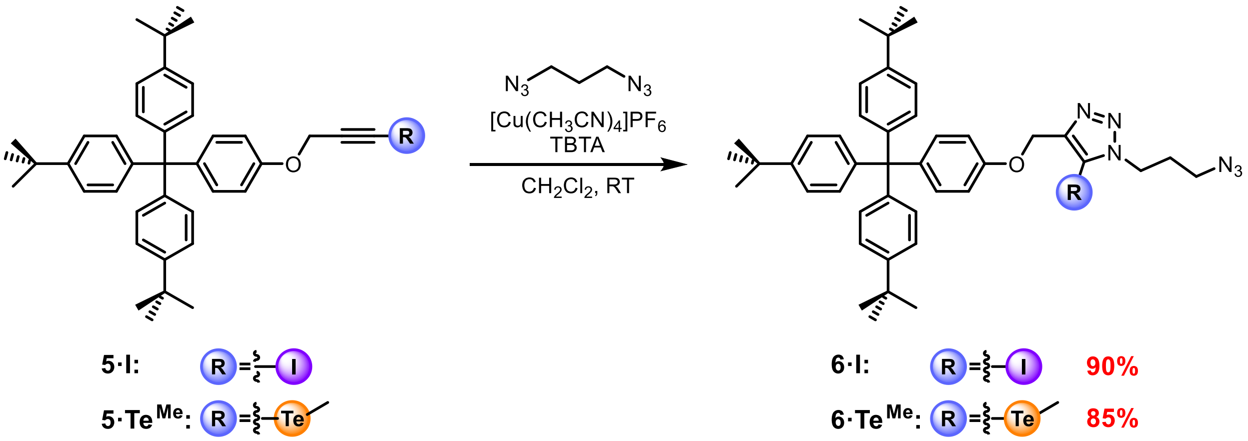

Fig. 3 | Synthetic route to target azido axle precursors (**6·I/Te^{Me}**).

Stopper Telluromethyl-triazole-functionalised Azide (6·Te^{Me})

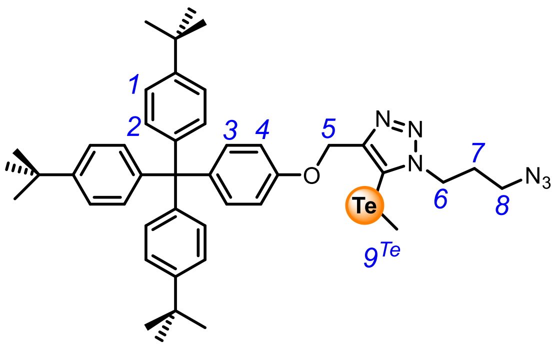

$[Cu(CH_3CN)_4]PF_6$ (54 mg, 0.15 mmol, 0.5 equiv) and TBTA (78 mg, 0.15 mmol, 0.5 equiv) were added to dry and degassed CH_2Cl_2 (5 mL) and the mixture was stirred at room temperature for 20 minutes. CH_2Cl_2 solutions of 1,3-diazido-propane (295 mg, 2.34 mmol, 8 equiv) and stopper telluromethyl-alkyne **5·Te^{Me}** (200 mg, 0.29 mmol, 1 equiv) were added sequentially. The reaction mixture was left stirring at room temperature in dark overnight. The crude was washed with basic EDTA (30 mL) and deionised water (30 mL × 3). The organic layer was dried over anhydrous MgSO4, filtered and concentrated *in vacuo*. Further purification with silica gel column chromatography (60% CH_2Cl_2 in *n*-hexane) gave the product as white powder (201 mg, 85%).

^{1}H NMR (600 MHz, acetone-d_6) δ (ppm): (d, *J* = 8.4 Hz, 6H; *2*), 7.13 (d, *J* = 8.4 Hz, 6H; *1*), 7.12 (d, *J* = 8.9 Hz, 2H; *3*), 6.99 (d, *J* = 8.9 Hz, 2H; *4*), 5.21 (s, 2H; *5*), 4.68 (t, *J* = 7.0 Hz, 2H; *6*), 3.48 (t, *J* = 6.5 Hz, 2H; *8*), 2.23 (q, *J* = 6.7Hz, 2H; *7*), 2.09 (s, 3H; *9^{Te}*), 1.31 (s, 27H; *tert*-butyl H).

^{13}C NMR (151 MHz, acetone-d_6) δ (ppm): 157.59, 152.05, 149.22, 145.24, 140.69, 132.87, 131.47, 125.08, 114.53, 106.03, 63.95, 63.76, 49.13, 49.03, 34.88, 31.66, 30.59, -13.25.

HRMS (ESI +ve) m/z: 811.3474 ($[M+H]^+$, $C_{44}H_{55}N_6OTe$ requires 811.3476).

Synthesis of Tridentate and Tetradentate [2]Rotaxanes

Synthetic routes to the target [2]rotaxane series via CuAAC-AMT methodology is shown in Fig. 4. In a typical reaction, an equimolar mixture of the macrocycle and $[Cu(CH_3CN)_4]PF_6$ was first stirred at room temperature in anhydrous CH_2Cl_2. A five-fold excess of the appropriate stopper azide and alkyne precursors were added, and the reaction mixture was left stirring under ambient conditions for 48 hours. If after which time TLC and mass spectrometry analysis of the crude mixture indicated the persistence of the alkyne and azide precursors, an additional equivalent of Cu(I) was added, and the reaction was allowed to stir for an extended period of 24 hours. This process of metal catalyst-addition was repeated until complete consumption of the CuAAC coupling partners was observed. Subsequently, the reaction mixture was subjected to an EDTA/NH4OH aqueous workup procedure, and the crude was purified by preparative TLC to afford the series of rotaxanes in variable yields. All [2]rotaxanes were characterised by ^{1}H and ^{13}C NMR spectroscopy, and high-resolution ESI mass spectrometry. The individual synthetic conditions for the [2]rotaxane series are summarised in Table 1.

Inspection of Table 1 details several key synthetic milestones and noteworthy observations warranting further discussion. Importantly, courtesy of the modular AMT approach, unprecedented access to all-ChB and mixed XB/ChB [2]rotaxanes containing up to four σ-hole triazole-based donors is achieved (Table 1, entries 1, 4–10). We also demonstrate that further elaboration of the ChB binding cavity is achievable by employing aryl-tellurium functionalised stopper precursors, as is demonstrated by the successful isolation of **8·XB$_2$ChB^{Ph}** and **8·XB$_2$ChB^{pCF3}**. It is apparent that as the steric demand of either the macrocyclic or axle component precursors increases, the rate of mechanical bond formation is considerably attenuated. This is perhaps most obviously demonstrated by the synthesis of **9·ChB$_4$** and **9·XB$_2$ChB$_2$** (Table 1, entries 5 and 7), which required a considerable 168 hours of reaction time. Indeed, another observation made during the synthesis which alludes to the Cu(I) ligating properties of Te donors is the requirement of a 5-fold excess of $[Cu(CH_3CN)_4]PF_6$ to achieve appreciable conversion, which is understandable if the addition of telluro-triazole-containing stopper precursors (**5·Te^{Me}** and **6·Te^{Me}**) effectively competes with the macrocycle for Cu(I) coordination. Interestingly, a minor but persistently observed side product in the synthesis of telluro-triazole-functionalised [2]rotaxanes under these AMT conditions were de-tellurated proto-triazole derivatives. This presumably occurs via an oxidative addition-type mechanism by insertion of Cu(I) into the triazole-carbon-tellurium bond and subsequent protonation of the triazolide intermediate.[10] Indeed, the lability of the C–Te bond was further evidenced by the

thwarted attempts to prepare the XB/ChB mixed [2]rotaxane **8·ChB$_2$XB**, comprised of the ChB macrocycle and XB-donor containing axle (Table 1, entry 2). Under analogous CuAAC-AMT reaction conditions performed with ChB macrocycle **3·Te^{Me}**, iodo-functionalised stopper alkyne **5·I** and stopper azide **7**, ^{1}H NMR and ESI-MS analysis revealed after 2 days no interlocked product was formed but complete conversion of **3·Te^{Me}** to the iodo-triazole functionalised macrocycle **3·I** was observed.

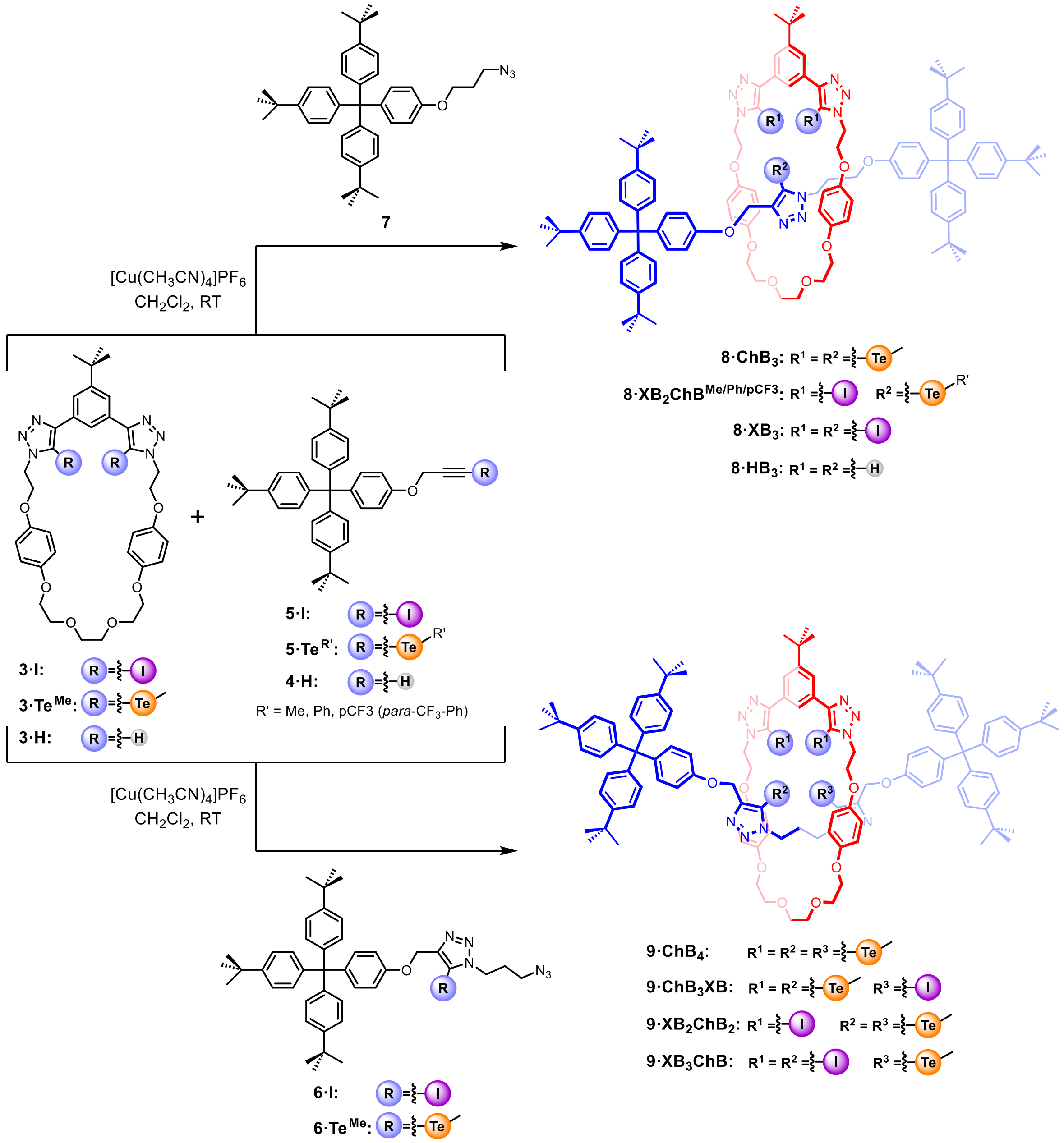

Fig. 4 | Synthetic routes to target [2]rotaxanes.

Table 1 | Reaction conditions and yields for the synthesis of σ-hole [2]rotaxanes by CuAAC-AMT procedure.[a]

Entry	[2]Rotaxanes	Macrocycle	Alkyne	Azide	Cu(I)[b]	Time	Yield[c]
1	**8·ChB$_3$**	**3·Te^{Me}**	**5·Te^{Me}**	**7**	1 equiv	2 d	24%
2	**8·ChB$_2$XB**	**3·Te^{Me}**	**5·I**	**7**	1 equiv	2 d	—[d]
3[e]	**8·XB$_3$**	**3·I**	**5·I**	**7**	3 equiv	5 d	31%
4	**8·XB$_2$ChB^{Me}**	**3·I**	**5·Te^{Me}**	**7**	1 equiv	2 d	25%
5	**9·ChB$_4$**	**3·Te^{Me}**	**5·Te^{Me}**	**6·Te^{Me}**	5 equiv	7 d	18%
6	**9·ChB$_3$XB**	**3·Te^{Me}**	**5·Te^{Me}**	**6·I**	1 equiv	2 d	23%
7	**9·XB$_2$ChB$_2$**	**3·I**	**5·Te^{Me}**	**6·Te^{Me}**	5 equiv	7 d	10%
8	**9·XB$_3$ChB**	**3·I**	**5·Te^{Me}**	**6·I**	1 equiv	2 d	17%
9	**8·XB$_2$ChB^{Ph}**	**3·I**	**5·Te^{Ph}**	**7**	2 equiv	3 d	20%
10	**8·XB$_2$ChB^{pCF3}**	**3·I**	**5·Te^{pCF3}**	**7**	3 equiv	4 d	13%
11	**8·HB$_3$**	**3·H**	**4·H**	**7**	1 equiv	2 d	18%

[a]Ratio of macrocycle:alkyne:azide = 1:5:5. Reactions were carried out in anhydrous CH_2Cl_2 at room temperature with concentration of 5 mM with respect to the macrocycle. [b]$[Cu(CH_3CN)_4]PF_6$ was added portionwise at 1 equivalent. [c]Isolated yield after chromatographic purification. [d]No rotaxane formed. [e]Reaction was performed at 35 °C.

Tridentate All-ChB [2]Rotaxane ($8\cdot ChB_3$)

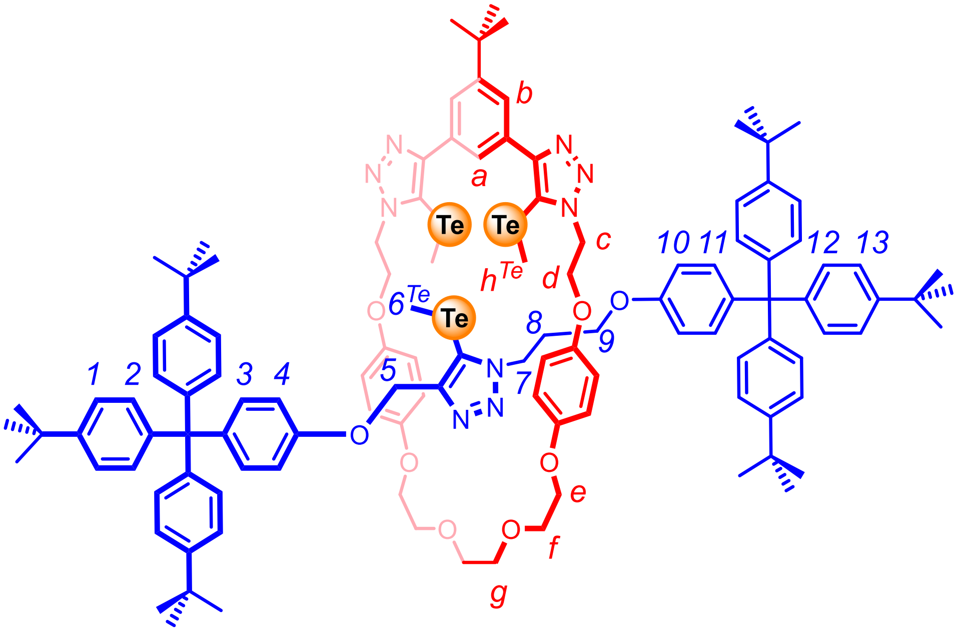

General Procedure for CuAAC-AMT Synthesis of [2]Rotaxanes

Macrocycle **3·Te**$^{\mathbf{Me}}$ (10 mg, 0.011 mmol, 1 equiv) and $[Cu(CH_3CN)_4]PF_6$ (4 mg, 0.011 mmol, 1 equiv) were dissolved in dry and degassed CH_2Cl_2 (0.7 mL) in a sealed vial, and the mixture was allowed to stir at room temperature under N_2 for 30 minutes. Stopper azide **7** (31 mg, 0.053 mmol, 5 equiv) and stopper alkyne **5·Te**$^{\mathbf{Me}}$ (36 mg, 0.053 mmol, 5 equiv), each dissolved in dry and degassed CH_2Cl_2 (0.7 mL), were sequentially added to the vial. The reaction mixture was left stirring at room temperature for 2 days, if after which time TLC analysis of the crude indicated the persistence of azide/alkyne precursors, additional portion of $[Cu(CH_3CN)_4]PF_6$ (4 mg, 0.011 mmol, 1 equiv) was added. This process was repeated until complete consumption of azide/alkyne was observed. Afterwards, the crude was diluted with CH_2Cl_2 (20 mL) and washed with aqueous 0.02 M NH_4OH/EDTA (20 mL), dried with $MgSO_4$, filtered and concentrated on rotary evaporator. Further purification with preparative TLC (10% EtOAc in CH_2Cl_2) afforded the target tridentate [2]rotaxane **8·ChB₃** as white powder (5.7 mg, 24%).

^{1}H NMR (500 MHz, acetone-*d*$_6$) δ (ppm): 8.62 (t, *J* = 1.6 Hz, 1H; *a*), 8.09 (d, *J* = 1.6 Hz, 2H; *b*), 7.32 (m, 12H; *2, 12*), 7.15 (m, 12H; *1, 13*), 7.08 (d, *J* = 8.8 Hz, 2H; *3*), 7.02 (d, *J* = 8.8 Hz, 2H; *11*), 6.81 (d, *J* = 8.8 Hz, 2H; *4*), 6.50 (d, *J* = 8.8 Hz, 2H; *10*), 6.47 (d, *J* = 9.1 Hz, 4H; hydroquinone ArH), 6.39 (d, *J* = 9.1 Hz, 4H; hydroquinone ArH), 5.01 (t, *J* = 4.7 Hz, 4H; *c*), 4.85 (s, 2H; *5*), 4.29 (t, *J* = 4.7 Hz, 4H; *d*), 4.16 (t, *J* = 7.4 Hz, 2H; *7*), 3.84 (t, *J* = 5.0 Hz, 4H;

e), 3.60 (t, *J* = 5.0 Hz, 4H; *f*), 3.54 – 3.47 (m, 6H; *g*, *9*), 1.98 (s, 6H; h^{Te}), 1.56 (s, 3H; 6^{Te}), 1.42 (s, 9H; macrocycle ^{t}Bu), 1.30 (s, 54H; axle ^{t}Bu).

^{13}C NMR (151 MHz, acetone-d_6) δ (ppm): 157.44, 157.39, 154.06, 153.28, 153.03, 151.71, 151.64, 149.09, 149.05, 145.49, 145.40, 140.27, 139.96, 132.93, 132.66, 132.59, 131.48, 125.79, 125.36, 125.11, 125.10, 116.22, 115.93, 114.50, 114.23, 105.58, 103.20, 71.32, 70.13, 68.79, 68.54, 65.18, 63.96, 63.31, 54.96, 51.30, 48.64, 35.70, 34.90, 34.89, 31.79, 31.72, 31.70, 30.34, -12.66, -13.05.

HRMS (ESI +ve) m/z: 2210.8261 ($[M]^{+\bullet}$, $C_{119}H_{143}O_8N_9Te_3$ requires 2210.8228).

Tridentate All-XB [2]Rotaxane (8·XB$_3$)

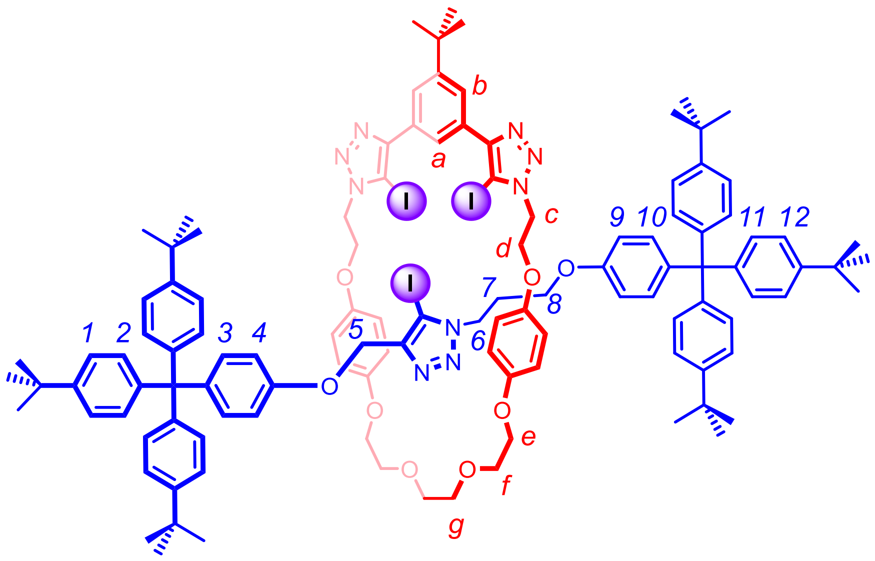

Following the general procedure for CuAAC-AMT synthesis of [2]rotaxanes between macrocycle **3·I** (10 mg, 0.011 mmol, 1 equiv), stopper azide **7** (32 mg, 0.055 mmol, 5 equiv) and stopper alkyne **5·I** (37 mg, 0.055 mmol, 5 equiv), the product was purified by preparative TLC (acetone/EtOAc/CH_2Cl_2 2:6:92) to give the target tridentate [2]rotaxane **8·XB$_3$** as white powder (7.4 mg, 31%).

^{1}H NMR (400 MHz, acetone-*d$_6$*) δ (ppm): 8.32 (t, *J* = 1.6 Hz, 1H; *a*), 8.02 (d, *J* = 1.6 Hz, 2H; *b*), 7.37 – 7.27 (m, 12H; *2, 11*), 7.18 – 7.11 (m, 12H; *1, 12*), 7.09 (d, *J* = 8.9 Hz, 2H; *3*), 6.98 (d, *J* = 8.9 Hz, 2H; *10*), 6.86 (d, *J* = 8.9 Hz, 2H; *4*), 6.45 (apparent s, 8H; *hydroquinone ArH*), 6.36 (d, *J* = 8.9 Hz, 2H; *9*), 4.88 (t, *J* = 4.7 Hz, 4H; *c*), 4.82 (s, 2H; *5*), 4.42 – 4.23 (m, 4H; *d*), 3.93 – 3.79 (m, 6H; *6, e*) 3.61 (t, *J* = 4.7 Hz, 4H; *f*), 3.52 (s, 4H; *g*), 3.35 (t, *J* = 5.9 Hz, 2H; *8*), 1.70 (q, *J* = 5.9 Hz, 2H; *7*), 1.42 (s, 9H; *macrocycle tert*-butyl *H*), 1.31 (s, 57H; axle *tert*-butyl *H*).

^{13}C NMR (151 MHz, acetone-*d$_6$*) δ (ppm): 157.38, 157.27, 154.07, 153.11, 152.29, 150.40, 149.15, 149.06, 147.68, 145.47, 145.32, 140.58, 139.98, 132.77, 132.64, 131.95, 131.53, 131.49, 125.38, 125.27, 125.12, 125.11, 116.18, 116.06, 114.54, 114.07, 82.81, 80.22, 71.41, 70.25, 68.56, 68.21, 64.81, 63.96, 62.22, 50.96, 48.16, 35.75, 34.91, 34.89, 31.79, 31.74, 31.70, 23.34.

HRMS (ESI +ve) m/z: 2163.7530 ($[M+H]^+$, $C_{116}H_{135}O_8N_9I_3$ requires 2163.7594).

Tridentate Mixed XB/ChB [2]Rotaxane ($8\cdot XB_2ChB^{Me}$)

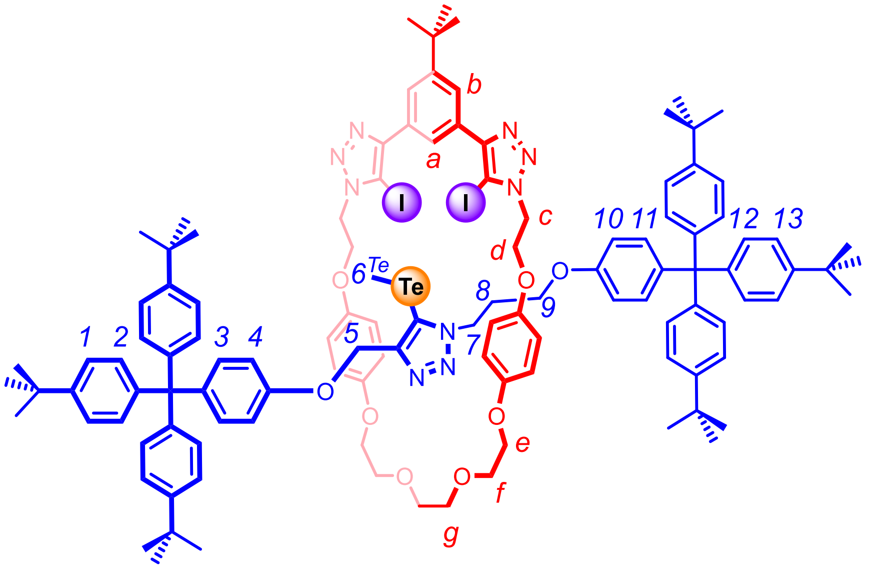

Following the general procedure for CuAAC-AMT synthesis of [2]rotaxanes between macrocycle **3·I** (10 mg, 0.011 mmol, 1 equiv), stopper azide **7** (32 mg, 0.055 mmol, 5 equiv) and stopper alkyne **$5\cdot Te^{Me}$** (38 mg, 0.055 mmol, 5 equiv), the product was purified by preparative TLC (10% EtOAc in CH_2Cl_2) to give the target tridentate [2]rotaxane **$8\cdot XB_2ChB^{Me}$** as white powder (6.0 mg, 25%).

^{1}H NMR (600 MHz, acetone-d_6) δ (ppm): 8.25 (t, J = 1.6 Hz, 1H; *a*), 8.01 (d, J = 1.6 Hz, 2H; *b*), 7.36 – 7.27 (m, 12H; *2, 12*), 7.14 – 7.10 (m, 12H; *1, 13*), 7.06 (d, J = 8.8 Hz, 2H; *3*), 6.93 (d, J = 8.8 Hz, 2H; *11*), 6.81 (d, J = 8.8 Hz, 2H; *4*), 6.49 (d, J = 8.8 Hz, 4H; *hydroquinone ArH*), 6.42 (d, J = 8.8 Hz, 4H; *hydroquinone ArH*), 6.31 (d, J = 8.8 Hz, 2H; *10*), 4.95 (s, 2H; *5*), 4.89 (t, J = 4.9 Hz, 4H; *c*), 4.34 (t, J = 5.0 Hz, 4H; *d*), 4.23 (t, J = 6.5 Hz, 2H; *7*), 3.88 – 3.80 (m, 4H; *e*), 3.60 (t, J = 5.0 Hz, 4H; *f*), 3.54 – 3.48 (m, 4H; *g*), 3.39 (t, J = 6.5 Hz, 2H; *9*), 1.85 (q, J = 6.5 Hz, 2H; *8*), 1.65 (s, 3H; 6^{Te}), 1.42 (s, 9H; macrocycle *tert*-butyl H), 1.31 (s, 54H; axle *tert*-butyl H).

^{13}C NMR (151 MHz, acetone-d_6) δ (ppm): 157.43, 157.27, 154.09, 153.11, 152.24, 151.69, 150.45, 149.13, 149.05, 145.48, 145.36, 140.37, 139.82, 132.73, 132.64, 131.92, 131.56, 131.49, 125.39, 125.11, 116.17, 116.11, 114.53, 114.00, 105.73, 80.24, 71.35, 70.20, 68.54, 68.23, 64.85, 63.96, 63.93, 63.42, 50.96, 48.57, 34.91, 34.90, 31.78, 31.75, 31.71, 30.35, 23.34, 14.36, -12.87.

HRMS (ESI +ve) m/z: 2178.7774 ($[M]^{+\bullet}$, $C_{117}H_{137}O_8N_9TeI_2$ requires 2178.7752).

Tridentate Mixed XB/ChB [2]Rotaxane (8·XB$_2$ChB^{Ph})

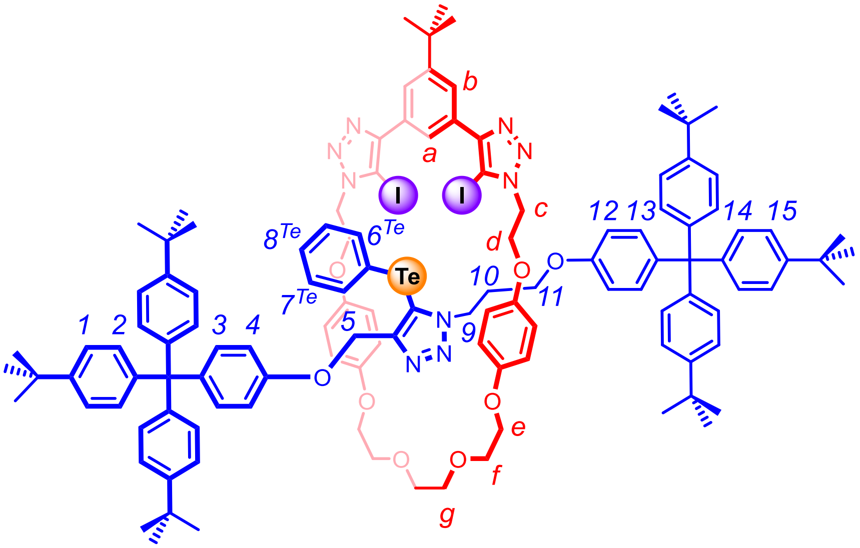

Following the general procedure for CuAAC-AMT synthesis of [2]rotaxanes between macrocycle **3·I** (10 mg, 0.011 mmol, 1 equiv), stopper azide **7** (32 mg, 0.055 mmol, 5 equiv) and stopper alkyne **5·Te^{Ph}** (41 mg, 0.055 mmol, 5 equiv), the product was purified by preparative TLC (10% EtOAc in CH_2Cl_2) to give the target tridentate [2]rotaxane **8·XB$_2$ChB^{Ph}** as white powder (4.9 mg, 20%).

^{1}H NMR (600 MHz, acetone-*d$_6$*) δ (ppm): 8.26 (t, *J* = 1.6 Hz, 1H; *a*), 8.00 (d, *J* = 1.6 Hz, 2H; *b*), 7.35 (d, *J* = 7.5 Hz, 2H; 7^{Te}), 7.34 – 7.29 (m, 12H; *2, 14*), 7.14 – 7.10 (m, 13H; *1, 15, 8^{Te}*), 7.04 (d, *J* = 7.5 Hz, 2H; *6^{Te}*), 7.02 (d, *J* = 8.9 Hz, 4H; *3*), 6.94 (d, *J* = 8.9 Hz, 2H; *13*), 6.76 (d, *J* = 8.9 Hz, 2H; *4*), 6.49 (d, *J* = 8.9 Hz, 4H; *hydroquinone ArH*), 6.40 (d, *J* = 8.9 Hz, 4H; *hydroquinone ArH*), 6.32 (d, *J* = 8.9 Hz, 4H; *10*), 4.99 (s, 2H; *5*), 4.88 (t, *J* = 4.9 Hz, 4H; *c*), 4.42 – 4.29 (m, 4H; *d*), 4.22 (t, *J* = 6.5 Hz, 2H; *9*), 3.86 – 3.79 (m, 4H; *e*), 3.59 (t, *J* = 5.0 Hz, 4H; *f*), 3.54 – 3.48 (m, 4H; *g*), 3.38 (t, *J* = 6.5 Hz, 2H; *11*), 1.77 (q, *J* = 6.5 Hz, 2H; *10*), 1.41 (s, 9H; *macrocycle tert*-butyl *H*), 1.31 (s, 54H; axle *tert*-butyl *H*).

^{13}C NMR (151 MHz, acetone-*d$_6$*) δ (ppm): 157.37, 157.27, 154.13, 153.10, 152.23, 150.40, 149.11, 149.04, 145.48, 145.36, 140.33, 139.87, 137.46, 132.69, 132.61, 131.85, 131.54, 131.49, 130.54, 128.94, 125.36, 125.11, 125.10, 116.26, 116.23, 115.17, 114.46, 114.09, 108.19, 80.19, 71.33, 70.16, 68.56, 68.25, 65.02, 63.94, 63.10, 50.92, 48.81, 35.72, 34.91, 34.89, 31.77, 31.74, 31.71, 23.33, 14.35, 1.40.

HRMS (ESI +ve) m/z: 2240.7954 ($[M]^{+\bullet}$, $C_{122}H_{139}O_8N_9TeI_2$ requires 2240.7909).

Tridentate Mixed XB/ChB [2]Rotaxane (8·XB$_2$ChB^{pCF3})

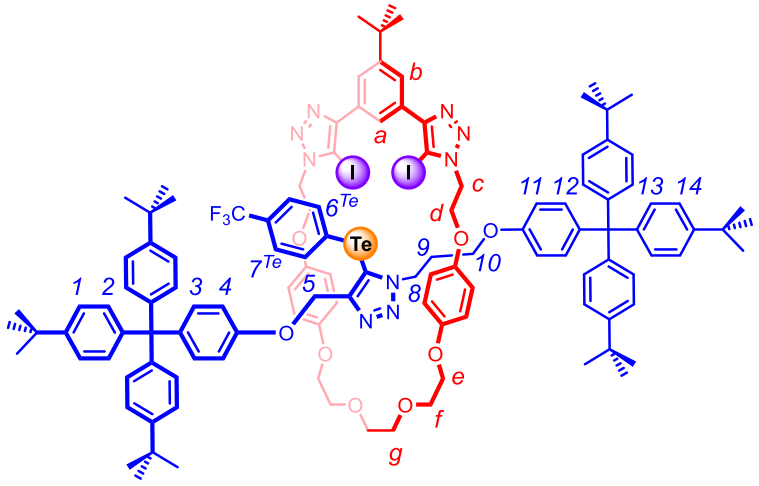

Following the general procedure for CuAAC-AMT synthesis of [2]rotaxanes between macrocycle **3·I** (10 mg, 0.011 mmol, 1 equiv), stopper azide **7** (32 mg, 0.055 mmol, 5 equiv) and stopper alkyne **5·Te^{pCF3}** (45 mg, 0.055 mmol, 5 equiv), the product was purified by preparative TLC (6% EtOAc in CH_2Cl_2) to give the target tridentate [2]rotaxane **8·XB$_2$ChB^{pCF3}** as white powder (3.3 mg, 13%).

^{1}H NMR (600 MHz, acetone-*d$_6$*) δ (ppm): 8.29 (t, *J* = 1.6 Hz, 1H; *a*), 8.00 (d, *J* = 1.6 Hz, 2H; *b*), 7.47 – 7.40 (m, 2H; 7^{Te}), 7.35 – 7.33 (m, 2H; 6^{Te}), 7.33 – 7.28 (m, 12H; *2, 13*), 7.15 – 7.08 (m, 12H; *1, 14*), 7.03 (d, *J* = 8.2 Hz, 2H; *3*), 6.97 (s, 2H), 6.47 (d, *J* = 9.1 Hz, 4H; *hydroquinone ArH*), 6.38 (d, *J* = 9.1 Hz, 4H; *hydroquinone ArH*), 4.99 – 4.92 (brs, 2H; *5*), 4.89 (t, *J* = 4.8 Hz, 4H; *c*), 4.38 – 4.31 (m, 4H; *d*), 4.24 – 4.14 (m, 2H; *8*), 3.84 – 3.77 (m, 4H; *e*), 3.58 (t, *J* = 5.2 Hz, 5H; *f*), 3.51 (s, 4H; *g*), 3.43 – 3.36 (m, 2H; *10*), 1.62 – 1.55 (m, 2H; *9*), 1.40 (s, 9H; *macrocycle ^{t}Bu H*), 1.30 (s, 54H; *axle ^{t}Bu H*).

^{13}C NMR (151 MHz, acetone-*d$_6$*) δ (ppm): 157.29, 154.11, 153.09, 152.70, 152.29, 150.36, 149.12, 149.05, 145.46, 145.34, 136.75, 132.68, 132.65, 131.85, 131.52, 131.47, 126.74 (q, $^4J_{C-F}$ = 4.0 Hz), 125.31, 125.11, 125.09, 116.19, 116.15, 114.39, 114.13, 80.44, 76.07, 71.32, 70.15, 68.51, 68.27, 65.02, 63.94, 63.05, 50.97, 48.87, 46.76, 35.72, 34.90, 34.89, 32.65, 31.75, 31.73, 31.70, 30.35, 23.34, 14.35, 12.29, 1.41 (signal of CF_3 missing due to poor signal-to-noise ratio).

^{19}F NMR (377 MHz, acetone-*d$_6$*) δ (ppm): −63.02.

HRMS (ESI +ve) m/z: 2308.7802 ([M+H]$^+$, $C_{123}H_{139}O_8N_9TeI_2F_3$ requires 2308.7827).

Tridentate All-HB [2]Rotaxane (8·HB$_3$)

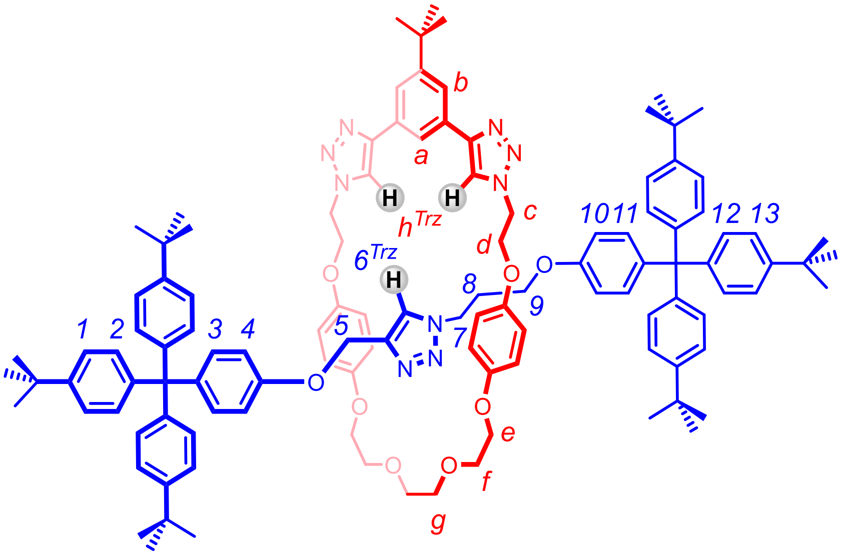

Following the general procedure for CuAAC-AMT synthesis of [2]rotaxanes between macrocycle **3·H** (10 mg, 0.015 mmol, 1 equiv), stopper azide **7** (45 mg, 0.076 mmol, 5 equiv) and stopper alkyne **4·H** (41 mg, 0.076 mmol, 5 equiv), the product was purified by preparative TLC (25% EtOAc in CH_2Cl_2) to give the target tridentate [2]rotaxane **8·HB$_3$** as white powder (5.0 mg, 18%).

^{1}H NMR (400 MHz, acetone-d_6) δ (ppm): 8.30 (s, 2H; H^{Trz}), 8.03 (d, J = 1.6 Hz, 2H; *b*), 7.95 (t, J = 1.6 Hz, 1H; *a*), 7.47 (s, 1H; 6^{Trz}), 7.36 – 7.23 (m, 12H; *2, 12*), 7.16 – 7.07 (m, 12H; *1, 13*), 7.06 – 7.00 (m, 4H; *3, 11*), 6.80 (d, J = 8.9 Hz, 2H; *4*), 6.59 – 6.46 (m, 10H; *10, hydroquinone ArH*), 4.85 (s, 2H; *5*), 4.71 (t, J = 4.8 Hz, 4H; *c*), 4.30 (t, J = 4.8 Hz, 4H; *d*), 3.93 (t, J = 6.5 Hz, 2H; *7*), 3.90 – 3.85 (m, 4H; *e*), 3.62 (t, J = 4.6 Hz, 4H; *f*), 3.54 (s, 4H; *g*), 3.48 (t, J = 6.5 Hz, 2H; *9*), 1.36 (s, 9H; *macrocycle ^{t}Bu H*), 1.30 (s, 54H; *axle ^{t}Bu H*).

^{13}C NMR (151 MHz, acetone-d_6) δ (ppm): 157.41, 157.27, 154.27, 153.02, 152.97, 149.12, 149.10, 148.11, 145.36, 145.28, 144.01, 140.53, 140.30, 132.78, 132.69, 132.56, 131.43, 125.11, 125.09, 124.87, 122.84, 122.24, 121.36, 116.70, 116.22, 114.33, 114.09, 71.44, 70.27, 68.77, 68.07, 64.95, 63.93, 61.93, 50.42, 47.39, 35.53, 34.89, 31.75, 31.70, 31.69, 23.34, 14.36, 1.42.

HRMS (ESI +ve) m/z: 1786.0609 ([M+H]$^+$, $C_{116}H_{138}O_8N_9$ requires 1786.0696).

Tetradentate All-ChB [2]Rotaxane (9·ChB$_4$)

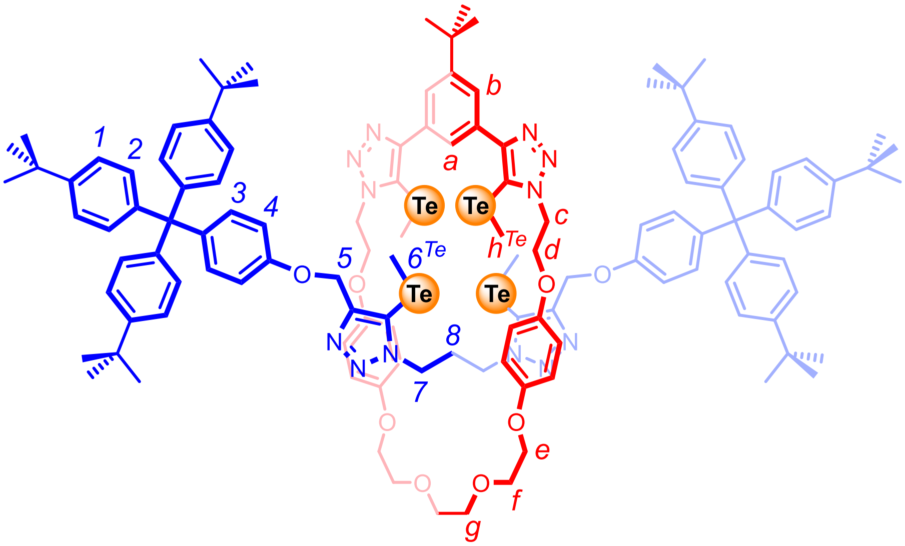

Following the general procedure for CuAAC-AMT synthesis of [2]rotaxanes between macrocycle **3·Te^{Me}** (10 mg, 0.011 mmol, 1 equiv), stopper azide **6·Te^{Me}** (43 mg, 0.053 mmol, 5 equiv) and stopper alkyne **5·Te^{Me}** (36 mg, 0.053 mmol, 5 equiv), the product was purified by preparative TLC (acetone/EtOAc/CH_2Cl_2 2:20:78) to give the target tetradentate [2]rotaxane **9·ChB$_4$** as white powder (4.7 mg, 18%).

^{1}H NMR (600 MHz, acetone-*d*$_6$) δ (ppm): 8.62 (t, *J* = 1.6 Hz, 1H; *a*), 8.06 (d, *J* = 1.6 Hz, 2H; *b*), 7.30 (d, *J* = 8.6 Hz, 12H; *2*), 7.14 (d, *J* = 8.6 Hz, 12H; *1*), 7.10 (d, *J* = 8.9 Hz, 4H; *3*), 6.87 (d, *J* = 8.9 Hz, 4H; *4*), 6.45 (d, *J* = 9.1 Hz, 4H; *hydroquinone ArH*), 6.29 (d, *J* = 9.1 Hz, 4H; *hydroquinone ArH*), 5.01 (t, *J* = 4.6 Hz, 4H; *c*), 4.97 (s, 4H; *5*), 4.31 – 4.25 (m, 8H; *d, 7*), 3.86 (t, *J* = 4.8 Hz, 4H; *e*), 3.64 (t, *J* = 4.8 Hz, 4H; *f*), 3.59 (s, 4H; *g*), 2.01 (s, 6H; *h*^{Te}), 1.65 (s, 6H; *6*^{Te}), 1.43 (s, 9H; *macrocycle tert*-butyl *H*), 1.30 (s, 54H; *axle tert*-butyl *H H*).

^{13}C NMR (151 MHz, acetone-*d*$_6$) δ (ppm): 157.40, 153.95, 153.35, 152.83, 151.96, 151.70, 149.10, 145.38, 140.35, 132.91, 132.75, 132.71, 131.47, 125.40, 125.09, 115.98, 115.73, 114.52, 105.75, 103.51, 71.28, 70.21, 68.64, 68.32, 63.96, 63.32, 51.34, 48.82, 34.89, 31.78, 31.71, 23.34, 14.36, -12.61, -12.69.

HRMS (ESI +ve) m/z: 2435.7847 ([M]$^{+•}$, $C_{123}H_{148}N_{12}O_8Te_4$ requires 2435.7773).

Tetradentate Mixed XB/ChB [2]Rotaxane (9·XB$_2$ChB$_2$)

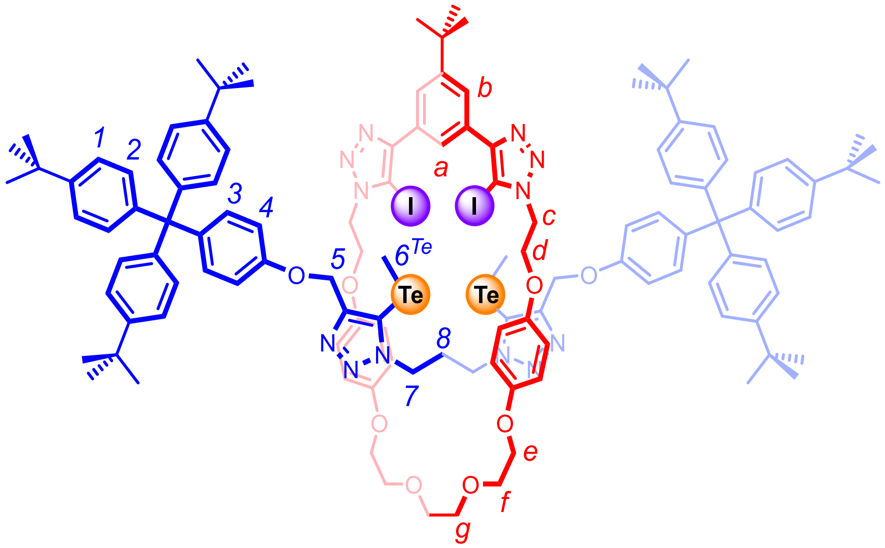

Following the general procedure for CuAAC-AMT synthesis of [2]rotaxanes between macrocycle **3·I** (10 mg, 0.011 mmol, 1 equiv), stopper azide **6·Te^{Me}** (45 mg, 0.055 mmol, 5 equiv) and stopper alkyne **5·Te^{Me}** (38 mg, 0.055 mmol, 5 equiv), the product was purified by preparative TLC (30% EtOAc in CH_2Cl_2) to give the target tetradentate [2]rotaxane **9·XB$_2$ChB$_2$** as white powder (2.6 mg, 10%).

^{1}H NMR (400 MHz, acetone-*d$_6$*) δ (ppm): 8.34 (t, *J* = 1.6 Hz, 1H; *a*), 8.01 (d, *J* = 1.6 Hz, 2H; *b*), 7.30 (d, *J* = 8.6 Hz, 12H; *2*), 7.12 (d, *J* = 8.6 Hz, 12H; *1*), 7.08 (d, *J* = 8.9 Hz, 4H; *3*), 6.85 (d, *J* = 8.9 Hz, 4H; *4*), 6.45 (d, *J* = 9.1 Hz, 4H; *hydroquinone ArH*), 6.32 (d, *J* = 9.1 Hz, 4H; *hydroquinone ArH*), 5.00 (s, 4H; *5*), 4.88 (t, *J* = 4.8 Hz, 4H; *c*), 4.31 (t, *J* = 4.8 Hz, 4H; *d*), 4.25 (t, *J* = 7.4 Hz, 4H; *7*), 3.87 (t, *J* = 4.9 Hz, 4H; *e*), 3.65 (t, *J* = 4.9 Hz, 4H; *f*), 3.61 (s, 4H; *g*), 1.69 (s, 6H; 6^{Te}), 1.43 (s, 9H; *macrocycle tert*-butyl *H*), 1.30 (s, 54H; axle *tert*-butyl *H*). (Proton signal *8* missing due to signal overlapping).

^{13}C NMR (151 MHz, acetone-*d$_6$*) δ (ppm): 157.38, 153.96, 152.90, 152.38, 151.98, 150.45, 149.12, 145.34, 140.42, 132.77, 131.49, 131.47, 125.10, 115.97, 115.91, 114.53, 105.66, 80.68, 71.33, 70.27, 68.33, 68.03, 63.95, 63.33, 50.96, 48.76, 35.77, 34.89, 32.65, 31.71, 23.34, 14.35, 1.41, -12.67.

HRMS (ESI +ve) m/z: 2403.7405 ($[M]^{+\bullet}$, $C_{121}H_{142}I_2N_{12}O_8Te_2$ requires 2403.7298).

Tetradentate Mixed XB/ChB [2]Rotaxane (9·XB$_3$ChB)

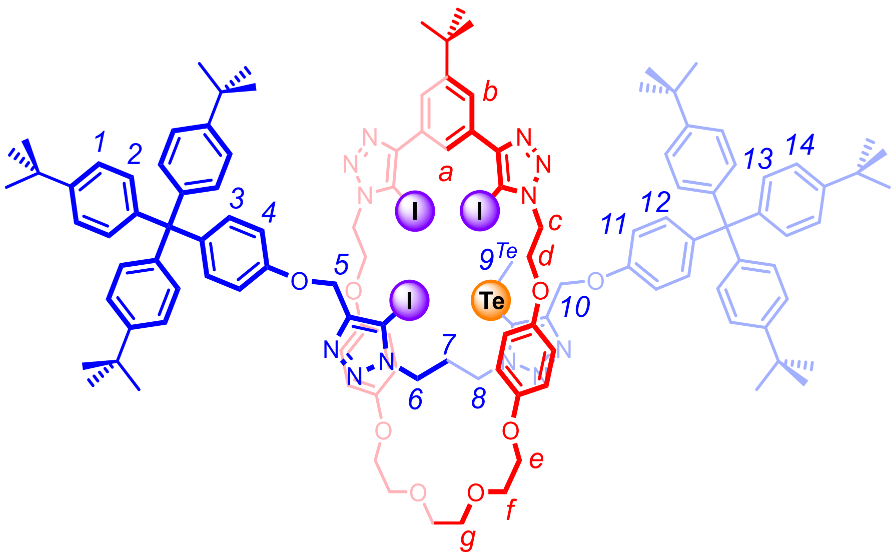

Following the general procedure for CuAAC-AMT synthesis of [2]rotaxanes between macrocycle **3·I** (10 mg, 0.011 mmol, 1 equiv), stopper azide **6·I** (44 mg, 0.055 mmol, 5 equiv) and stopper alkyne **5·Te^{Me}** (38 mg, 0.055 mmol, 5 equiv), the product was purified by preparative TLC (acetone/EtOAc/CH_2Cl_2 2:20:78) to give the target tetradentate [2]rotaxane **9·XB$_3$ChB** as white powder (4.5 mg, 17%).

^{1}H NMR (600 MHz, acetone-d_6) δ (ppm): 8.37 (t, J = 1.6 Hz, 1H; *a*), 8.01 (d, J = 1.6 Hz, 2H; *b*), 7.30 (d, J = 8.6 Hz, 12H; *2, 13*), 7.12 (d, J = 8.6 Hz, 12H; *1, 14*), 7.10 – 7.06 (m, 4H; *3, 12*), 6.90 – 6.83 (m, 4H; *4, 11*), 6.46 (d, J = 9.1 Hz, 4H; *hydroquinone ArH*), 6.37 (d, J = 9.1 Hz, 4H; *hydroquinone ArH*), 5.00 (s, 2H; *5*), 4.88 (t, J = 4.7 Hz, 4H; *c*), 4.86 (s, 2H; *10*), 4.32 (t, J = 4.7 Hz, 4H; *d*), 4.22 (t, J = 7.3 Hz, 2H; *6*), 4.03 (t, J = 7.3 Hz, 2H; *8*), 3.88 (t, J = 4.7 Hz, 4H; *e*), 3.65 (t, J = 4.7 Hz, 4H; *f*), 3.59 (s, 4H; *g*), 1.65 (s, 3H; *9^{Te}*), 1.43 (s, 9H; *macrocycle ^{t}Bu H*), 1.30 (s, 54H; *axle ^{t}Bu H*).

^{13}C NMR (151 MHz, acetone-d_6) δ (ppm): 157.40, 157.33, 153.96, 152.95, 152.36, 151.96, 150.51, 149.13, 147.97, 145.33, 145.31, 140.59, 140.43, 132.79, 131.96, 131.49, 125.51, 125.42, 125.10, 116.00, 115.97, 114.56, 114.53, 105.69, 82.88, 80.58, 71.36, 70.28, 68.38, 68.03, 63.95, 63.34, 62.14, 50.98, 48.55, 48.35, 35.75, 34.89, 31.78, 31.71, 30.73, 30.47, 23.34, 14.36, 1.41, -12.75 (peaks missing due to signal overlapping).

HRMS (ESI +ve) m/z: 2388.7104 ($[M+H]^+$, $C_{120}H_{140}I_3N_{12}O_8Te$ requires 2388.7141).

Tetradentate Mixed XB/ChB [2]Rotaxane (9·ChB$_3$XB)

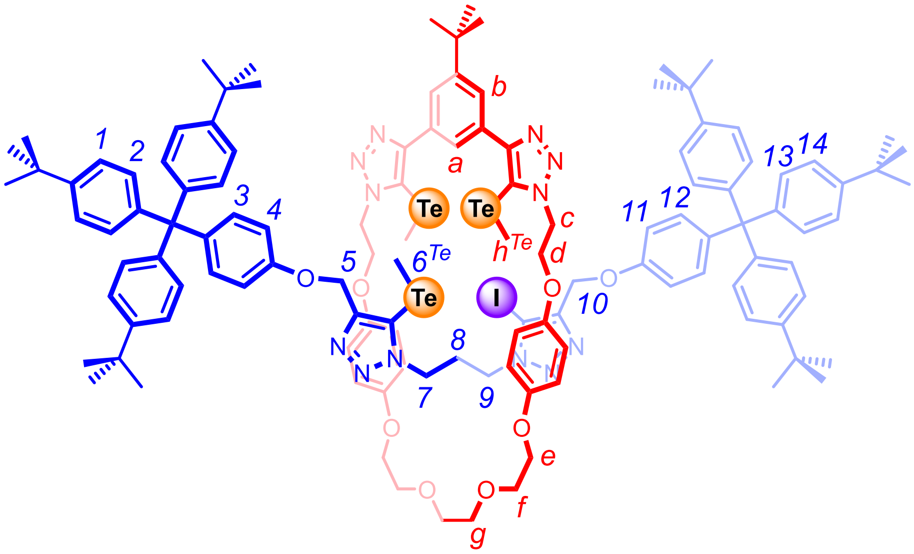

Following the general procedure for CuAAC-AMT synthesis of [2]rotaxanes between macrocycle **3·Te^{Me}** (10 mg, 0.011 mmol, 1 equiv), stopper azide **6·I** (42 mg, 0.053 mmol, 5 equiv) and stopper alkyne **5·Te^{Me}** (36 mg, 0.053 mmol, 5 equiv), the product was purified by preparative TLC (acetone/EtOAc/CH_2Cl_2 2:20:78) to give the target tetradentate [2]rotaxane **9·ChB$_3$XB** as white powder (5.9 mg, 23%).

^{1}H NMR (600 MHz, acetone-d_6) δ (ppm): 8.62 (d, J = 1.6 Hz, 1H; *a*), 8.07 (d, J = 1.6 Hz, 2H; *b*), 7.33 – 7.27 (m, 12H; *2, 13*), 7.16 – 7.12 (m, 12H; *1, 14*), 7.12 – 7.09 (m, 4H; *3, 12*), 6.92 – 6.87 (m, 4H; *4, 11*), 6.44 (d, J = 8.8 Hz, 4H; *hydroquinone ArH*), 6.33 (d, J = 8.8 Hz, 4H; *hydroquinone ArH*), 5.00 (t, J = 4.8 Hz, 4H; *c*), 4.98 (s, 2H; *10*), 4.87 (s, 2H; *5*), 4.28 (t, J = 4.8 Hz, 4H; *d*), 4.23 (t, J = 7.4 Hz, 2H; *9*), 4.03 (t, J = 7.4 Hz, 2H; *7*), 3.88 (d, J = 4.1 Hz, 4H; *e*), 3.65 (t, J = 4.1 Hz, 4H; *f*), 3.60 (s, 4H; *g*), 2.00 (s, 6H; h^{Te}), 1.61 (s, 3H; 6^{Te}), 1.43 (s, 9H; *macrocycle* ^{t}*Bu H*), 1.30 (s, 54H; *axle* ^{t}*Bu H*).

^{13}C NMR (151 MHz, acetone-d_6) δ (ppm): 157.42, 157.36, 153.95, 153.39, 152.87, 151.95, 151.73, 149.13, 149.11, 147.94, 145.36, 145.32, 140.57, 140.39, 132.94, 132.75, 132.73, 131.47, 126.05, 125.46, 125.09, 115.99, 115.77, 114.54, 114.52, 105.73, 103.47, 82.93, 71.33, 70.25, 68.62, 68.37, 63.96, 63.34, 62.16, 51.35, 48.61, 48.39, 35.70, 34.88, 31.79, 31.70, 30.35, 23.34, 14.36, -12.60, -12.74 (peaks missing due to signal overlapping).

HRMS (ESI +ve) m/z: 2417.7587 ($[M]^{+\bullet}$, $C_{122}H_{145}IN_{12}O_8Te_3$ requires 2417.7521).

S2 Spectral Characterisation of Novel Compounds

Spectral Characterisation of Macrocycle Precursors

XB Macrocycle (3·I)

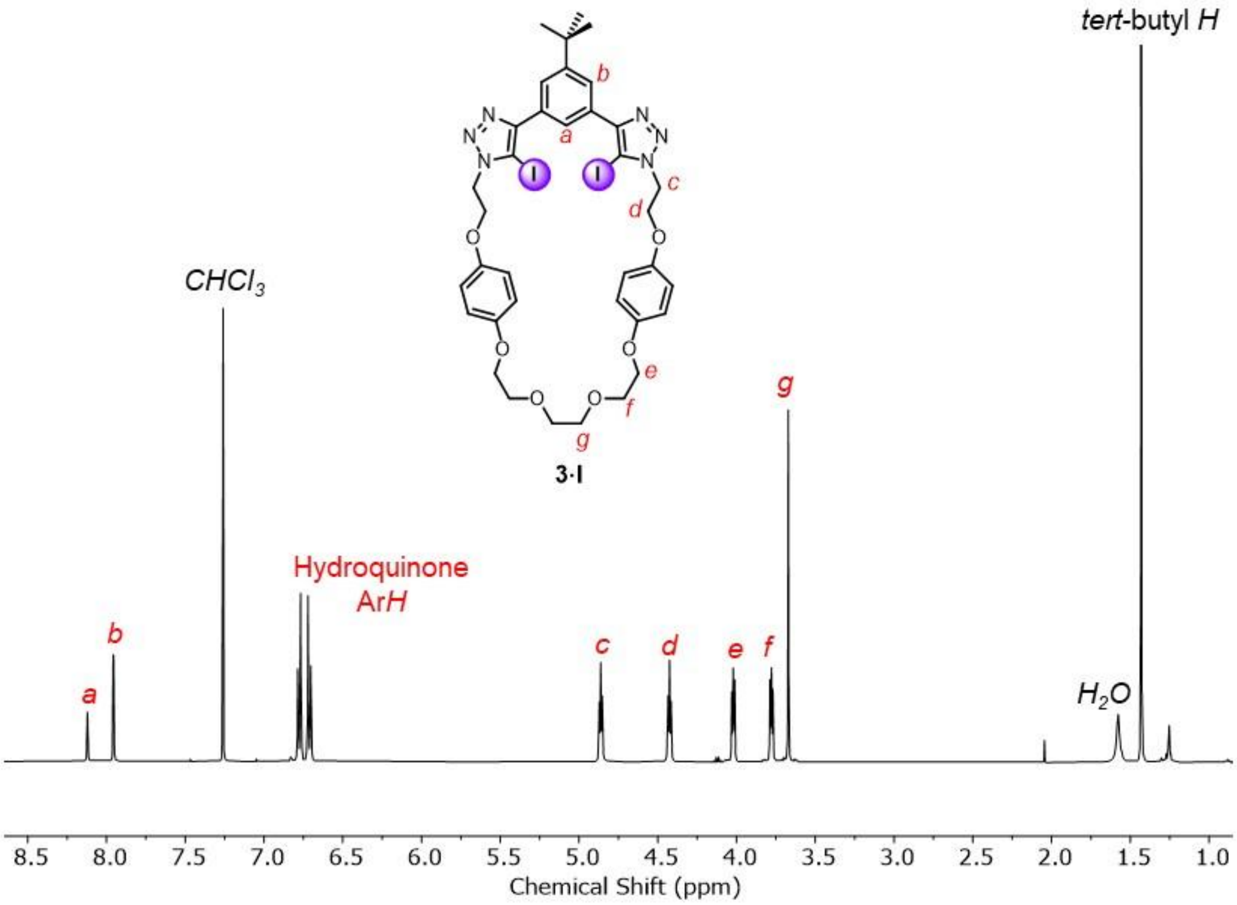

Fig. 5 | ^{1}H NMR spectrum of **3·I** (400 MHz, 298 K, $CDCl_3$).

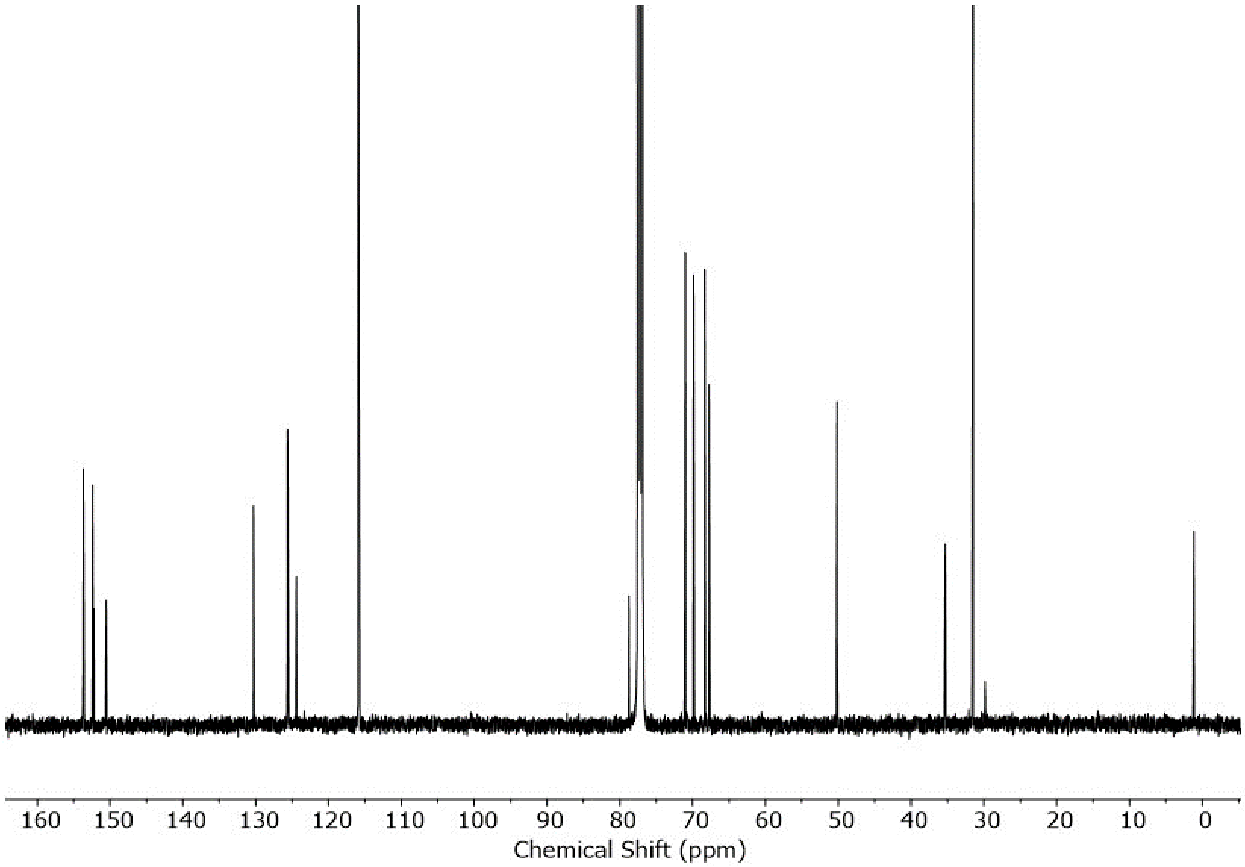

Fig. 6| ^{13}C NMR spectrum of **3·I** (126 MHz, 298 K, $CDCl_3$).

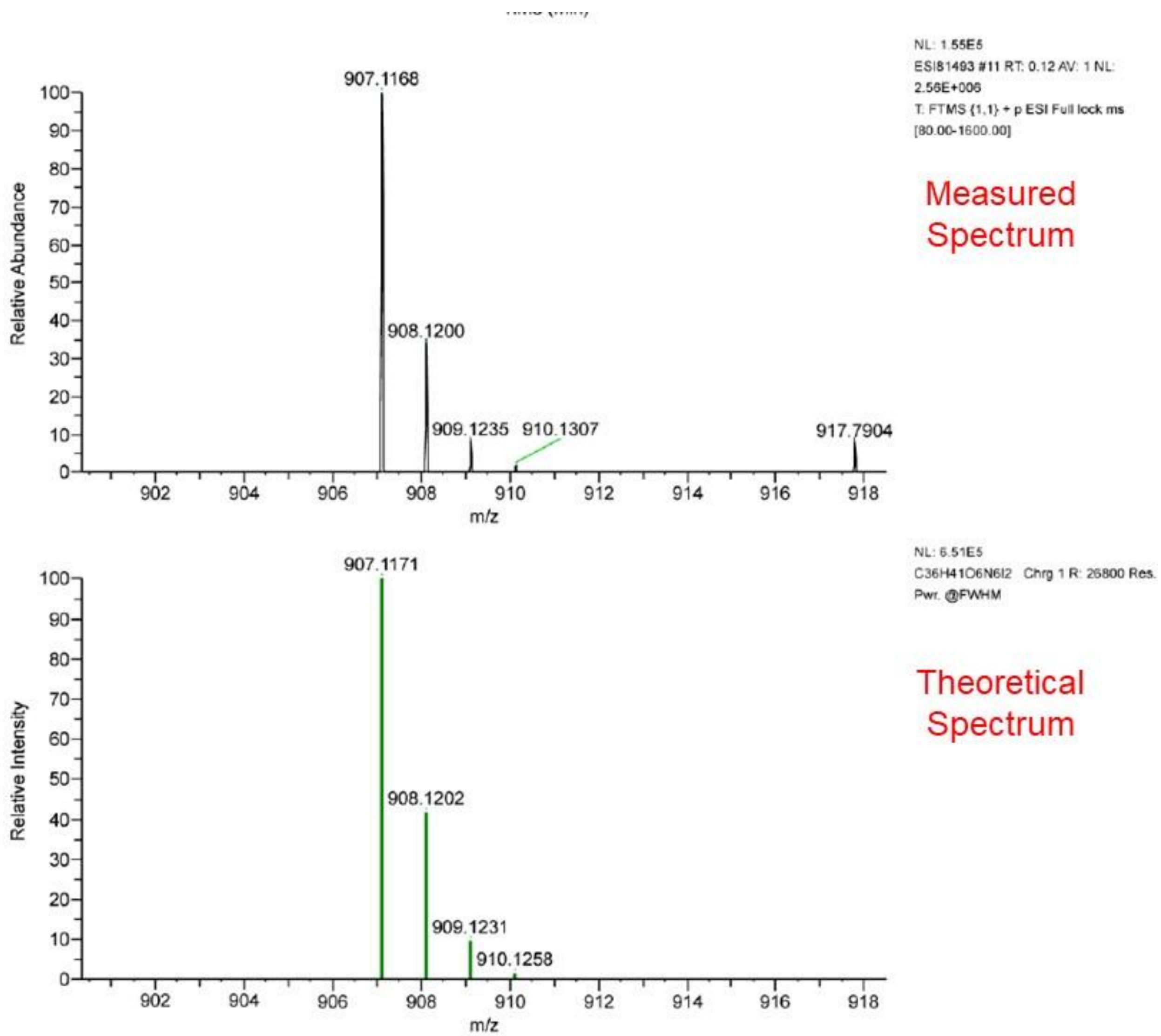

Fig. 7 | Measured (top) and theoretical (bottom) high-resolution ESI mass spectrum of **3·I**.

ChB Macrocycle (3·Te^{Me})

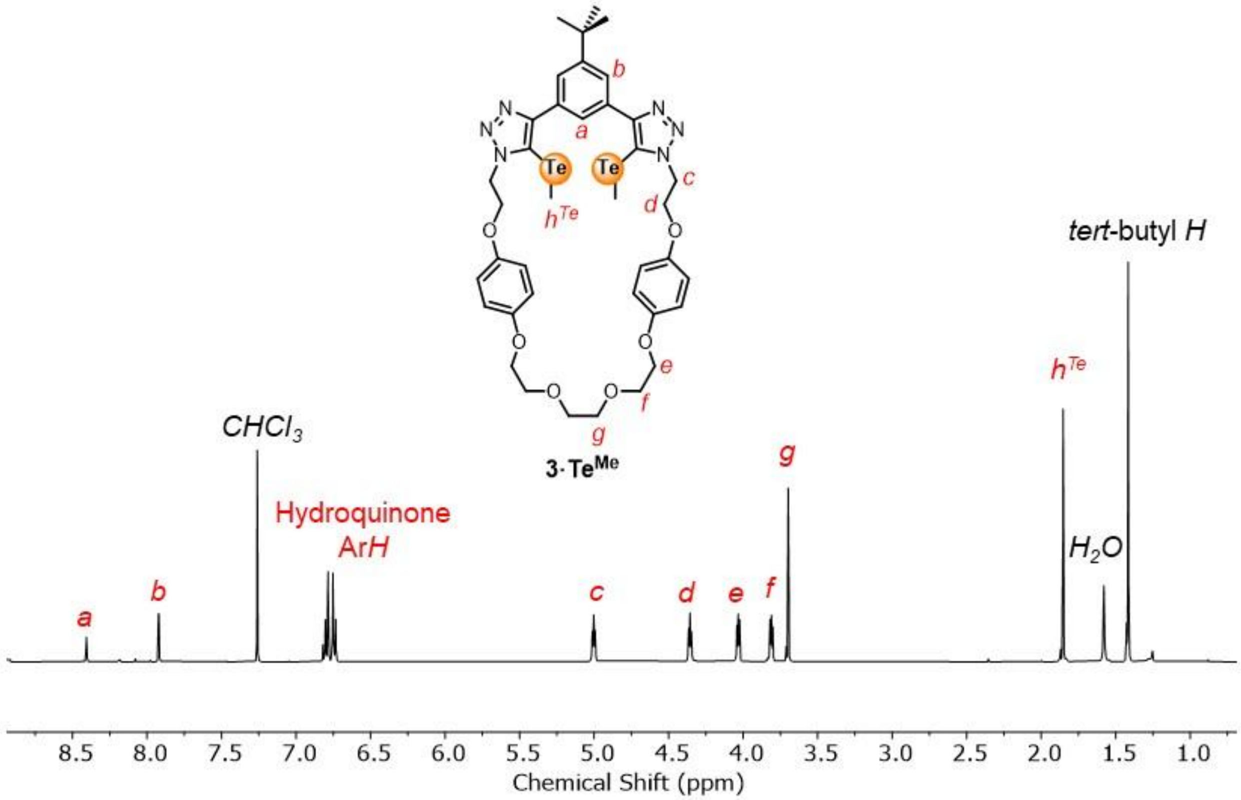

Fig. 8 | ^{1}H NMR spectrum of **3·Te^{Me}** (400 MHz, 298 K, CDCl$_3$).

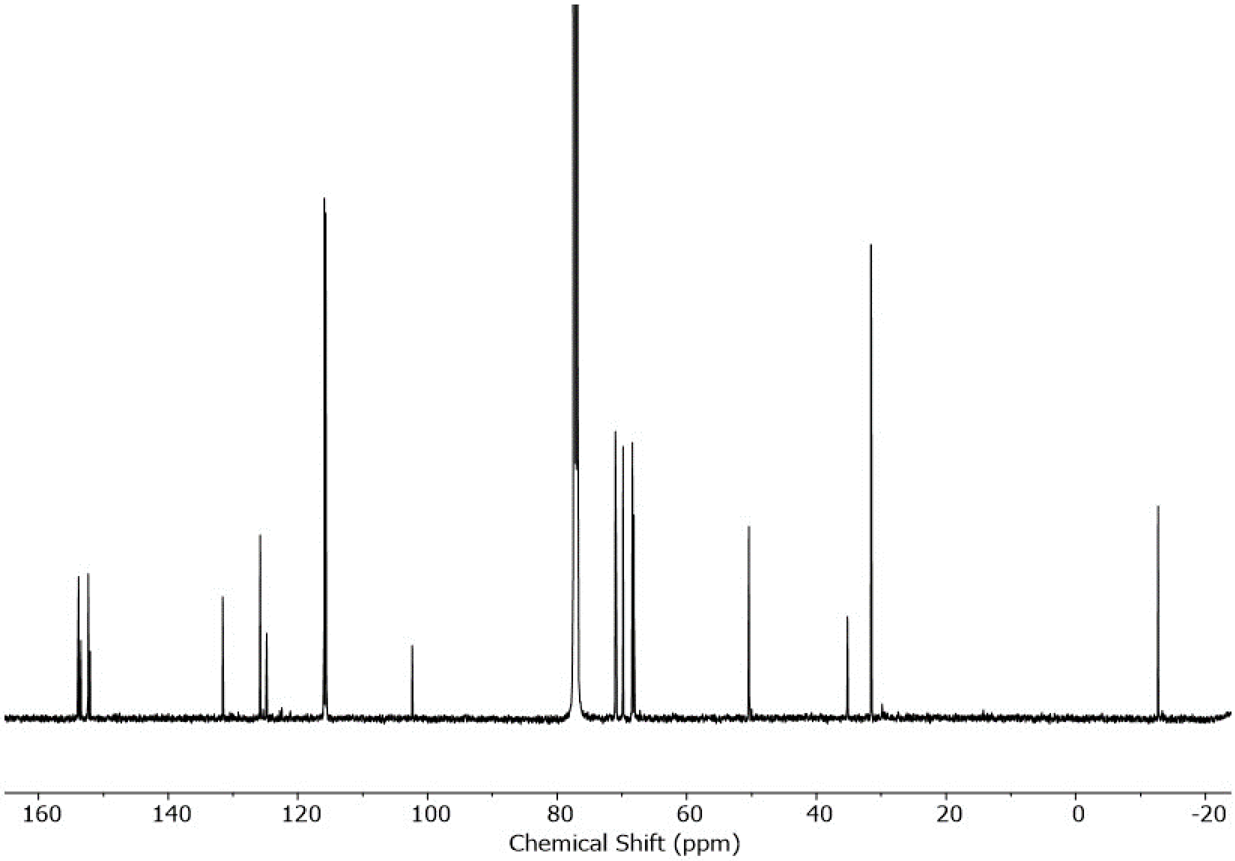

Fig. 9 | ^{13}C NMR spectrum of **3·Te^{Me}** (126 MHz, 298 K, $CDCl_3$).

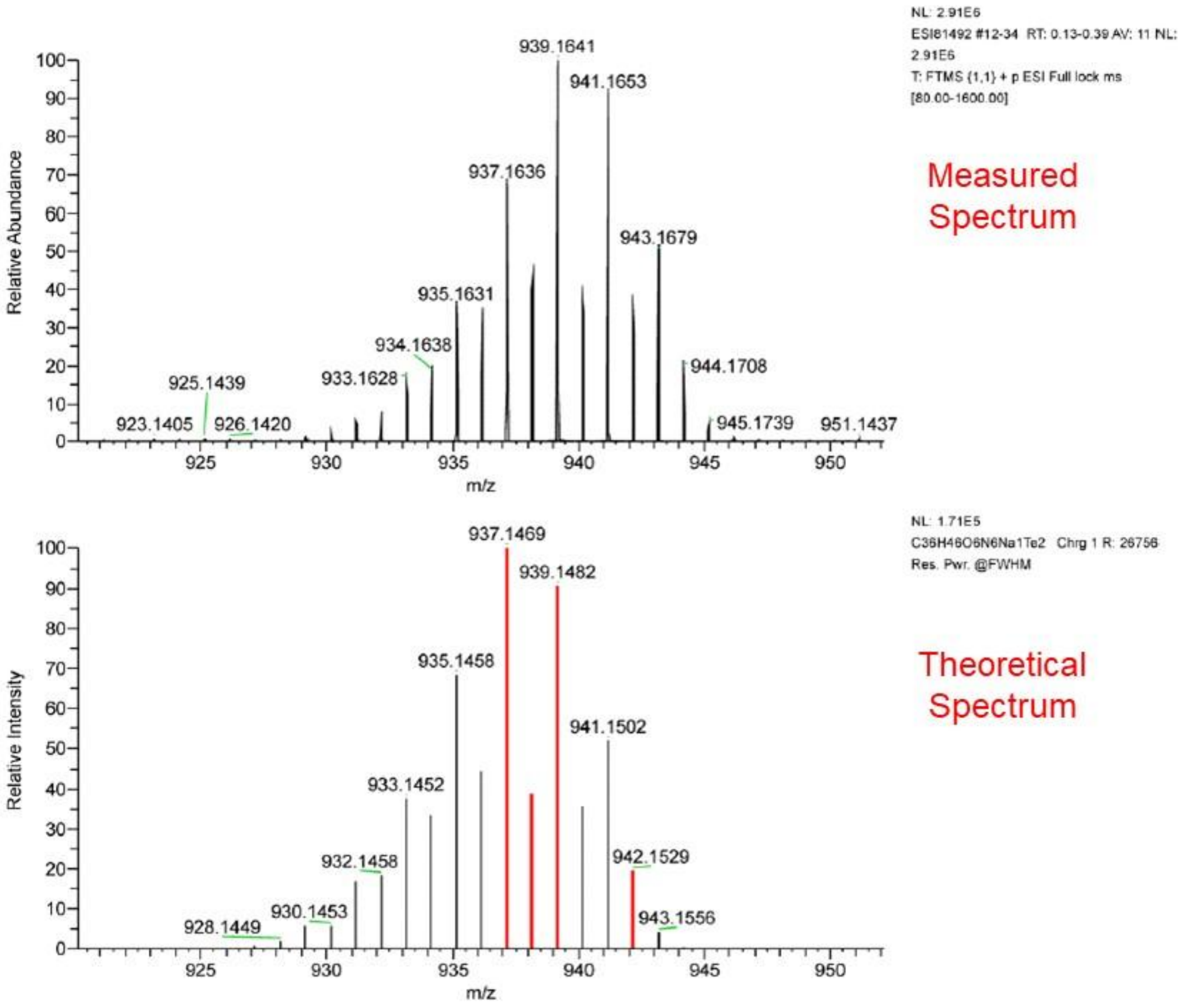

Fig. 10 | Measured (top) and theoretical (bottom) high-resolution ESI mass spectrum of **3·Te^{Me}**.

HB Macrocycle (3·H)

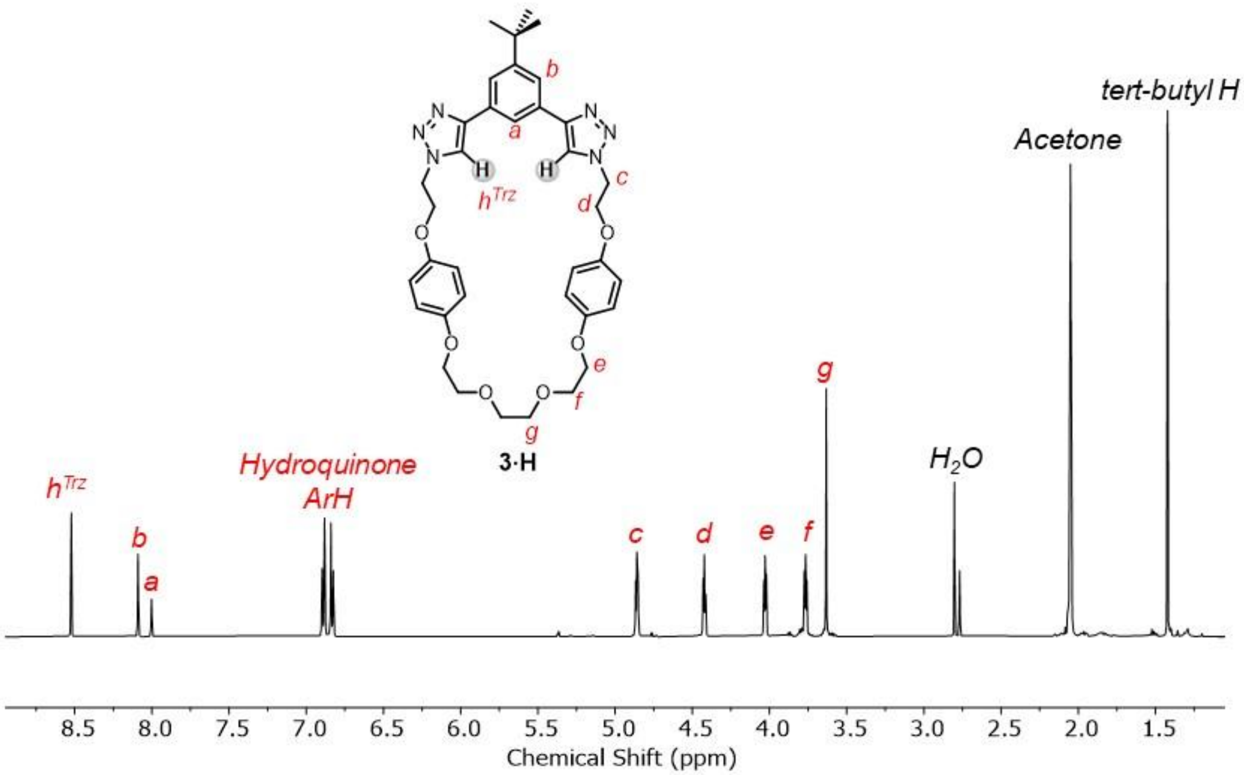

Fig. 11| ^{1}H NMR spectrum of **3·H** (400 MHz, 298 K, acetone-d_6).

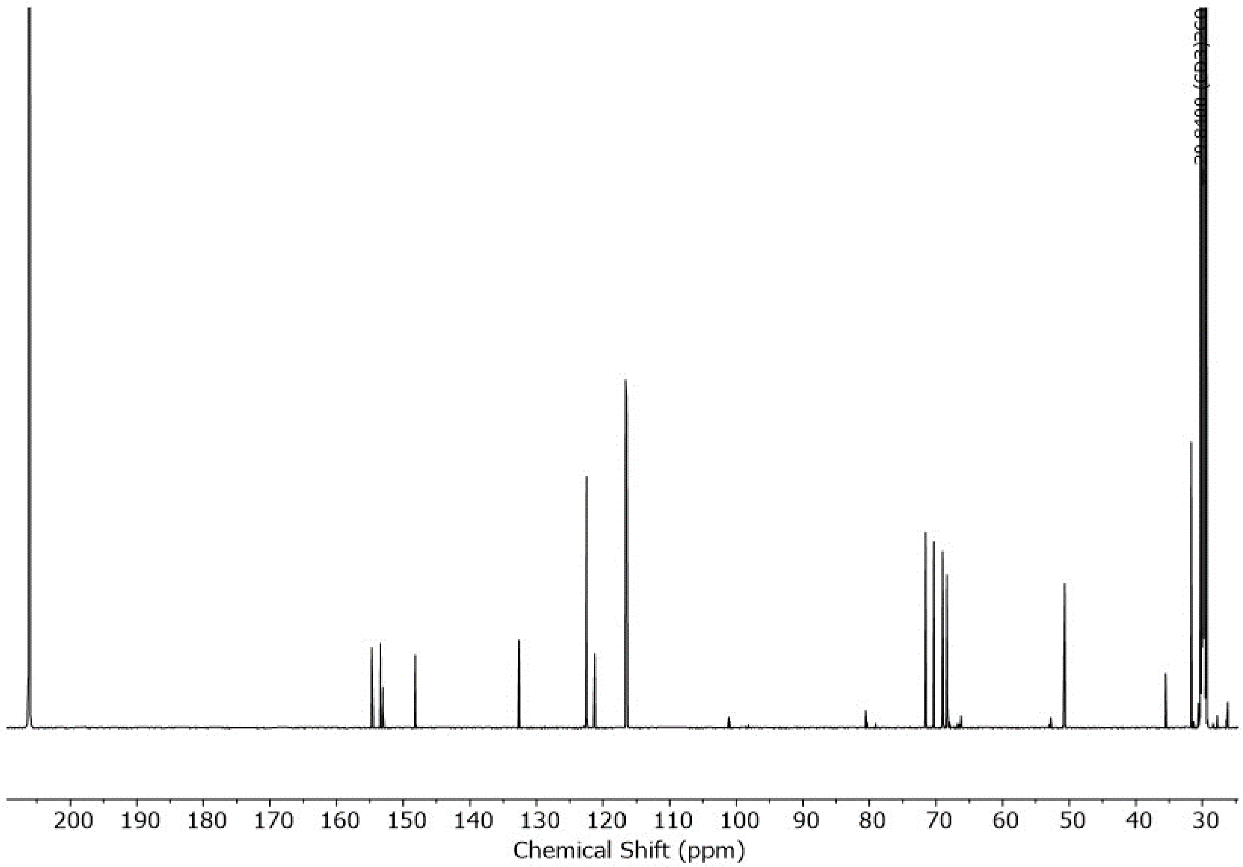

Fig. 12 | ^{13}C NMR spectrum of **3·H** (126 MHz, 298 K, acetone-d_6).

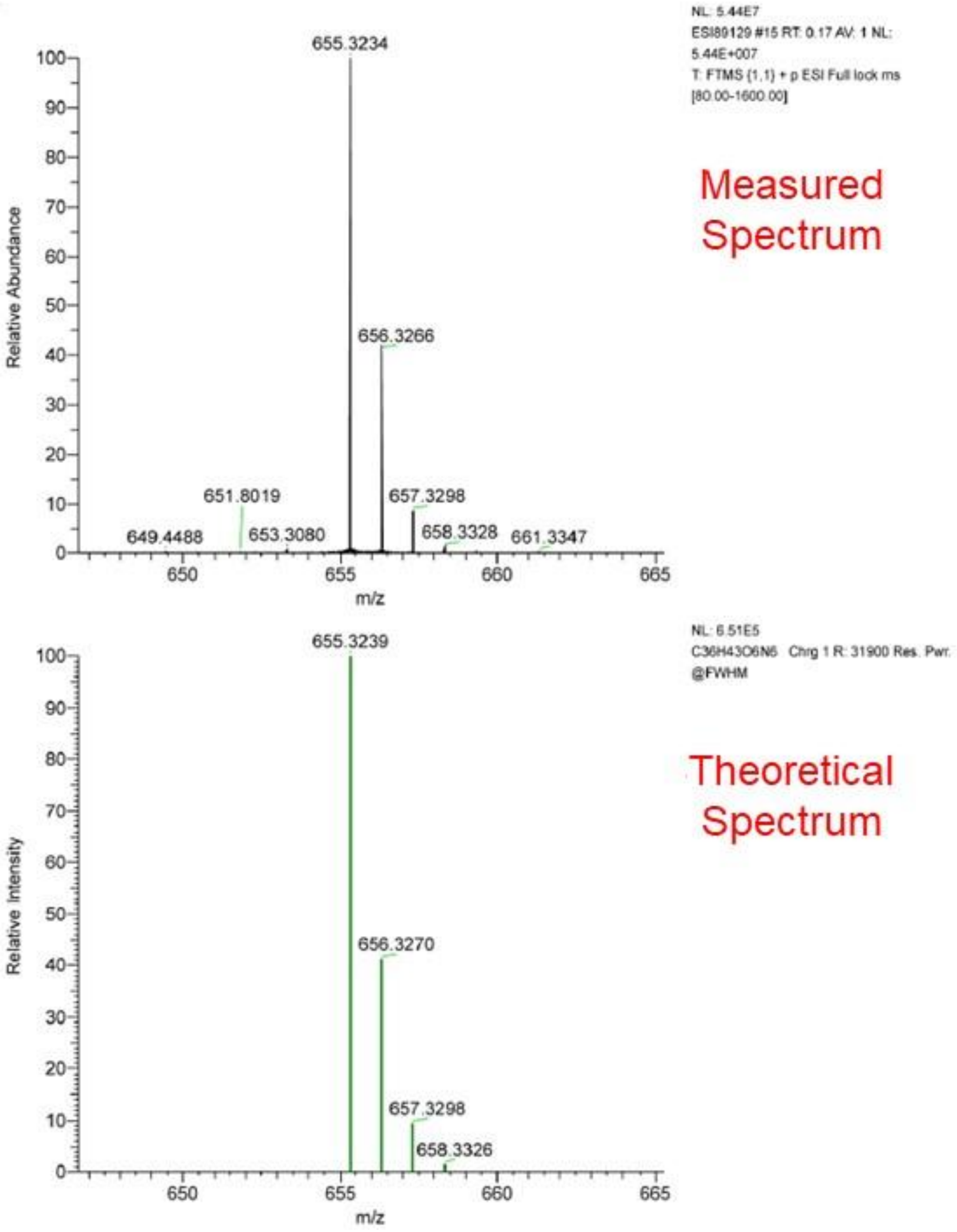

Fig. 13 | Measured (top) and theoretical (bottom) high-resolution ESI mass spectrum of **3·H**.

<u>*Spectral Characterisation of Alkynyl Axle Precursors*</u>

Stopper telluromethyl-alkyne (5·Te^{Me})

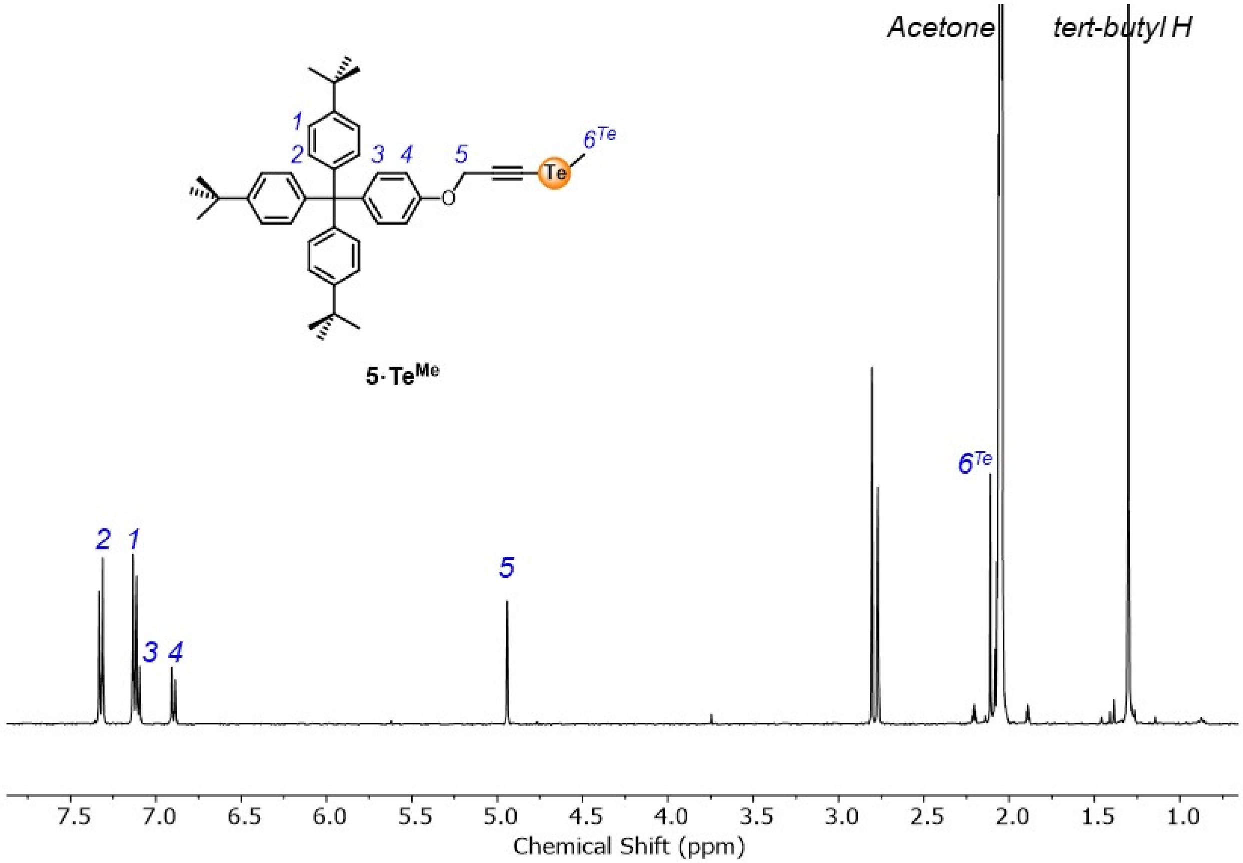

Fig. 14 | ^{1}H NMR spectrum of **5·Te^{Me}** (400 MHz, 298 K, acetone-*d*$_6$).

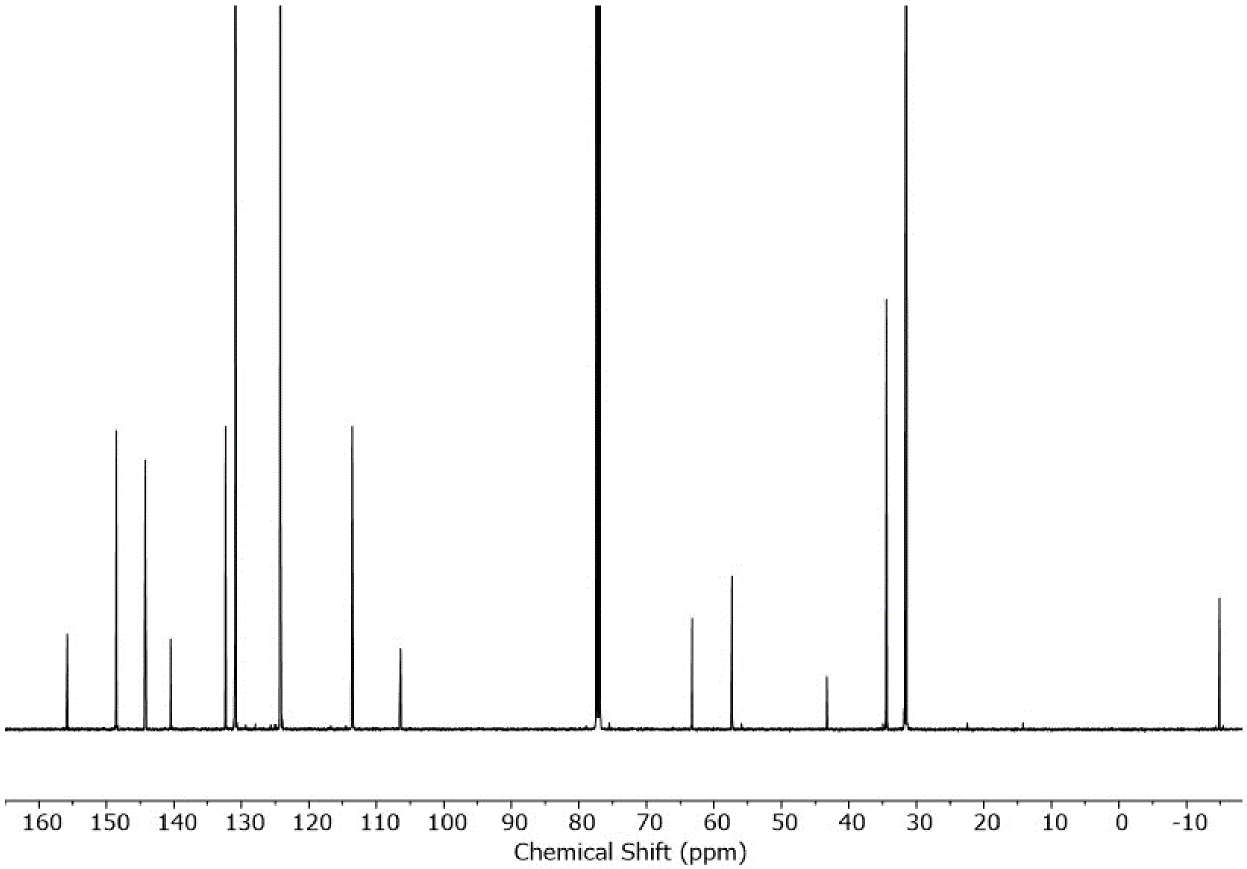

Fig. 15 | ^{13}C NMR spectrum of **5·Te^{Me}** (126 MHz, 298 K, $CDCl_3$).

Stopper Phenyl Telluroalkyne (5·Te^{Ph})

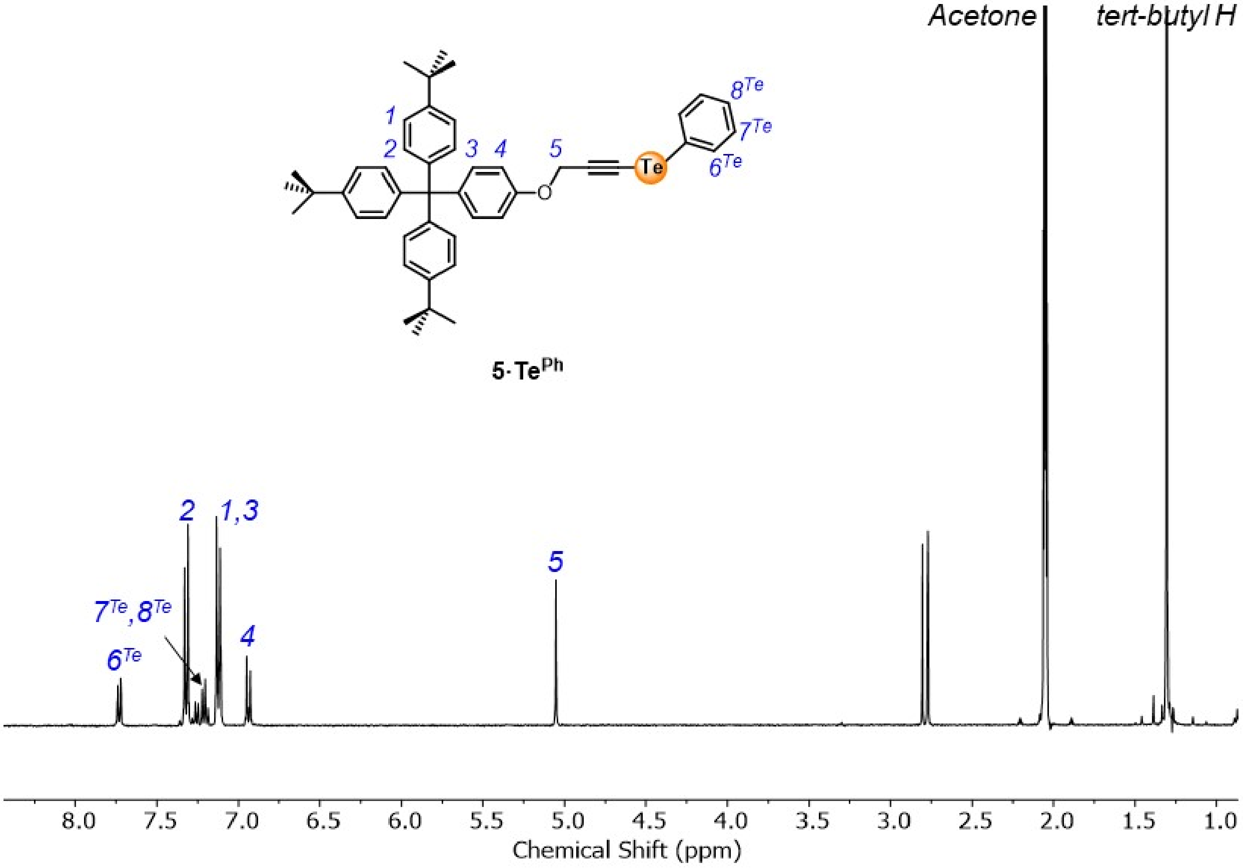

Fig. 16 | ^{1}H NMR spectrum of **5·Te^{Ph}** (400 MHz, 298 K, acetone-d_6).

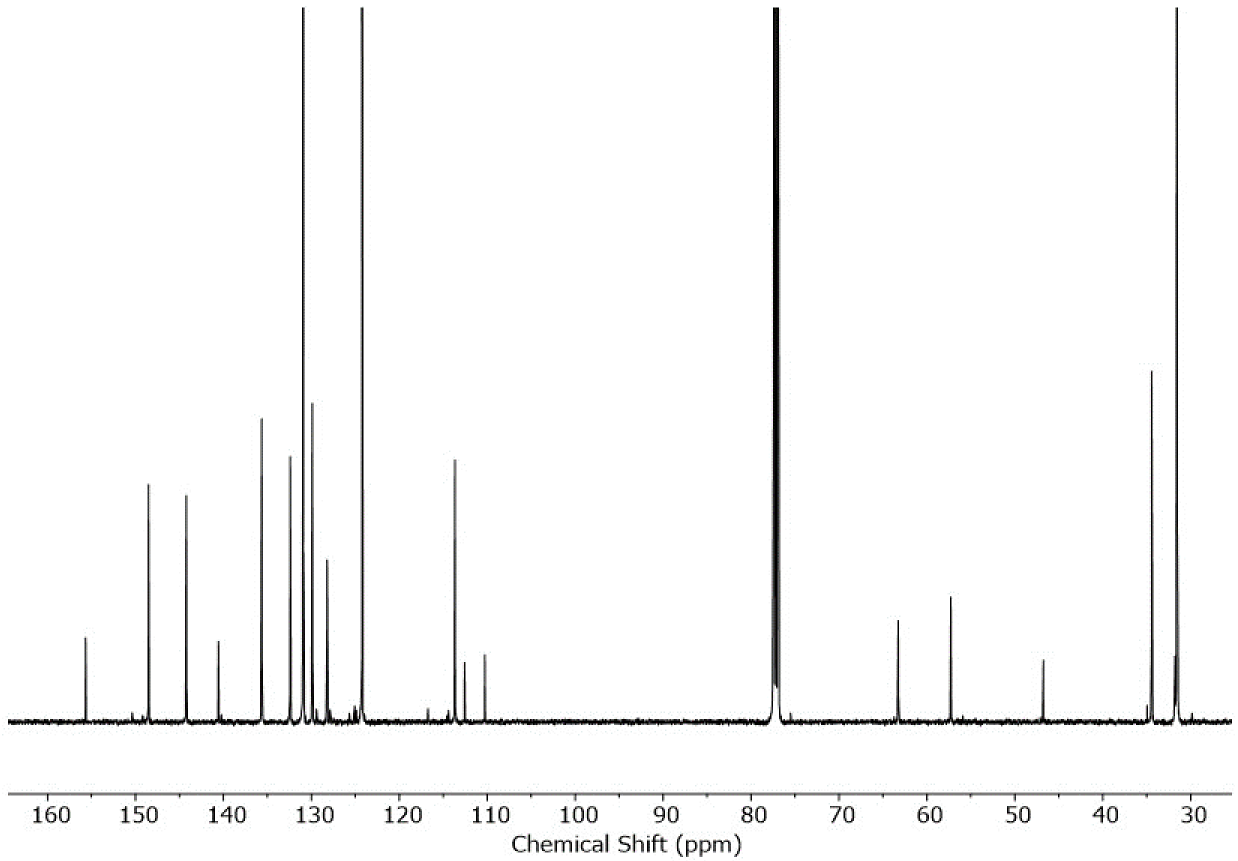

Fig. 17 | ^{13}C NMR spectrum of **5·Te^{Ph}** (126 MHz, 298 K, $CDCl_3$).

Stopper *para*-Trifluoromethyl Telluroalkyne (5·Te^{pCF3})

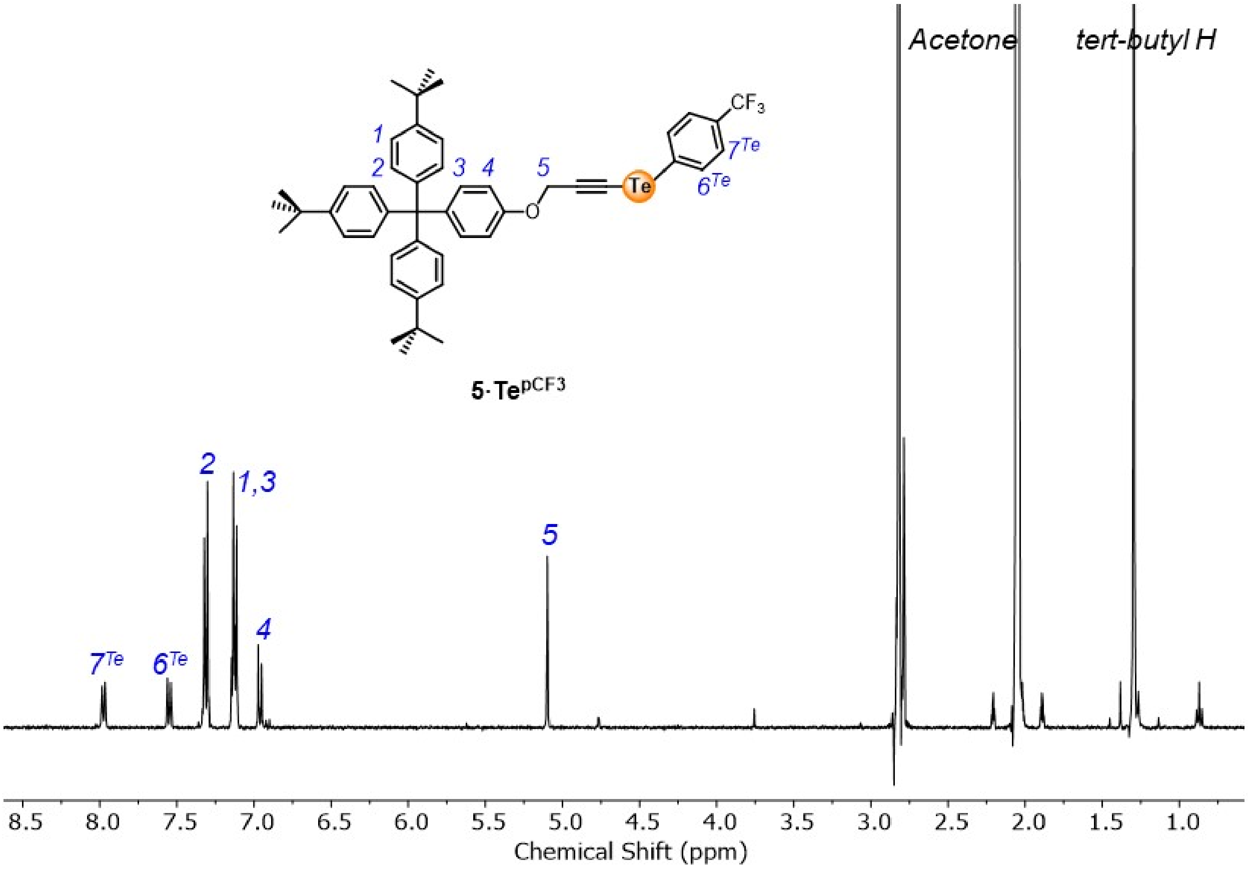

Fig. 18 | ^{1}H NMR spectrum of **5·Te^{pCF3}** (400 MHz, 298 K, acetone-d_6).

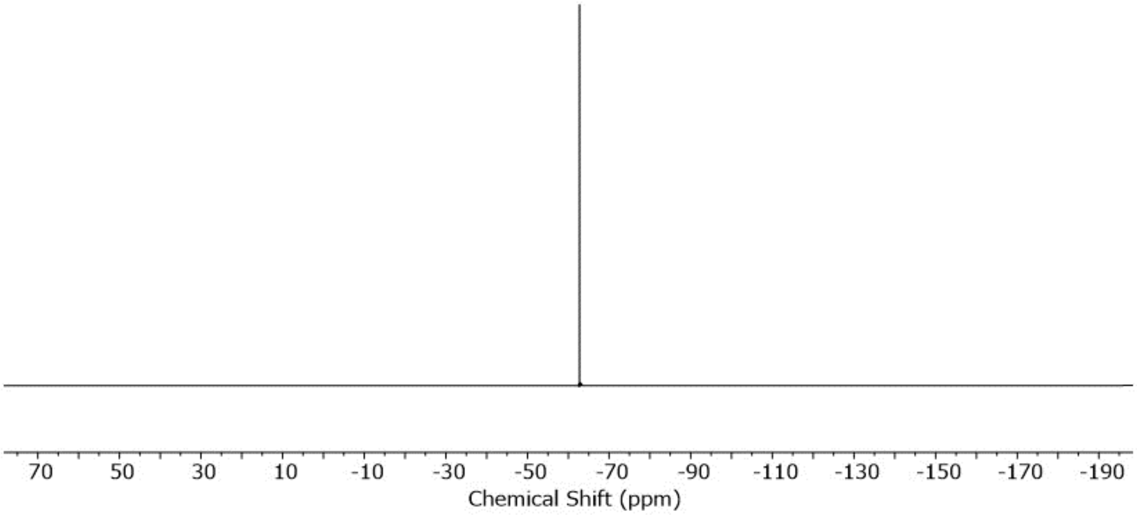

Fig.19 | ^{19}F NMR spectrum of **5·Te^{pCF3}** (377 MHz, 298 K, $CDCl_3$).

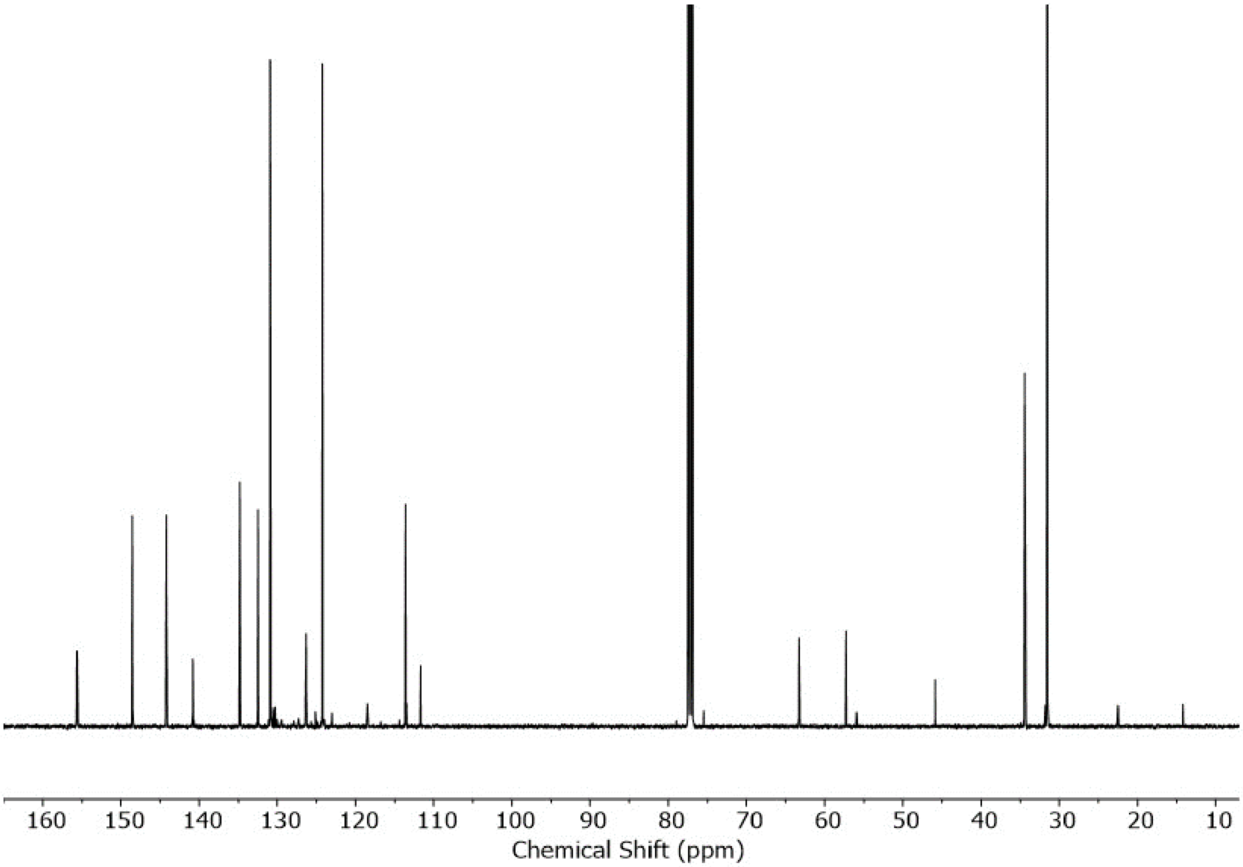

Fig. 20 | ^{13}C NMR spectrum of **5·Te^{pCF3}** (126 MHz, 298 K, $CDCl_3$).

Stopper Telluromethyl-triazole-functionalised Azide (6·Te^{Me})

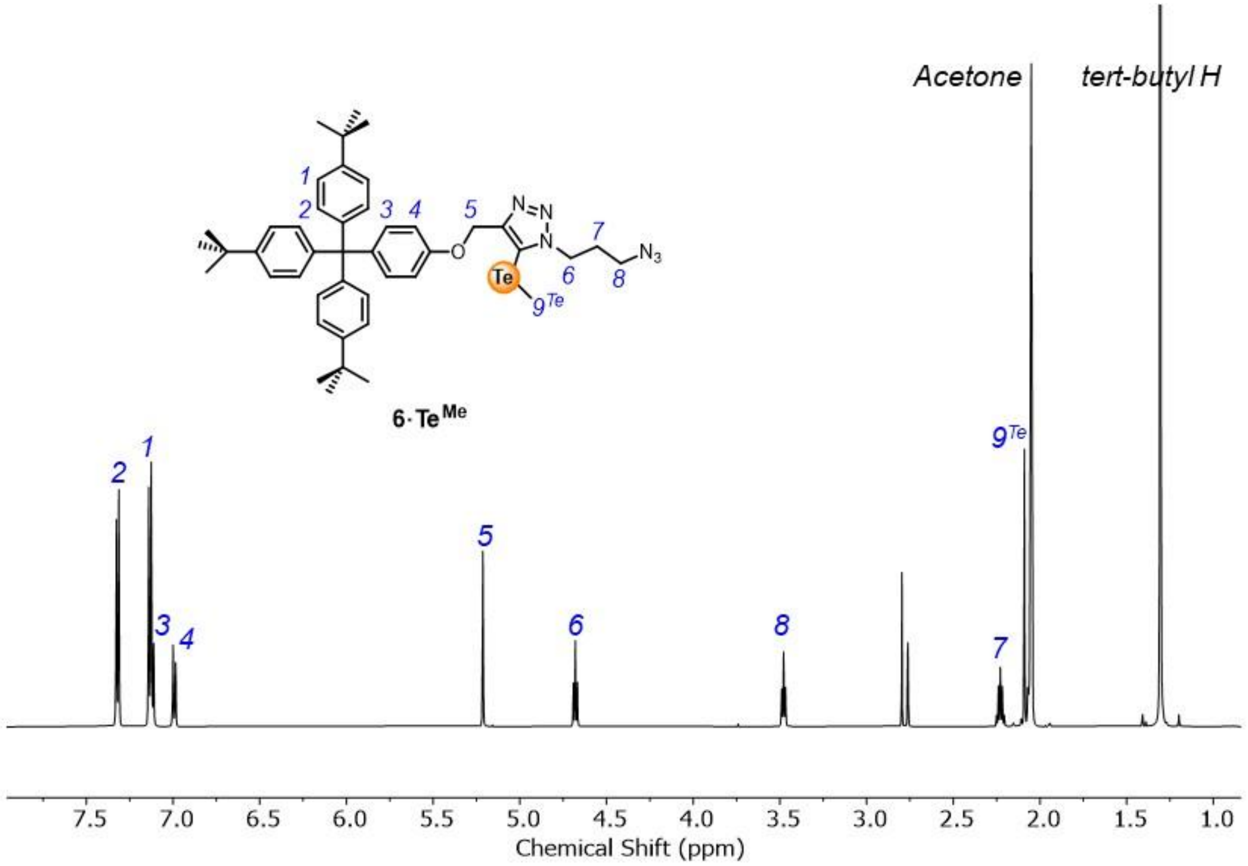

Fig. 21 | ^{1}H NMR spectrum of **6·Te^{Me}** (400 MHz, 298 K, acetone-d_6).

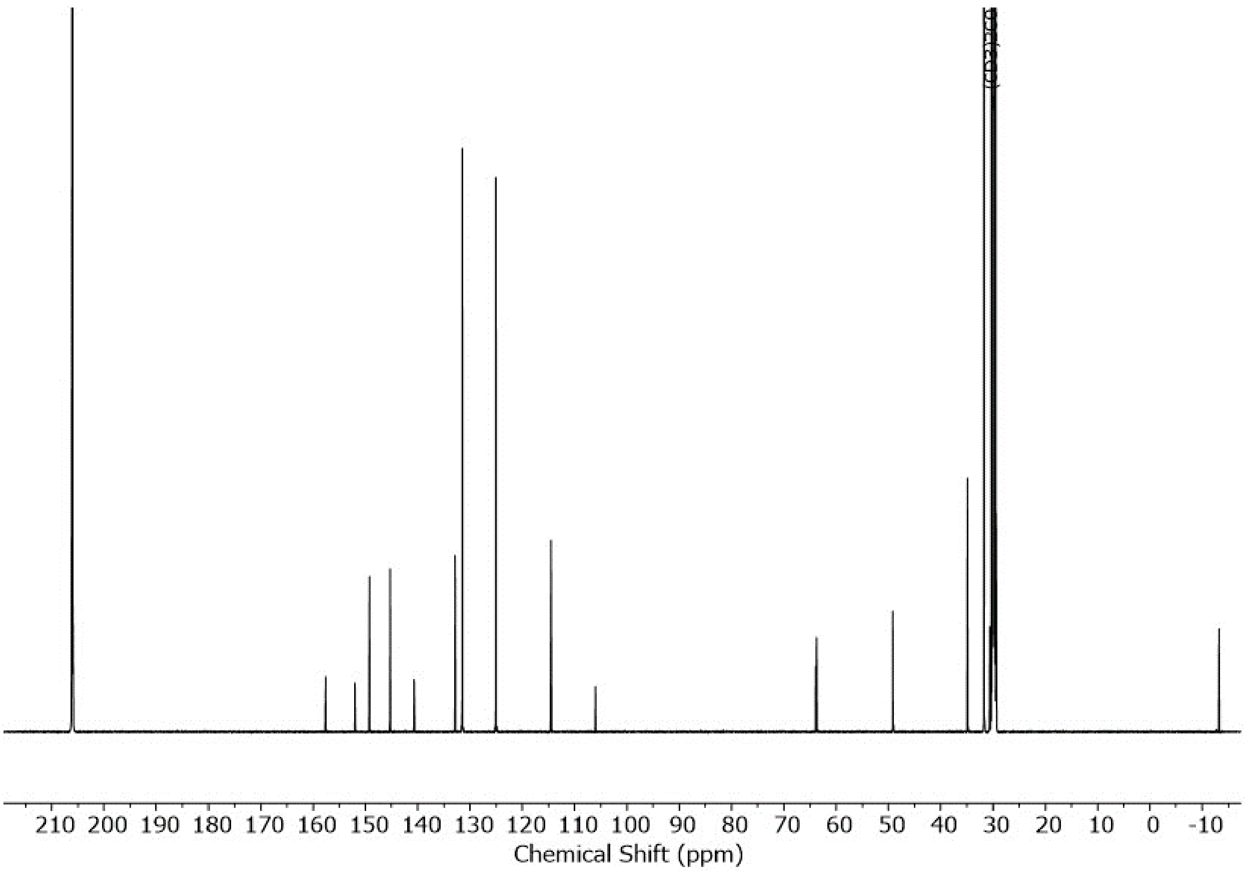

Fig. 22 | ^{13}C NMR spectrum of **6·Te^{Me}** (126 MHz, 298 K, acetone-d_6).

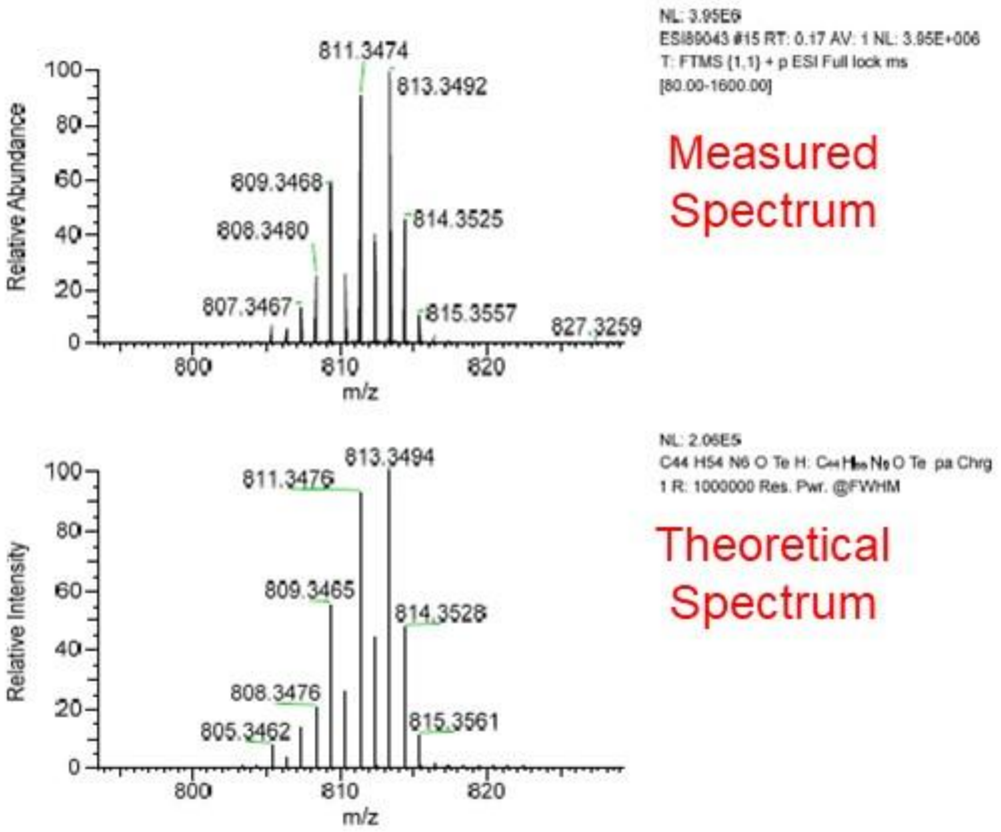

Fig. 23 | Measured (top) and theoretical (bottom) high-resolution ESI mass spectrum of **6·Te^{Me}**.

Spectral Characterisation of Tridentate and Tetradentate [2]Rotaxanes

Tridentate All-ChB [2]Rotaxane (8·ChB$_3$)

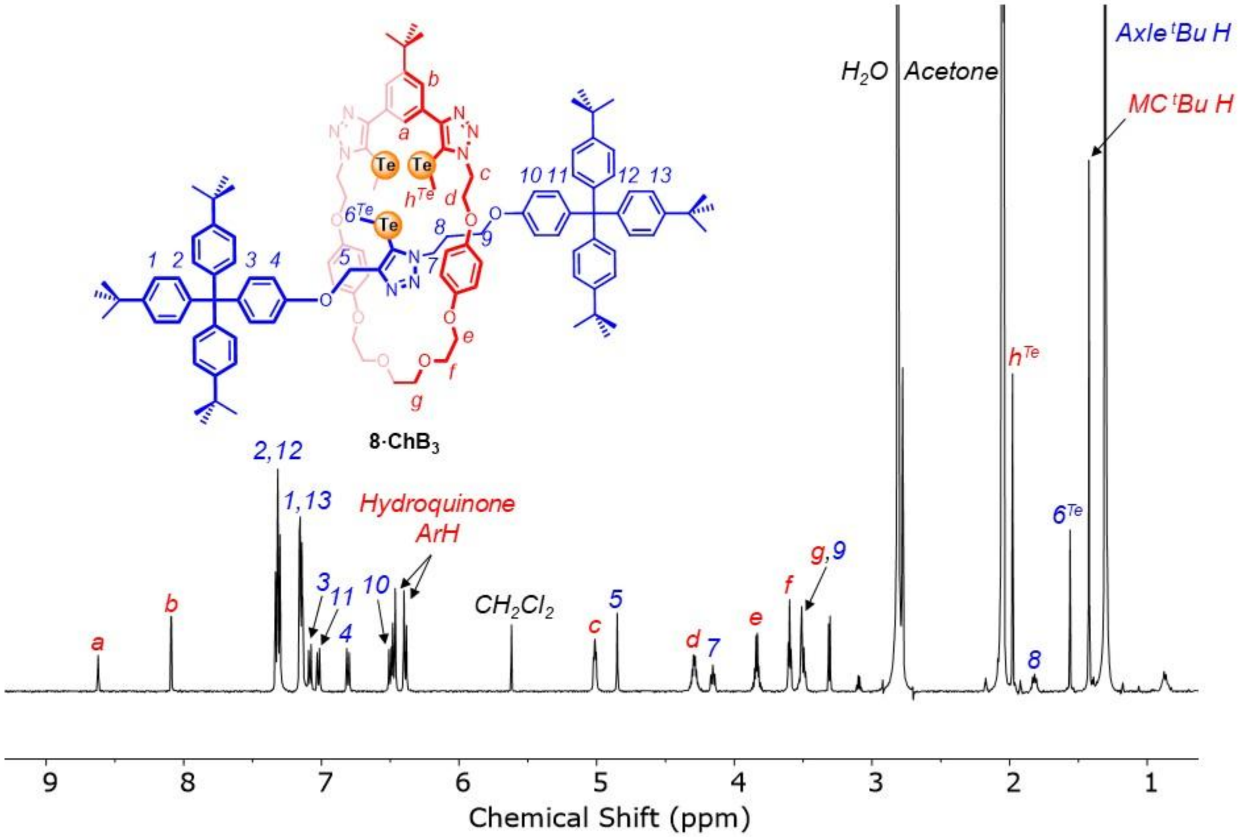

Fig. 24 | ^{1}H NMR spectrum of **8·ChB$_3$** (500 MHz, 298 K, acetone-d_6).

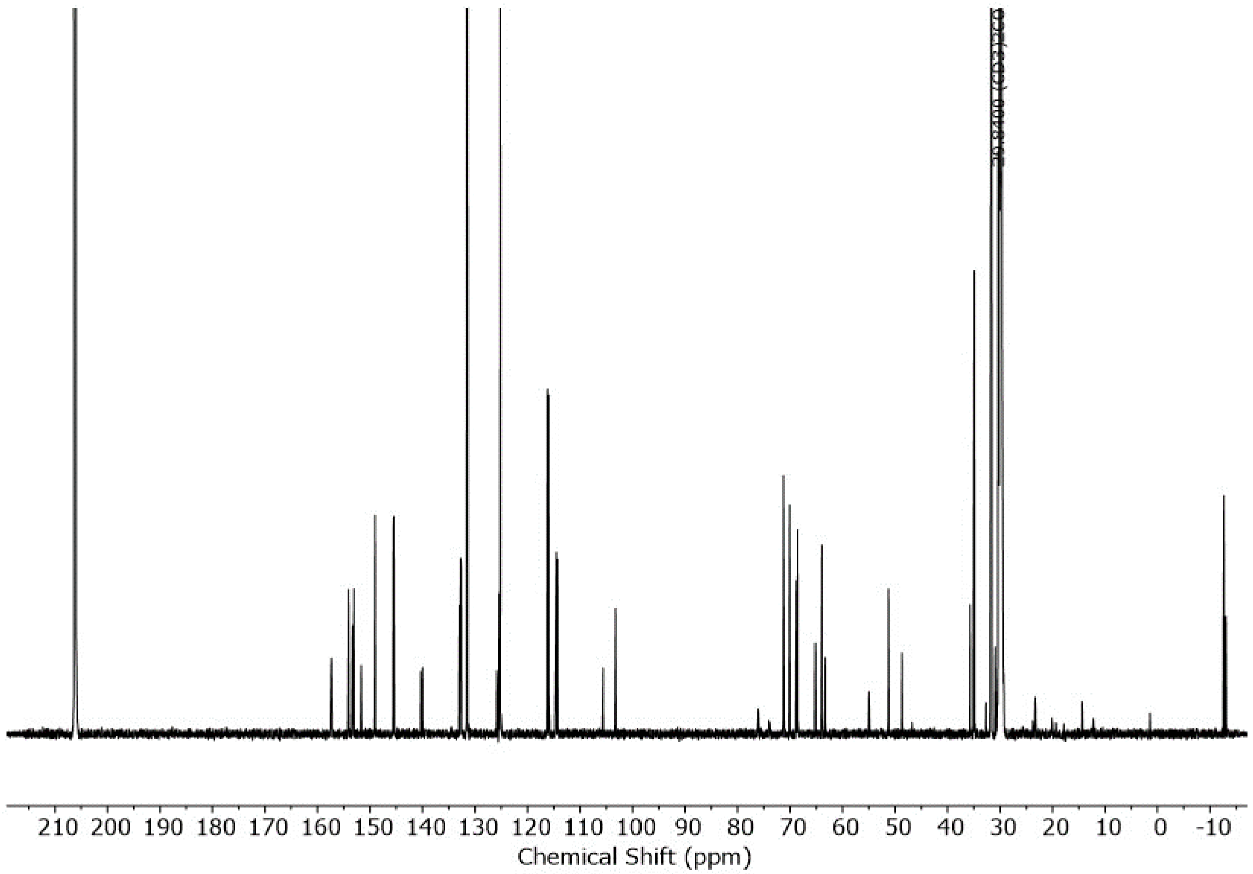

Fig. 25 | ^{13}C NMR spectrum of **8·ChB$_3$** (151 MHz, 298 K, acetone-d_6).

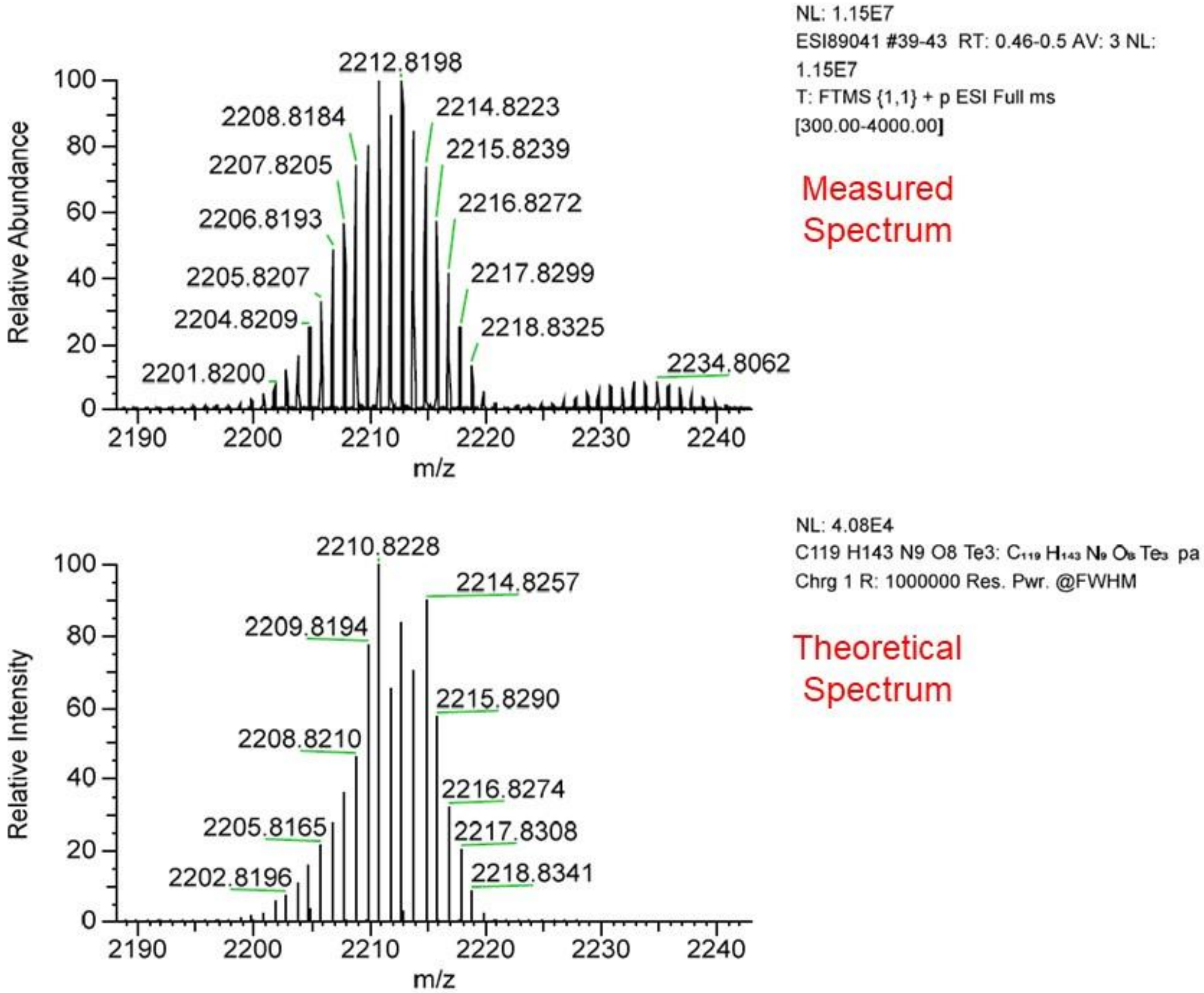

Fig. 26 | Measured (top) and theoretical (bottom) high-resolution ESI mass spectrum of **8·ChB$_3$**.

Tridentate All-XB [2]Rotaxane (8·XB$_3$)

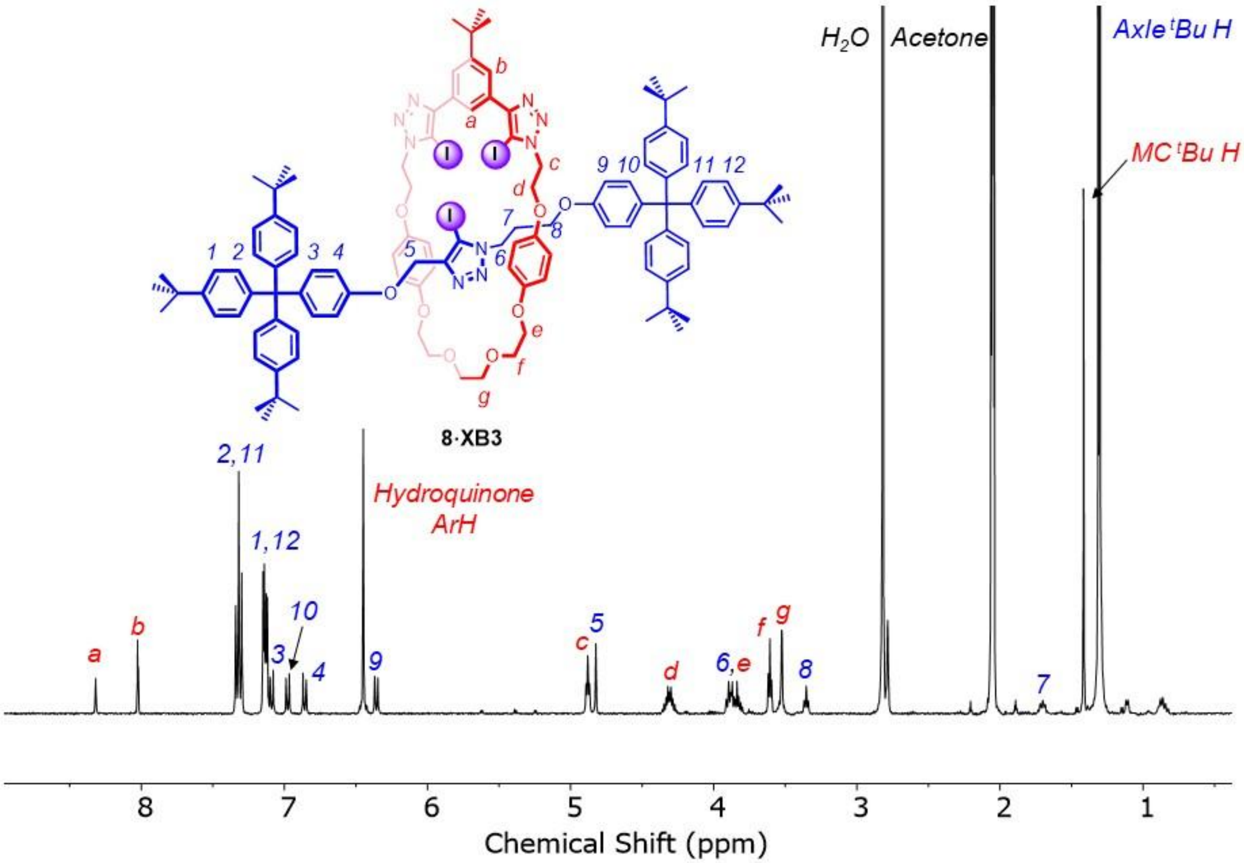

Fig. 27 | ^{1}H NMR spectrum of **8·XB$_3$** (400 MHz, 298 K, acetone-d_6).

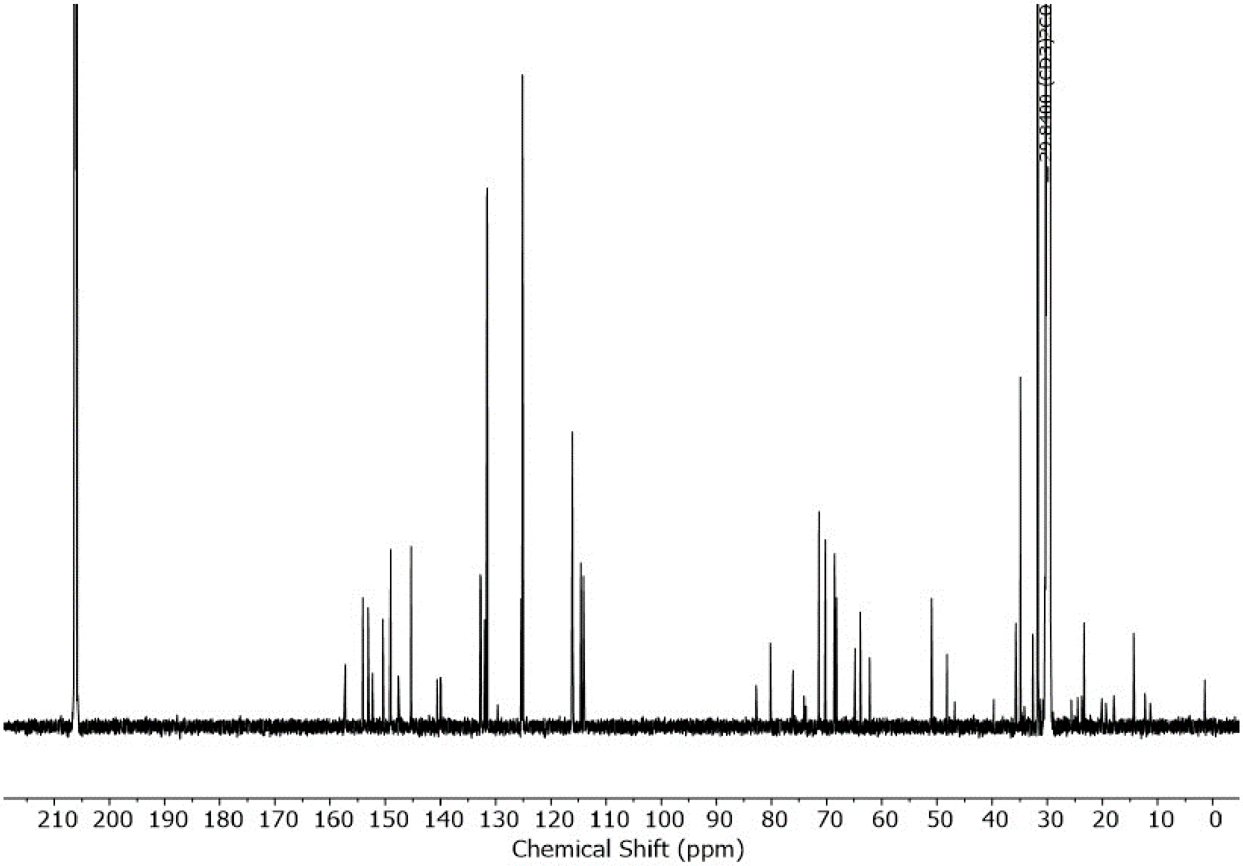

Fig. 28 | ^{13}C NMR spectrum of **8·XB$_3$** (151 MHz, 298 K, acetone-d_6).

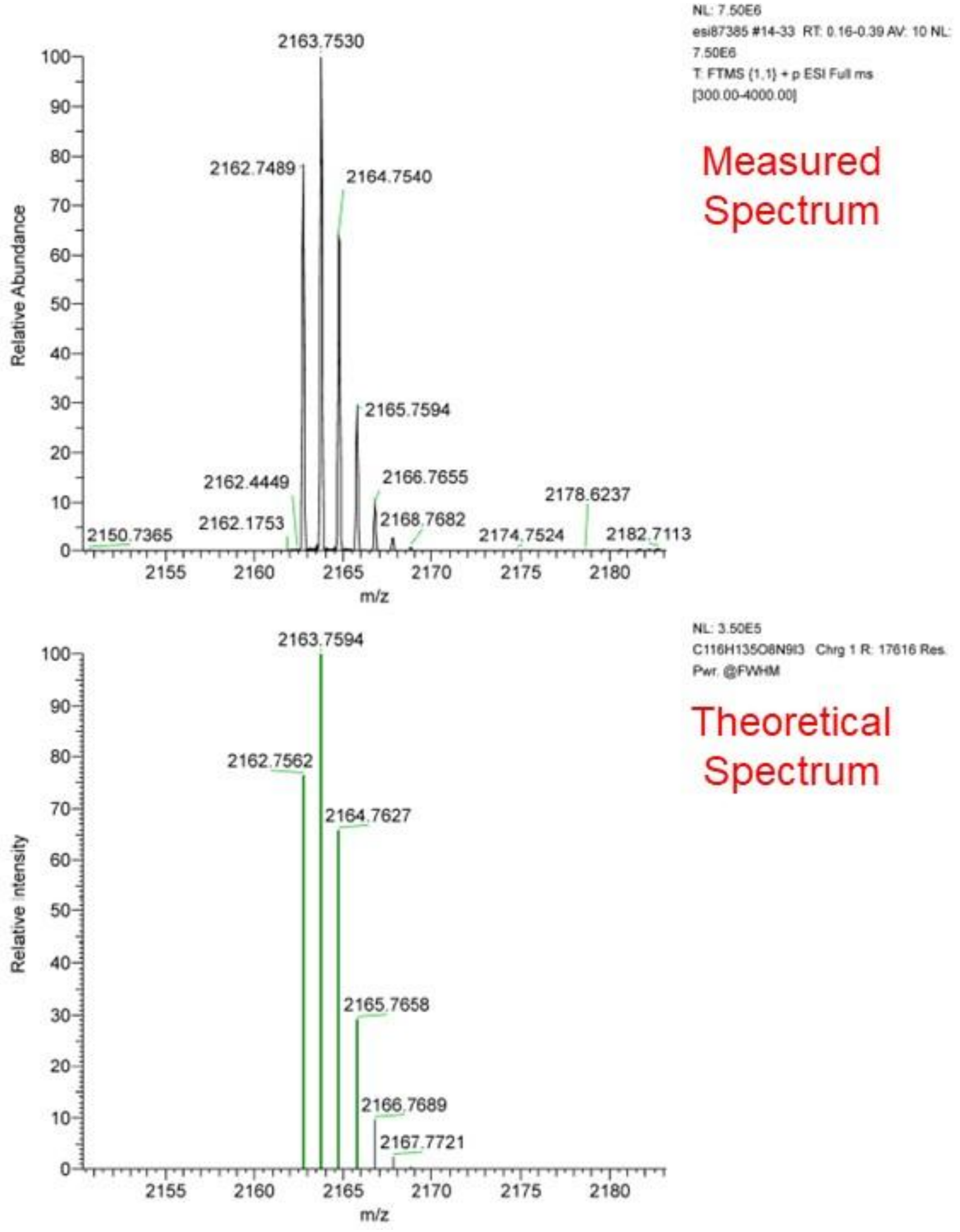

Fig. 29 | Measured (top) and theoretical (bottom) high-resolution ESI mass spectrum of **8·XB$_3$**.

Tridentate Mixed XB/ChB [2]Rotaxane (8·XB_2ChB^{Me})

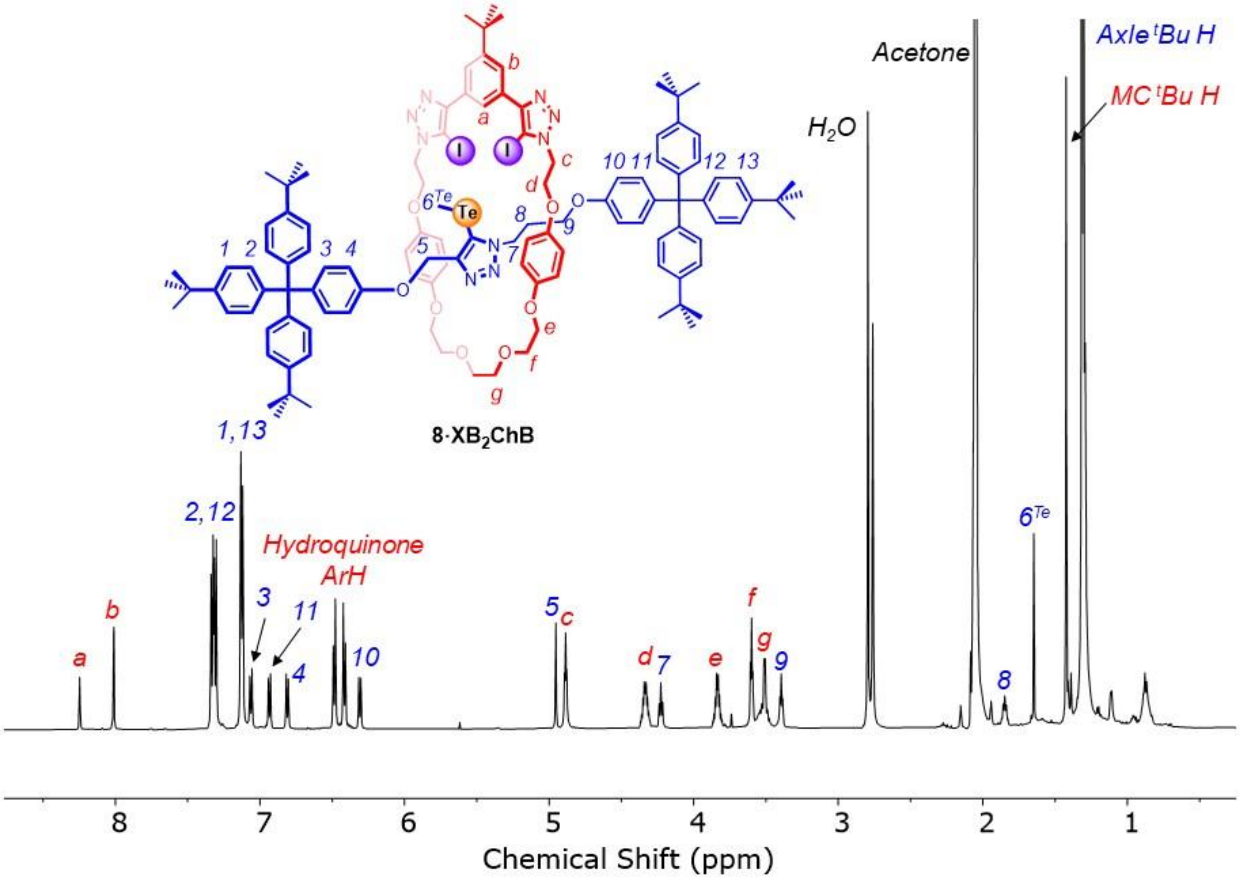

Fig. 30 | ^{1}H NMR spectrum of **8·XB_2ChB^{Me}** (600 MHz, 298 K, acetone-d_6).

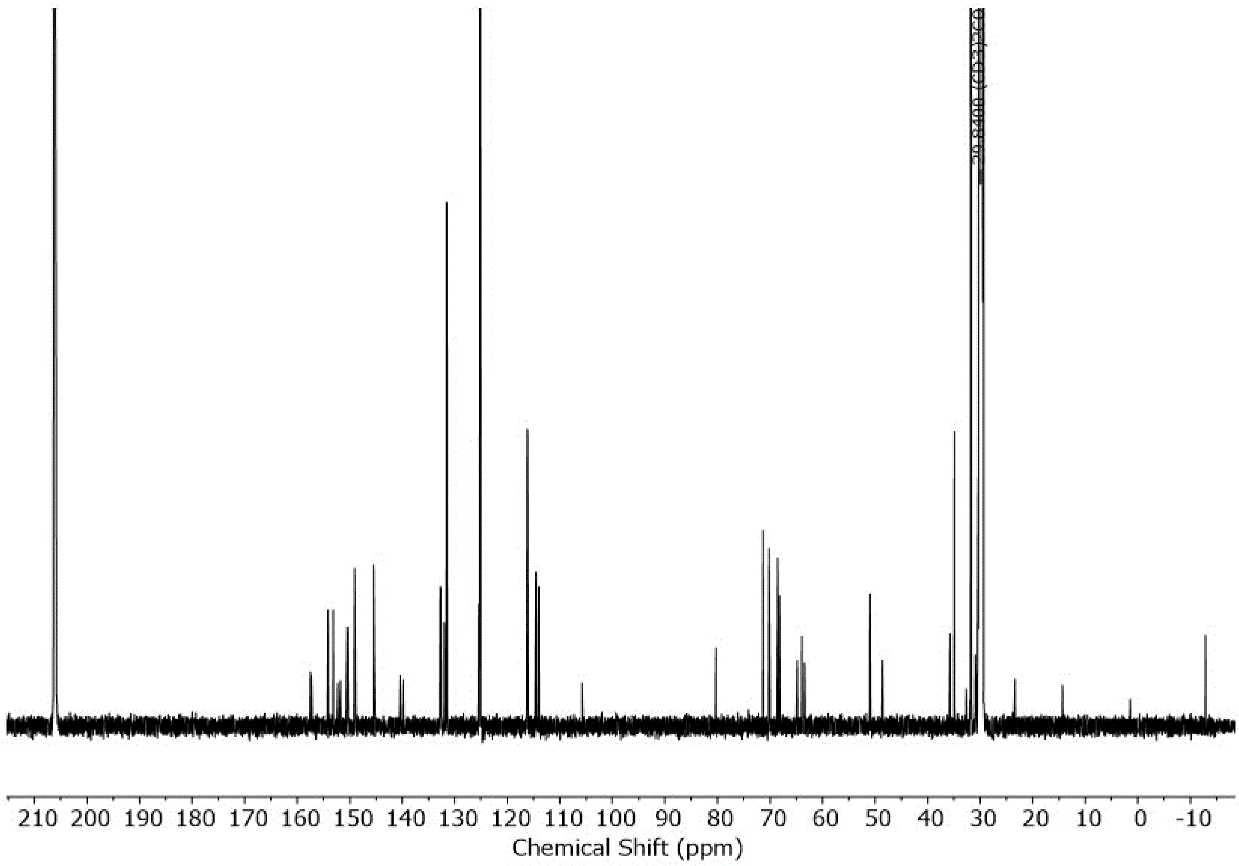

Fig. 31 | ^{13}C NMR spectrum of **8·XB$_2$ChB^{Me}** (151 MHz, 298 K, acetone-d_6).

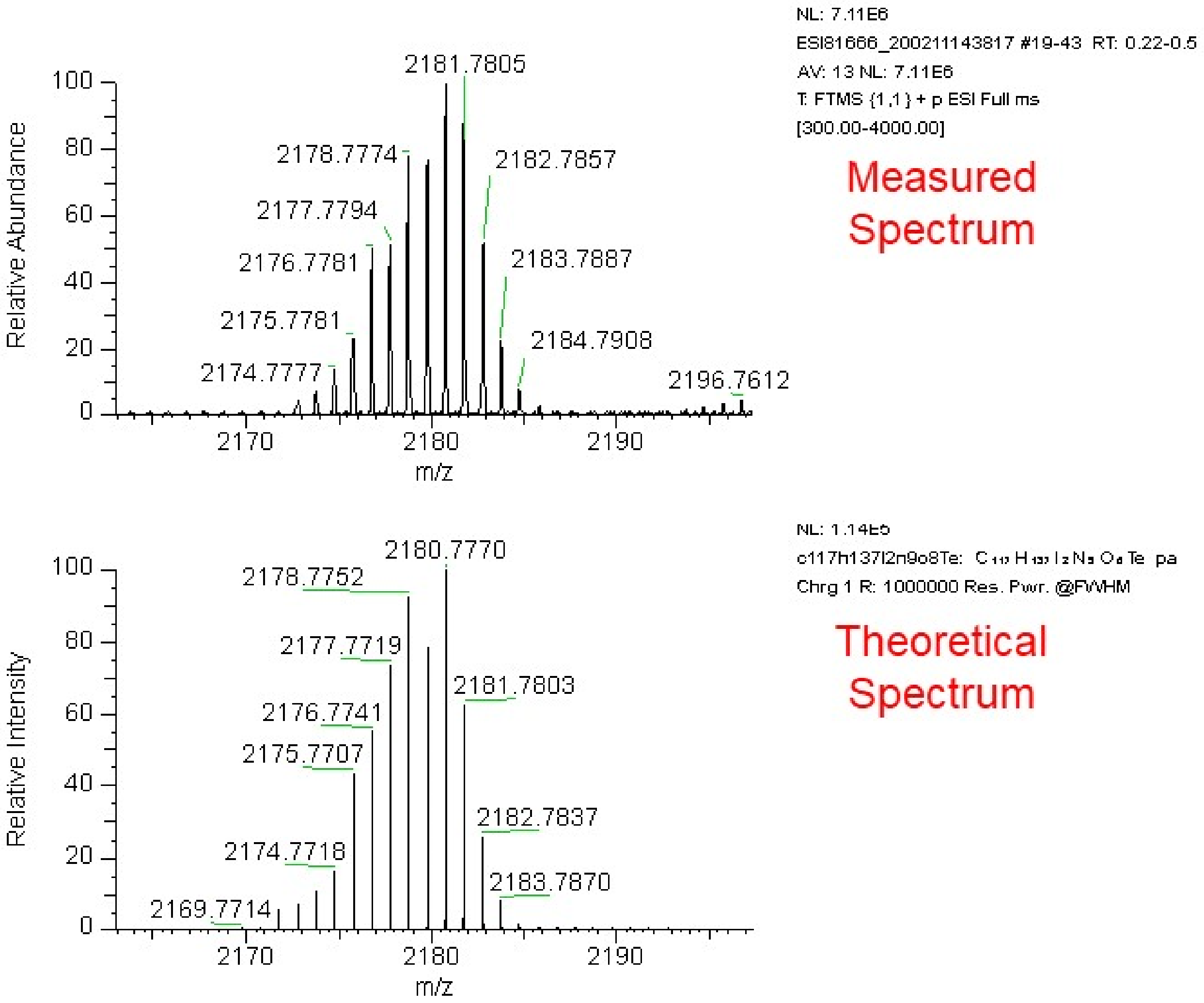

Fig. 32 | Measured (top) and theoretical (bottom) high-resolution ESI mass spectrum of **8·XB$_2$ChB^{Me}**.

Tridentate Mixed XB/ChB [2]Rotaxane (8·XB$_2$ChB^{Ph})

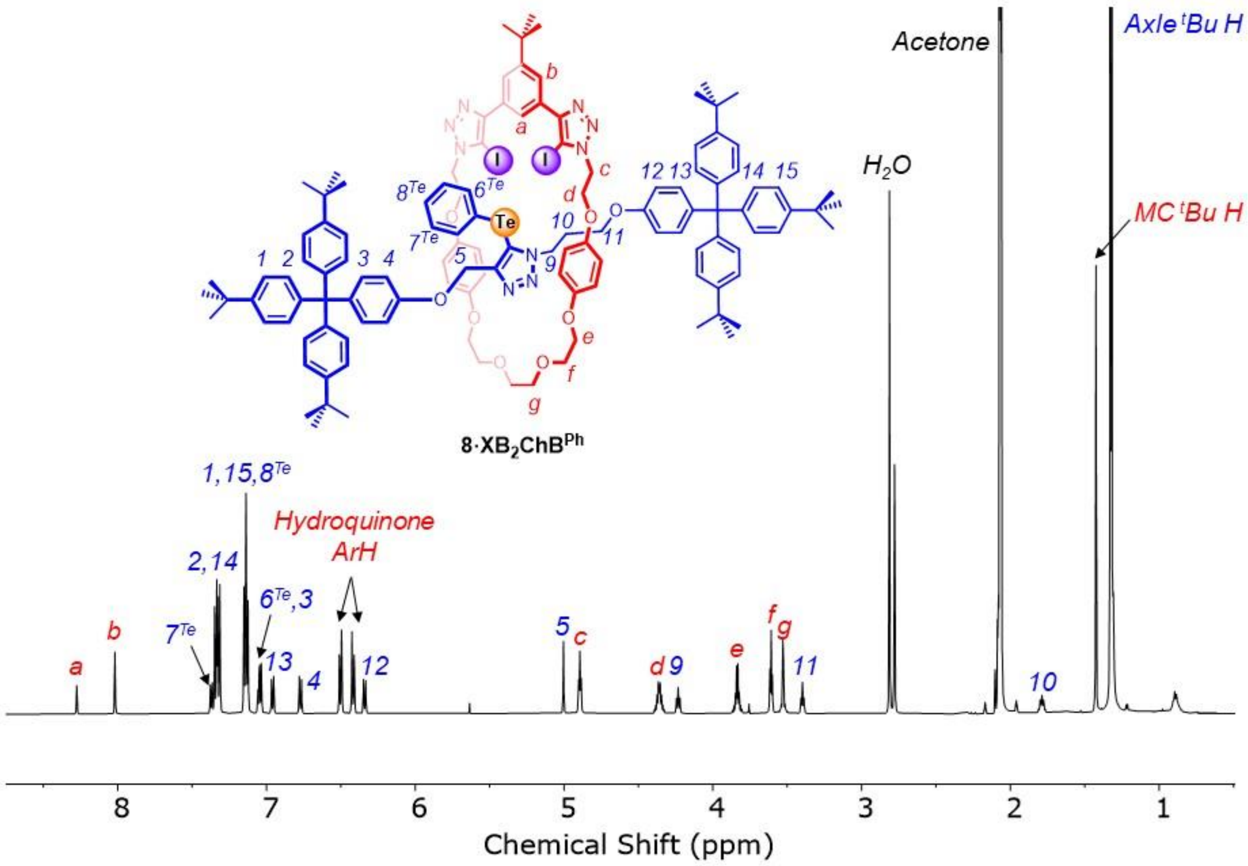

Fig. 33 | ^{1}H NMR spectrum of **8·XB$_2$ChB^{Ph}** (600 MHz, 298 K, acetone-d_6).

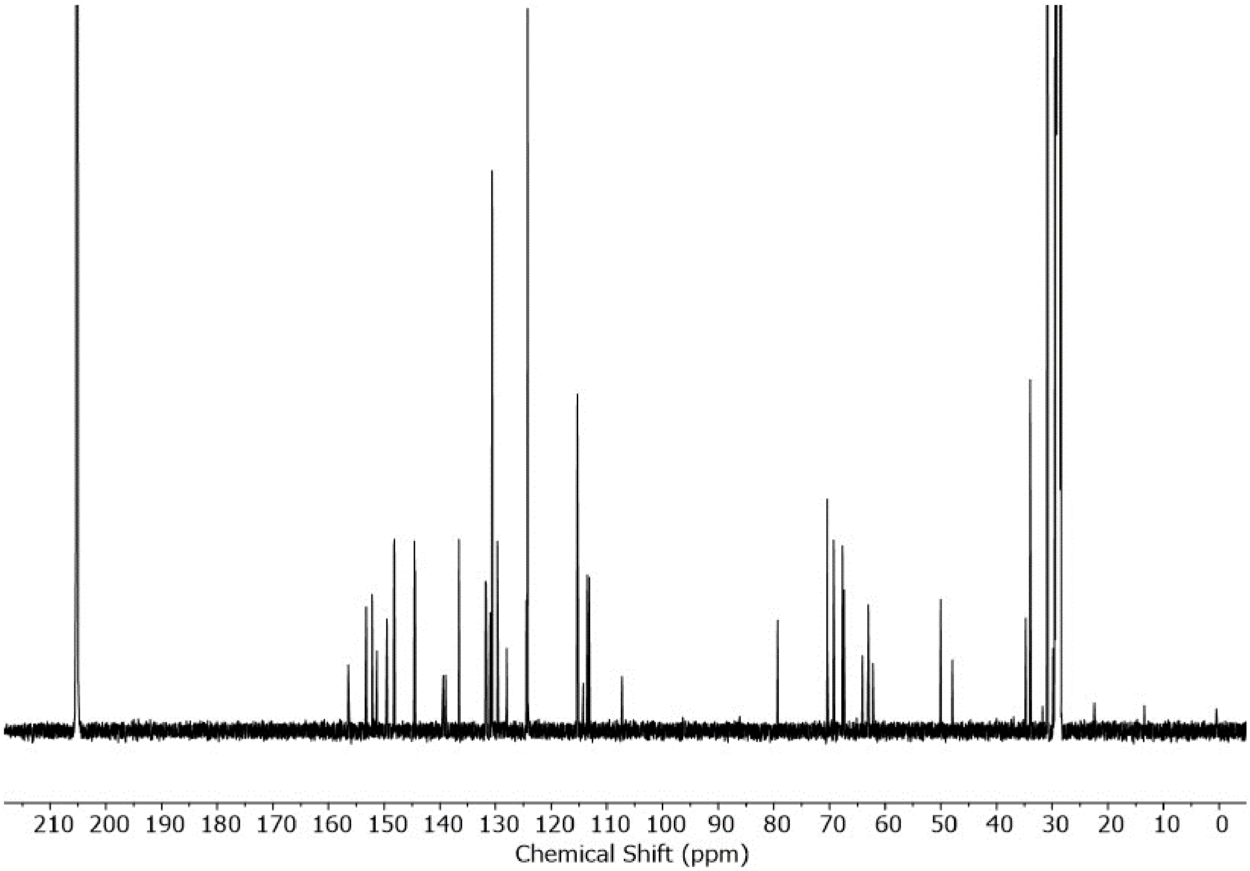

Fig. 34 | ^{13}C NMR spectrum of **8·XB$_2$ChB^{Ph}** (151 MHz, 298 K, acetone-d_6).

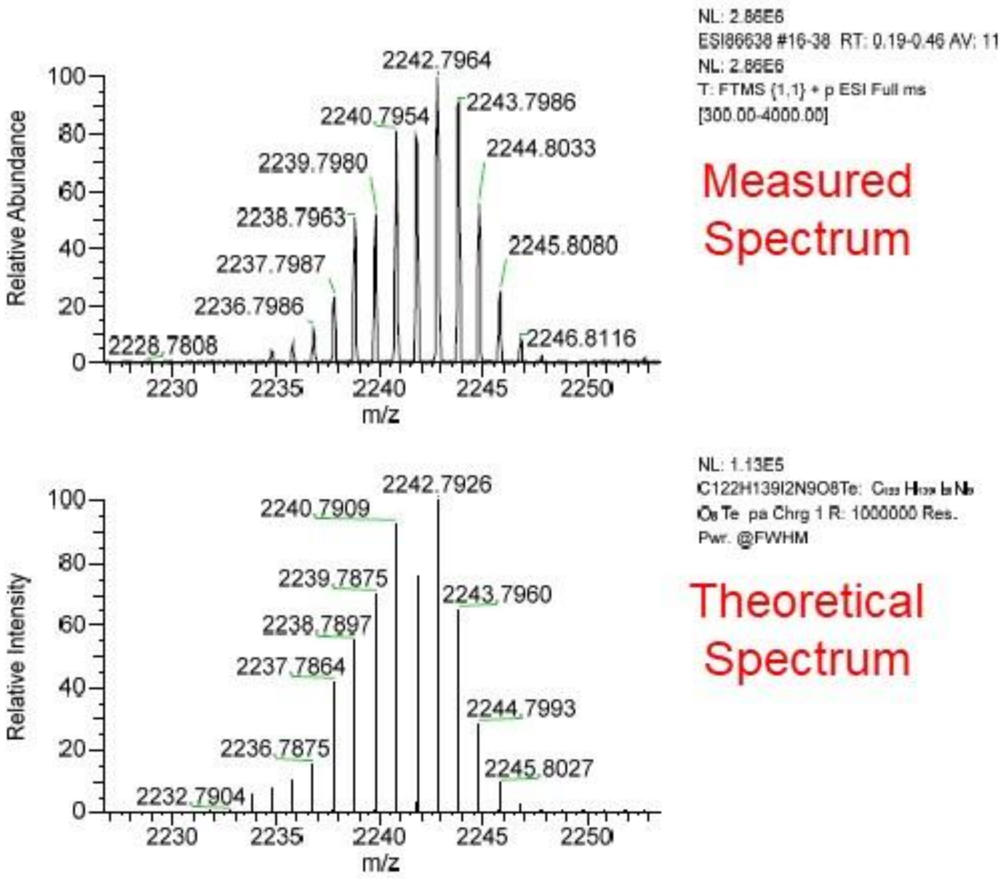

Fig. 35 | Measured (top) and theoretical (bottom) high-resolution ESI mass spectrum of **8·XB$_2$ChB^{Ph}**.

Tridentate Mixed XB/ChB [2]Rotaxane (8·XB$_2$ChB^{pCF3})

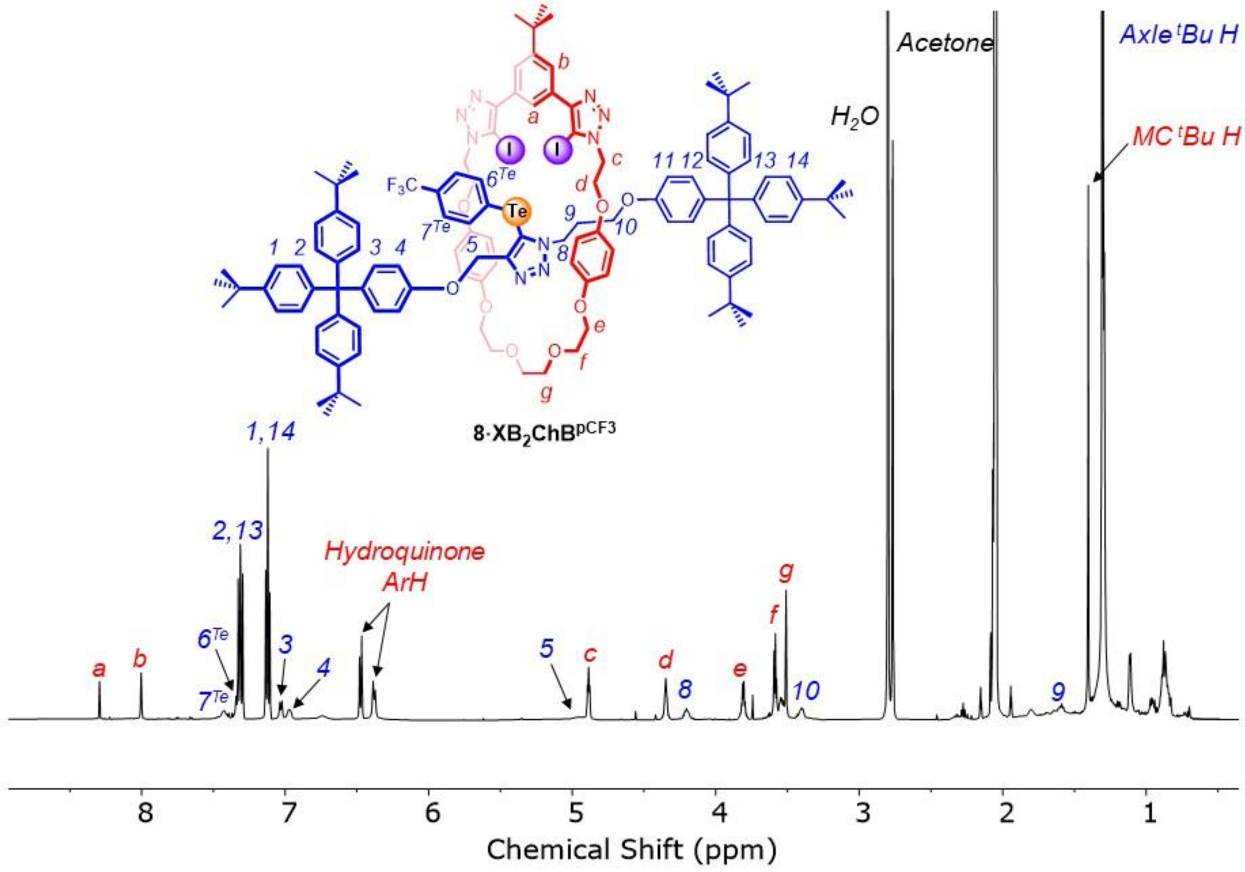

Fig. 36 | ^{1}H NMR spectrum of **8·XB$_2$ChB^{pCF3}** (600 MHz, 298 K, acetone-d_6).

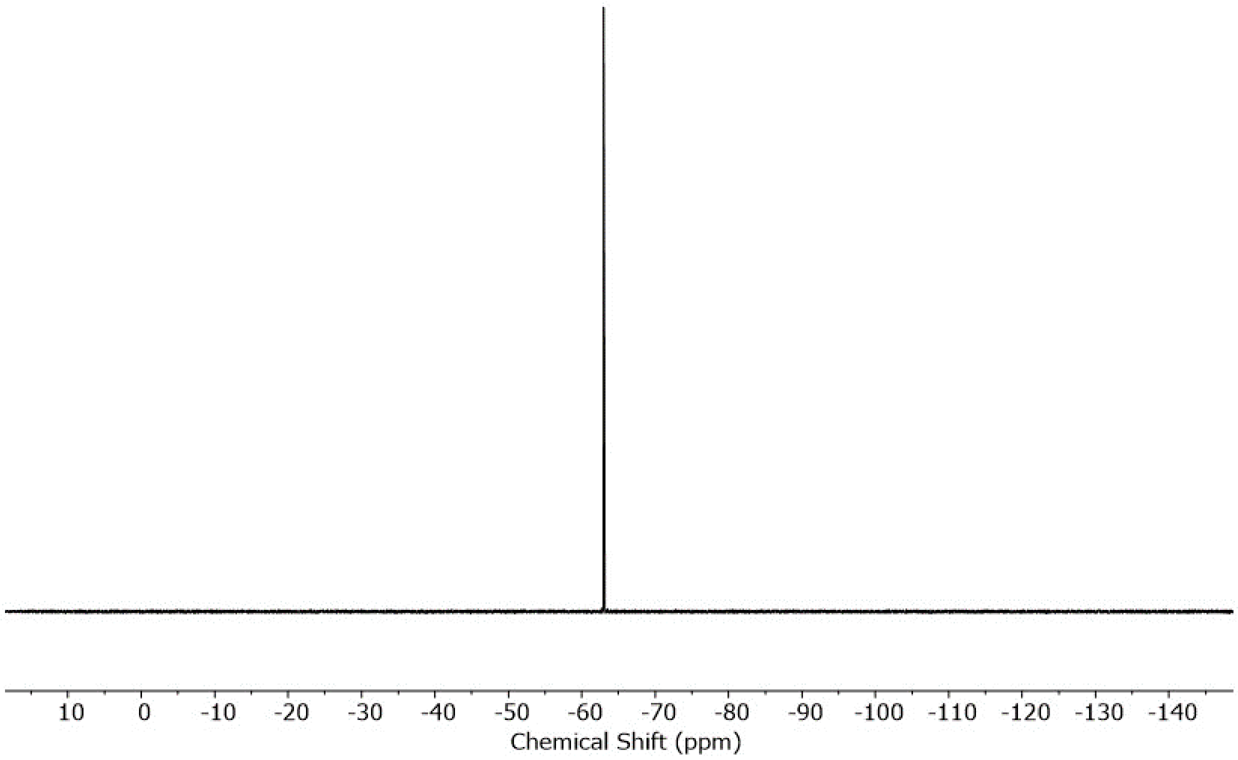

Fig. 37 | ^{19}F NMR spectrum of **8·XB$_2$ChB^{pCF3}** (377 MHz, 298 K, acetone-d_6).

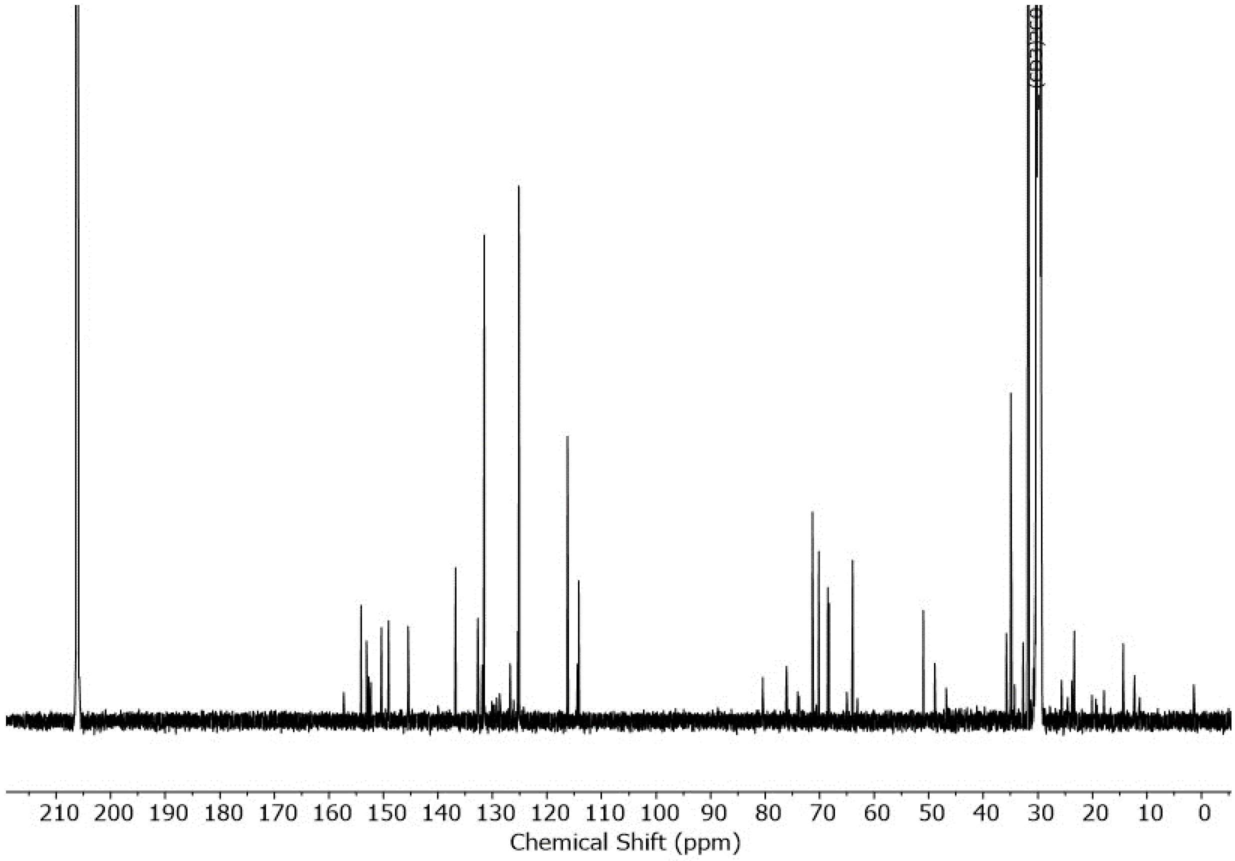

Fig. 38 | ^{13}C NMR spectrum of **8·XB$_2$ChB^{pCF3}** (151 MHz, 298 K, acetone-d_6).

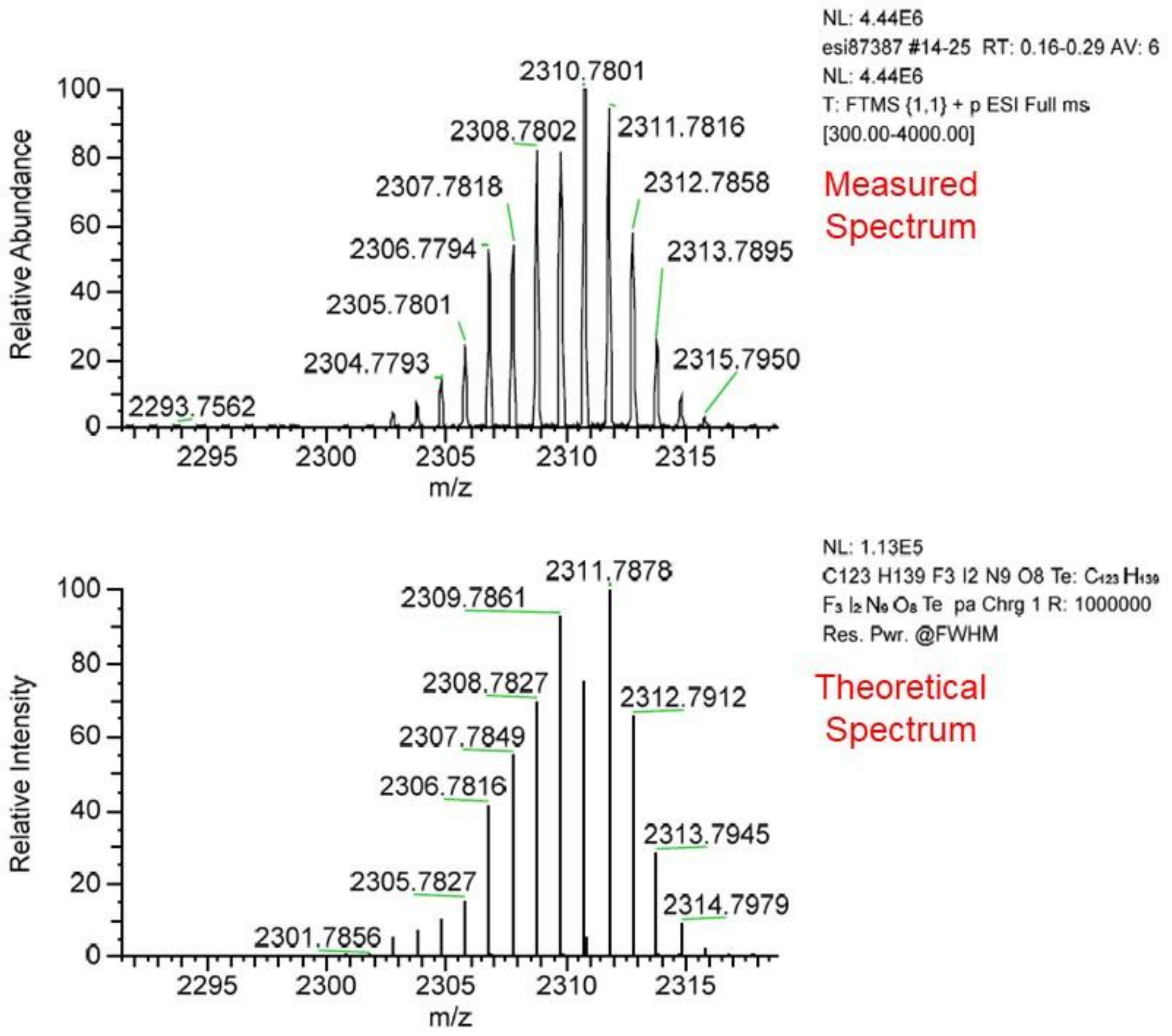

Fig. 39 | Measured (top) and theoretical (bottom) high-resolution ESI mass spectrum of **8·XB$_2$ChB^{pCF3}**.

Tridentate All-HB [2]Rotaxane (8·HB$_3$)

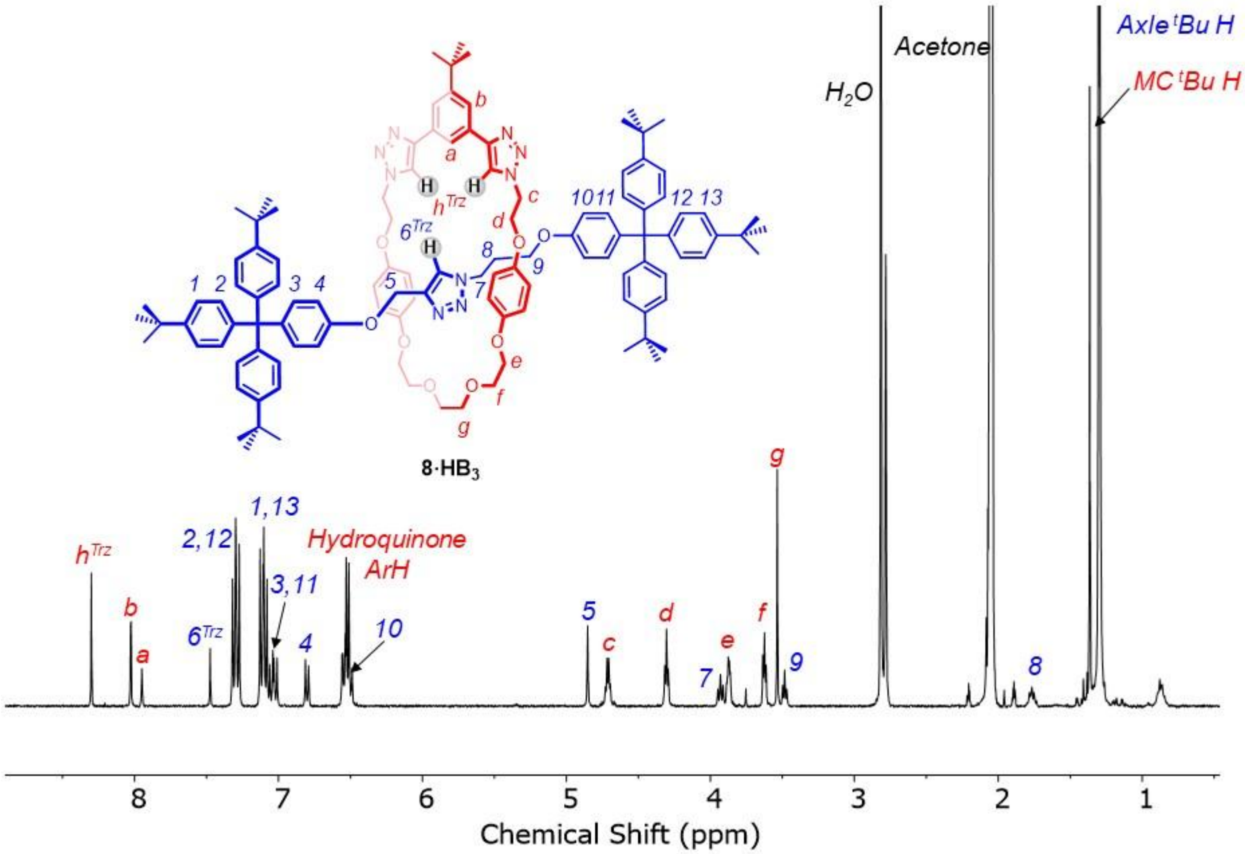

Fig. 40 | ^{1}H NMR spectrum of **8·HB$_3$** (400 MHz, 298 K, acetone-d_6).

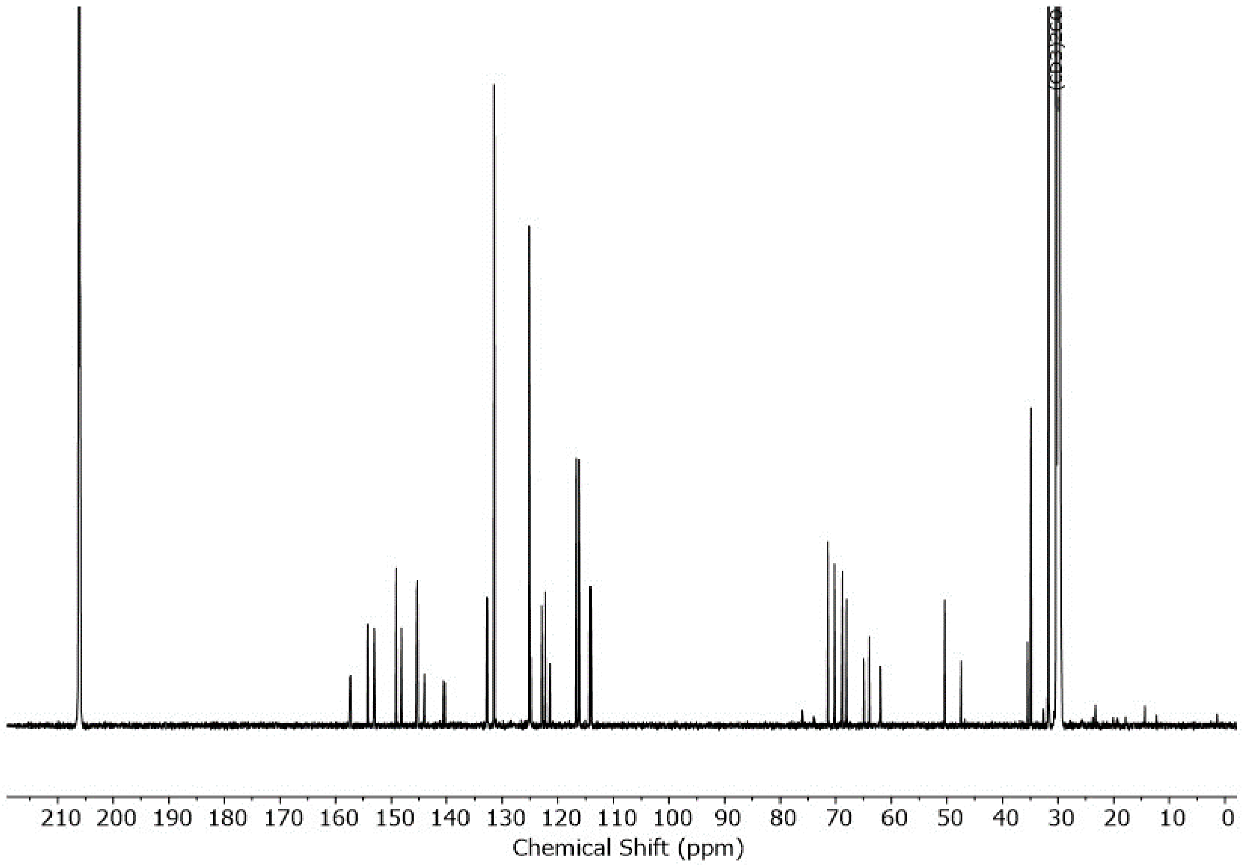

Fig. 41 | ^{13}C NMR spectrum of **8·HB$_3$** (151 MHz, 298 K, acetone-d_6).

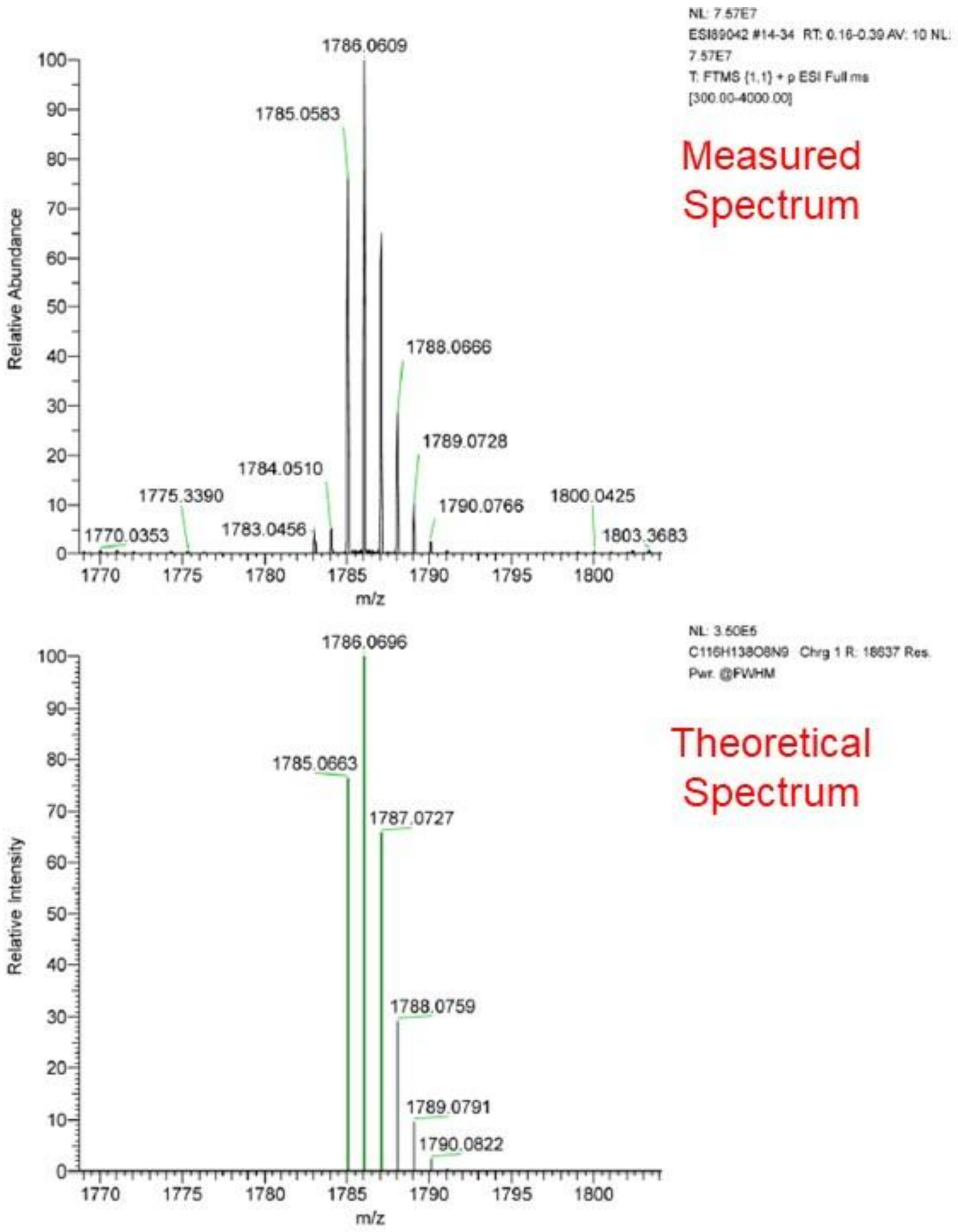

Fig. 42 | Measured (top) and theoretical (bottom) high-resolution ESI mass spectrum of **8·HB$_3$**.

Tetradentate All-ChB [2]Rotaxane (9·ChB$_4$)

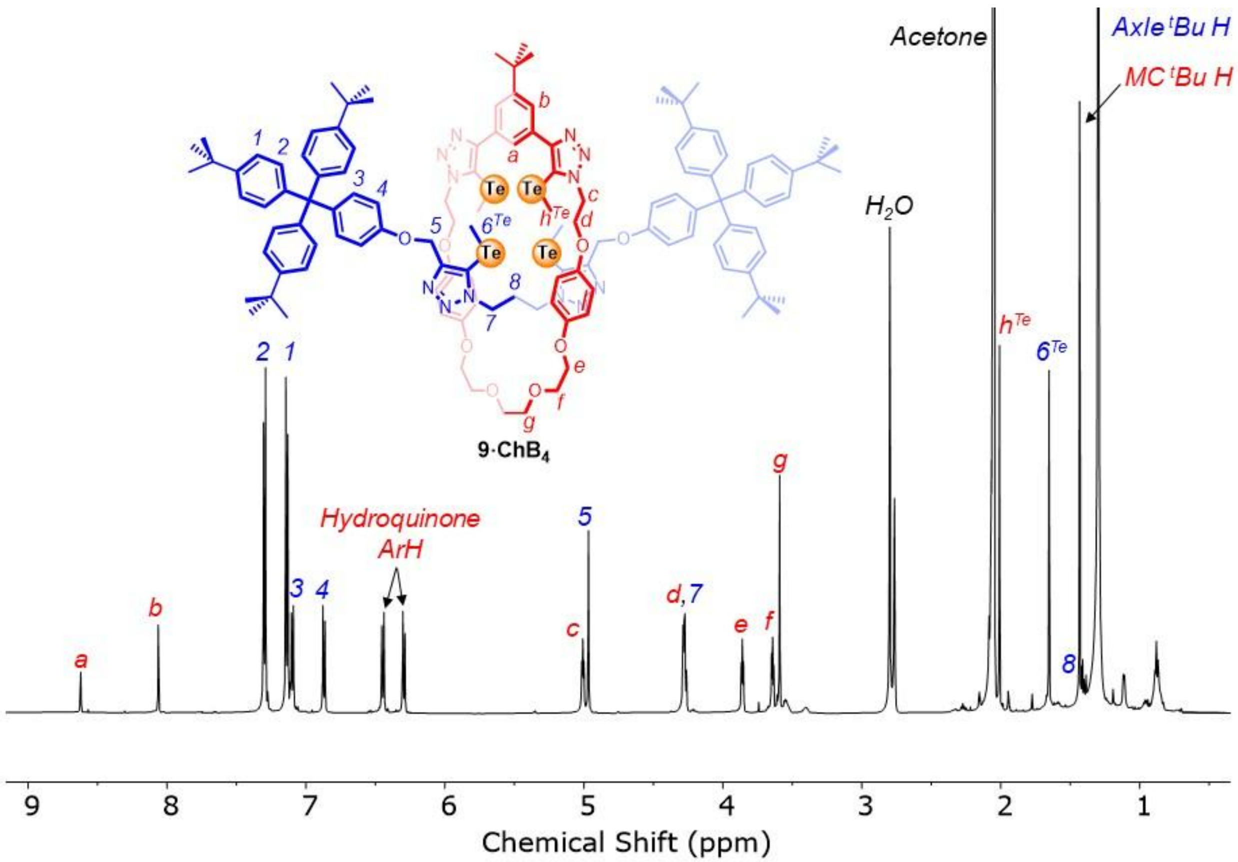

Fig. 43 | ^{1}H NMR spectrum of **9·ChB$_4$** (600 MHz, 298 K, acetone-d_6).

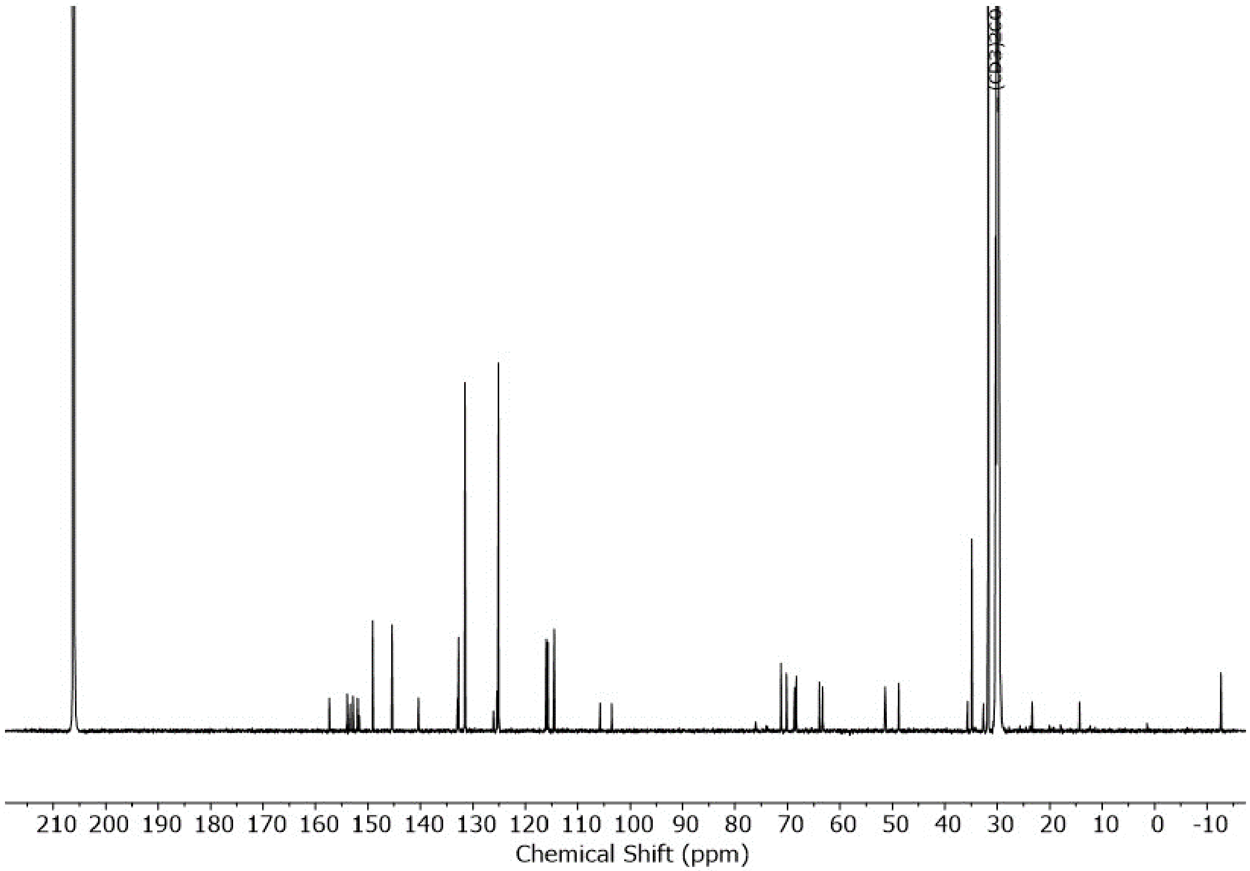

Fig. 44 | ^{13}C NMR spectrum of **9·ChB$_4$** (151 MHz, 298 K, acetone-d_6).

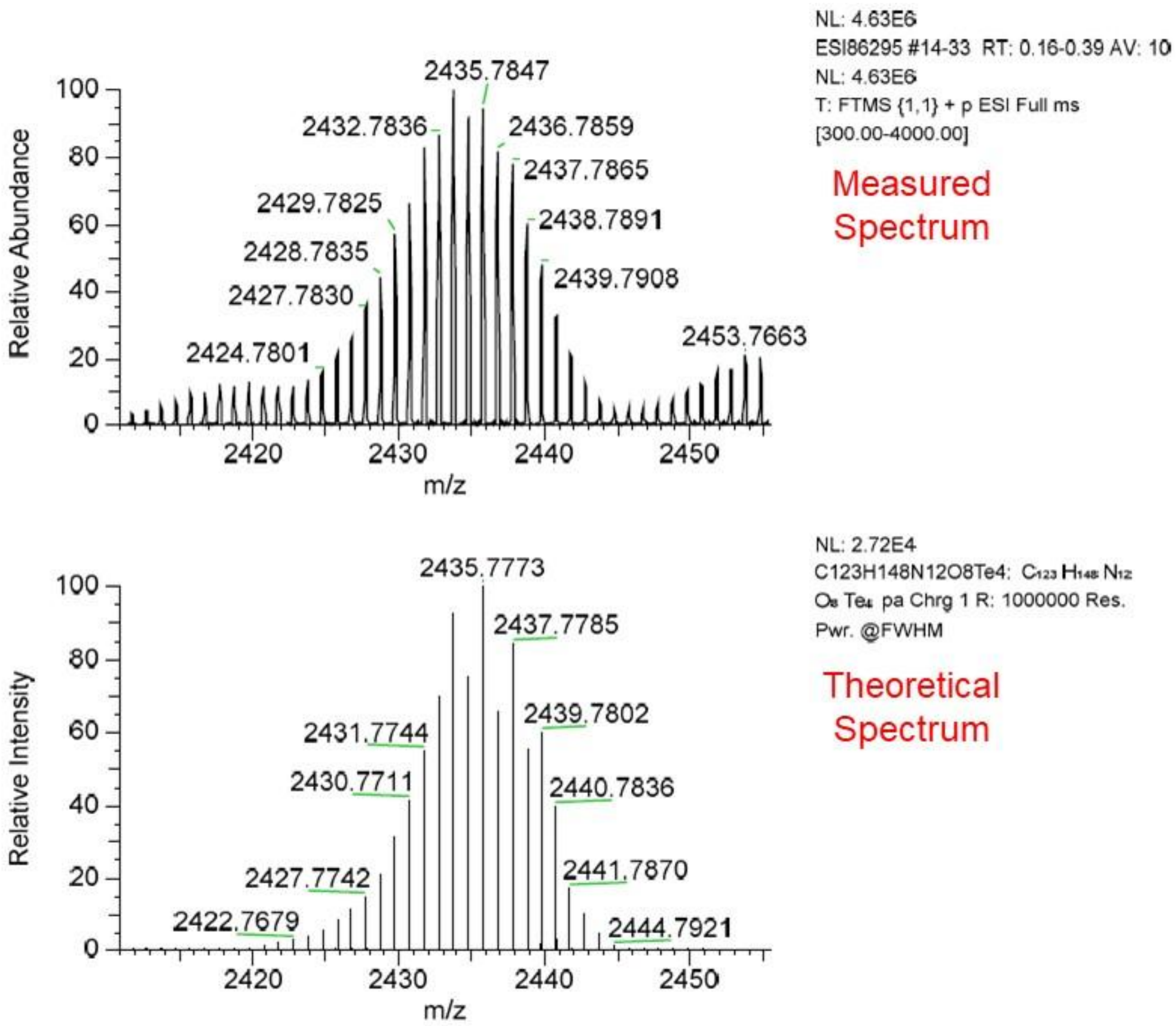

Fig. 45 | Measured (top) and theoretical (bottom) high-resolution ESI mass spectrum of **9·ChB$_4$**.

Tetradentate Mixed XB/ChB [2]Rotaxane ($9\cdot XB_2ChB_2$)

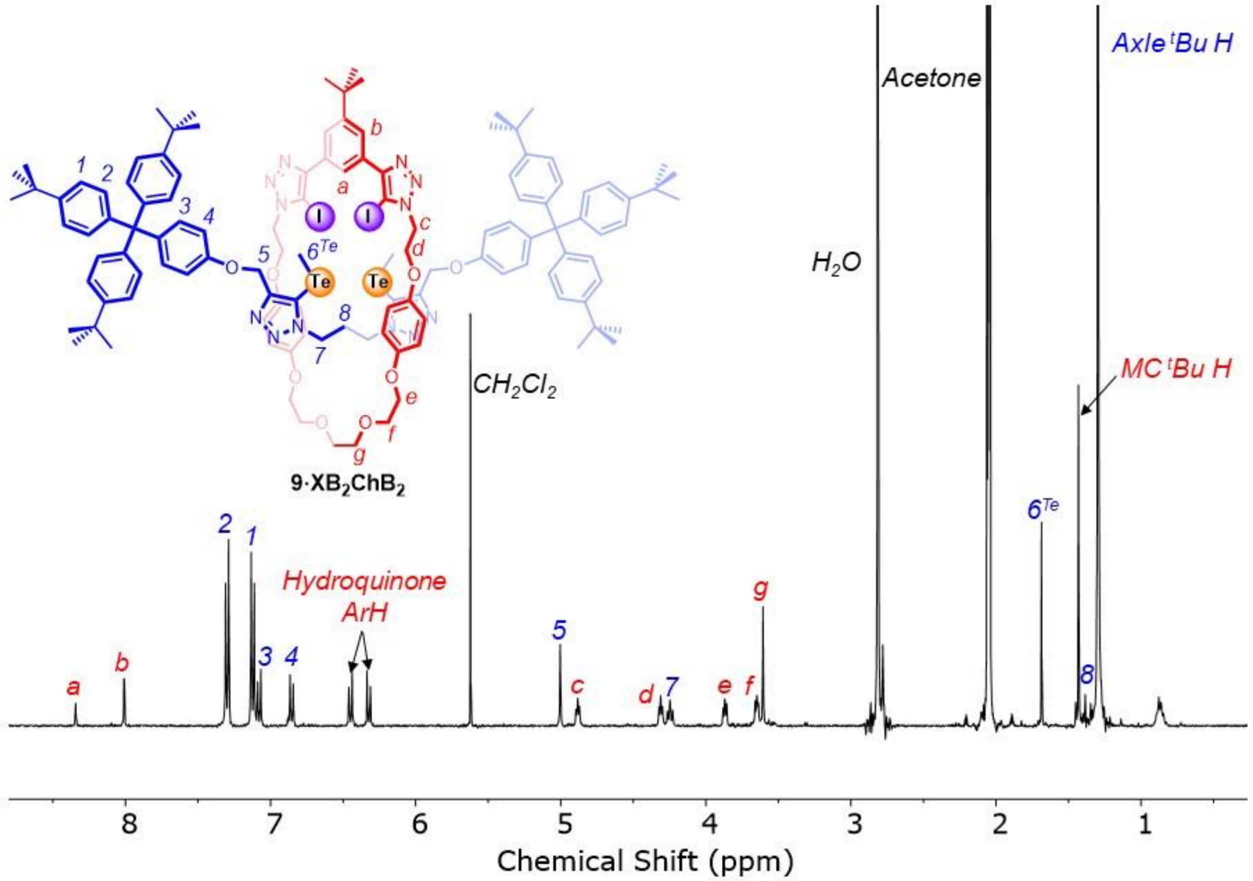

Fig. 46 | ^{1}H NMR spectrum of **$9\cdot XB_2ChB_2$** (400 MHz, 298 K, acetone-d_6).

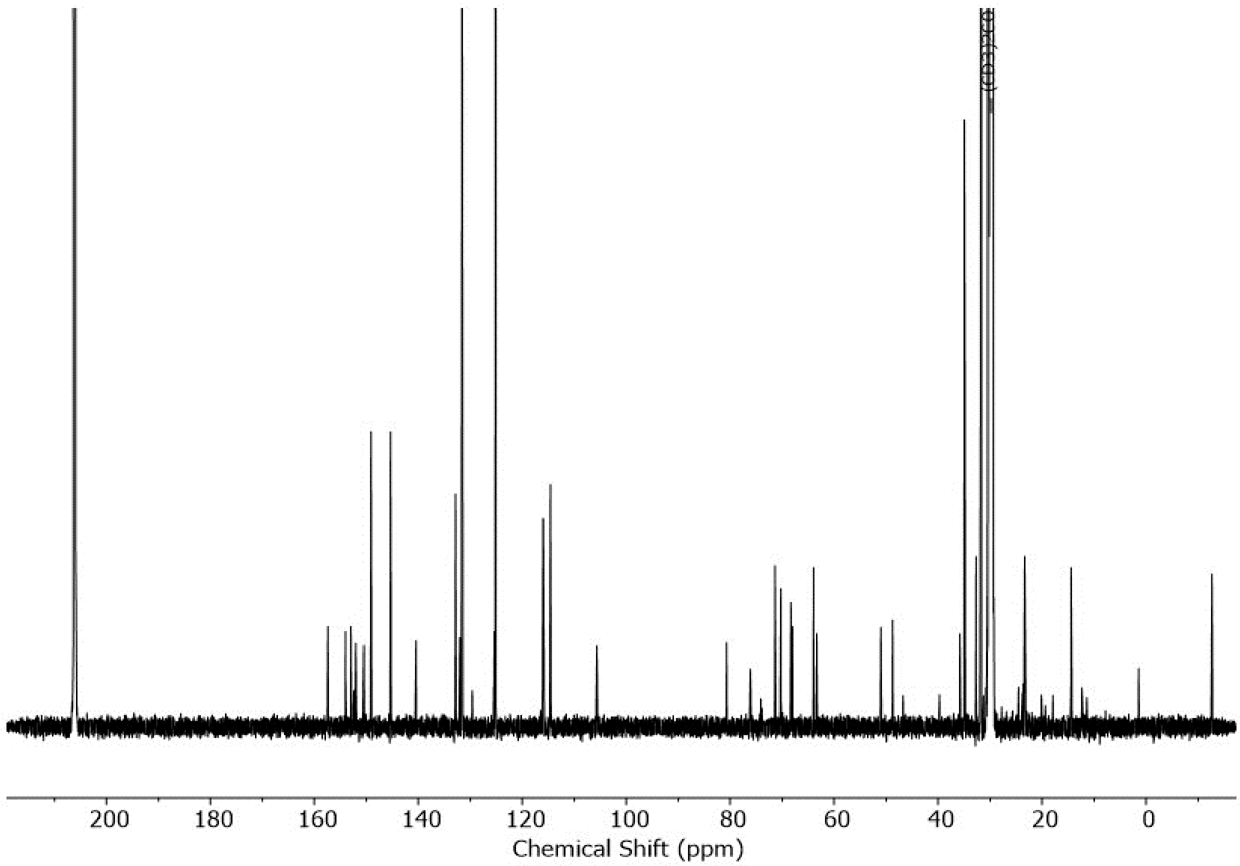

Fig. 47 | ^{13}C NMR spectrum of **9·XB$_2$ChB$_2$** (151 MHz, 298 K, acetone-d_6).

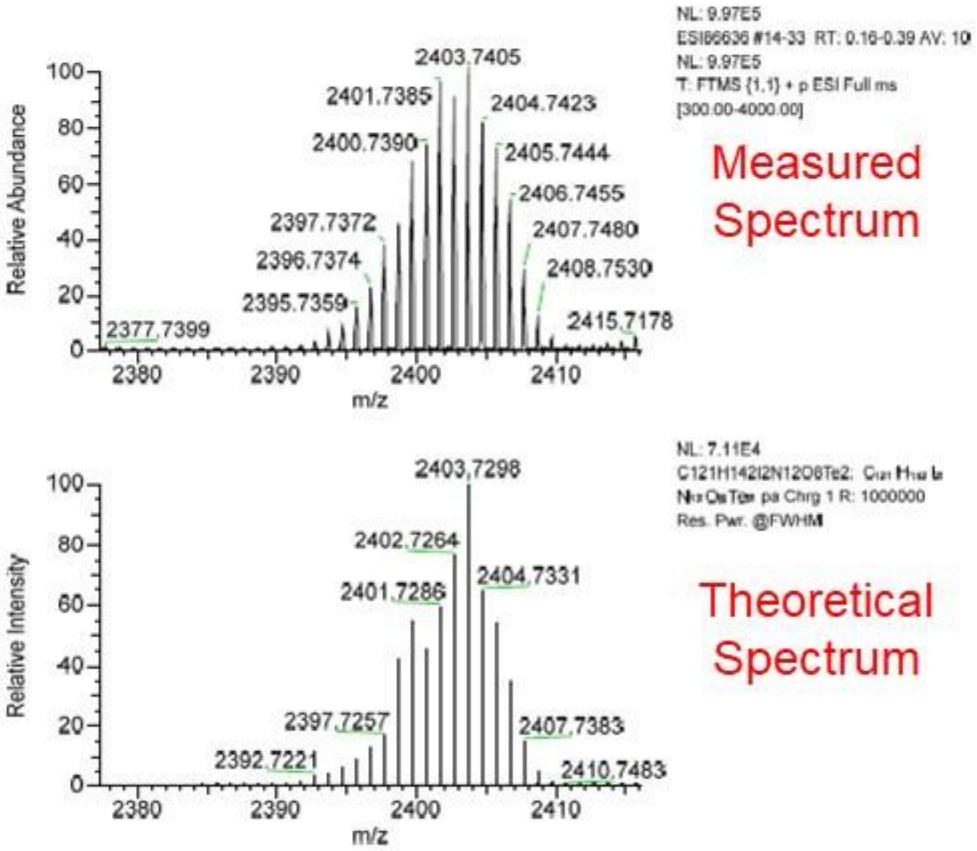

Fig. 48 | Measured (top) and theoretical (bottom) high-resolution ESI mass spectrum of **9·XB$_2$ChB$_2$**.

Tetradentate Mixed XB/ChB [2]Rotaxane (9·XB$_3$ChB)

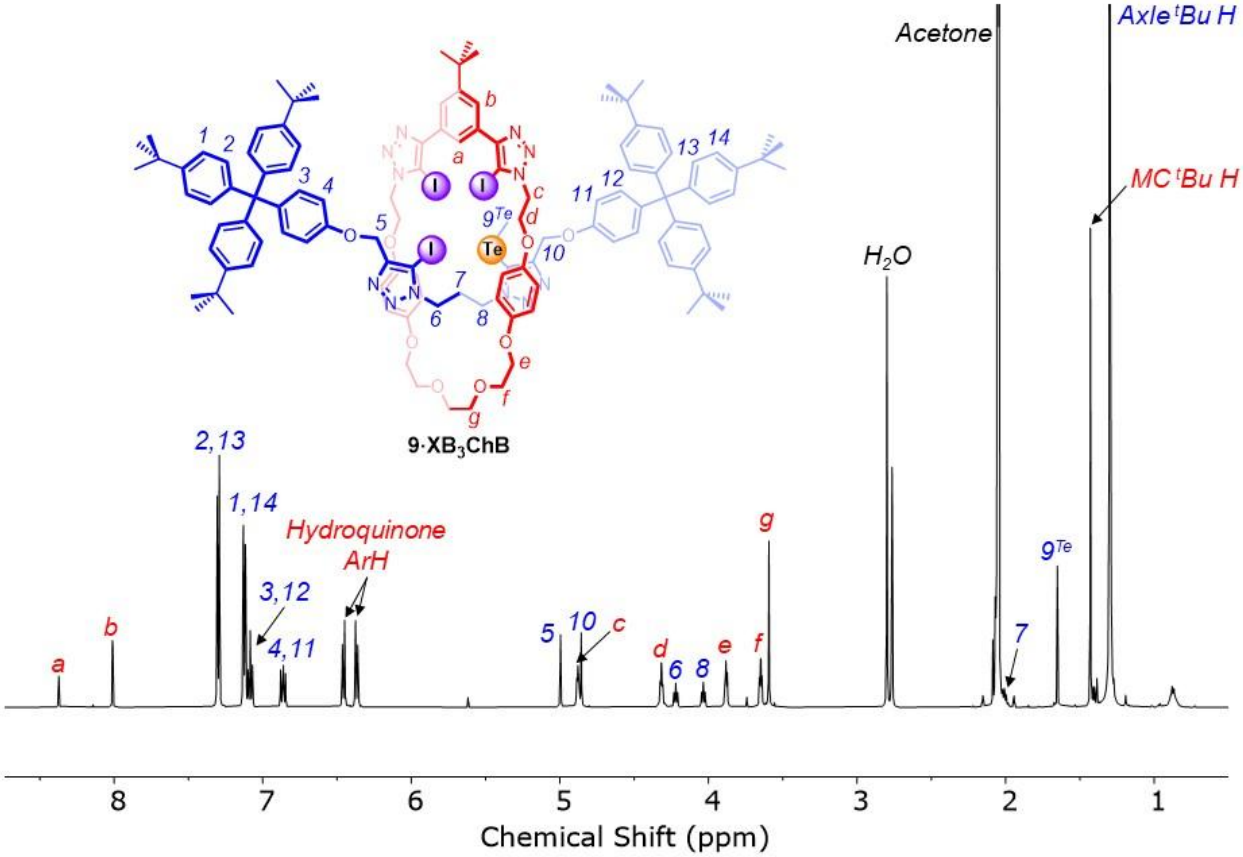

Fig. 49 | ^{1}H NMR spectrum of **9·XB$_3$ChB** (600 MHz, 298 K, acetone-d_6).

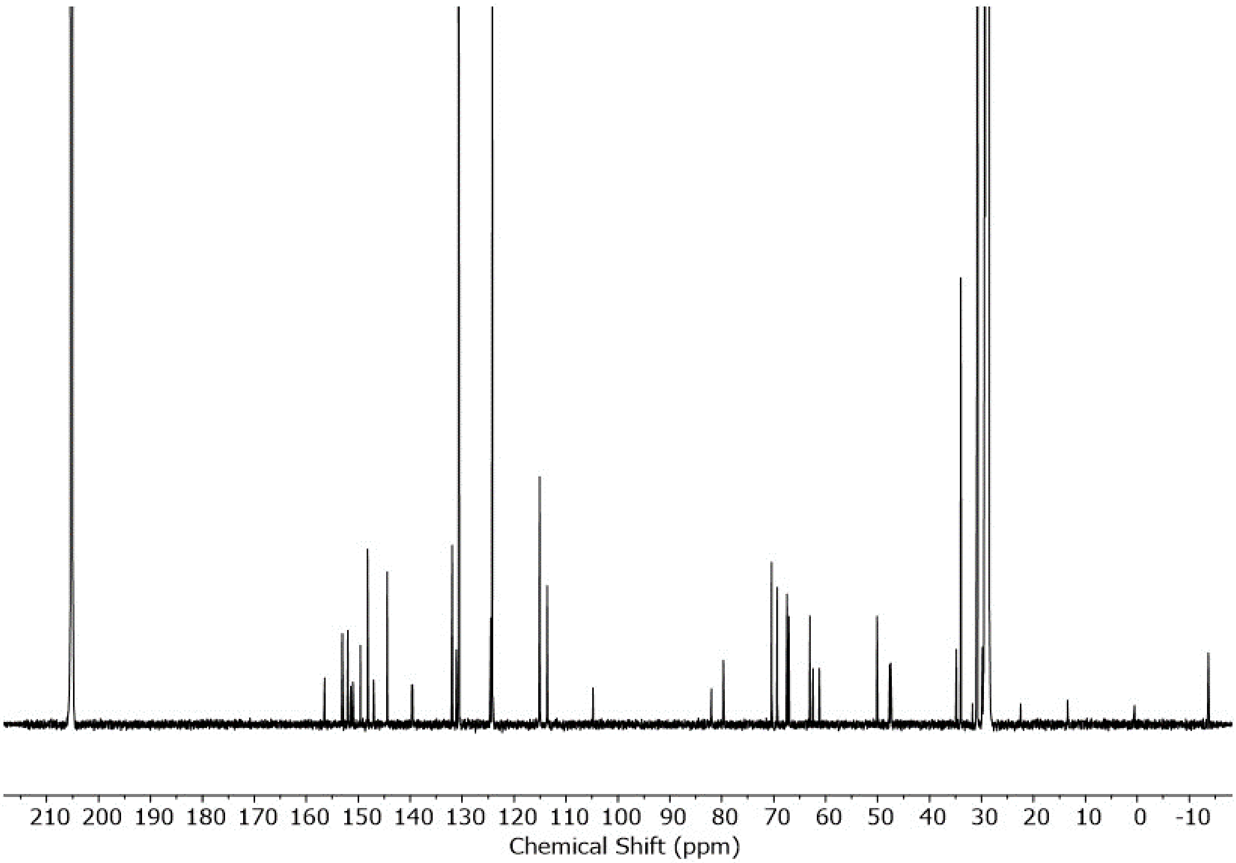

Fig. 50 | ^{13}C NMR spectrum of **9·XB$_3$ChB** (151 MHz, 298 K, acetone-d_6).

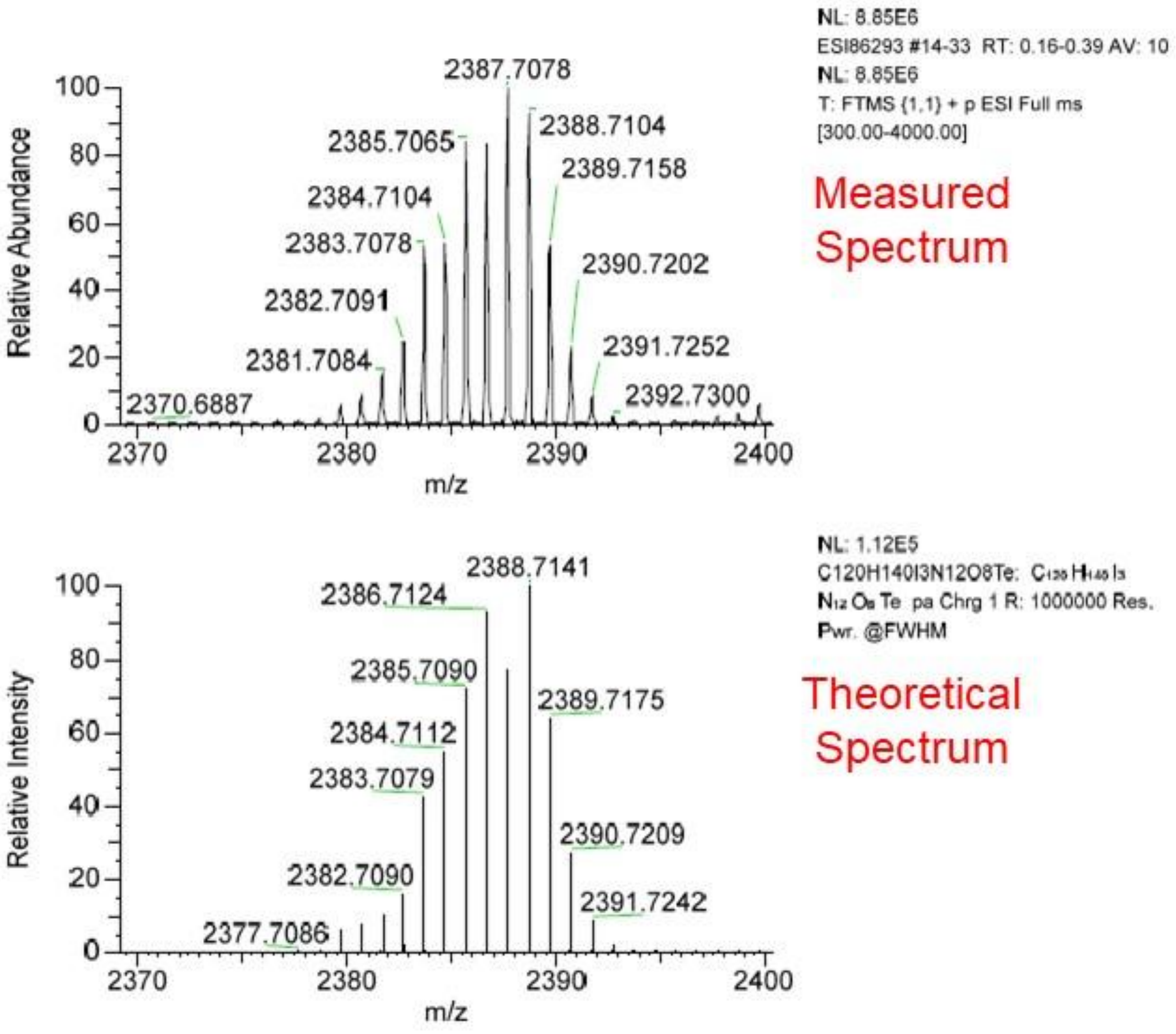

Fig. 51 | Measured (top) and theoretical (bottom) high-resolution ESI mass spectrum of **9·XB$_3$ChB**.

Tetradentate Mixed XB/ChB [2]Rotaxane ($9\cdot ChB_3XB$)

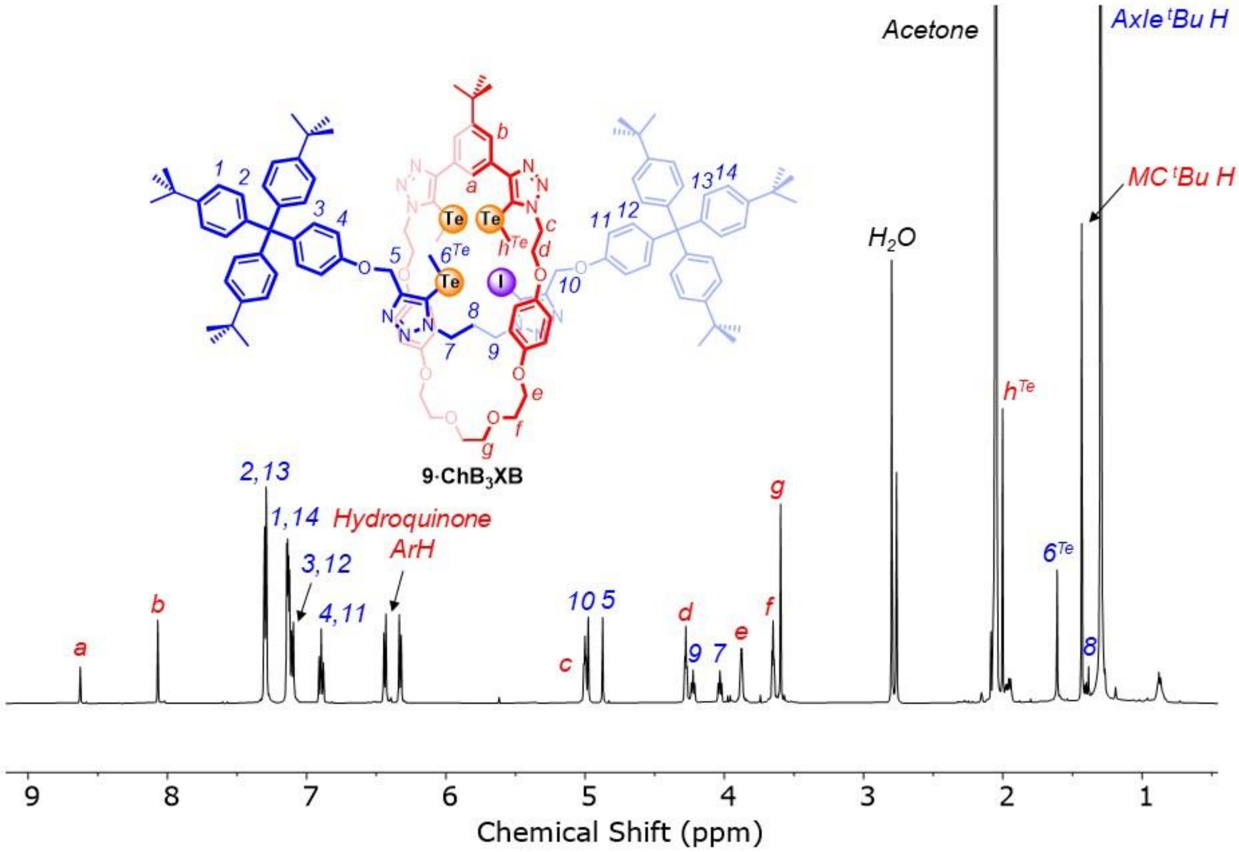

Fig. 52 | ^{1}H NMR spectrum of **$9\cdot ChB_3XB$** (600 MHz, 298 K, acetone-d_6).

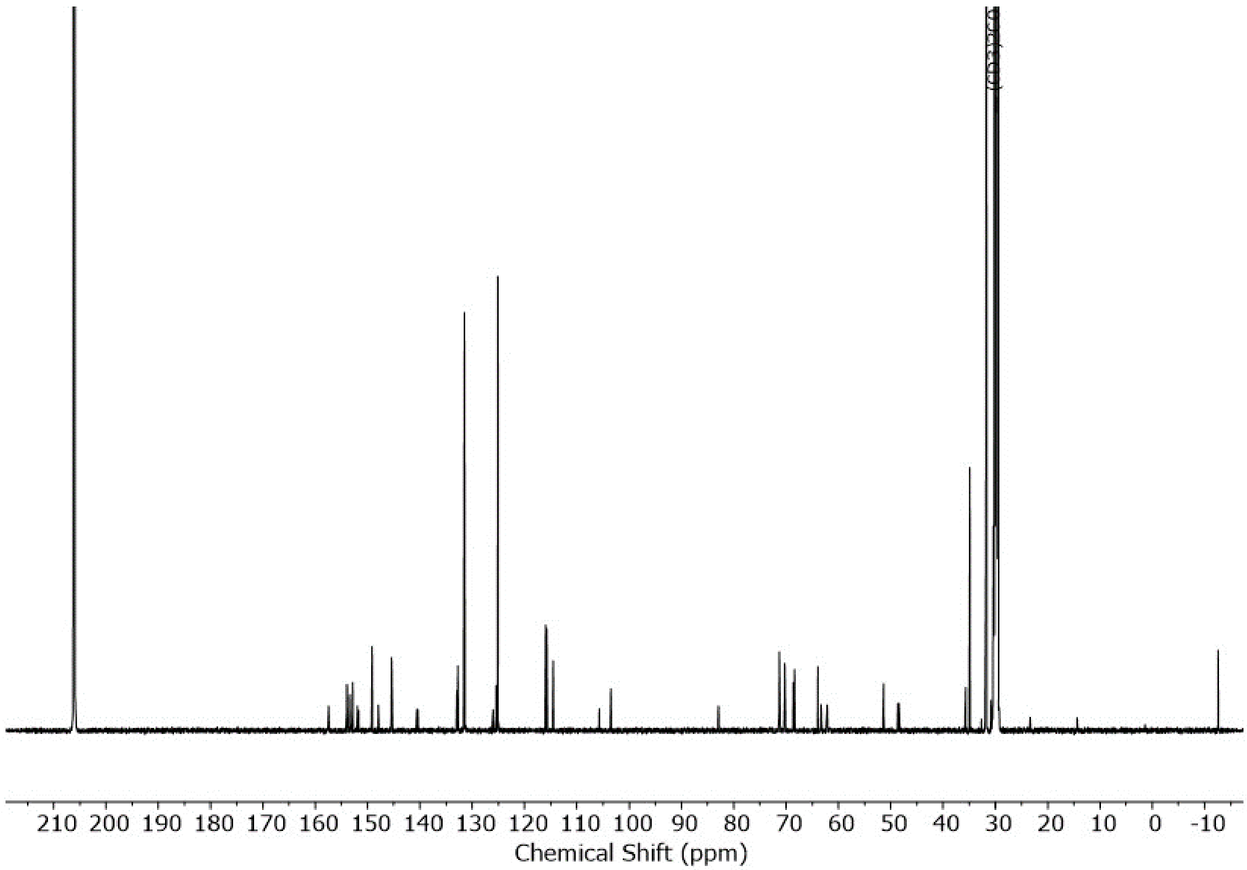

Fig. 53 | ^{13}C NMR spectrum of **9·ChB$_3$XB** (151 MHz, 298 K, acetone-d_6).

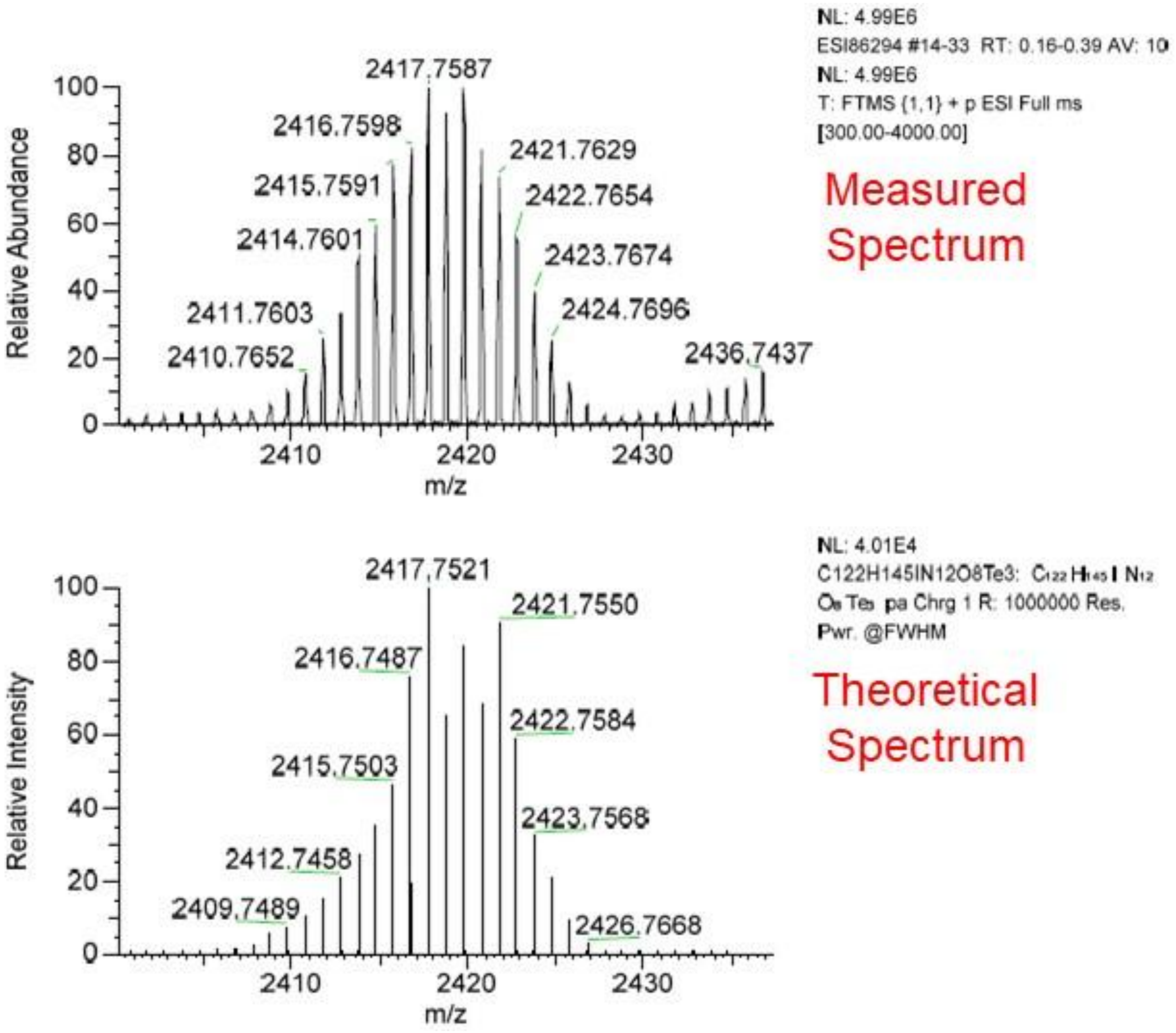

Fig. 54 | Measured (top) and theoretical (bottom) high-resolution ESI mass spectrum of **9·ChB$_3$XB**.

S3 ^{1}H NMR Anion Binding Studies

General Procedures

^{1}H NMR titration experiments were performed on a Bruker AVIII 500 MHz spectrometer. In a typical experiment, a solution of the appropriate TBA salt was added to a solution of the receptor molecule at 298 K in acetone-d_6 or D_2O/acetone-d_6 (2% v/v). Both TBA salt and receptor were dissolved in the same solvent. TBA^+ was chosen as the counter-cation due to its non-coordinating nature. A 50/25 mM solution of the salt was added to 500 μL of a 1.0/0.5 mM solution of receptor, where 1.0 equivalent of salt added corresponds to 10.0 μL of the salt solution. 17 data points corresponding to 0.0, 0.2, 0.4, 0.6, 0.8, 1.0, 1.2, 1.4, 1.6, 1.8, 2.0, 2.5, 3.0, 4.0, 5.0, 7.0 and 10.0 equivalents of added guest anion were obtained. The binding of anions with all receptors were found to be fast on the NMR timescale. All of the titrations were performed in triplicate, with the exception of those which demonstrated no measurable binding, and those which possessed K_a values larger than can be accurately determined by NMR titration which were performed in duplicate.

For anion titrations, only one representative spectrum is shown for each titration in Fig. 55–75, followed by the binding isotherms obtained by monitoring the changes in the chemical shift of a) macrocycle telluromethyl proton h^{Te} for **8·ChB$_3$** and **9·ChB$_4$** (Fig. 76 and 79), b) macrocycle internal benzene proton *a* for **8·XB$_2$ChB$^{Me/Ph/pCF3}$**, **9·XB$_2$ChB$_2$** and **9·XB$_3$ChB** (Fig. 77, 78, 81 and 82), or c) axle methylene proton *10* for **9·ChB$_3$XB** (Fig. 80) as a function of anion concentration. The values of the observed chemical shifts and guest concentration at each titration data point were input into the BindFit[11–13] software, with initial estimates of K_a and limiting chemical shifts. These parameters were refined using non-linear least-squares analyses to obtain the best fit between the empirical and calculated chemical shifts based on a 1:1 host-guest binding model. The input parameters were iteratively varied until convergence of the best fit values of K_a were obtained. K_a values of all of the repeats of the titrations were within 1-2% of those values summarised in Research Article Table 1, and the errors quoted were derived from the fitting error of the representative titration experiment, with the value of which was the median of the triplicate.

^{1}H NMR Anion Titration Spectra

Tridentate All-ChB [2]Rotaxane (8·ChB$_3$)

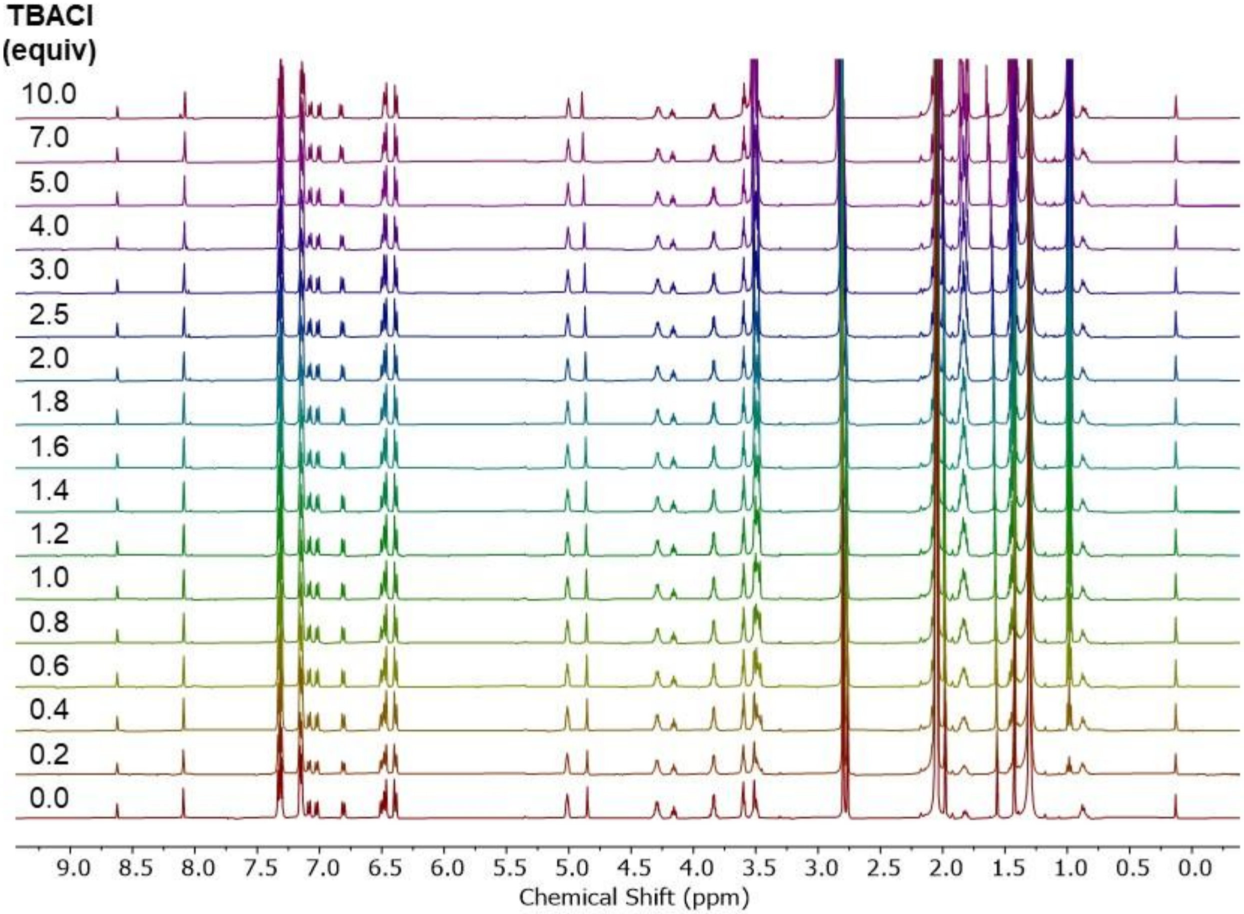

Fig. 55 | ^{1}H NMR titration spectra of **8·ChB$_3$** upon addition of 10 equivalents of TBACl ([**8·ChB$_3$**] = 1.0 mM, 500 MHz, 298 K, acetone-*d*$_6$).

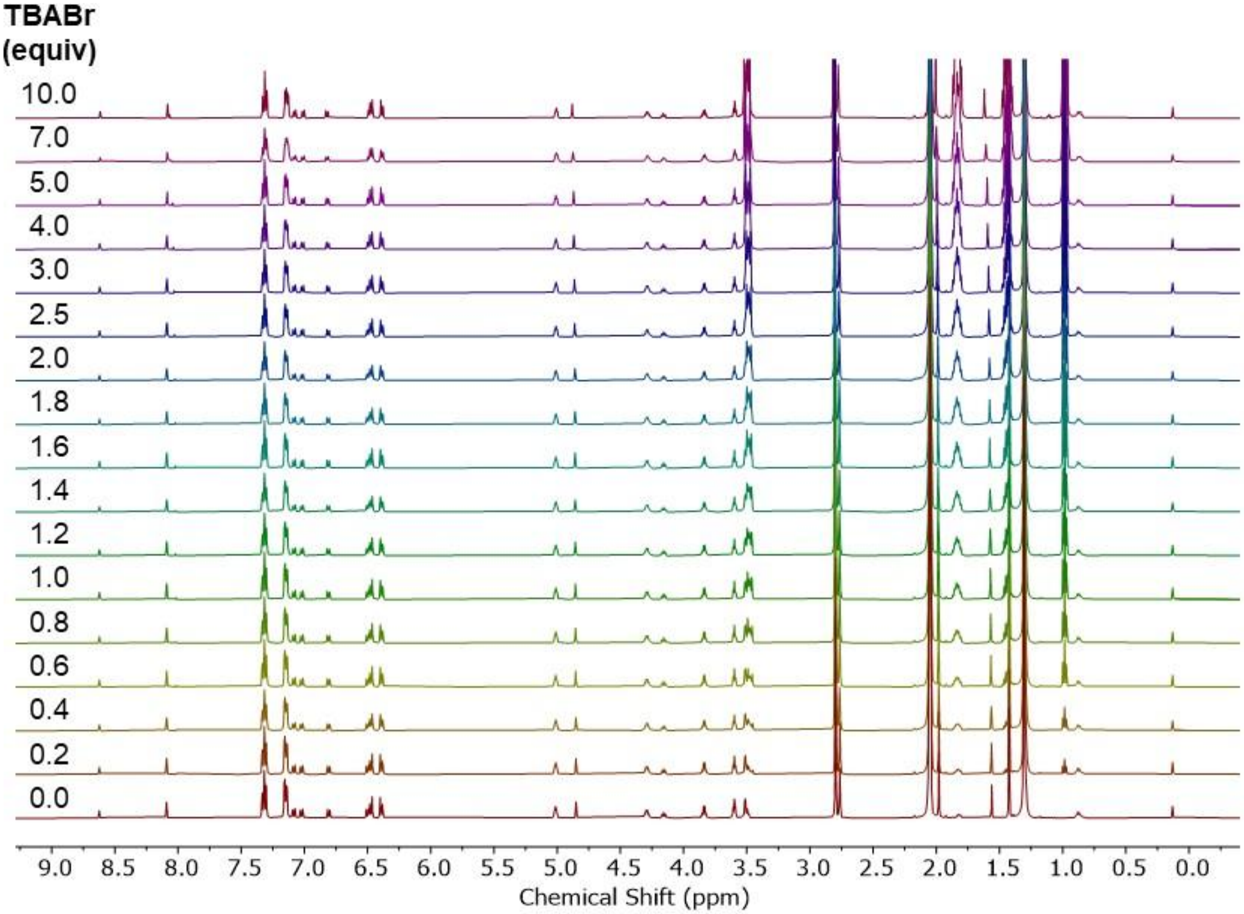

Fig. 56 | ^{1}H NMR titration spectra of **8·ChB₃** upon addition of 10 equivalents of TBABr ([**8·ChB₃**] = 1.0 mM, 500 MHz, 298 K, acetone-d_6).

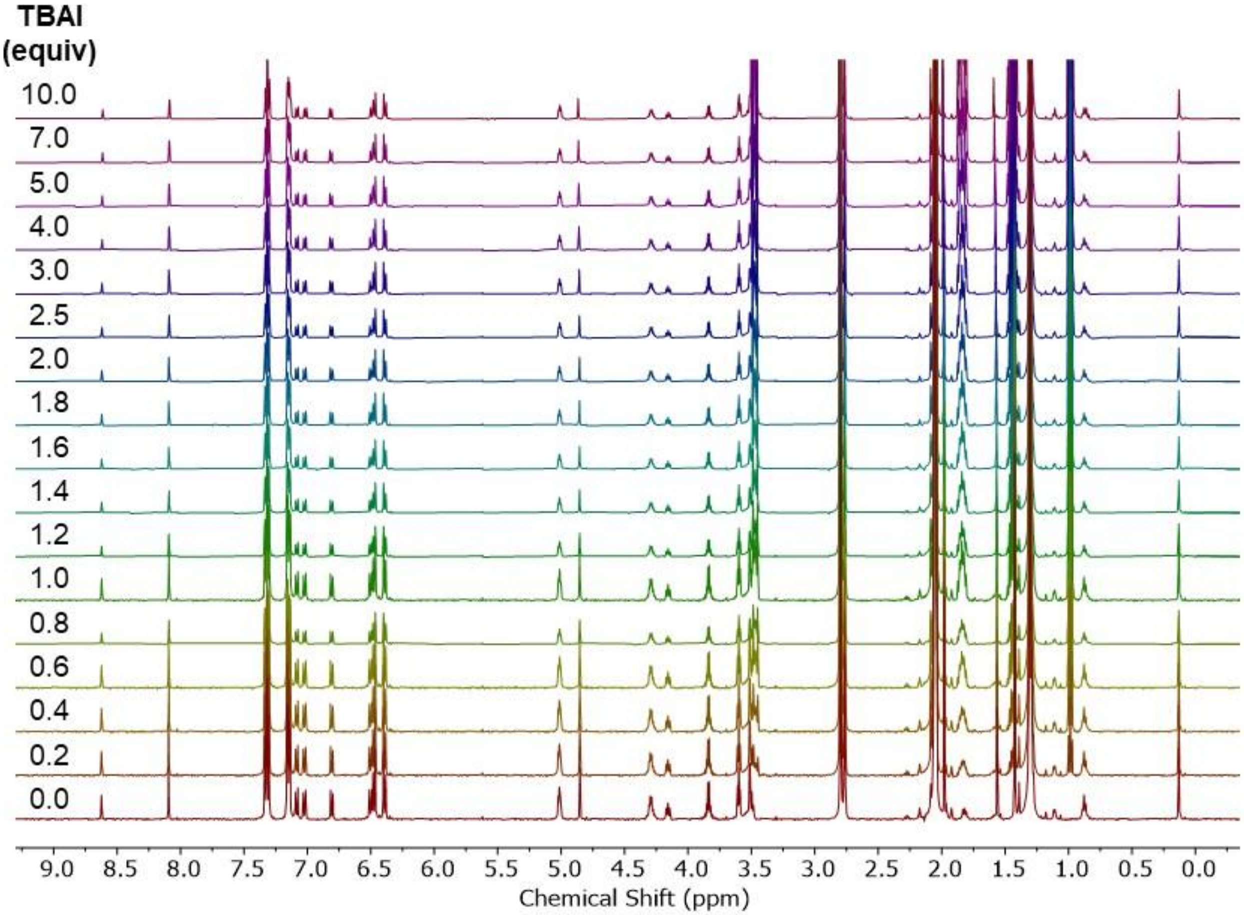

Fig. 57 | ^{1}H NMR titration spectra of **8·ChB$_3$** upon addition of 10 equivalents of TBAI ([**8·ChB$_3$**] = 1.0 mM, 500 MHz, 298 K, acetone-d_6).

Tridentate Mixed XB/ChB [2]Rotaxane (8·XB$_2$ChB^{Me})

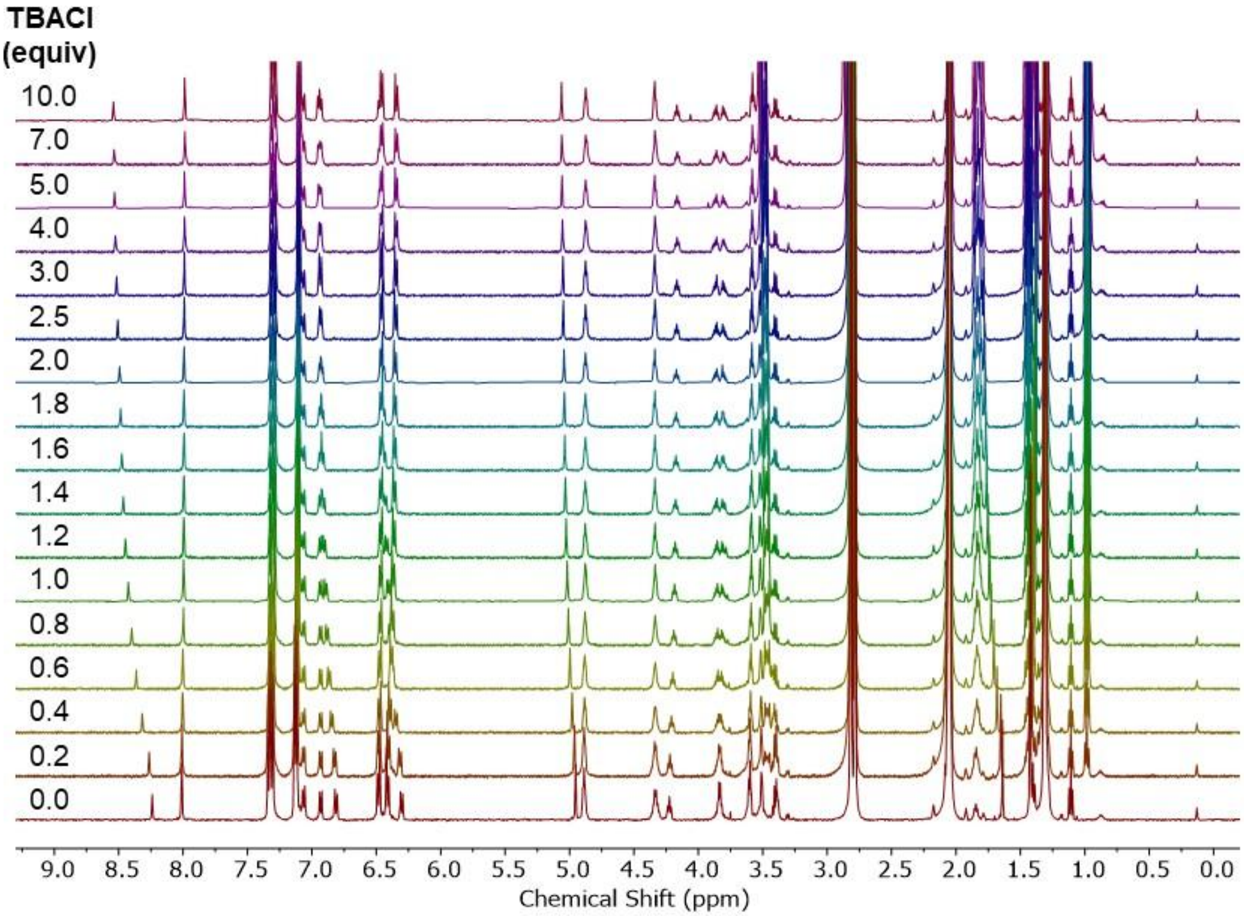

Fig. 58 | ^{1}H NMR titration spectra of **8·XB$_2$ChB^{Me}** upon addition of 10 equivalents of TBACl ([**8·XB$_2$ChB^{Me}**] = 1.0 mM, 500 MHz, 298 K, acetone-d_6).

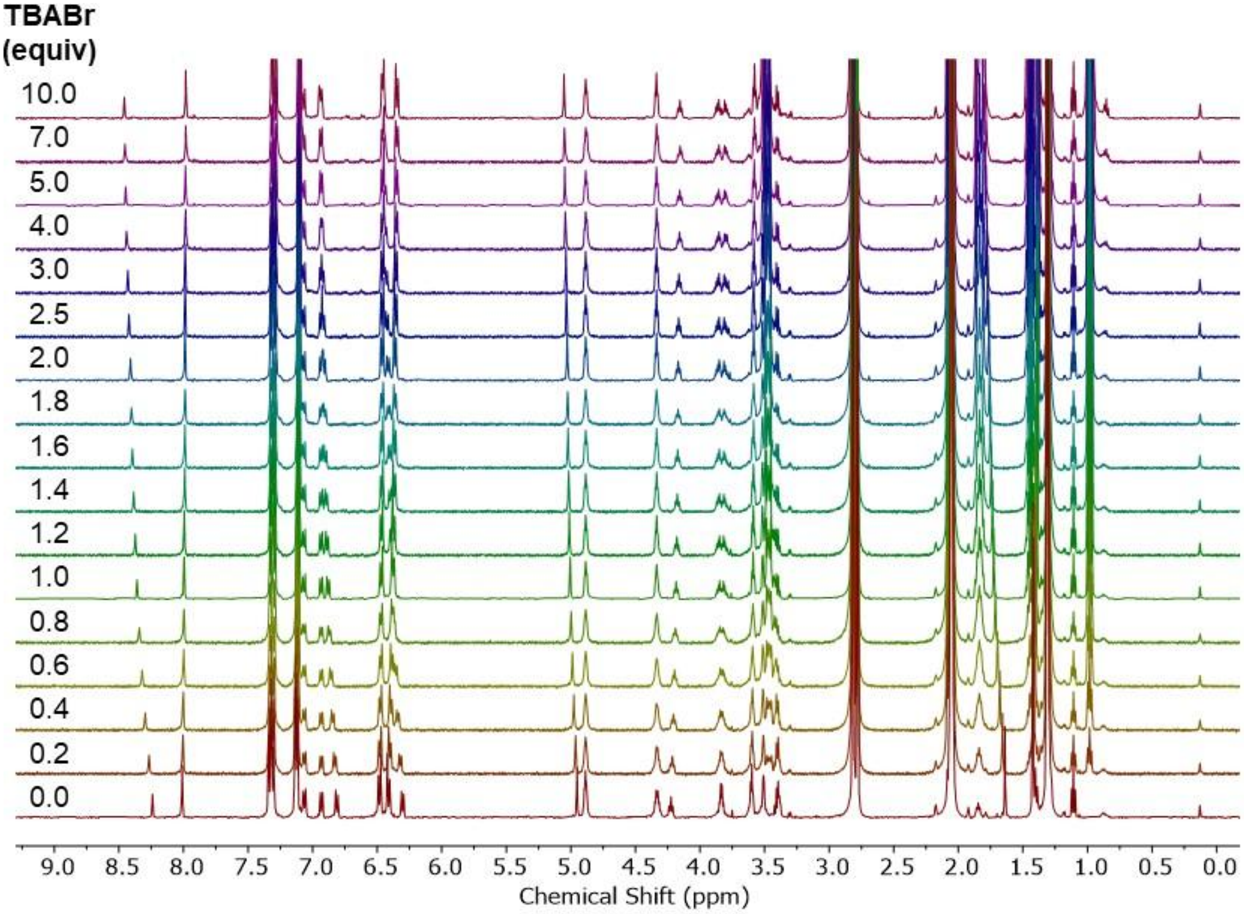

Fig. 59 | ^{1}H NMR titration spectra of **8·XB$_2$ChB^{Me}** upon addition of 10 equivalents of TBABr ([**8·XB$_2$ChB^{Me}**] = 1.0 mM, 500 MHz, 298 K, acetone-d_6).

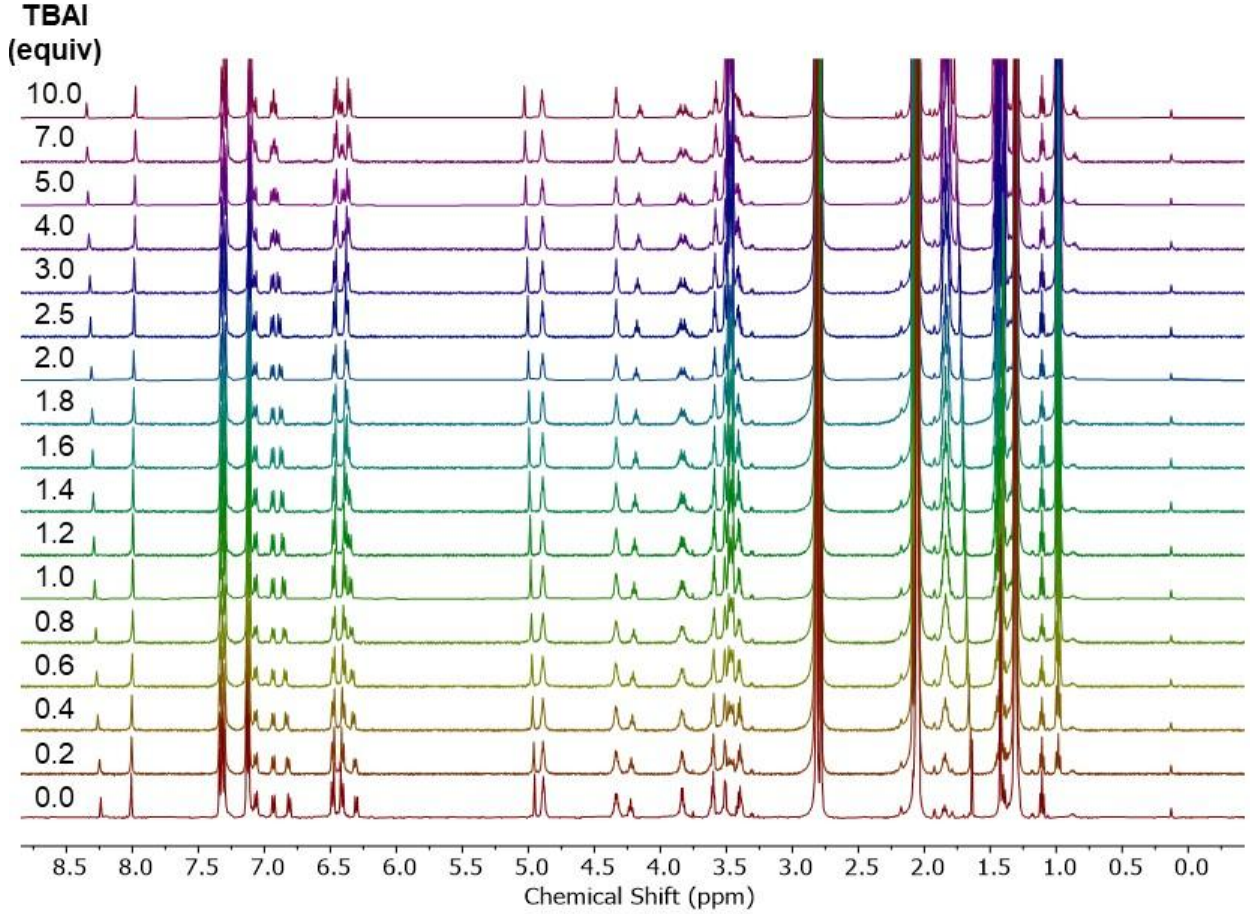

Fig. 60 | ^{1}H NMR titration spectra of **8·XB$_2$ChB^{Me}** upon addition of 10 equivalents of TBAI ([**8·XB$_2$ChB^{Me}**] = 1.0 mM, 500 MHz, 298 K, acetone-*d*$_6$).

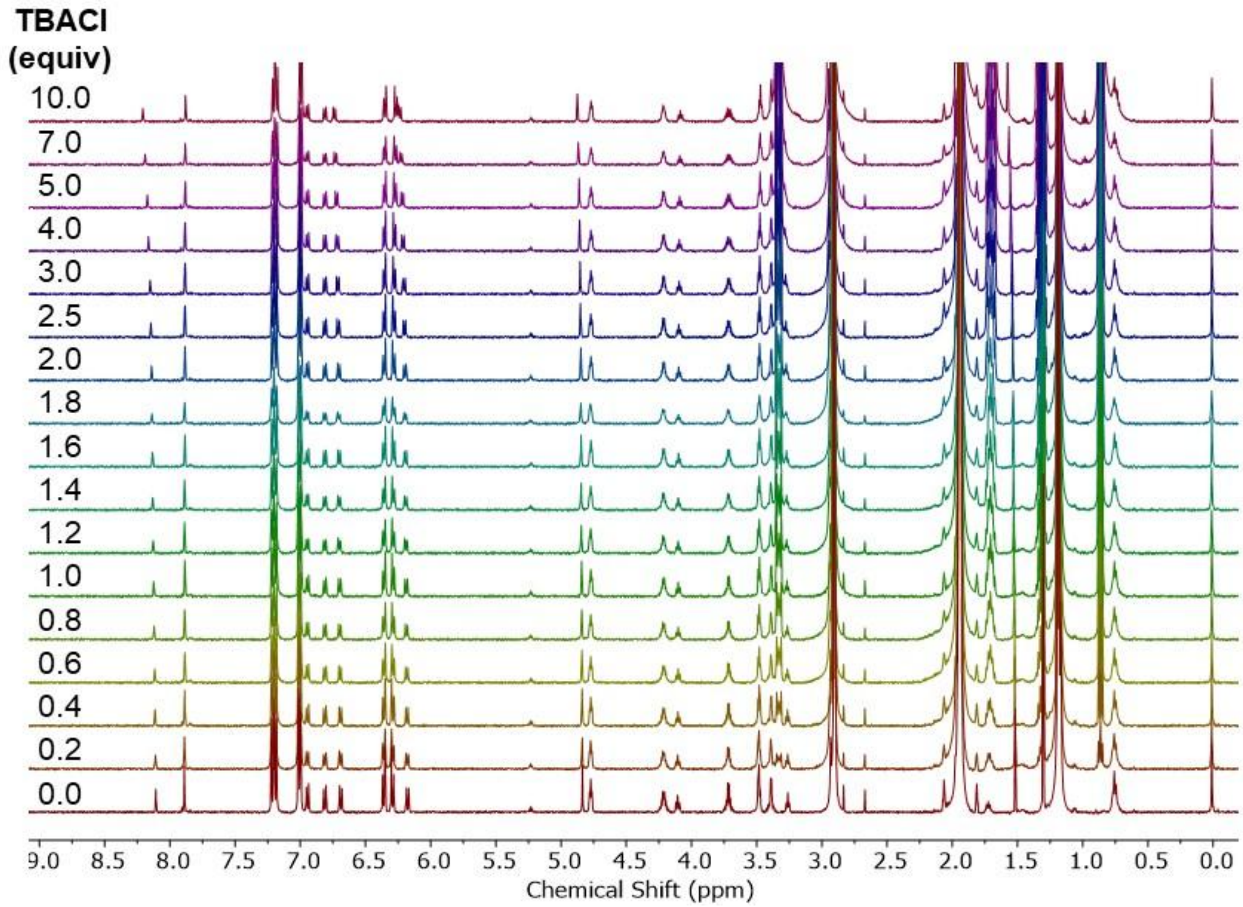

Fig. 61 | ^{1}H NMR titration spectra of **8·XB$_2$ChB^{Me}** upon addition of 10 equivalents of TBACl ([**8·XB$_2$ChB^{Me}**] = 1.0 mM, 500 MHz, 298 K, 2% D_2O in acetone-d_6).

Tridentate Mixed XB/ChB [2]Rotaxane (8·XB_2ChB^{Ph})

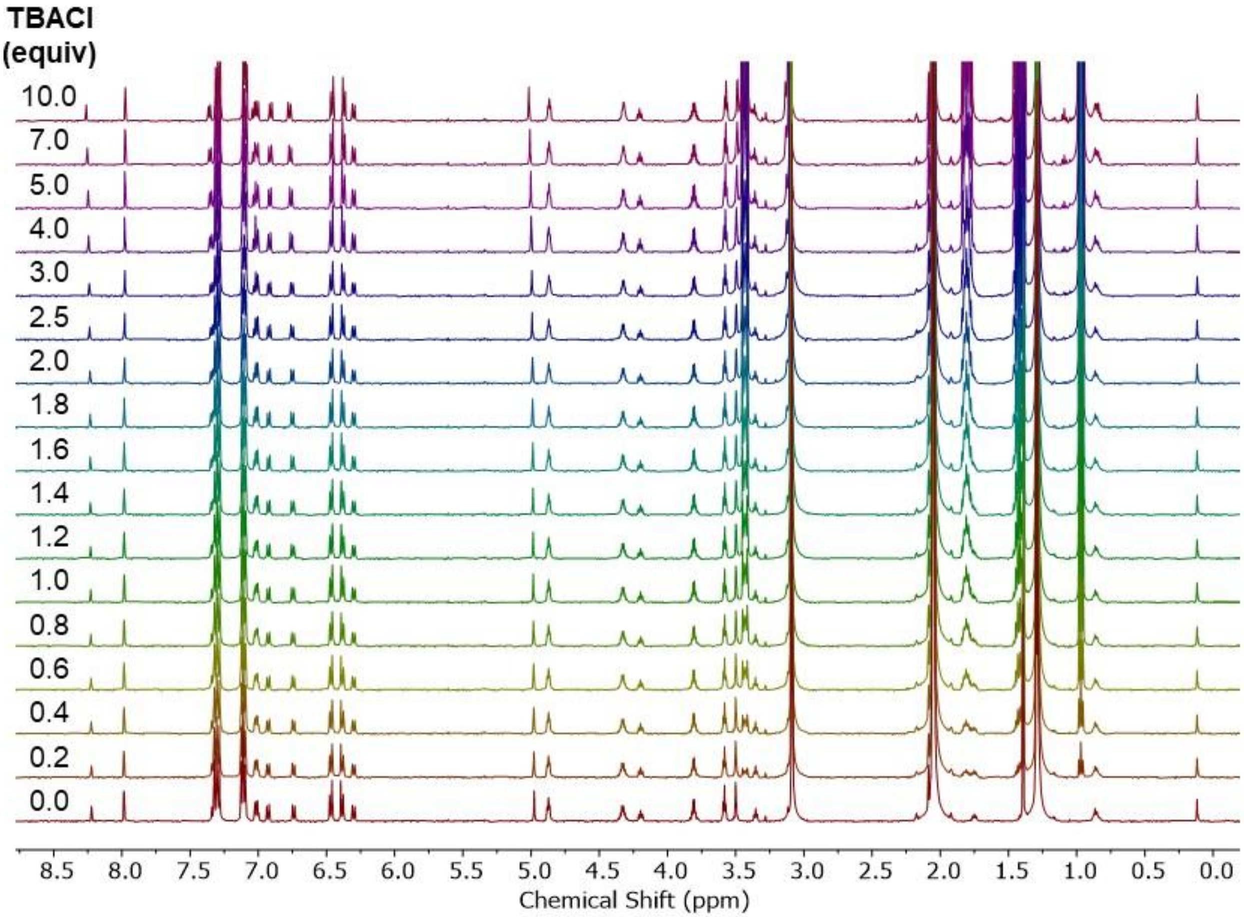

Fig. 62 | ^{1}H NMR titration spectra of **8·XB_2ChB^{Ph}** upon addition of 10 equivalents of TBACl ([**8·XB_2ChB^{Ph}**] = 1.0 mM, 500 MHz, 298 K, 2% D_2O in acetone-d_6).

Tridentate Mixed XB/ChB [2]Rotaxane (8·XB_2ChB^{pCF3})

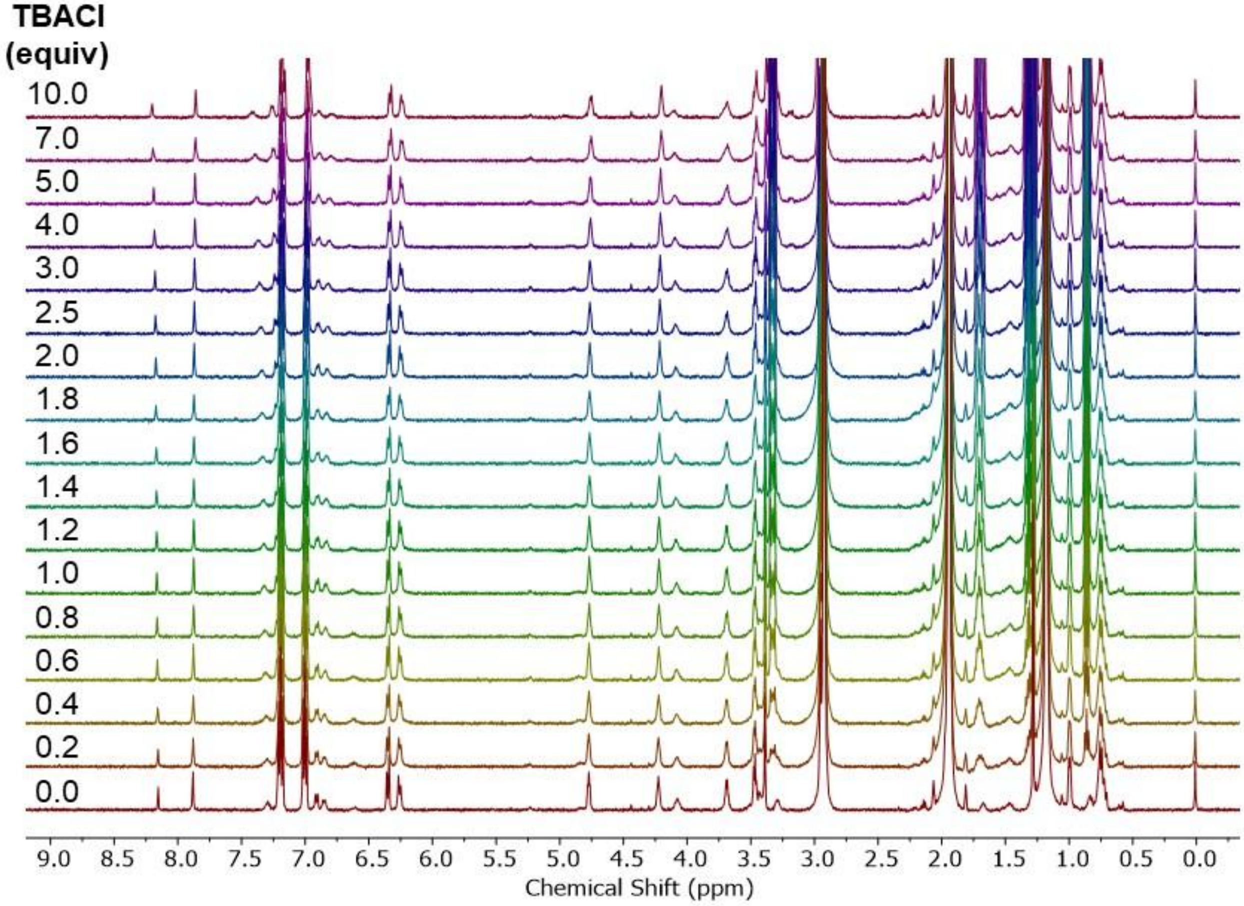

Fig. 63 | ^{1}H NMR titration spectra of **8·XB_2ChB^{pCF3}** upon addition of 10 equivalents of TBACl ([**8·XB_2ChB^{pCF3}**] = 1.0 mM, 500 MHz, 298 K, 2% D_2O in acetone-d_6).

Tetradentate All-ChB [2]Rotaxane (9·ChB$_4$)

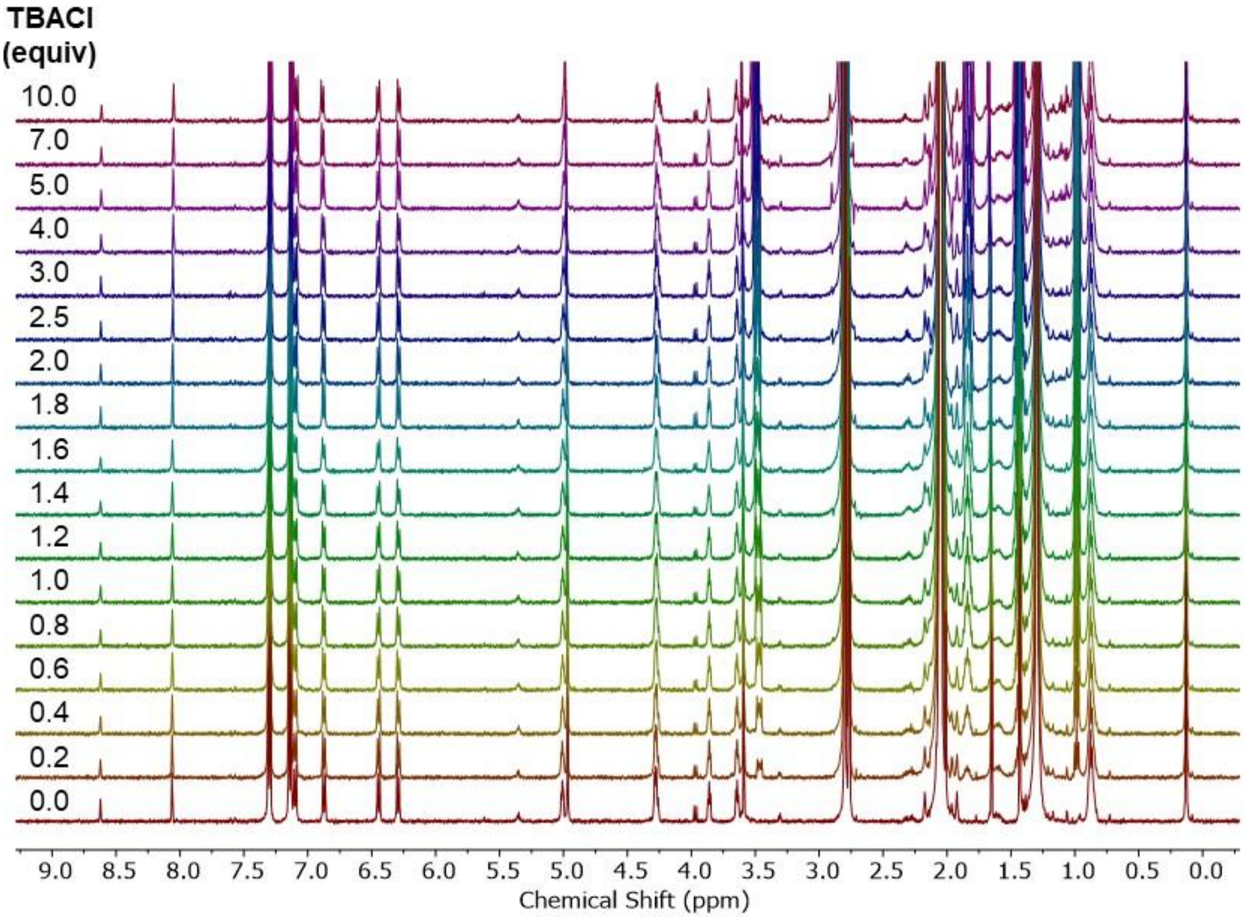

Fig. 64 | ^{1}H NMR titration spectra of **9·ChB$_4$** upon addition of 10 equivalents of TBACl ([**9·ChB$_4$**] = 0.5 mM, 500 MHz, 298 K, acetone-d_6).

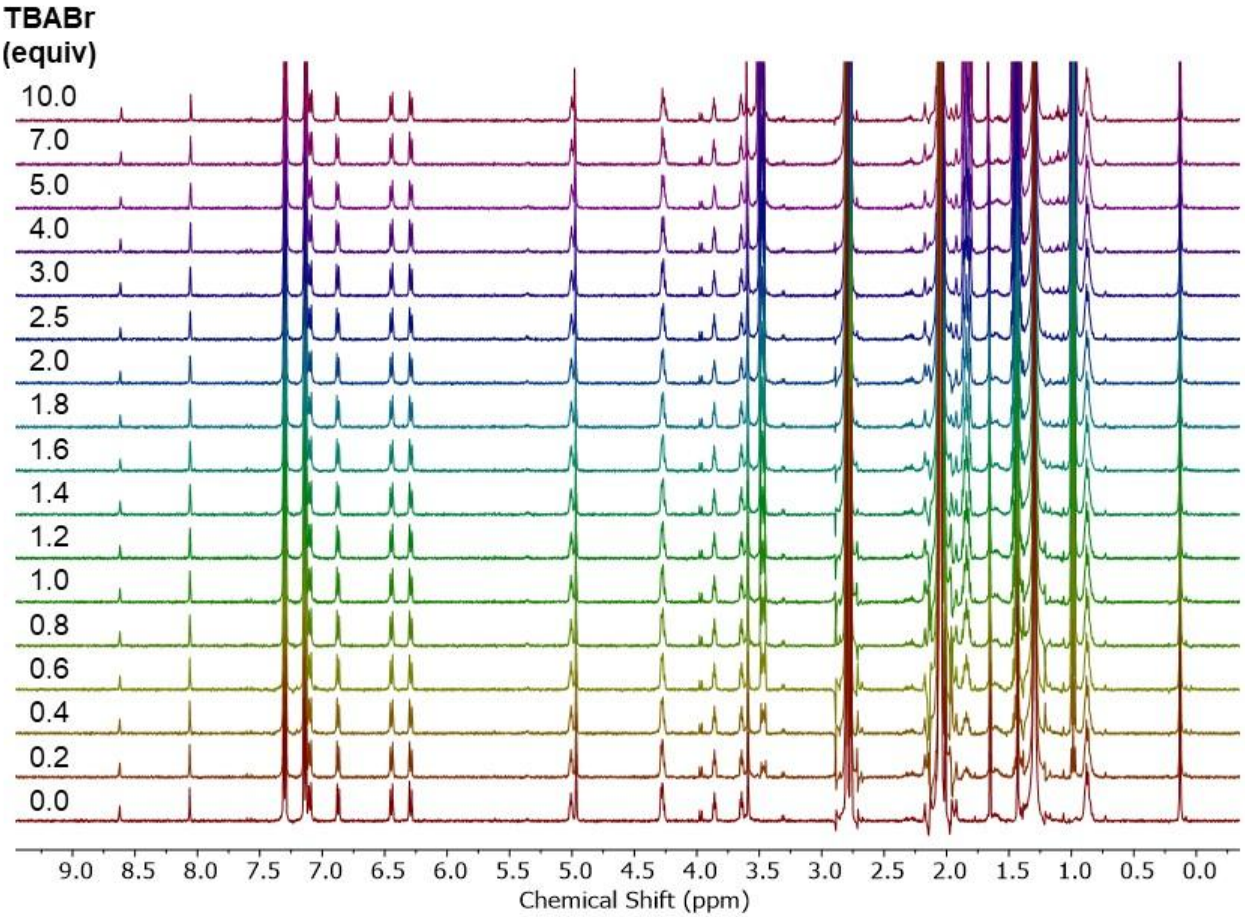

Fig. 65 | ^{1}H NMR titration spectra of **9·ChB$_4$** upon addition of 10 equivalents of TBABr ([**9·ChB$_4$**] = 0.5 mM, 500 MHz, 298 K, acetone-d_6).

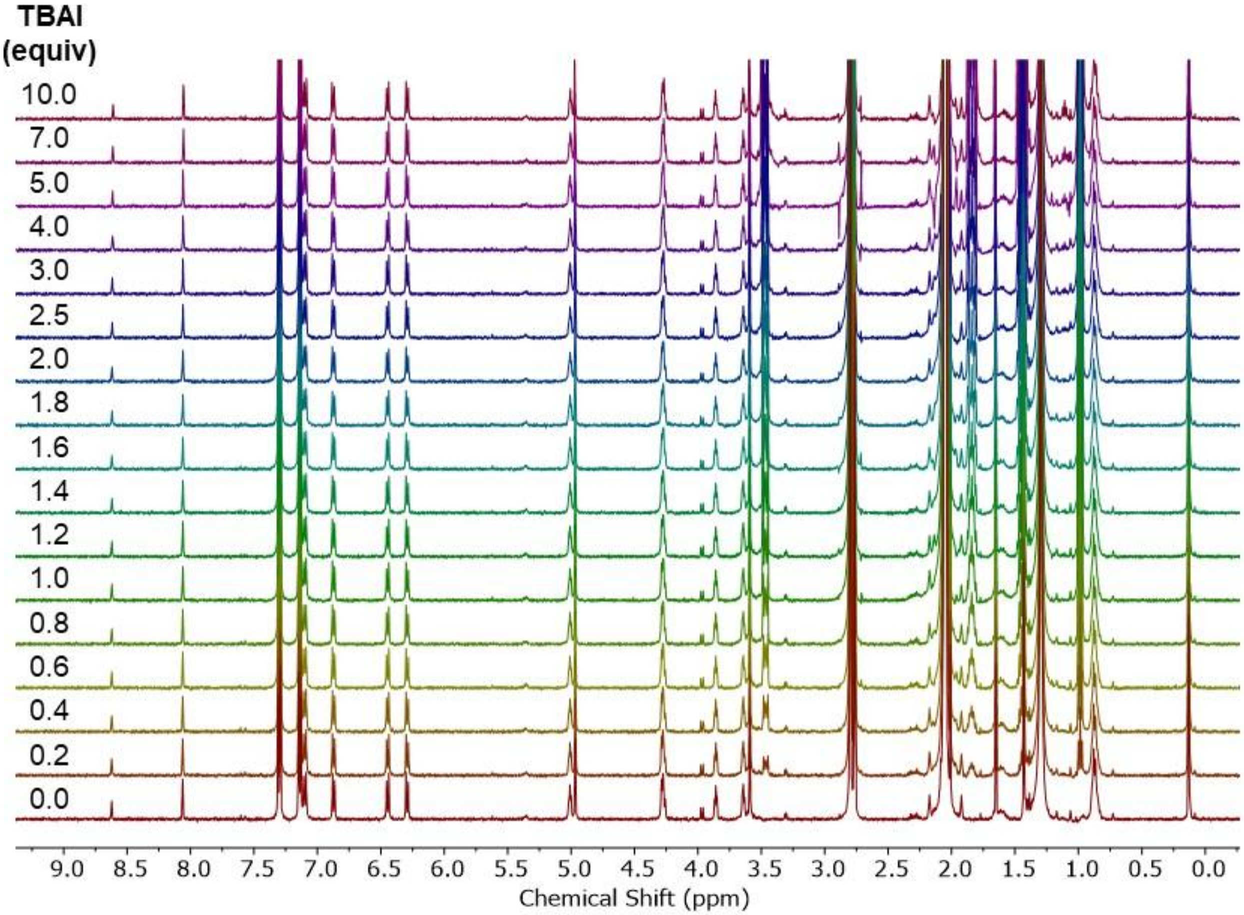

Fig. 66 | ^{1}H NMR titration spectra of **9·ChB$_4$** upon addition of 10 equivalents of TBAI ([**9·ChB$_4$**] = 0.5 mM, 500 MHz, 298 K, acetone-d_6).

Tetradentate Mixed XB/ChB [2]Rotaxane (9·ChB$_3$XB)

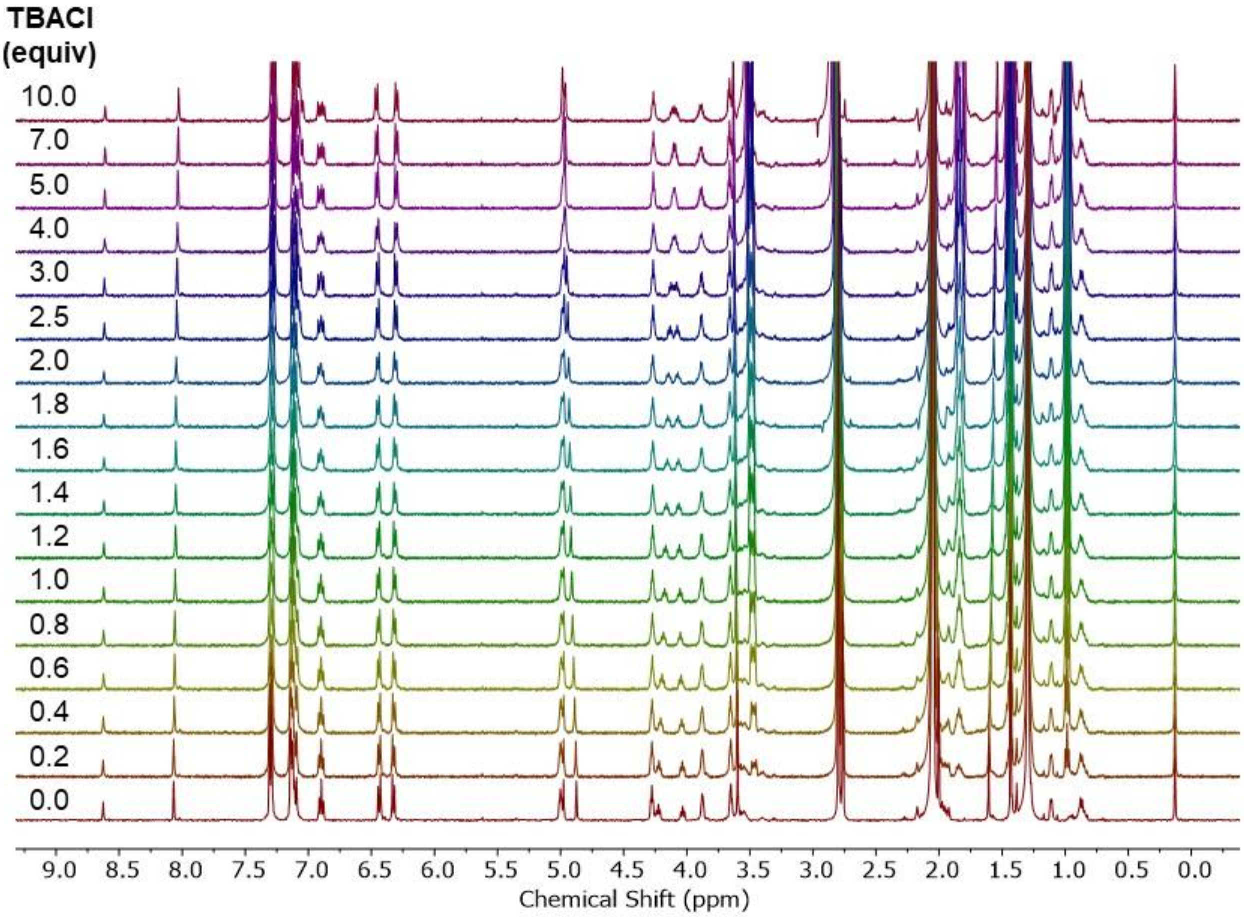

Fig. 67 | ^{1}H NMR titration spectra of **9·ChB$_3$XB** upon addition of 10 equivalents of TBACl ([**9·ChB$_3$XB**] = 1.0 mM, 500 MHz, 298 K, acetone-d_6).

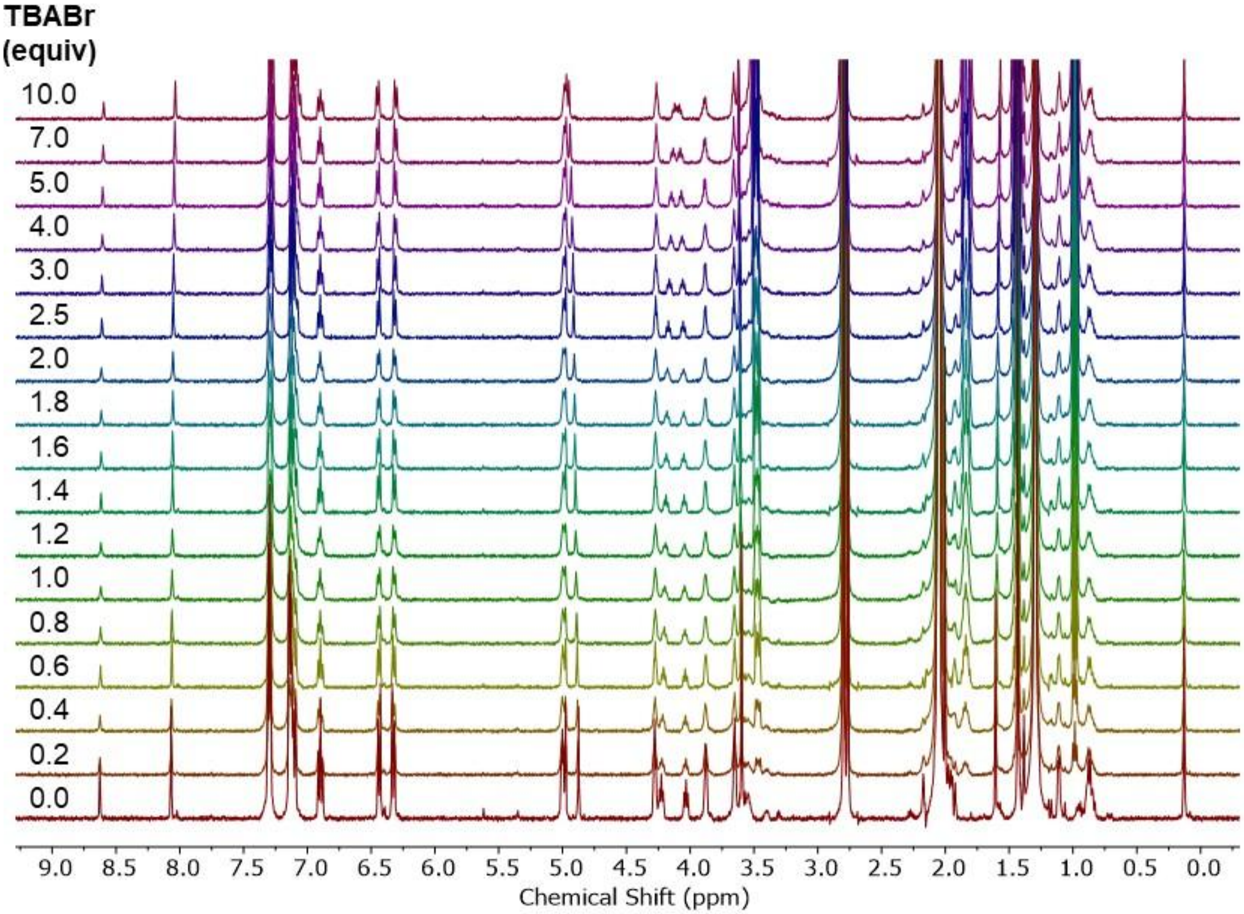

Fig. 68 | ^{1}H NMR titration spectra of **9·ChB$_3$XB** upon addition of 10 equivalents of TBABr ([**9·ChB$_3$XB**] = 1.0 mM, 500 MHz, 298 K, acetone-d_6).

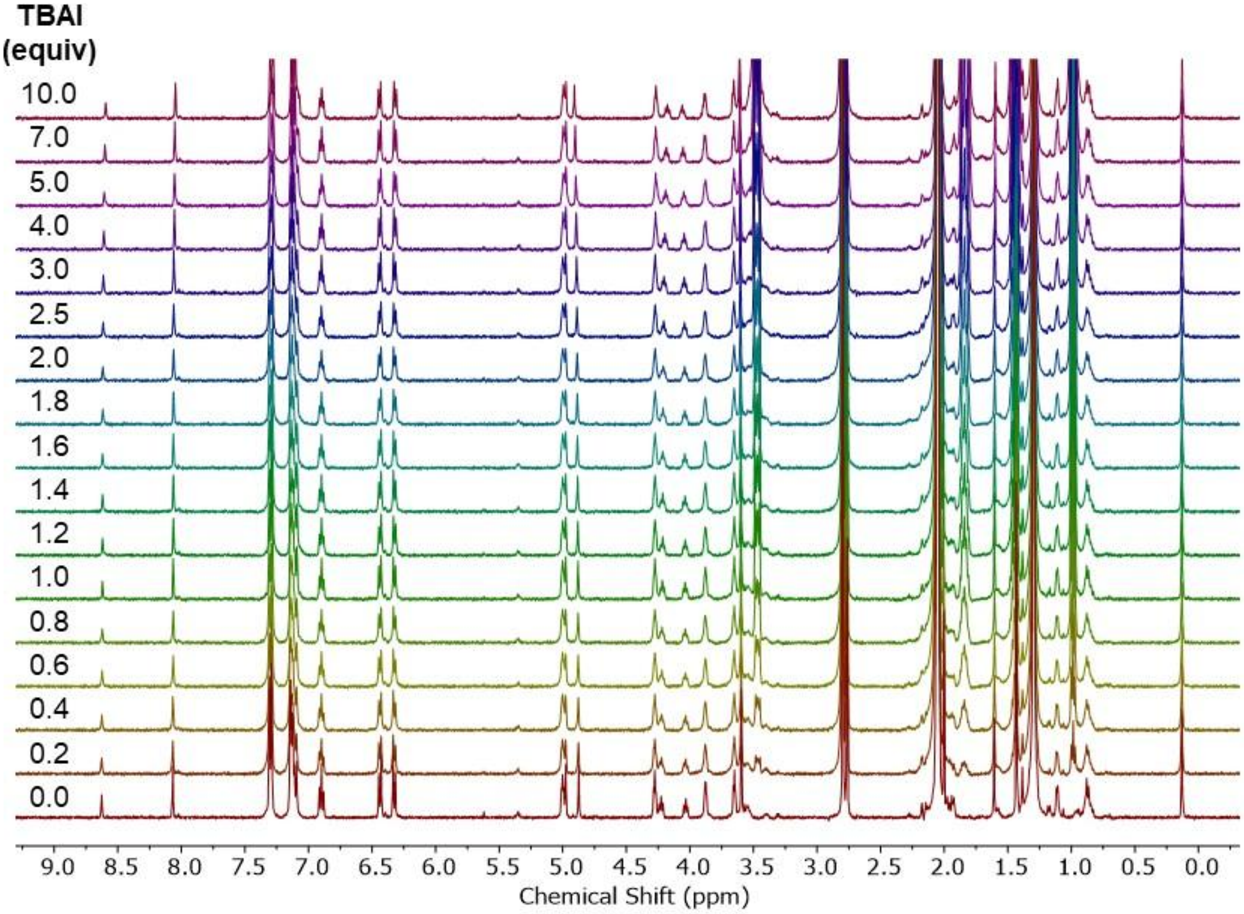

Fig. 69 | ^{1}H NMR titration spectra of **9·ChB$_3$XB** upon addition of 10 equivalents of TBAI ([**9·ChB$_3$XB**] = 1.0 mM, 500 MHz, 298 K, acetone-d_6).

Tetradentate Mixed XB/ChB [2]Rotaxane (9·XB_2ChB_2)

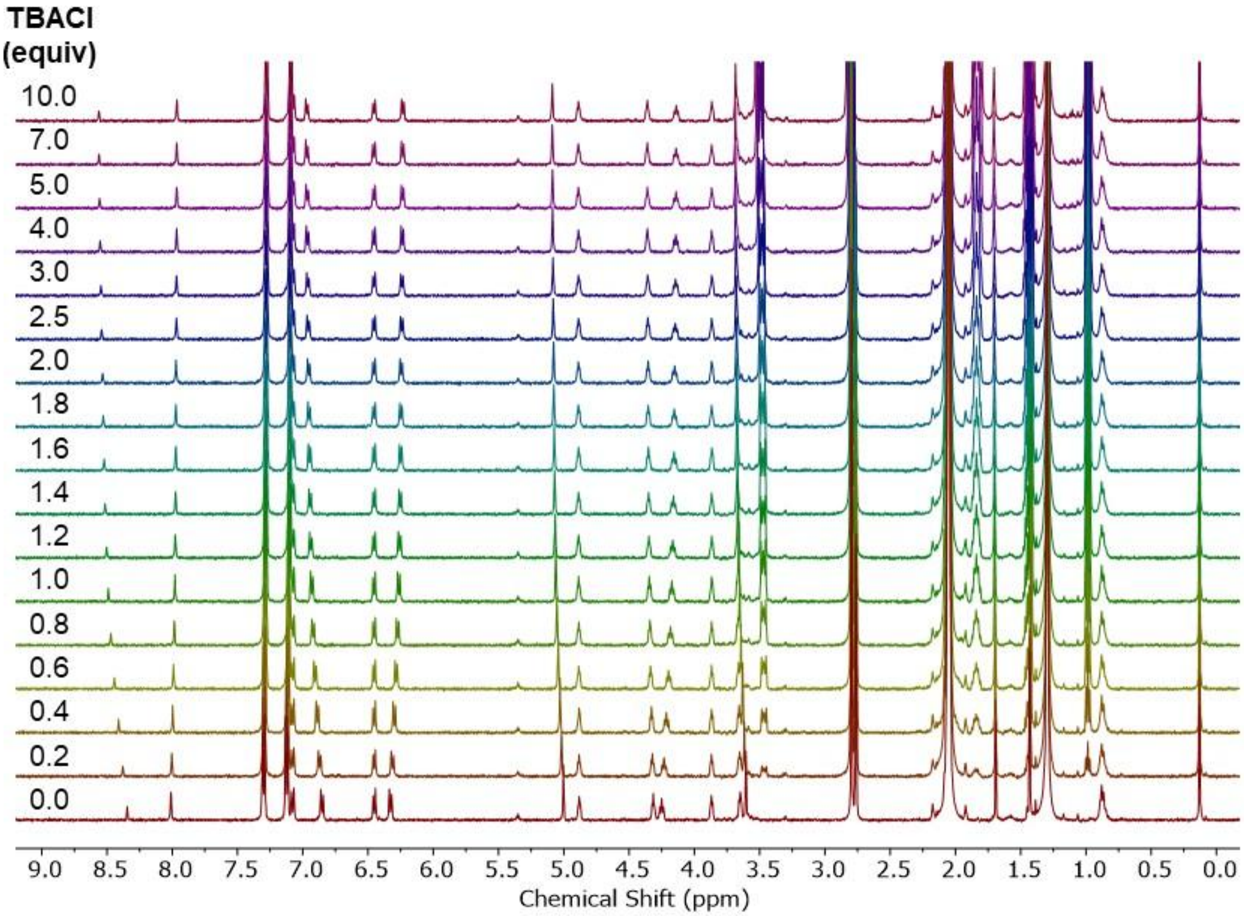

Fig. 70 | ^{1}H NMR titration spectra of **9·XB_2ChB_2** upon addition of 10 equivalents of TBACl ([**9·XB_2ChB_2**] = 0.5 mM, 500 MHz, 298 K, acetone-d_6).

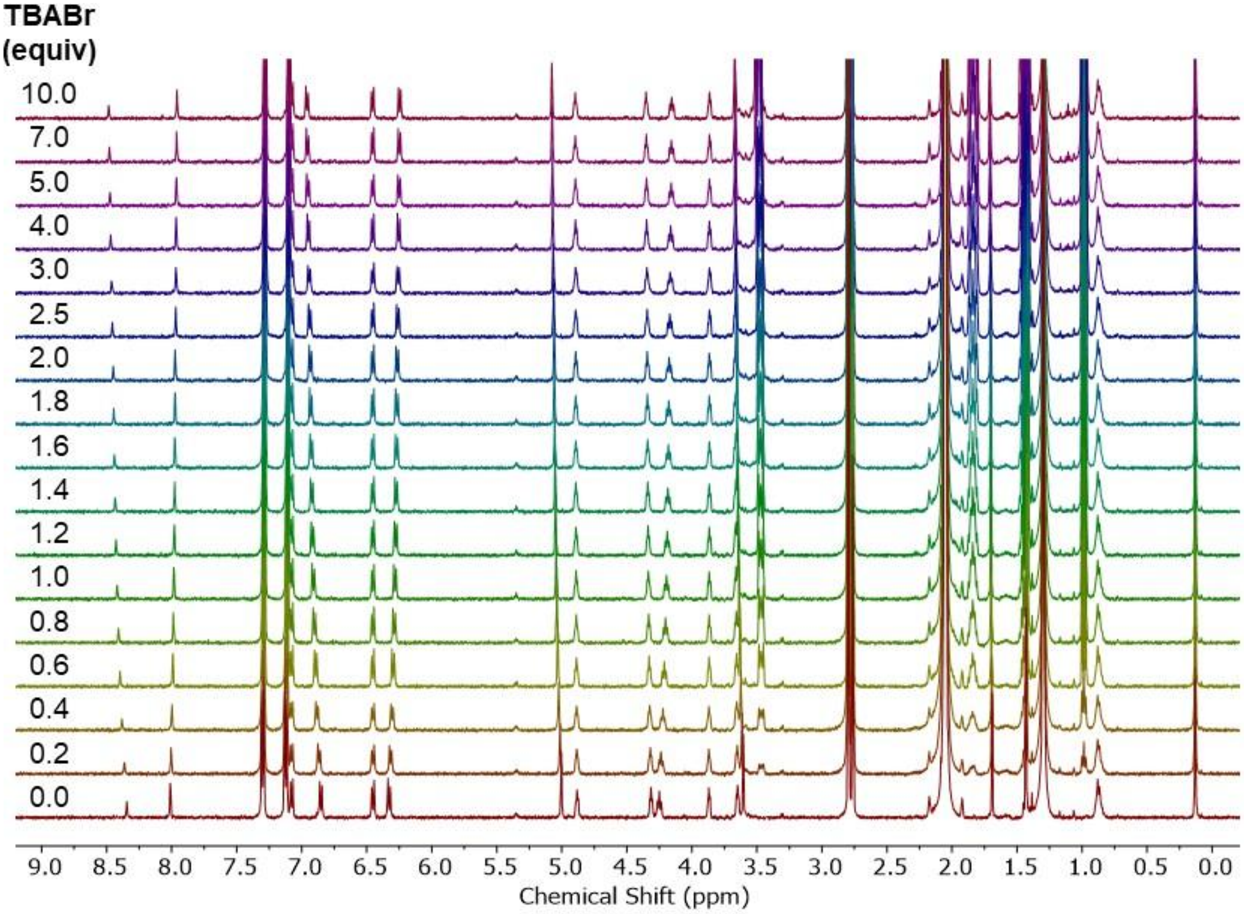

Fig. 71 | ^{1}H NMR titration spectra of **9·XB$_2$ChB$_2$** upon addition of 10 equivalents of TBABr ([**9·XB$_2$ChB$_2$**] = 0.5 mM, 500 MHz, 298 K, acetone-d_6).

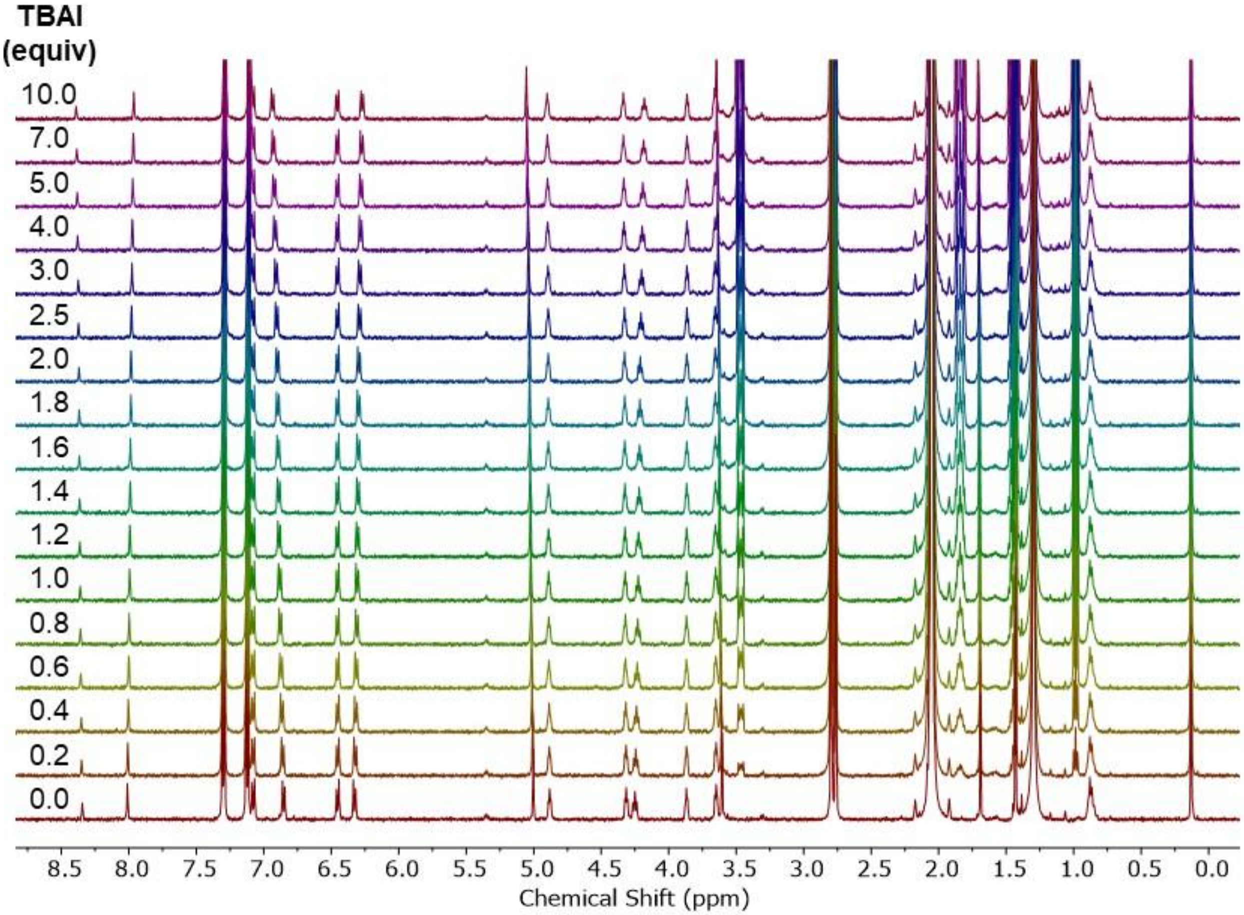

Fig. 72 | ^{1}H NMR titration spectra of **9·XB$_2$ChB$_2$** upon addition of 10 equivalents of TBAI ([**9·XB$_2$ChB$_2$**] = 0.5 mM, 500 MHz, 298 K, acetone-d_6).

Tetradentate Mixed XB/ChB [2]Rotaxane (9·XB$_3$ChB)

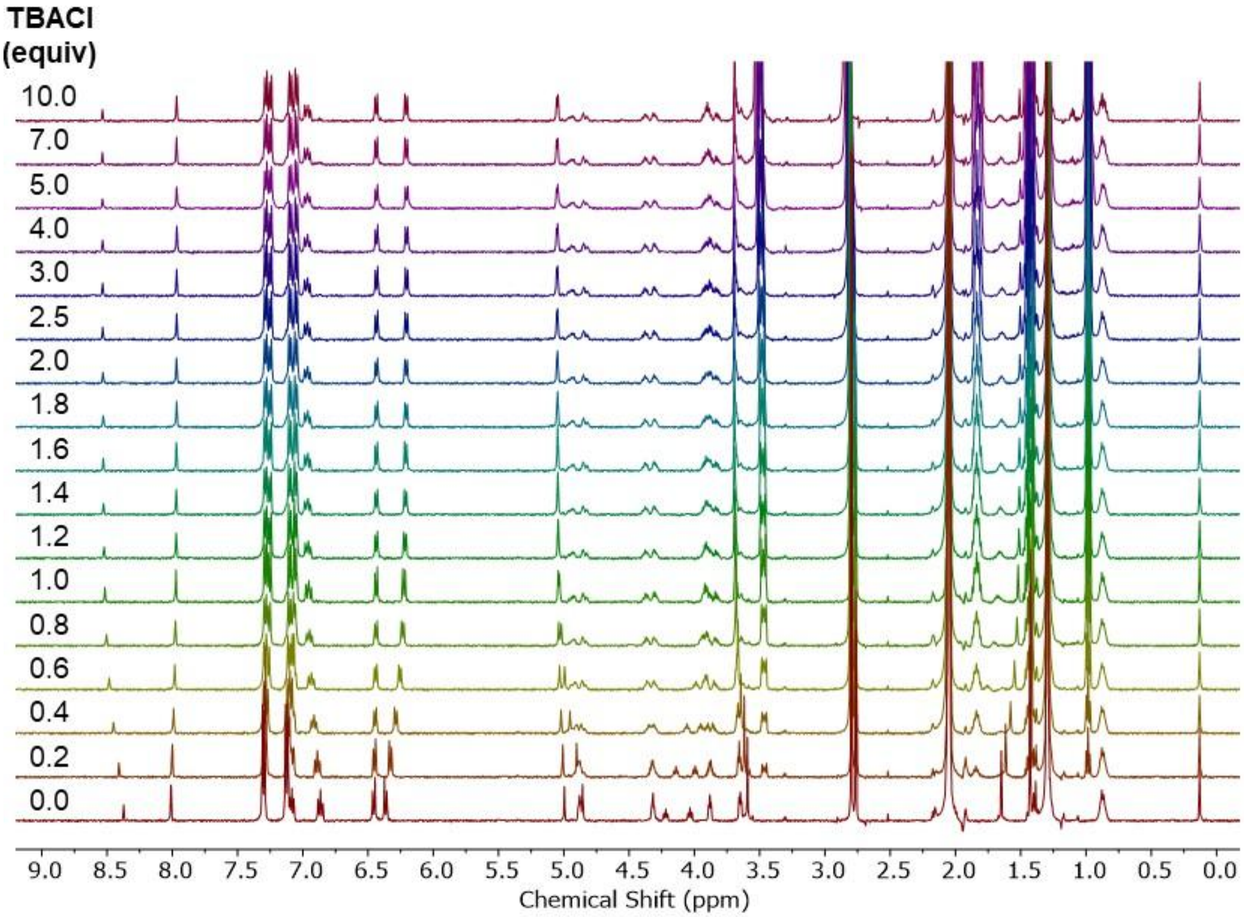

Fig. 73 | ^{1}H NMR titration spectra of **9·XB$_3$ChB** upon addition of 10 equivalents of TBACl ([**9·XB$_3$ChB**] = 1.0 mM, 500 MHz, 298 K, acetone-d_6).

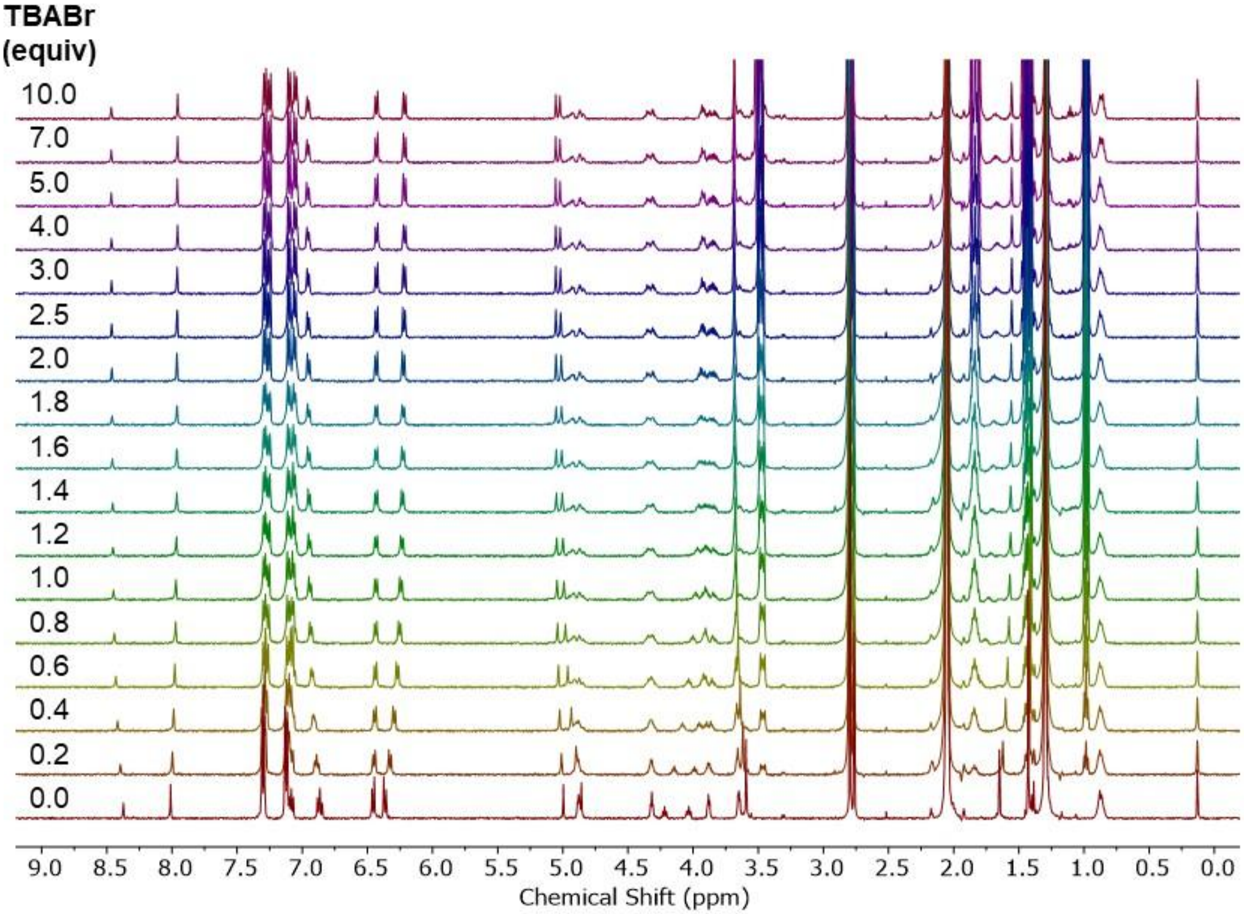

Fig. 74 | ^{1}H NMR titration spectra of **9·XB$_3$ChB** upon addition of 10 equivalents of TBABr ([**9·XB$_3$ChB**] = 1.0 mM, 500 MHz, 298 K, acetone-*d*$_6$).

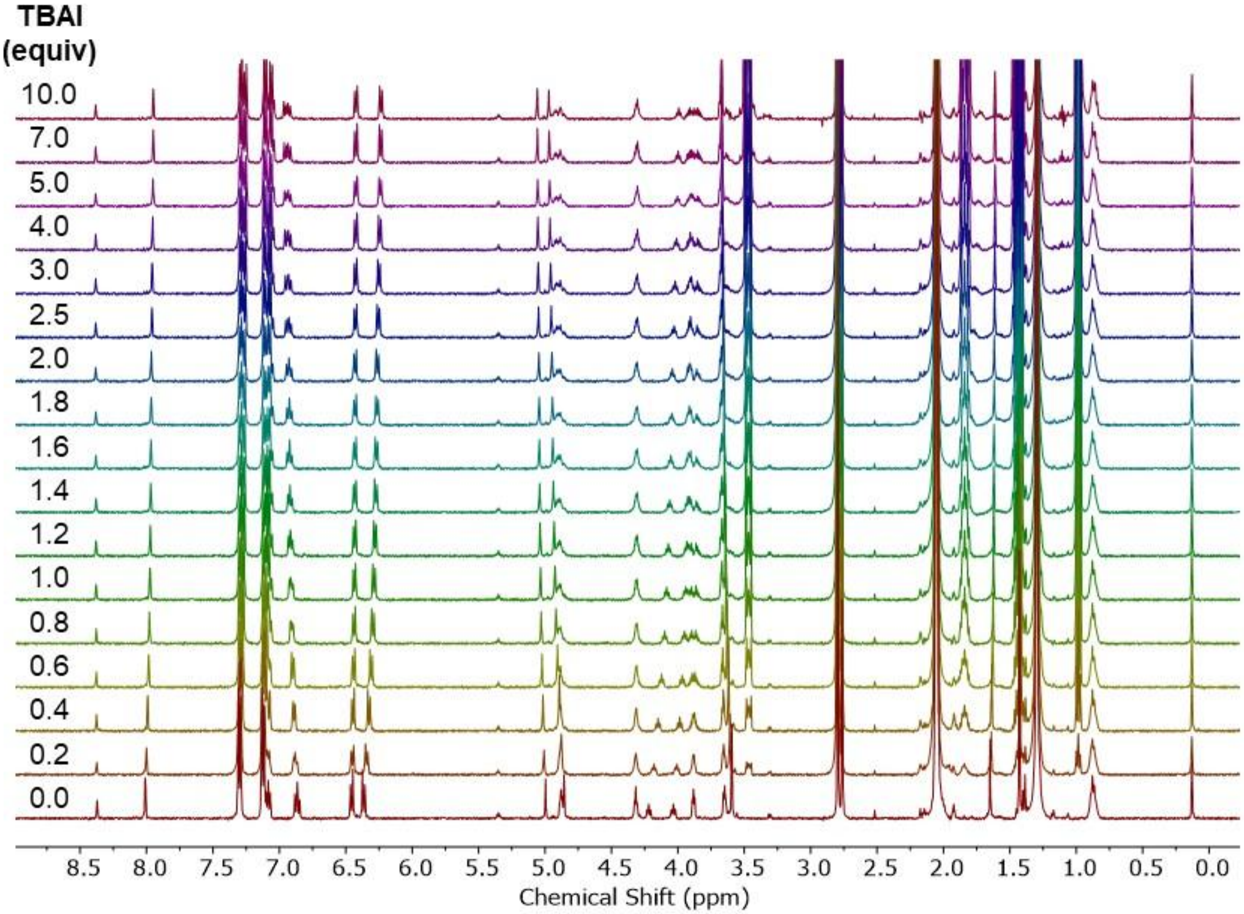

Fig. 75 | ^{1}H NMR titration spectra of **9·XB$_3$ChB** upon addition of 10 equivalents of TBAI ([**9·XB$_3$ChB**] = 1.0 mM, 500 MHz, 298 K, acetone-d_6).

^{1}H NMR Anion Binding Isotherms

Tridentate All-ChB [2]Rotaxane (8·ChB$_3$)

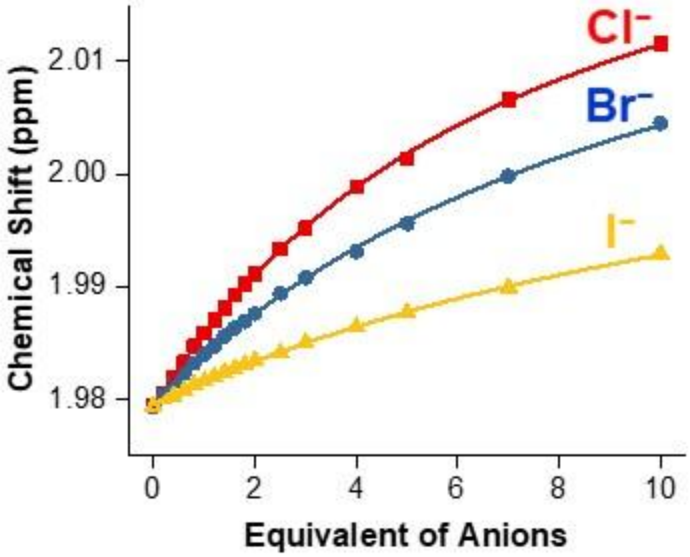

Fig. 76 | Binding isotherms of **8·ChB$_3$** showing changes in chemical shift of macrocycle telluromethyl proton h^{Te} with increasing equivalents of anions. ([**8·ChB$_3$**] = 1.0 mM, 500 MHz, 298 K, acetone-d_6).

Tridentate Mixed XB/ChB [2]Rotaxane (8·XB$_2$ChB^{Me})

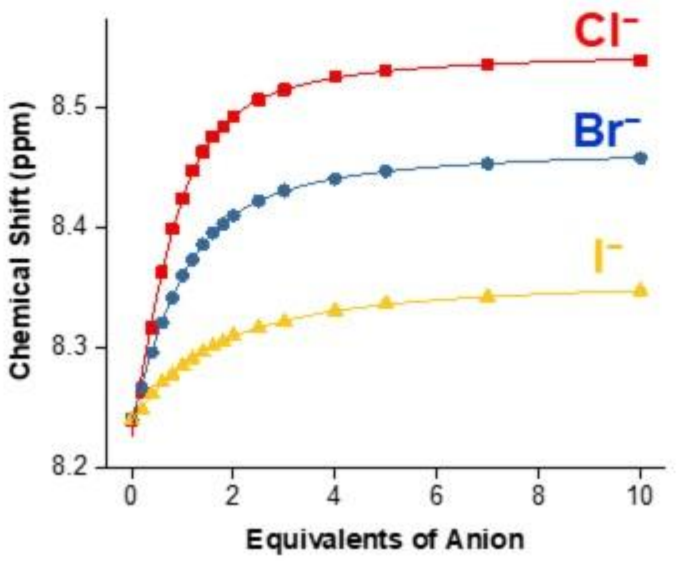

Fig. 77 | Binding isotherms of **8·XB$_2$ChB^{Me}** showing changes in chemical shift of macrocycle internal benzene proton *a* with increasing equivalents of anions. ([**8·XB$_2$ChB^{Me}**] = 1.0 mM, 500 MHz, 298 K, acetone-d_6).

Tridentate Mixed XB/ChB [2]Rotaxanes (8·$XB_2ChB^{R'}$)

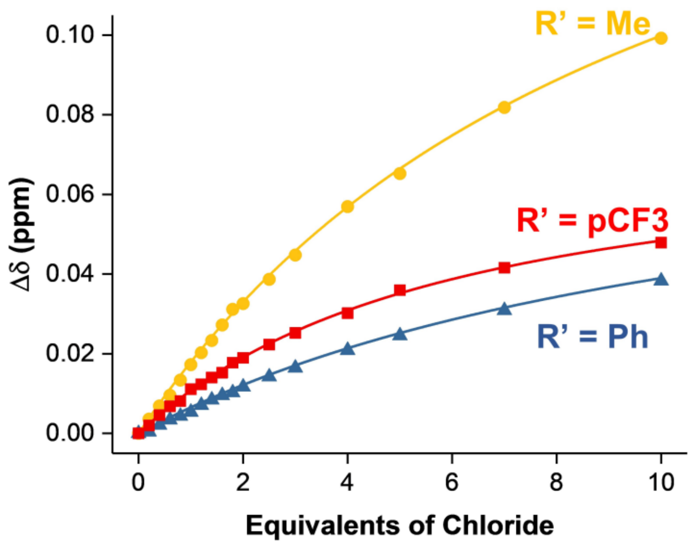

Fig. 78 | Binding isotherms of **8·XB_2ChB^{Me}**, **8·XB_2ChB^{Ph}** and **8·XB_2ChB^{pCF3}** generated by monitoring the change in chemical shift (Δδ) of internal benzene proton *a* as a function of equivalents of chloride anions added (500 MHz, 2:98 D_2O/acetone-d_6, 298 K, [Rotaxane] = 1.0 mM).

Tetradentate All-ChB [2]Rotaxane (9·ChB_4)

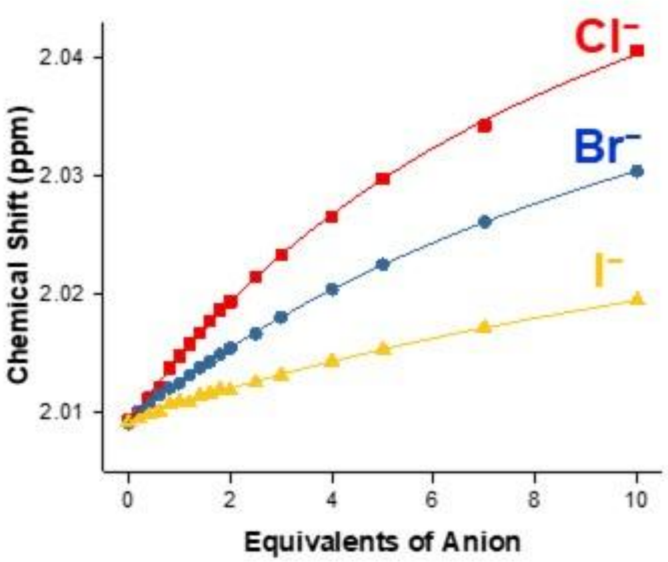

Fig. 79 | Binding isotherms of **9·ChB_4** showing changes in chemical shift of macrocycle telluromethyl proton h^{Te} with increasing equivalents of anions. ([**9·ChB_4**] = 0.5 mM, 500 MHz, 298 K, acetone-d_6).

Tetradentate Mixed XB/ChB [2]Rotaxane (9·ChB_3XB)

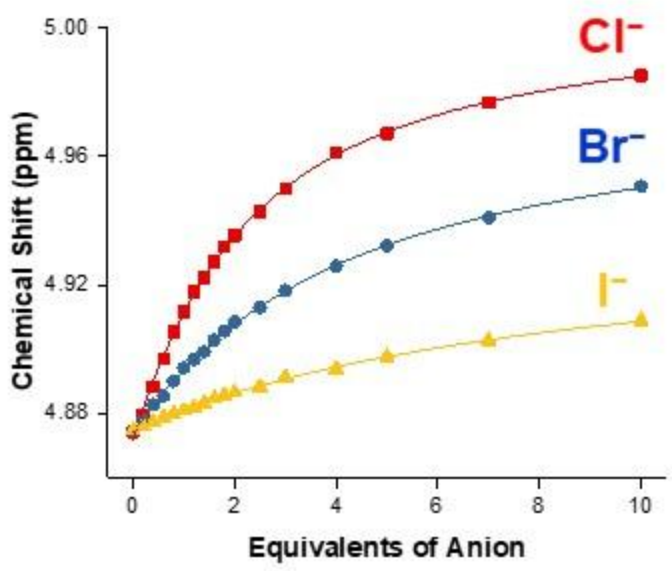

Fig. 80 | Binding isotherms of **9·ChB_3XB** showing changes in chemical shift of axle methylene proton *10* with increasing equivalents of anions. ([**9·ChB_3XB**] = 1.0 mM, 500 MHz, 298 K, acetone-d_6).

Tetradentate Mixed XB/ChB [2]Rotaxane (9·XB_2ChB_2)

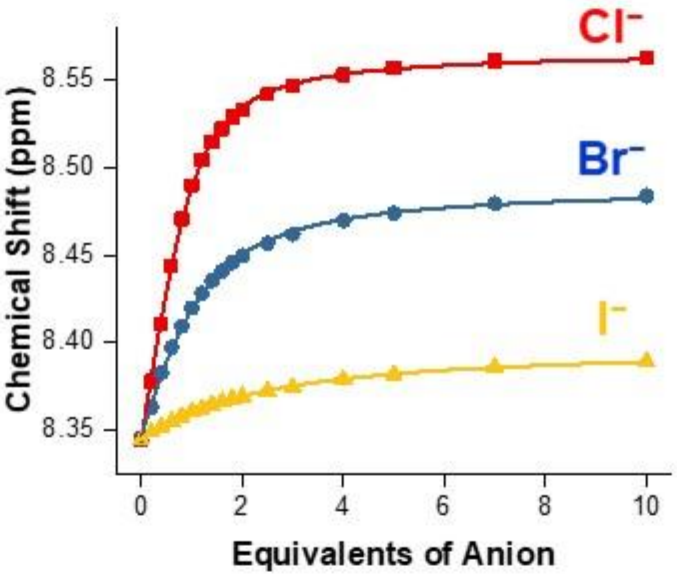

Fig. 81 | Binding isotherms of **9·XB_2ChB_2** showing changes in chemical shift of macrocycle internal benzene proton *a* with increasing equivalents of anions. ([**9·XB_2ChB_2**] = 0.5 mM, 500 MHz, 298 K, acetone-d_6).

Tetradentate Mixed XB/ChB [2]Rotaxane (9·XB$_3$ChB)

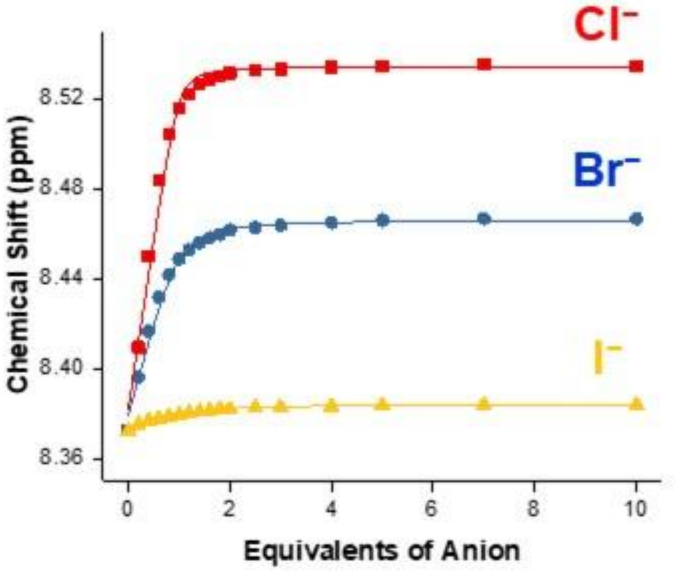

Fig. 82 | Binding isotherms of **9·XB$_3$ChB** showing changes in chemical shift of macrocycle internal benzene proton *a* with increasing equivalents of anions. ([**9·XB$_3$ChB**] = 1.0 mM, 500 MHz, 298 K, acetone-d_6).

S4 ^{1}H NMR Cation Binding Studies

General Procedures

^{1}H NMR titration experiments were performed on a Bruker AVIII 500 MHz spectrometer. In a typical experiment, a solution of $[Cu(CH_3CN)_4]PF_6$, $AgPF_6$ or $TlPF_6$ was added to a solution of the receptor molecule at 298 K in CD_3CN. Both metal salt and receptor were dissolved in the same solvent. PF_6^- was chosen as the counter-anion due to its non-coordinating nature. A 50 mM solution of the salt was added to 500 μL of a 1.0 mM solution of receptor, where 1.0 equivalent of salt added corresponds to 10.0 μL of the salt solution. 17 data points corresponding to 0.0, 0.2, 0.4, 0.6, 0.8, 1.0, 1.2, 1.4, 1.6, 1.8, 2.0, 2.5, 3.0, 4.0, 5.0, 7.0 and 10.0 equivalents of added guest anion were obtained. The binding of cations with all receptors were found to be fast on the NMR timescale. All of the titrations were performed in triplicate, with the exception of those which demonstrated no measurable binding, and those which possessed K_a values larger than can be accurately determined by NMR titration which were performed in duplicate.

For cation titrations, only one representative spectrum is shown for each titration in Fig. 83–101, followed by the binding isotherms obtained by monitoring the changes in the chemical shift of macrocycle internal benzene proton *a* (Fig. 102–107) as a function of cation concentration. The values of the observed chemical shifts and guest concentration at each titration data point were input into the BindFit[11–13] software, with initial estimates of K_a and limiting chemical shifts. These parameters were refined using non-linear least-squares analyses to obtain the best fit between the empirical and calculated chemical shifts based on a 1:1 host-guest binding model. The input parameters were iteratively varied until convergence of the best fit values of K_a were obtained. K_a values of all of the repeats of the titrations were within 1-2% of those values summarised in Research Article Table 2, and the errors quoted were derived from the fitting error of the representative titration experiment, with the value of which was the median of the triplicate.

^{1}H NMR Cation Titration Spectra

ChB Macrocycle (3·Te^{Me})

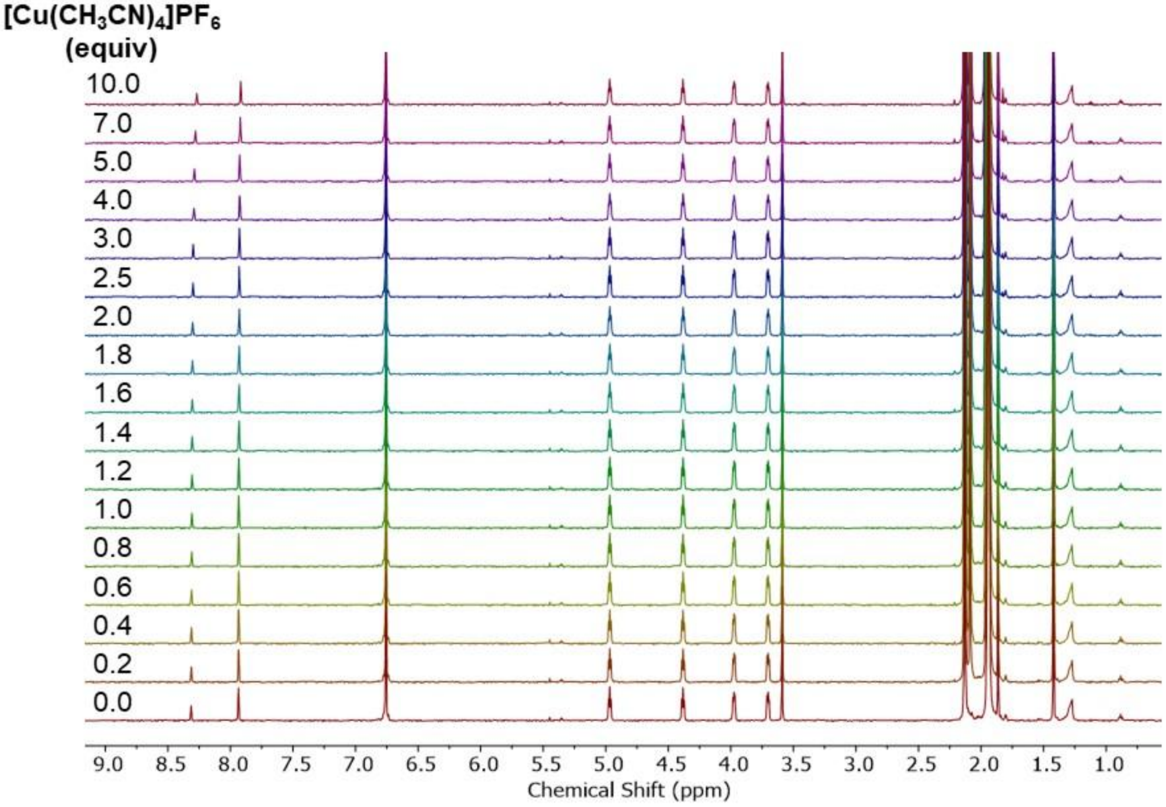

Fig. 83 | ^{1}H NMR titration spectra of **3·Te^{Me}** upon addition of 10 equivalents of $[Cu(CH_3CN)_4]PF_6$ ([**3·Te^{Me}**] = 1.0 mM, 500 MHz, 298 K, CD_3CN).

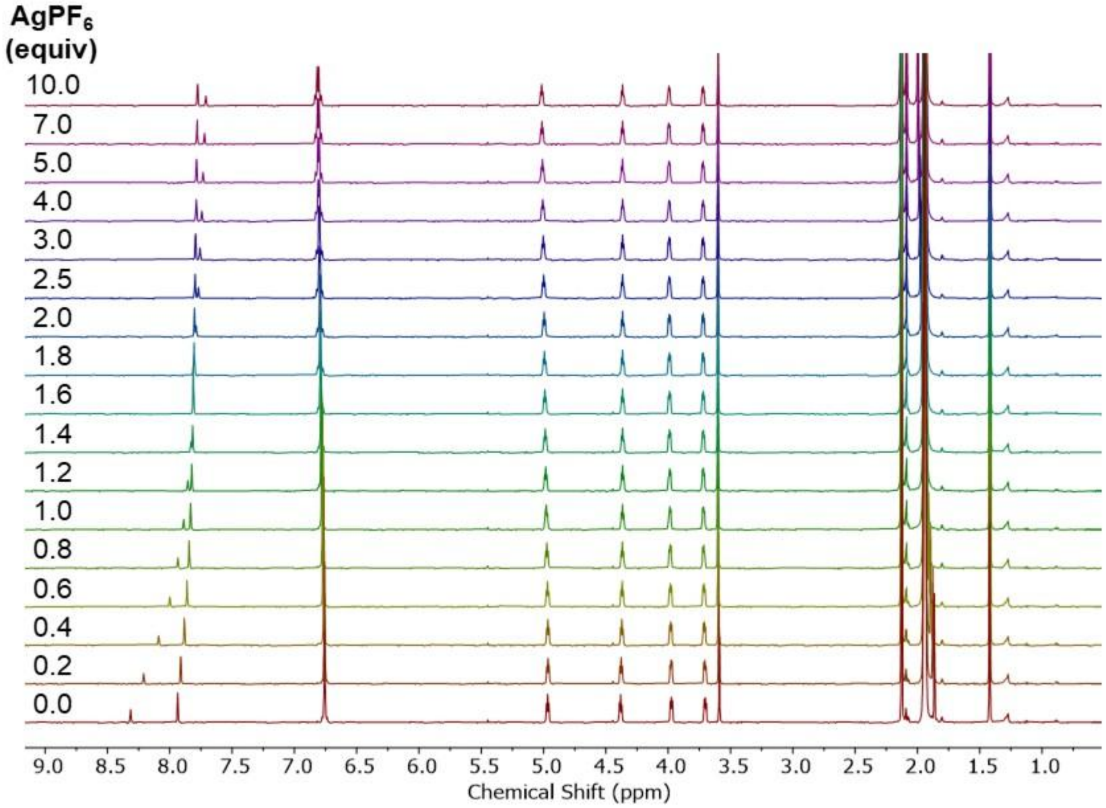

Fig. 84 | ^{1}H NMR titration spectra of **3·Te^{Me}** upon addition of 10 equivalents of $AgPF_6$ ([**3·Te^{Me}**] = 1.0 mM, 500 MHz, 298 K, CD_3CN).

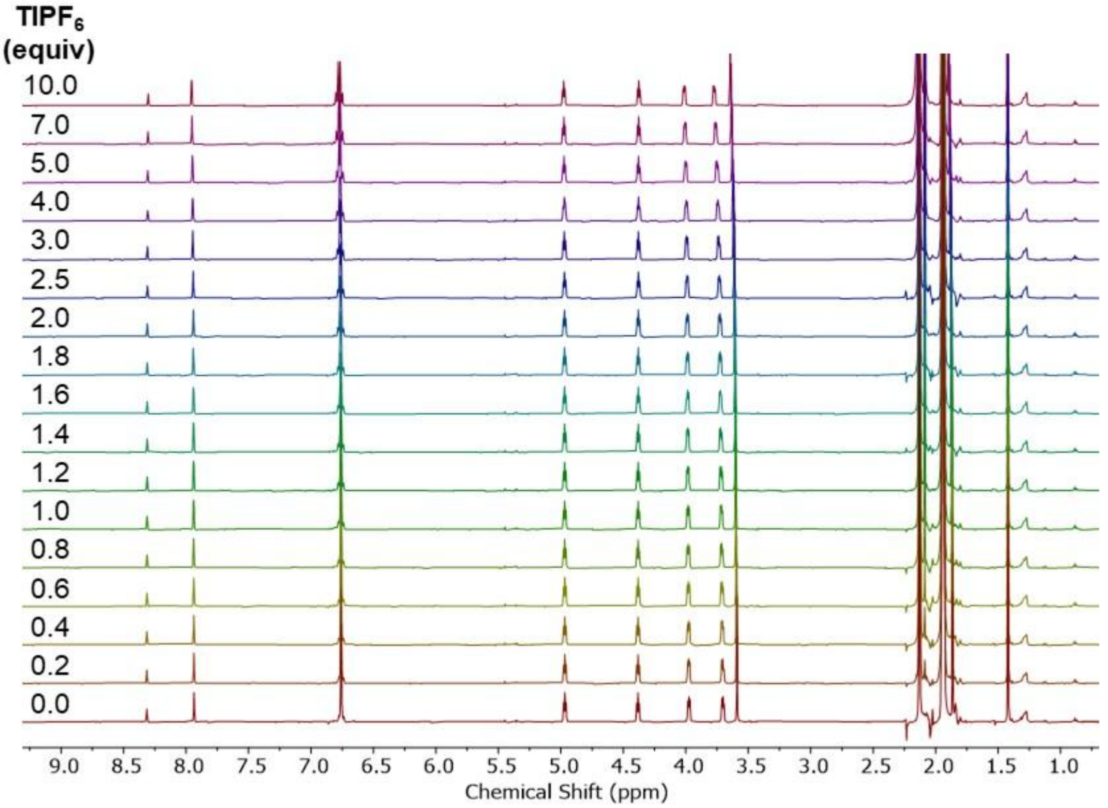

Fig. 85 | ^{1}H NMR titration spectra of **3·Te^{Me}** upon addition of 10 equivalents of $TlPF_6$ ([**3·Te^{Me}**] = 1.0 mM, 500 MHz, 298 K, CD_3CN).

XB Macrocycle (3·I)

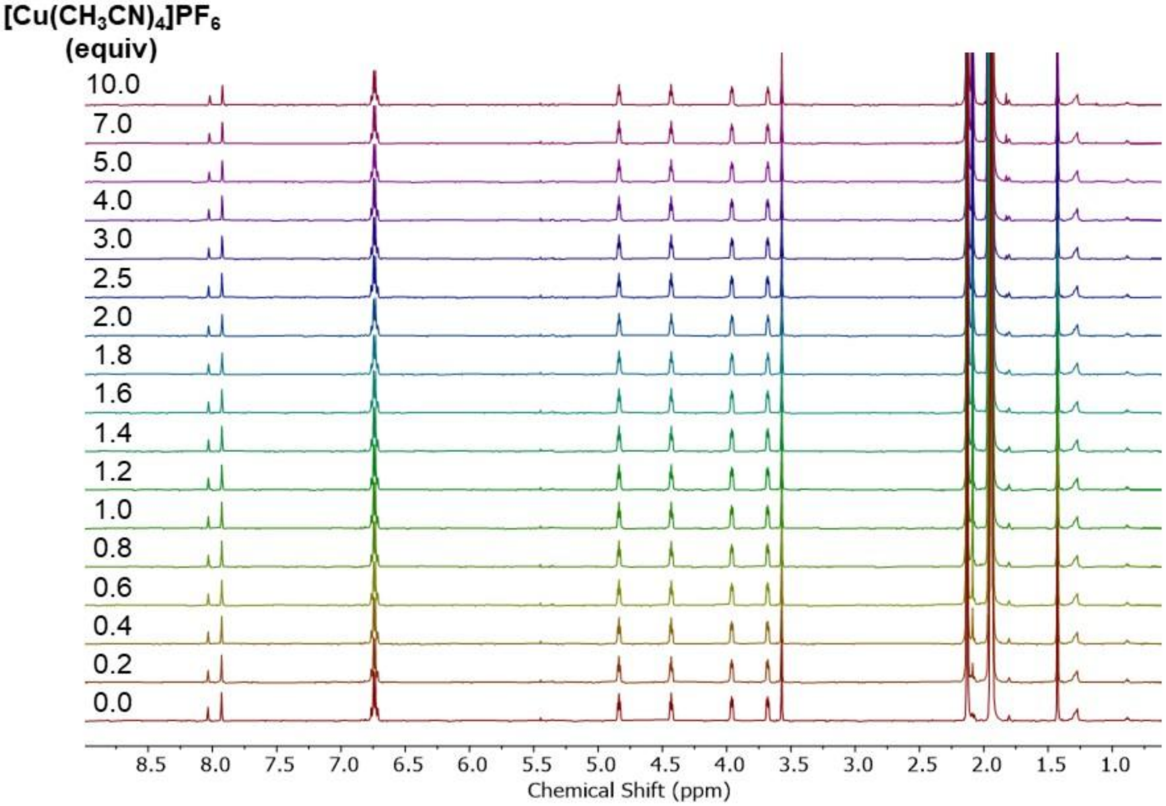

Fig. 86 | ^{1}H NMR titration spectra of **3·I** upon addition of 10 equivalents of $[Cu(CH_3CN)_4]PF_6$ ([**3·I**] = 1.0 mM, 500 MHz, 298 K, CD_3CN).

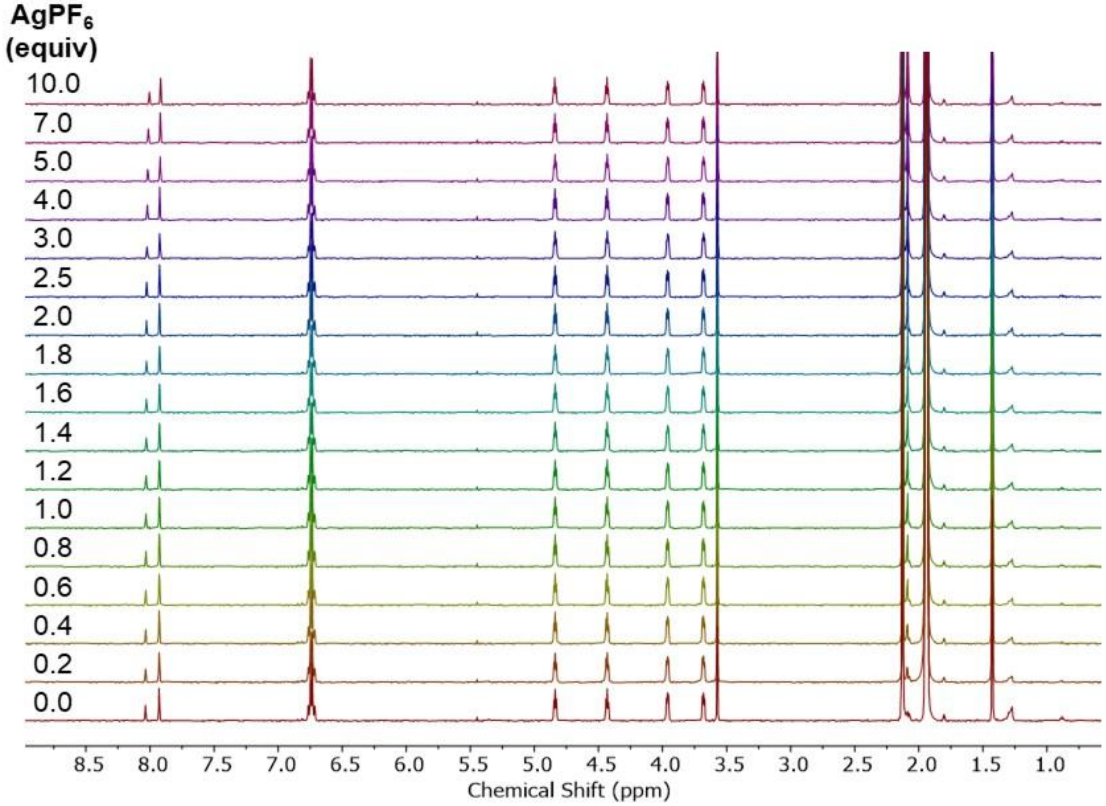

Fig. 87 | ^{1}H NMR titration spectra of **3·I** upon addition of 10 equivalents of $AgPF_6$ ([**3·I**] = 1.0 mM, 500 MHz, 298 K, CD_3CN).

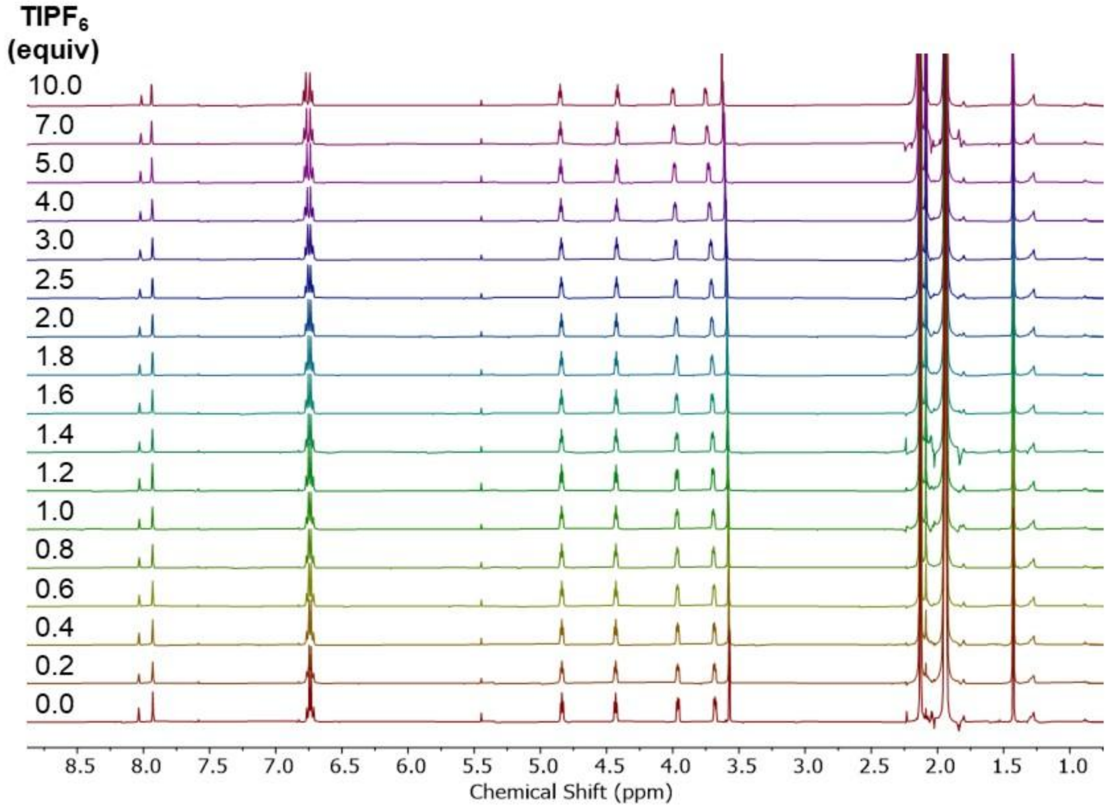

Fig. 88 | ^{1}H NMR titration spectra of **3·I** upon addition of 10 equivalents of $TlPF_6$ ([**3·I**] = 1.0 mM, 500 MHz, 298 K, CD_3CN).

Tridentate All-ChB [2]Rotaxane (8·ChB$_3$)

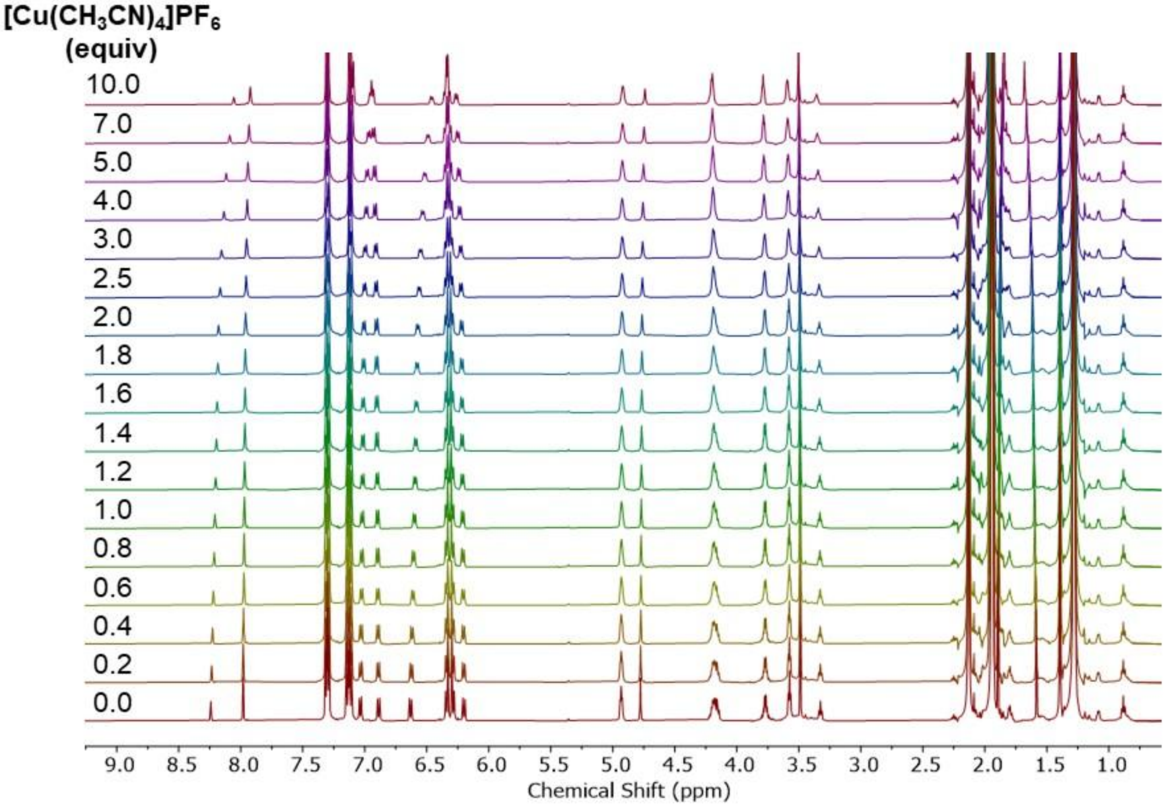

Fig. 89 | ^{1}H NMR titration spectra of **8·ChB$_3$** upon addition of 10 equivalents of $[Cu(CH_3CN)_4]PF_6$ ([**8·ChB$_3$**] = 1.0 mM, 500 MHz, 298 K, CD_3CN).

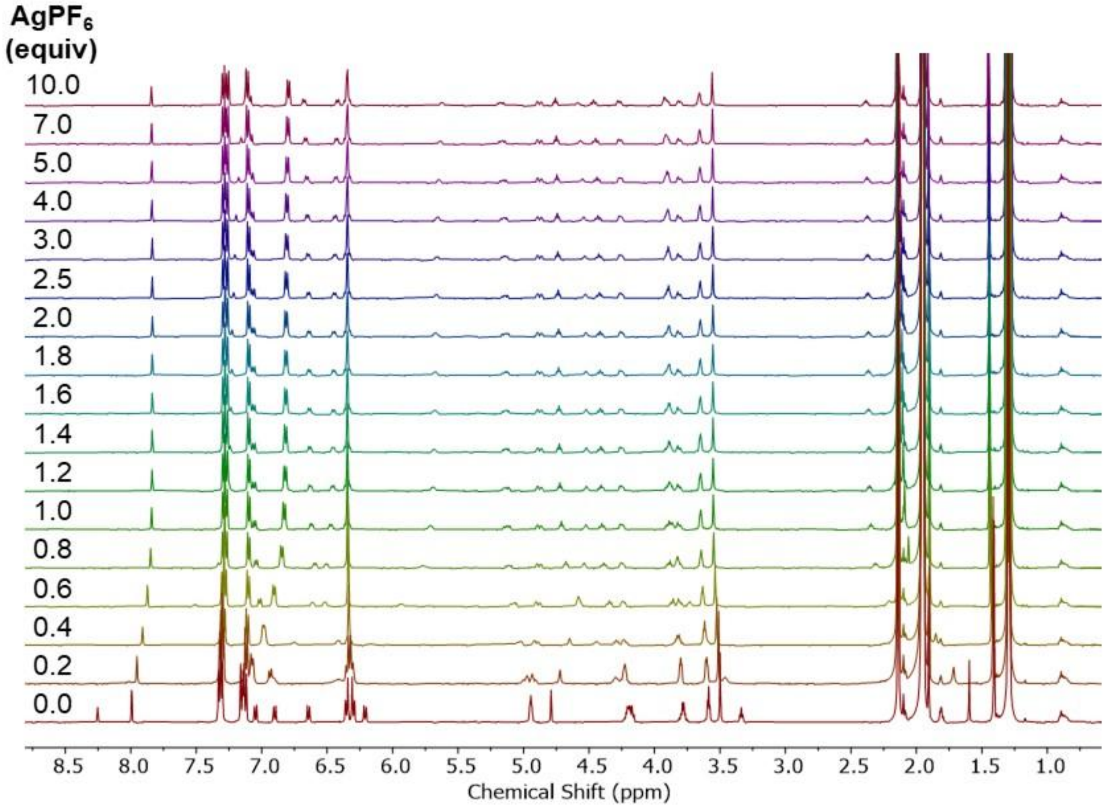

Fig. 90 | ^{1}H NMR titration spectra of **8·ChB$_3$** upon addition of 10 equivalents of $AgPF_6$ ([**8·ChB$_3$**] = 1.0 mM, 500 MHz, 298 K, CD_3CN).

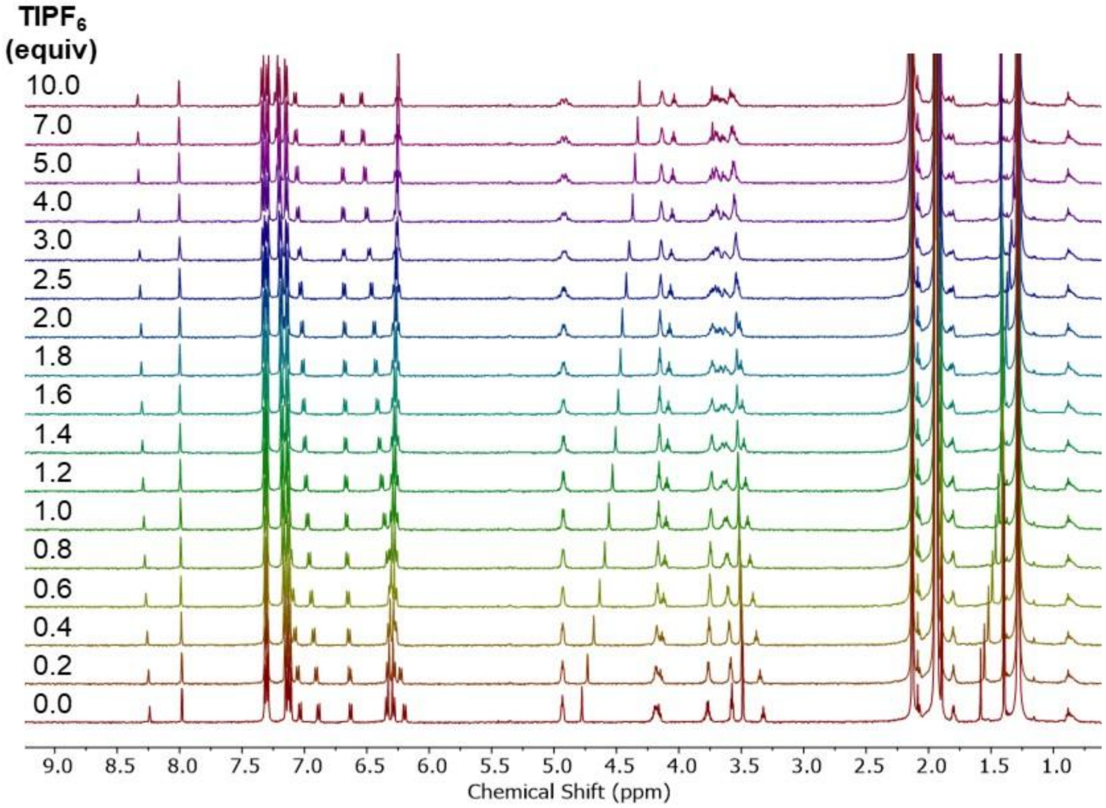

Fig. 91 | ^{1}H NMR titration spectra of **8·ChB$_3$** upon addition of 10 equivalents of $TlPF_6$ ([**8·ChB$_3$**] = 1.0 mM, 500 MHz, 298 K, CD_3CN).

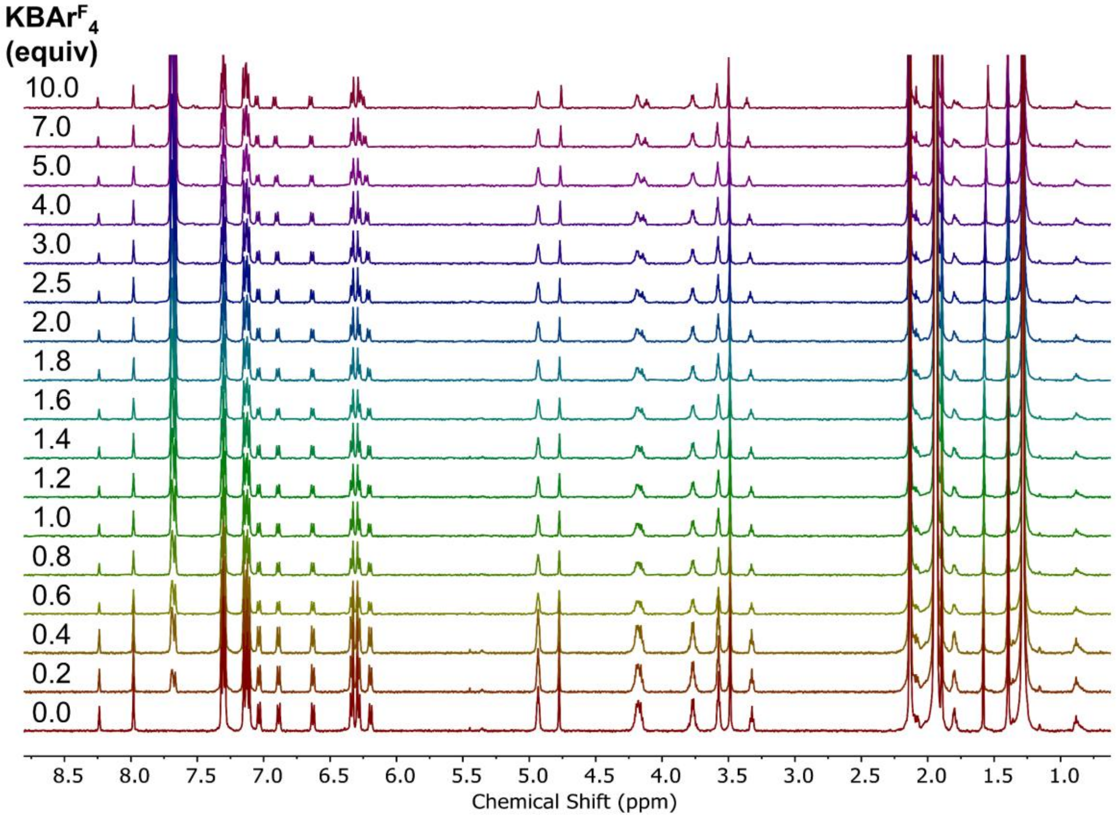

Fig. 92 | ^{1}H NMR titration spectra of **8·ChB$_3$** upon addition of 10 equivalents of KBAr$^F_4$ ([**8·ChB$_3$**] = 1.0 mM, 500 MHz, 298 K, CD$_3$CN).

Tridentate All-XB [2]Rotaxane (8·XB$_3$)

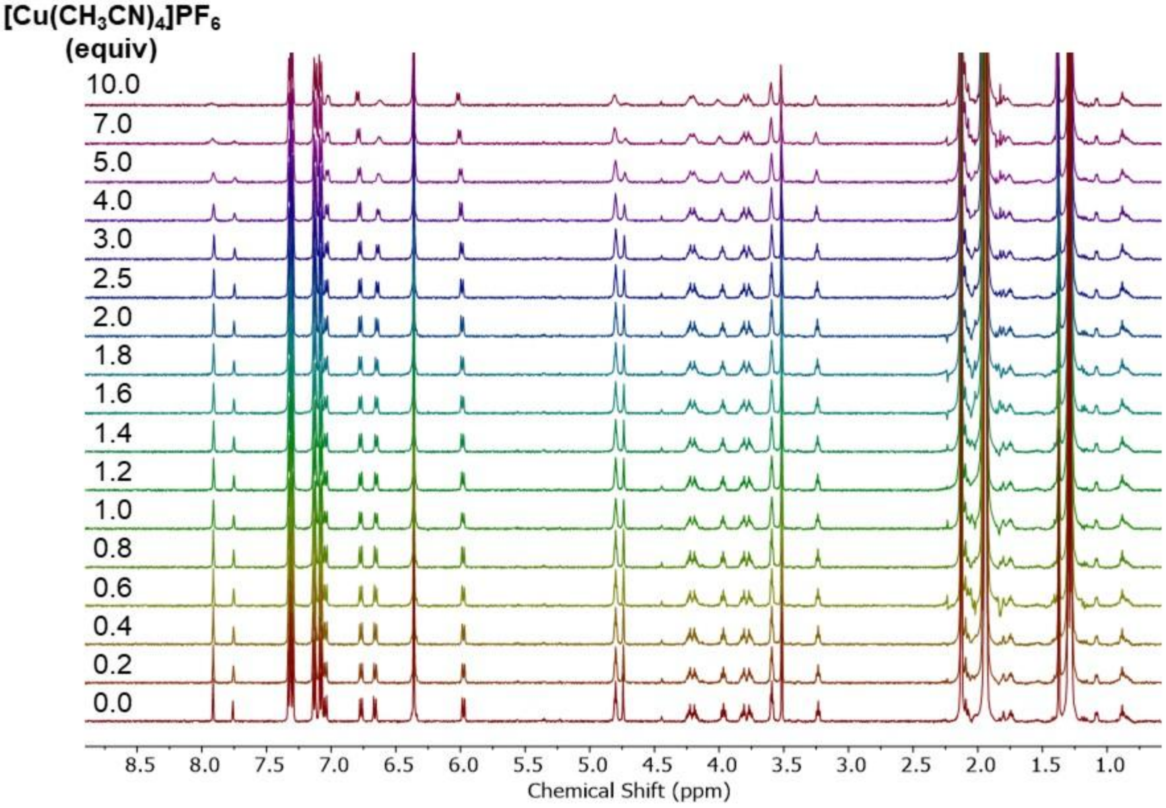

Fig. 93 | ^{1}H NMR titration spectra of **8·XB$_3$** upon addition of 10 equivalents of $[Cu(CH_3CN)_4]PF_6$ ([**8·XB$_3$**] = 1.0 mM, 500 MHz, 298 K, CD_3CN).

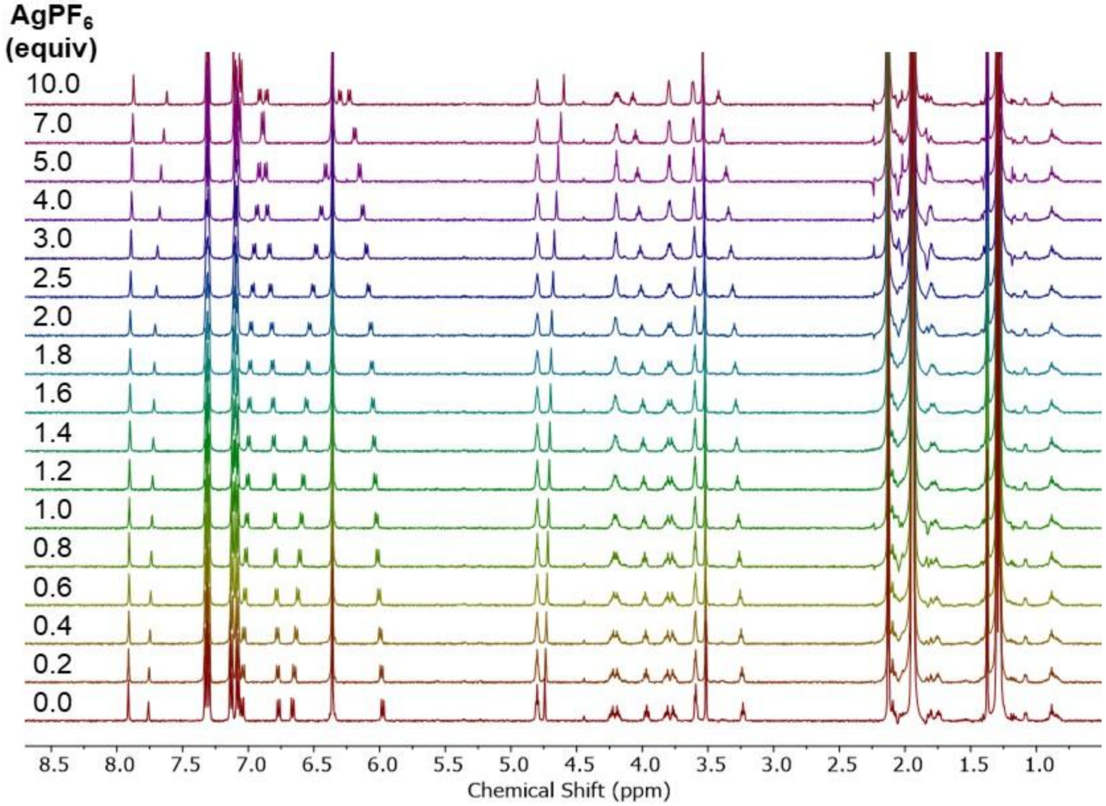

Fig. 94 | ^{1}H NMR titration spectra of **8·XB$_3$** upon addition of 10 equivalents of $AgPF_6$ ([**8·XB$_3$**] = 1.0 mM, 500 MHz, 298 K, CD_3CN).

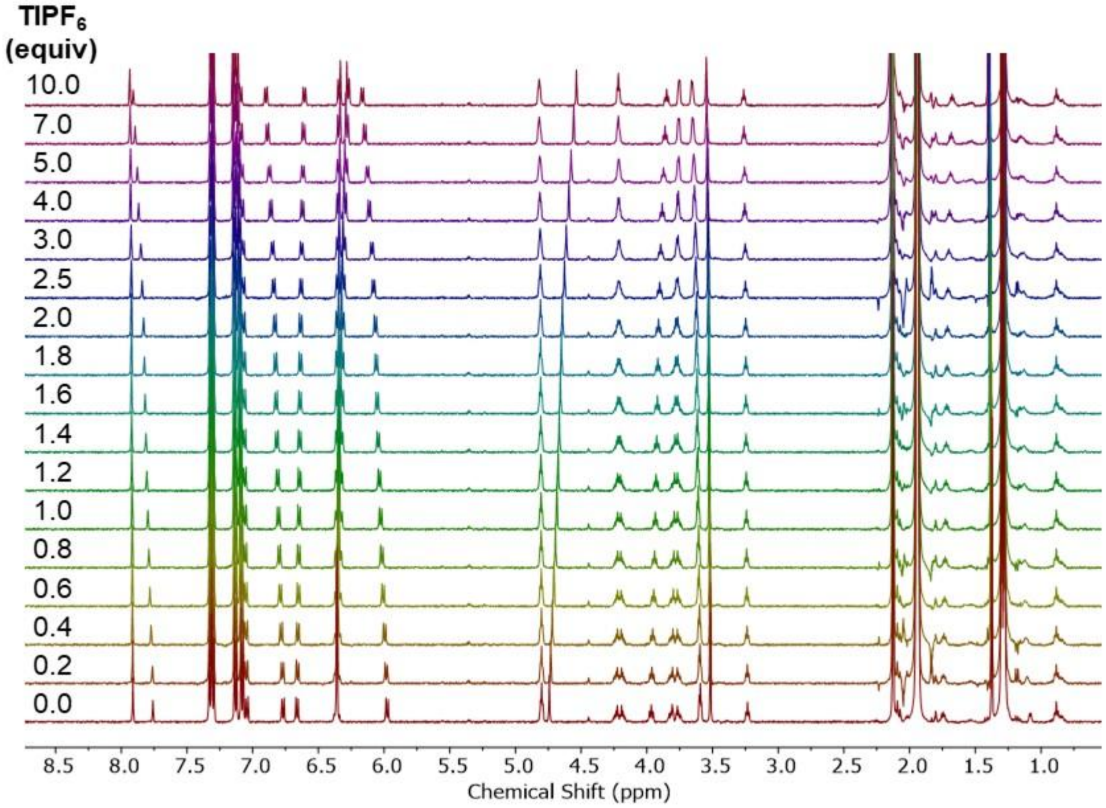

Fig. 95 | ^{1}H NMR titration spectra of **8·XB$_3$** upon addition of 10 equivalents of $TlPF_6$ ([**8·XB$_3$**] = 1.0 mM, 500 MHz, 298 K, CD_3CN).

Tridentate All-HB [2]Rotaxane (8·HB$_3$)

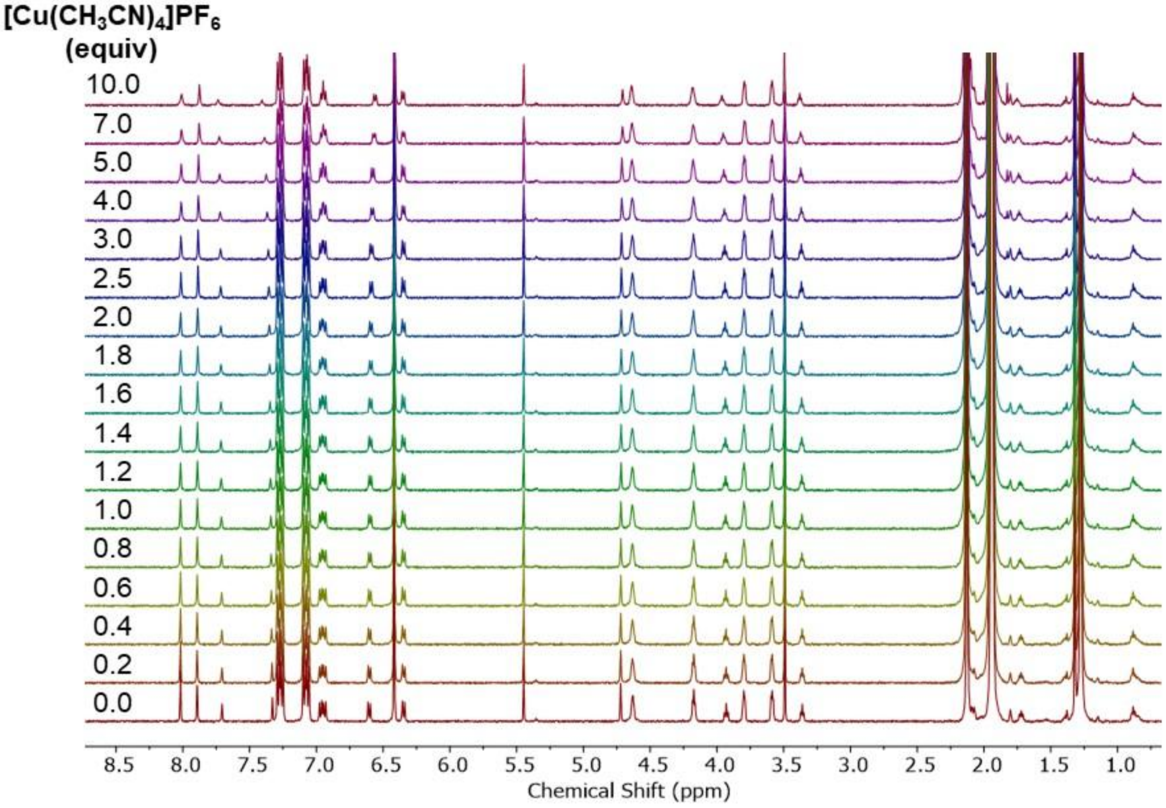

Fig. 96 | ^{1}H NMR titration spectra of **8·HB$_3$** upon addition of 10 equivalents of [Cu(CH_3CN)$_4$]PF_6 ([**8·HB$_3$**] = 1.0 mM, 500 MHz, 298 K, CD_3CN).

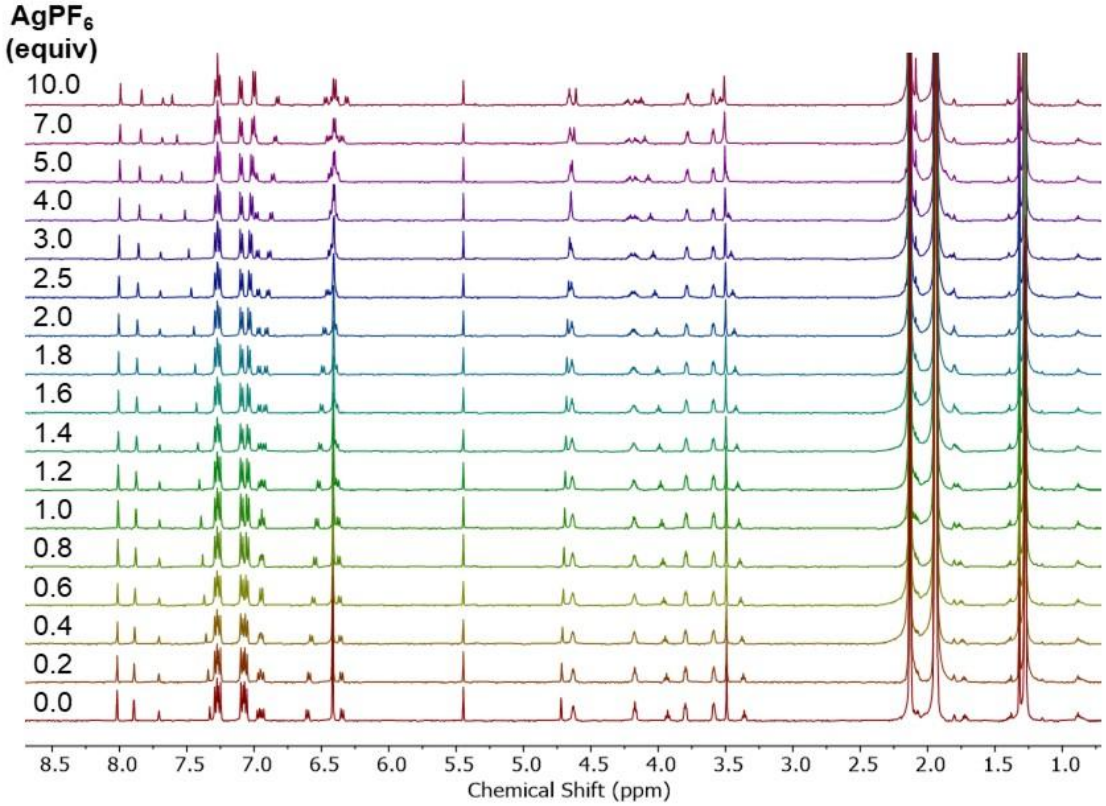

Fig. 97 | ^{1}H NMR titration spectra of **8·HB$_3$** upon addition of 10 equivalents of $AgPF_6$ ([**8·HB$_3$**] = 1.0 mM, 500 MHz, 298 K, CD_3CN).

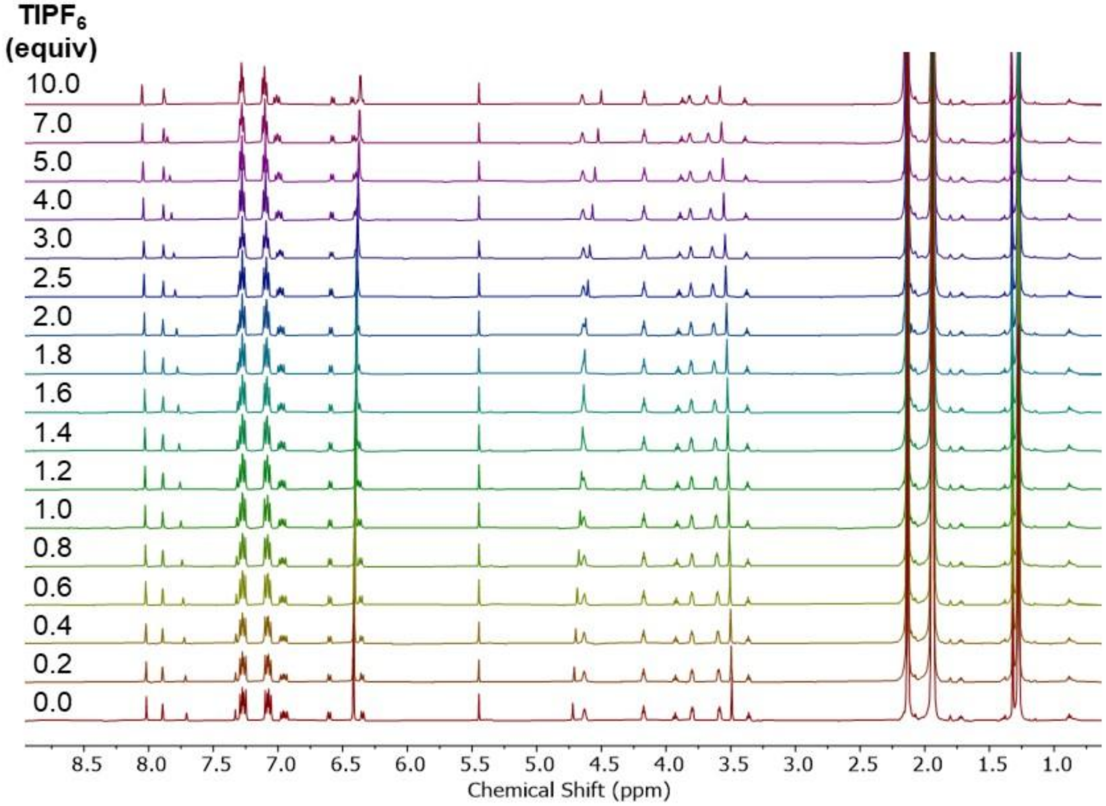

Fig. 98 | ^{1}H NMR titration spectra of **8·HB$_3$** upon addition of 10 equivalents of $TlPF_6$ ([**8·HB$_3$**] = 1.0 mM, 500 MHz, 298 K, CD_3CN).

Tetradentate All-ChB [2]Rotaxane (9·ChB$_4$)

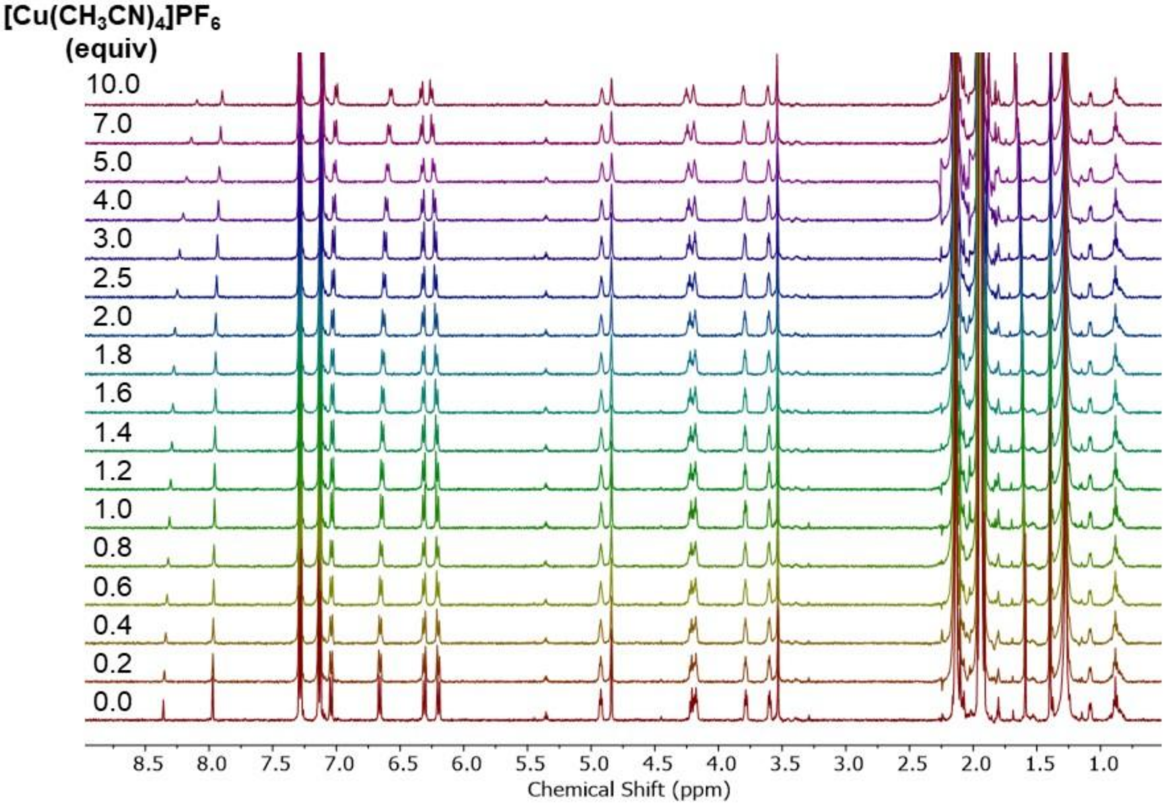

Fig. 99 | ^{1}H NMR titration spectra of **9·ChB$_4$** upon addition of 10 equivalents of $[Cu(CH_3CN)_4]PF_6$ ([**9·ChB$_4$**] = 1.0 mM, 500 MHz, 298 K, CD_3CN).

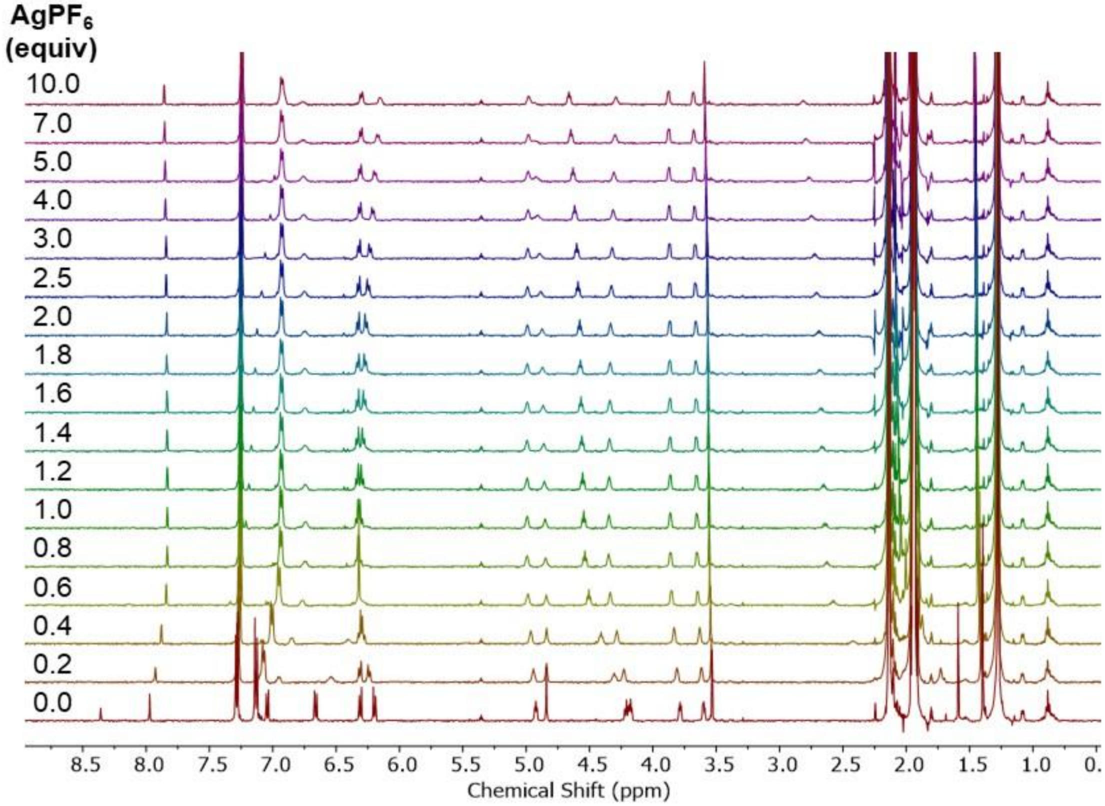

Fig. 100 | ^{1}H NMR titration spectra of **9·ChB$_4$** upon addition of 10 equivalents of AgPF$_6$ ([**9·ChB$_4$**] = 1.0 mM, 500 MHz, 298 K, CD$_3$CN).

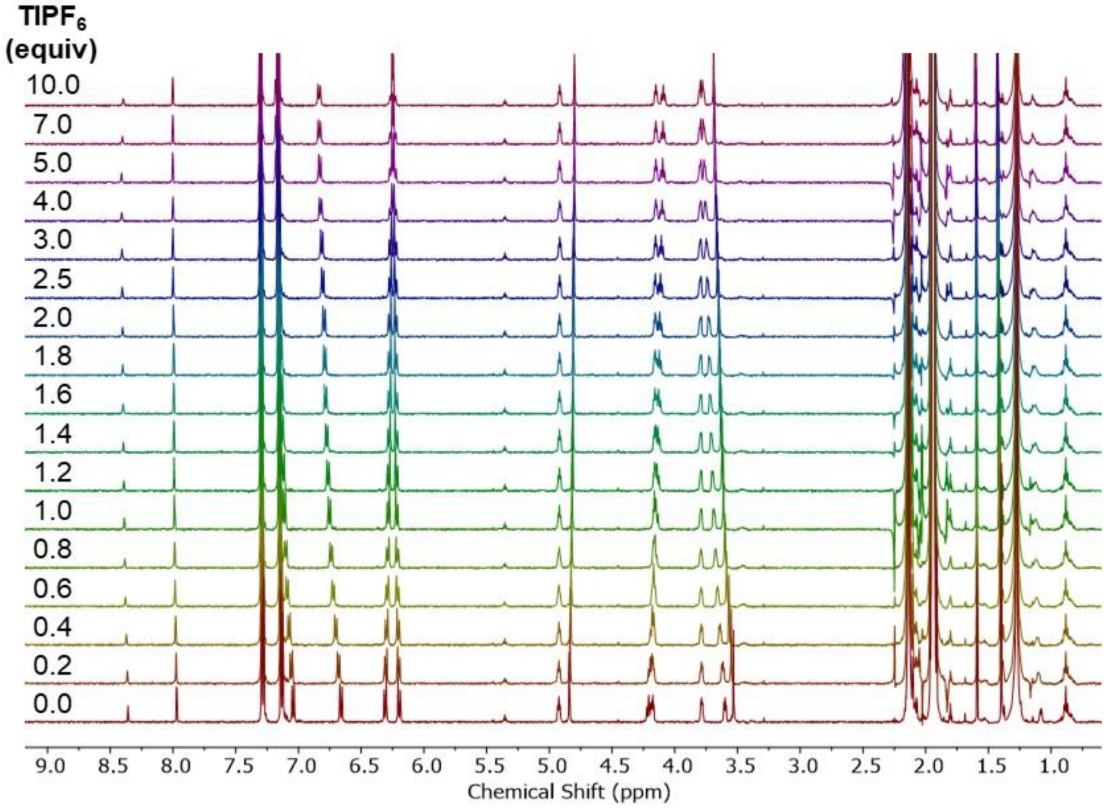

Fig. 101 | ^{1}H NMR titration spectra of **9·ChB$_4$** upon addition of 10 equivalents of $TlPF_6$ ([**9·ChB$_4$**] = 1.0 mM, 500 MHz, 298 K, CD_3CN).

^{1}H NMR Cation Binding Isotherms

ChB Macrocycle (3·Te^{Me})

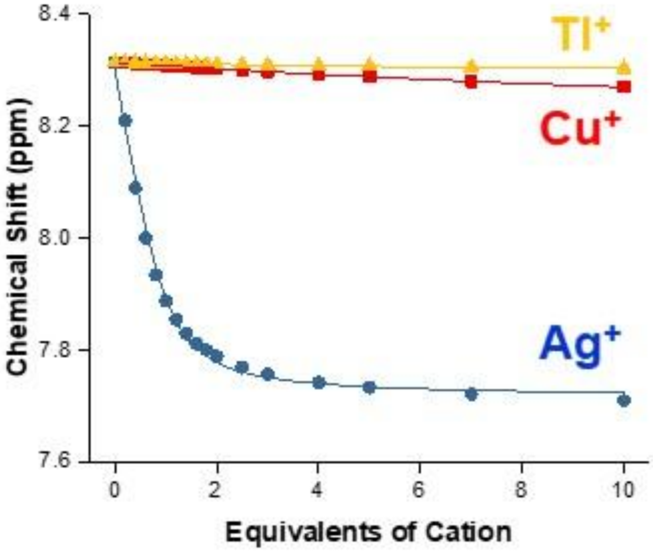

Fig. 102 | Binding isotherms showing changes in chemical shift of internal benzene proton *a* with increasing equivalents of metal cations. ([**3·Te^{Me}**] = 1.0 mM, 500 MHz, 298 K, CD_3CN).

XB Macrocycle (3·I)

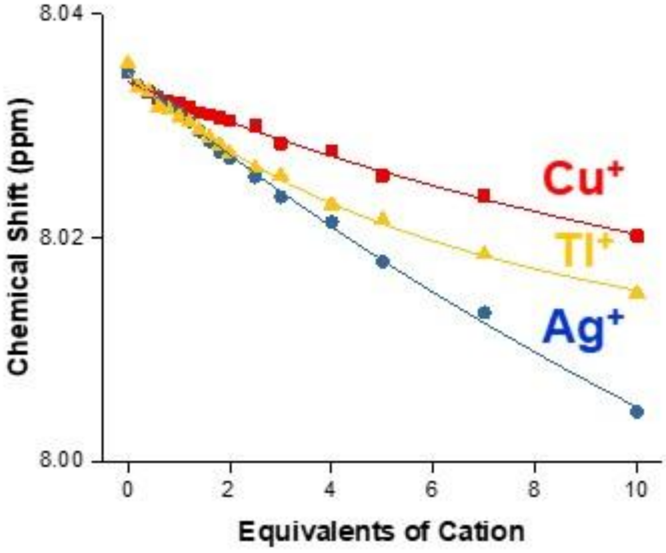

Fig. 103 | Binding isotherms showing changes in chemical shift of internal benzene proton *a* with increasing equivalents of metal cations. ([**3·I**] = 1.0 mM, 500 MHz, 298 K, CD_3CN).

Tridentate All-ChB [2]Rotaxane (8·ChB$_3$)

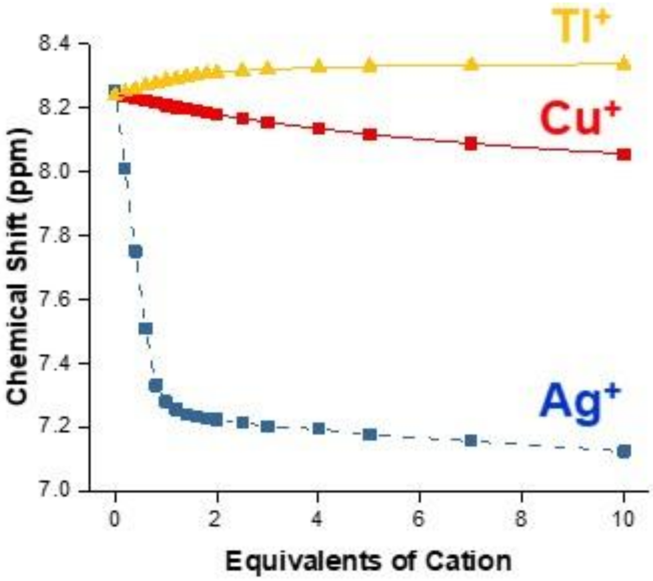

Fig. 104 | Binding isotherms showing changes in chemical shift of internal benzene proton *a* with increasing equivalents of metal cations. ([**8·ChB$_3$**] = 1.0 mM, 500 MHz, 298 K, CD_3CN).

Tridentate All-XB [2]Rotaxane (8·XB$_3$)

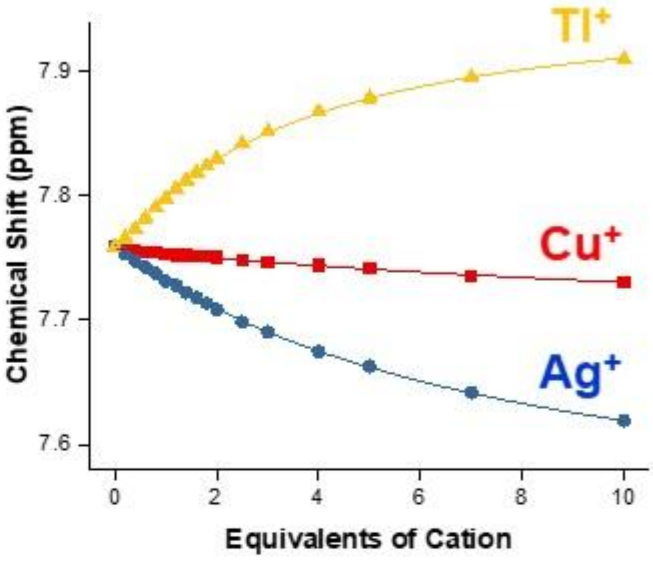

Fig. 105 | Binding isotherms showing changes in chemical shift of internal benzene proton *a* with increasing equivalents of metal cations. ([**8·XB$_3$**] = 1.0 mM, 500 MHz, 298 K, CD_3CN).

Tridentate All-HB [2]Rotaxane ($8 \cdot HB_3$)

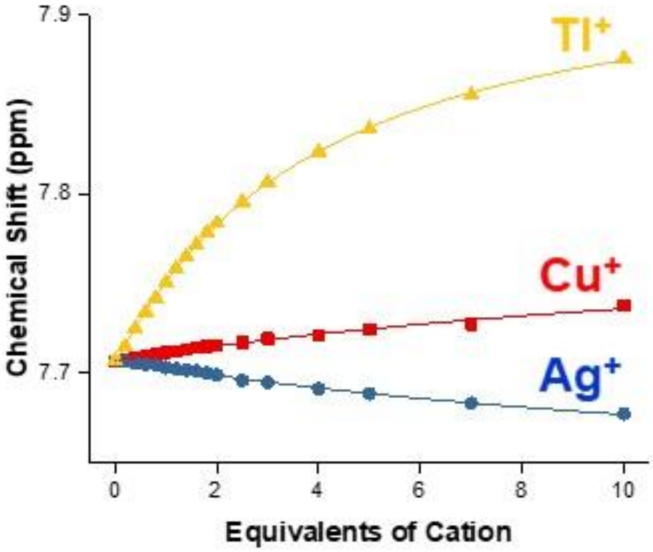

Fig. 106 | Binding isotherms showing changes in chemical shift of internal benzene proton *a* with increasing equivalents of metal cations. ([**8·HB$_3$**] = 1.0 mM, 500 MHz, 298 K, CD_3CN).

Tetradentate All-ChB [2]Rotaxane ($9 \cdot ChB_4$)

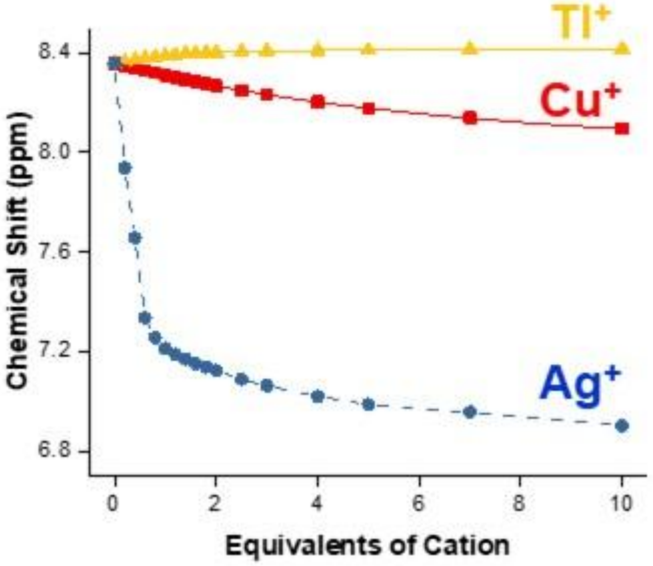

Fig. 107 | Binding isotherms showing changes in chemical shift of internal benzene proton *a* with increasing equivalents of metal cations. ([**9·ChB$_4$**] = 1.0 mM, 500 MHz, 298 K, CD_3CN).

S5 DFT Computational Modelling

Starting structures

Starting structures of macrocycles **3·Te^{Me}**, **3·I**, and **11·I**, and [2]rotaxanes **8·ChB$_3$** and **8·XB$_3$** were generated from crystal structures deposited with the Cambridge Crystallographic Data Centre (CCDC),[14] as follows. Rotaxane **8·XB$_3$** was initially constructed from the crystal structures with Refcodes DAMPAK,[15] and WOMZUW.[16] The former entry provided the structure for the axle of **8·XB$_3$**, as well as most of its macrocyclic component, whilst the bis-iodo-methylpyridinium in the latter crystal structure was used as the template for the bis-iodo-tert-phenyl moiety. The subsequent replacement of the DAMPAK's isophthalamide cleft by the bis-iodo-*tert*-phenyl moiety, through adequate atomic manipulation, yielded **8·XB$_3$** with the macrocyclic (**3·I**) and axle components correctly assembled in the orthogonal interlocked manner. Macrocycle **11·I** was generated from **3·I** with the addition of the necessary amide linked TEG chains and removal of the *tert*-butyl moiety.

8·ChB$_3$ was created by changing the iodine binding units by TeMe ones, while **3·Te^{Me}** was directly taken from the rotaxane. Due to the large size of the three [2]rotaxane receptors and for computing performance reasons, the axles' bulky 4,4',4"-(phenylmethanetriyl)tris(tert-butylbenzene) stoppers were replaced by methyl groups affording the pseudo-[2]rotaxanes **8·ChB$_3$'** and **8·XB$_3$'**. In line, the TEG chains of **11·I** were replaced with methoxyethyl ones, affording **11·I'**.

Quantum calculations

All DFT calculations were carried out with Gaussian 16, Rev. C.01.[17] Theoretical studies started with gas-phase geometry optimizations of **8·ChB$_3$'**, which were performed at the PBE0[18]-D3[19](BJ)[20]/def2-TZVP[21–23] theory level. Afterwards, using MultiWFN 3.7,[24,25] the Molecular Electrostatic Potential (MEP) was calculated for **8·ChB3'** as well as for its isolated macrocycle and axle prototype components.

DFT computational investigations on halide **8·ChB$_3$'** model complexes were carried out in acetone implicit solvent model (CPCM[26,27]) using the M06-2X[28,29] functional coupled with the def2-TZVP[21] basis set, enhanced with diffusion functions for the tellurium centres and halides (def2-TZVPD[30,31]). DFT calculations on metal complexes were undertaken at the PBE0-D3(BJ)/def2-TZVP theory level in acetonitrile. Different implicit solvent models for acetonitrile

were tested, as detailed below. All DFT optimised structures were identified as local minima characterised by the absence of negative frequencies.

The interactions between the halides or metal cations with the macrocycles or pseudo-[2]rotaxane receptors were also evaluated by post-processing the wave functions of the optimised geometries, through Natural Bond Orbital (NBO) analysis, using the NBO7 program.[32,33] NBO7 was also used to assess the atomic charges in the optimised structures of the complexes and free receptors by Natural Population Analysis, affording NPA charges.[34]

The nature of the Te···X^- and M···Te (**8·ChB$_3$'**) and M···I (**8·XB$_3$',**) interactions was further characterised using Fuzzy Bond Order (FBO) analysis[35] as implemented in MultiWFN 3.7.[24,25]

The interaction energies (Δ*E*) for metal complexes in acetonitrile were estimated through the following equation: $\Delta E = E_{\text{complex}} - E_{\text{receptor}} - E_{\text{cation}}$, with these individual terms being estimated from corresponding optimised structures of the metal complex, the receptor, and cation, respectively. Moreover, each energy term was obtained as $E = \mathcal{E}_0 + E_{\text{Tot}}$, where $\mathcal{E}_0$ is the total electronic energy and E_{Tot} is the total internal energy, accounting for contributions from the translational, rotational, vibrational, and electronic motions. Furthermore, E_{Tot} also includes the zero-point vibrational energy correction, being null for each isolated cation.

Description of ChB interactions in acetone

Following our previous theoretical studies on ChB-driven ion-pair recognition by large a heteroditopic receptor,[36] the ChB interactions were initially evaluated using the CPCM solvent model (Table 2). The need to accurately model XB interactions in solution led to the refinement of the SMD Coulomb radii for Br and I, yielding the SMD18 solvation model.[37] The refined radius of 2.60 Å is smaller than the Br default radius of 3.06 Å, while the 2.74 Å radius for I is significantly larger than the original SMD radius of 1.98 Å. On the other hand, there is a large difference between the SMD18 radius for I and the SMD radius for Te (2.06 Å), which are sequential elements in the periodic table. Despite this apparent discrepancy, we decided to ascertain the applicability of SMD18 to model the chalcogen bonding interactions of **8·ChB$_3$'**, given that only TeMe binding units are involved.[38] The DFT optimised structures, with the ChB interaction dimensions and E^2 values listed in Table, are close to the CPCM computed ones (RMSD values of 0.15 Å for Cl^-, 0.14 Å for Br^-, and 0.44 Å for I^-), namely taking into account the flexibility of this interlocked system. Despite of the low anion association constant values for **8·ChB$_3$** (Research Article Table 1), both solvation models yield average E^2 values for

8·ChB₃' that linearly correlate with them ($R^2 \geq 0.98$). Despite these resemblances, the discussion presented in the Main Text is based on the CPCM solvation model results.

Table 2 | Chalcogen bond dimensions (distances in Å, angles in °), together with NBO Second-order perturbation theory energies (E^2, kcal mol^{-1}) for the halide complexes of **8·ChB₃'** optimized in acetone at M06-2X/Def2-TZVP(D).

Solvation model	Halide	Component	Chalcogen	Te···X⁻	C_{trz}-Te···X⁻	E^2
CPCM	Cl⁻	Macrocycle	Te1	3.31	170.8	8.4
			Te2	3.60	147.3	3.0
		Axle	Te3	3.44	175.0	6.2
		Average		*3.45*	*164.4*	*5.9*
	Br⁻	Macrocycle	Te1	3.50	171.2	7.4
			Te2	3.66	154.2	4.3
		Axle	Te3	3.68	175.3	4.7
		Average		*3.61*	*166.9*	*5.5*
	I⁻	Macrocycle	Te1	3.71	172.2	6.8
			Te2	3.82	157.7	4.8
		Axle	Te3	3.92	174.7	3.9
		Average		*3.82*	*168.2*	*5.2*
SMD18	Cl⁻	Macrocycle	Te1	3.31	169.7	8.4
			Te2	3.61	147.9	3.1
		Axle	Te3	3.48	173.9	5.6
		Average		*3.47*	*164.4*	*5.7*
	Br⁻	Macrocycle	Te1	3.49	169.8	7.6
			Te2	3.68	154.2	4.2
		Axle	Te3	3.72	174.8	4.3
		Average		*3.63*	*164.4*	*5.4*
	I⁻	Macrocycle	Te1	3.73	170.0	6.4
			Te2	3.78	162.0	5.7
		Axle	Te3	3.95	177.1	3.7
		Average		*3.82*	*164.4*	*5.2*

Cation coordination by 3·Te^{Me}

The tellurium-based triazole binding motifs of macrocycle **3·Te^{Me}** were considered fluxional, able to adopt three putative conformations depending on their relative spatial orientations (Fig. 108): both pointing inwards (*endo*/*endo*), one pointing outwards (*endo*/*exo*), or both pointing outwards (*exo*/*exo*). These conformations are equally likely to occur, given that their DFT optimised structures have comparable energies, with differences not exceeding 1.2 kcal mol^{-1} between the extreme *exo*/*exo* and *endo/endo* conformations in the SMD solvation model. They were subsequently used to investigate the ability of **3·Te^{Me}** to bind Cu(I), Ag(I) or Tl(I) in a bidentate fashion through (Te,Te) (*endo*/*endo*), (Te,N) (*endo*/*exo*), or (N,N) (*exo*/*exo*) donor atoms. Overall, the tellurium-mediated *endo*/*endo* coordination mode is slightly favoured, followed by the *endo*/*exo* and *exo*/*exo* binding modes (Fig. 109). The energy differences (ΔE_{conf}, Table 3) between the lowest energy *endo*/*endo* and either the *endo*/*exo* or *exo*/*exo* conformation are found to depend on the identity of the metal cation. Larger ΔE_{conf} values were estimated for the *exo*/*exo* Cu(I) (15.7 kcal mol^{-1}) and Ag(I) (22.2 kcal mol^{-1}) complexes,

indicating that this coordination mode is highly unlikely to occur. The PCM solvation model yields equivalent conformational binding preferences, but with smaller energy differences.

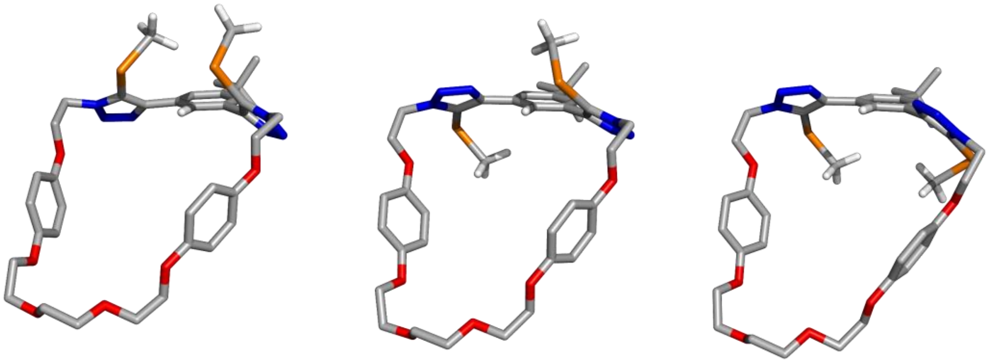

Fig. 108 | PBE0-D3(BJ)/def2-TZVP/acetonitrile–SMD optimised structures of free macrocycle **3·Te^{Me}** with triazole binding sites adopting *endo*/*endo* (left), *endo*/*exo* (centre) and *exo*/*exo* (right) spatial dispositions.

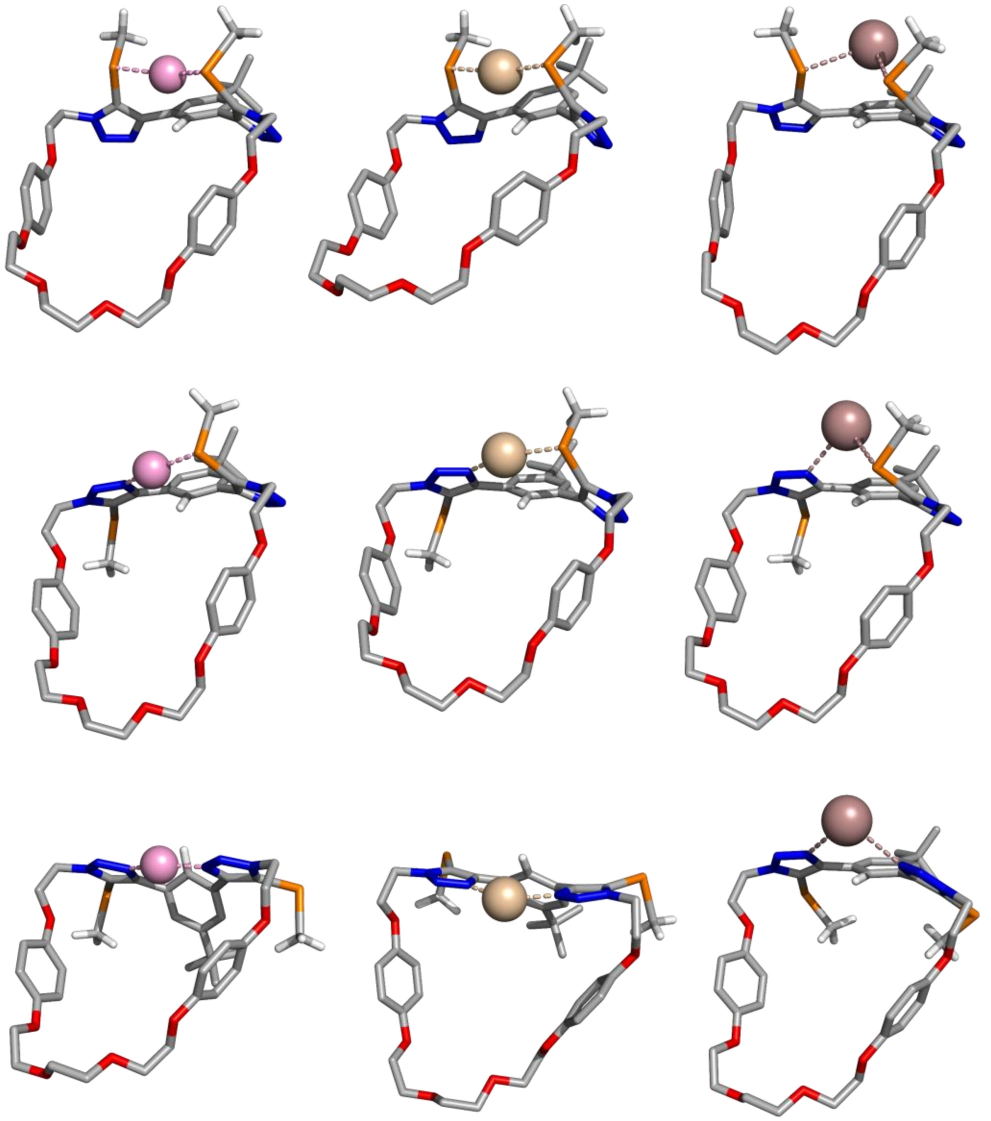

Fig. 109 | PBE0-D3(BJ)/def2-TZVP/acetonitrile–SMD optimised structures of Cu(I) (left), Ag(I) (centre), and Tl(I) (right) cations complexes of **3·Te^{Me}**, in the *endo*/*endo* (top), *endo*/*exo* (middle) and *exo*/*exo* (bottom) binding geometries. The bonding interactions are represented as dashed lines, regardless of their nature.

Table 3 | Energy differences (ΔE_{conf})^{a} between the *endo*/*exo* or *exo*/*exo* conformations of free or complexed **3·Te^{Me}** and the *endo*/*endo* conformation (kcal mol^{-1}).

Solvent Model	Free		Cu(I)		Ag(I)		Tl(I)	
	endo/*exo*	*exo*/*exo*	*endo*/*exo*	*exo*/*exo*	*endo*/*exo*	*exo*/*exo*	*endo*/*exo*	*exo*/*exo*
SMD	0.7	1.2	2.9	15.7	7.6	22.2	3.6	4.3
PCM	0.0	0.1	0.2	10.1	4.7	16.3	1.9	1.3

$^{a)}$ Values were calculated using thermal corrected energies (E), which were obtained as $E = \mathcal{E}_0 + E_{Tot}$, from the uncorrected electronic energies ($\mathcal{E}_0$) and the internal energy corrections (E_{Tot}) due to translational, rotational, vibrational, and electronic motions).

Given that the source for Cu(I) was tetrakis(acetonitrile)copper(I) hexafluorophosphate and the complexation occurs in acetonitrile media, the trigonal coordination mode of *endo*/*endo* **3·Te^{Me}** was also considered, with the third position of the Cu(I) coordination sphere occupied by an acetonitrile molecule. The structures optimised in the SMD (Fig. 110) and PCM solvation models are similar, with a RMSD value of 0.23 Å, and have Te⋯Cu(I)⋯Te and Te⋯Cu(I)⋯N angles close to 120° (Table 4). The Cu(I)⋯Te distances are slightly longer than those observed in the linear geometry (Table 5).

This trigonal binding geometry is akin to the one reported for the Cu(I)·**8·ChB3'** complex, with the acetonitrile molecule being replaced by the axle's TeMe binding unit.

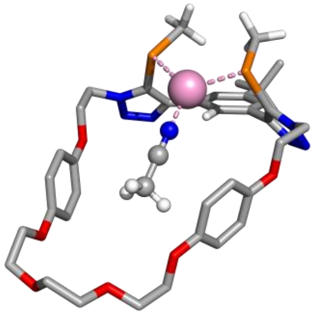

Fig. 110 | PBE0-D3(BJ)/def2-TZVP/acetonitrile-SMD optimised structure of the Cu(I) complex of **3·Te^{Me}**, in the *endo*/*endo* binding geometry, in the presence of a coordinated acetonitrile solvent molecule.

Table 4 | Selected bond distances and angles (distances in Å and angles in °) of the M⋯Te/N interactions in the Cu(I) cation complexes of **3·Te^{Me}**, in two solvation models.

Solvation model	Tellurium/ Acetonitrile	Bonding dimensions			
		M⋯Te/N	C_{trz}-Te⋯M	C_{Me}-Te⋯M	Te⋯M⋯Te/N^{a}
SMD	Te1	2.56	100.0	106.6	119.9; 123.5; 116.4
	Te2	2.59	97.6	107.3	
	N_{ACN}	1.95	-	-	
PCM	Te1	2.54	96.4	104.8	124.2; 119.4; 116.1
	Te2	2.55	95.2	105.5	
	N_{ACN}	1.94	-	-	

$^{a)}$ The angles subtended at the metal centre are given in the following order: Te1⋯M⋯Te2, Te1⋯M⋯N_{ACN}, and Te2⋯M⋯N_{ACN}.

Implicit solvent models benchmark for cation coordination studies

Preliminary DFT geometry calculations on **3·Te^{Me}** and its cation complexes were carried out using the SMD[39] and PCM[40] solvent models to alternatively describe the acetonitrile solvent effects. The energy differences (ΔE_{conf}) between the *endo*/*exo* or *exo*/*exo* conformations of the free or complexed macrocycles and the *endo*/*endo* lowest energy conformation are gathered in Table 3. Selected bond distances and angles calculated for the optimised geometries of Cu(I), Ag(I), and Tl(I) complexes with the macrocycle alternatively adopting the *endo*/*endo*, *endo*/*exo*, and *exo*/*exo* chelating conformations (Fig. 109) are summarised in Table 5–7. Overall, regardless of the metal cation and binding coordination mode, the average coordination distances are almost independent of the solvent model, except for the Tl(I)···N distances in the *exo*/*exo* binding arrangement, where the differences between SMD and the PCM optimised structures are *ca*. 0.16 Å. The impact of the solvent models on the angles involving the metal centres is marginal, with the larger difference between the SMD and the PCM optimised geometries being only *ca*. 7° for the Te···Cu(I)···Te angle in the *endo*/*endo* Cu(I) complex of **3·Te^{Me}**. On the other hand, the interaction energies (ΔE, estimated as given above; Table 8), with **3·Te^{Me}** adopting the same conformation in both the complexed and free states, are largely dependent on the solvent model adopted. The PCM and SMD solvation models' results yield opposite interaction energy trends for the linear Cu(I) and Ag(I) complexes. Given that the results obtained with the SMD solvation model agree with the experimental ones, and that SMD has been largely used in DFT studies with transition[41–43] and post-transition[44,45] metal complexes, as well as in compounds with tellurium[46] or iodine,[47] this solvent model was adopted in the subsequent calculations.

Table 5 | Selected bond distances and angles (distances in Å and angles in °) in two alternative implicit solvent models of acetonitrile, for the *endo*/*endo* cation complexes of **3·Te^{Me}**.

Cation	Te donors	SMD				PCM			
		M···Te	C_{trz}-Te···M	C_{Me}-Te···M	Te···M···Te	M···Te	C_{trz}-Te···M	C_{Me}-Te···M	Te···M···Te
Cu(I)	Te1	2.53	94.8	101.5	146.7	2.54	92.1	99.4	139.1
	Te2	2.53	94.1	100.9		2.54	90.6	100.2	
Ag(I)	Te1	2.67	96.1	98.3	165.9	2.70	96.0	99.0	165.5
	Te2	2.67	96.8	98.3		2.70	96.3	98.6	
Tl(I)	Te1	3.46	92.0	83.9	75.0	3.49	91.4	89.4	76.4
	Te2	3.44	95.3	87.7		3.46	93.2	89.8	
	C_{ar}	3.18; 3.92	-	-	-	3.11; 3.70	-	-	-

Table 6 | Selected bond distances and angles (distances in Å and angles in °) in two alternative implicit solvent models of acetonitrile, for the *endo*/*exo* cation complexes of **3·Te^{Me}**.

Cation	Te/N donors	SMD M···Te/N	SMD C_{trz}-Te···M	SMD C_{Me}-Te···M	SMD Te/N···M···Te/N	PCM M···Te/N	PCM C_{trz}-Te···M	PCM C_{Me}-Te···M	PCM Te/N···M···Te/N
Cu(I)	Te1	2.51	101.0	98.4	166.2	2.49	100.1	99.8	171.1
	N2	1.98	-	-		1.95	-	-	
Ag(I)	Te1	2.65	101.0	98.0	158.9	2.67	102.4	98.5	157.4
	N2	2.20	-	-		2.21	-	-	
Tl(I)	Te1	3.43	104.4	84.8	95.0	3.47	102.4	88.1	98.5
	N2	2.88	-	-		2.74	-	-	

Table 7 | Selected bond distances and angles (distances in Å and angles in °) in two alternative implicit solvent models of acetonitrile, for the *exo*/*exo* cation complexes of **3·Te^{Me}**.

Cation	N donors	SMD M···N	SMD N···M···N	PCM M···N	PCM N···M···N
Cu(I)	N1	2.03	148.2	1.98	153.3
	N2	2.02		1.98	
Ag(I)	N1	2.29	137.2	2.31	136.2
	N2	2.30		2.31	
Tl(I)	N1	3.05	105.0	2.92	109.2
	N2	3.08		2.92	

Table 8 | Uncorrected electronic binding energies ($\Delta\mathcal{E}_0$),[a] Zero Point Corrections (ΔZPE),[b] and thermal corrected binding energies (ΔE)[c] (in kcal mol^{-1}) for the cation complexes of **3·Te^{Me}**.[d]

Solvent Model	Energy term	Cu(I) *endo*/*endo*	Cu(I) *endo*/*exo*	Cu(I) *exo*/*exo*	Ag(I) *endo*/*endo*	Ag(I) *endo*/*exo*	Ag(I) *exo*/*exo*	Tl(I) *endo*/*endo*	Tl(I) *endo*/*exo*	Tl(I) *exo*/*exo*
SMD	$\Delta\mathcal{E}_0$	-39.5	-37.8	-25.7	-46.5	-39.9	-25.7	-12.3	-9.5	-9.5
	ΔZPE	0.7	1.5	1.8	0.9	1.3	1.4	0.2	0.3	0.6
	ΔE	-38.4	-36.2	-23.9	-45.2	-38.3	-24.2	-11.3	-8.4	-8.2
PCM	$\Delta\mathcal{E}_0$	-62.8	-62.6	-53.0	-39.1	-34.5	-23.0	-17.9	-15.9	-16.6
	ΔZPE	1.1	1.3	1.8	0.9	1.1	1.4	0.6	0.5	0.5
	ΔE	-61.4	-61.1	-51.4	-37.7	-33.0	-21.5	-16.7	-14.7	-15.4

[a)] The energy values were not corrected for basis set superposition errors; [b)] ΔZPE is included in the ΔE term; [c)] $\Delta E = \Delta\mathcal{E}_0 + \Delta E_{Tot}$, where ΔE_{Tot} accounts for the differences in the internal energy due to translational, rotational, vibrational and electronic motions; [d)] the absolute energy terms are defined in the Quantum calculations section.

Metal complexes of 8·ChB3'

Table 9 | Selected bond distances and angles (distances in Å and angles in °), together with NBO analysis (E^2, kcal mol^{-1}) of the M···Te interactions in the Cu(I), Ag(I), and Tl(I) cation complexes of **8·ChB$_3$'**.

Metal	Component	Chalcogen	Bonding dimensions M···Te	Bonding dimensions C_{trz}-Te···M	Bonding dimensions C_{Me}-Te···M	Bonding dimensions Te···M···Te[a]	NBO analysis Interaction	NBO analysis E^2
Cu(I)	Macrocycle	Te1	2.58	95.2	106.9	121.7;	$n_{Te} \rightarrow LV_{Cu+}$	65.6
		Te2	2.56	98.5	103.8	108.7;	$n_{Te} \rightarrow LV_{Cu+}$	72.5
	Axle	Te3	2.59	117.1	95.5	120.8	$n_{Te} \rightarrow LV_{Cu+}$	68.5
	Average		*2.58*	*103.6*	*102.1*	*117.1*	*Average*	*68.9*
Ag(I)	Macrocycle	Te1	2.75	94.9	108.2	118.0;	$n_{Te} \rightarrow LV_{Ag+}$	77.4
		Te2	2.74	100.7	103.4	115.7;	$n_{Te} \rightarrow LV_{Ag+}$	82.8
	Axle	Te3	2.73	111.4	95.5	121.2	$n_{Te} \rightarrow LV_{Ag+}$	90.7
	Average		*2.74*	*102.3*	*102.3*	*118.3*	*Average*	*83.7*
Tl(I)	Macrocycle	Te1	3.66	92.7	112.3	166.9;	$n_{Te} \rightarrow LV_{Tl+}$	19.1
		Te2	3.83	85.2	127.4	66.0;	$n_{Te} \rightarrow LV_{Tl+}$	18.4
	Axle	Te3	3.59	120.7	76.1	117.1	$n_{Te} \rightarrow LV_{Tl+}$	19.7
	Average		*3.69*	*99.6*	*105.2*	*116.6*	*Average*	*19.1*
	C_{ar}	-	3.31; 3.39	-	-	-	$\Omega_{C\equiv C} \rightarrow LV_{Tl+}$	14.8
	Average		3.34				*Average*	2.5

[a)] The angles subtended at the metal centre are given in the following order: Te1···M···Te2, Te1···M···Te3, and Te2···M···Te3.

Detailed NBO analysis of the metal complexes of **3·Te^{Me}**

Table 10 summarises the E^2 interaction energies assessed from the *endo*/*endo* binding geometries of the **3·Te^{Me}** metal complexes DFT optimised in acetonitrile (SMD) at the PBE0-D3(BJ)/def2-TZVP theory level. These energies are the individual contributions from the interactions between the metals' Lone Vacancies orbitals (LV_{M+}) and the tellurium donors Lone Pair orbitals (n_{Te}), as well as the interactions between Tl(I) and the C≕C aromatic bonds' orbitals ($\Omega_{C≕C}$). It is worth noting that the metal complexes of **3·Te^{Me}**, with only two M···Te endotopic interactions, displays higher average E^2 stabilisation energies (74.7 kcal mol^{-1} for Cu(I) and 111.0 kcal mol^{-1} for Ag(I)) than the Cu(I) and Ag(I) complexes of **8·ChB$_3$**' (68.9 kcal mol^{-1} for Cu(I) and 83.7 kcal mol^{-1} for Ag(I)). In addition, the *endo*/*endo* Tl(I) complex of **3·Te^{Me}** is stabilised by M···Te interactions worth 30.7 kcal mol^{-1}, while the cation···π interactions amount to an E^2 contribution of 8.1 kcal mol^{-1}.

The E^2 stabilisation energies of Cu(I)·**3·Te^{Me}** coordinated to an acetonitrile molecule (trigonal binding mode) are also given in Table 10, with the Cu(I)···Te interactions being weaker than in the linear binding arrangement. Indeed, the average E^2 value (65.7 kcal mol^{-1}) is much closer to the average value computed for the **8·ChB$_3$'** complex (68.9 kcal mol^{-1}), also with a trigonal coordination geometry.

Table 10 | NBO Second-order perturbation theory energies (E^2, kcal mol^{-1}) for the cation metal complexes of **3·Te^{Me}**, in the SMD solvation model.

Metal	Binding mode	Interaction	E^2
Cu(I)	Linear geometry	$n_{Te} \rightarrow LV_{Cu+}$	75.0
		$n_{Te} \rightarrow LV_{Cu+}$	74.5
	Trigonal geometry	$n_{Te} \rightarrow LV_{Cu+}$	68.2
		$n_{Te} \rightarrow LV_{Cu+}$	63.2
Ag(I)	Linear geometry	$n_{Te} \rightarrow LV_{Ag+}$	111.0
		$n_{Te} \rightarrow LV_{Ag+}$	110.9
Tl(I)	Linear geometry	$n_{Te} \rightarrow LV_{Tl+}$	14.8
		$n_{Te} \rightarrow LV_{Tl+}$	15.9
		$\Omega_{C\cdots C} \rightarrow LV_{Tl+}$	8.1

Metal complexes of XB-based receptors 8·XB$_3$' and 3·I

Following the experimental evidence hinting at the possibility of the C$_{trz}$-I binding sites of **8·XB$_3$'**, **3·I** and **11·I'** to function as Lewis bases, the metal cation binding ability of these receptors was investigated with the iodine binding units disposed in a convergent mode, with the macrocycle adopting an *endo*/*endo* conformation. The DFT optimised structures of the Cu(I), Ag(I), and Tl(I) complexes of **8·XB$_3$'** are shown in Fig. 112. The Cu(I) and Ag(I) complexes display distorted trigonal coordination environments, with the distances and angles around the metal centres listed in Table 11. The Ag·**8·XB$_3$'** complex displays an average Ag(I)···I distance of 2.83 Å, being naturally longer than the average Cu(I)···I distances of 2.66 Å. The average E^2 energy for the interactions between the lone pair orbitals (n_I) from the C$_{trz}$-I binding sites and the LV$_{Ag+}$ orbitals is 41 kcal mol^{-1}, while the Cu(I)···I interactions lead to a smaller average E^2 value of 38 kcal mol^{-1}.

In the DFT optimised structure of Tl·**8·XB$_3$**' (Fig. 112), like in **8·ChB$_3$'**, the metal centre between the iodine macrocyclic binding units also establishes cation···π interactions, with Tl(I)···C$_{ar}$ distances between 3.30 and 3.43 Å to the *tert*-butyl phenyl. These features result in an average Tl(I)···I distance of 3.86 Å. The cooperative sharing of Tl(I) between the three iodine binding units of **8·XB$_3$'** results in average E^2 energies of 14 kcal mol^{-1} (Table 11). The cation···π interactions afford a total E^2 value of 11.2 kcal mol^{-1}. In the DFT optimised structure of Tl·**3·I** (Fig. 111) the Tl(I) cation interactions with the iodine binding centres afford an average E^2 energy of 9 kcal mol^{-1}, while the cation···π interactions total 6.6 kcal mol^{-1} (Table 12). The E^2 energy values for the Tl(I)···I interactions are significantly smaller than those for Tl(I)···Te in the **8·ChB$_3$'** and **3·Te^{Me}** complexes, in agreement with the weaker experimentally observed coordination ability of **8·XB$_3$** and **3·I** (Research Article Table 2).

The impact of the SMD18 solvation model (see above) on the M···I interactions was also evaluated,[37] with the reoptimization of **8·XB$_3$'** metal complexes with iodine's larger radius. The structures of the Ag(I) and Cu(I) complexes computed with SMD18 have M···I distances comparable to those calculated in SMD (Table 11). In contrast, the Tl·**8·XB$_3$'** and Tl·**3·I** SMD18 optimised structures display Tl(I)···I distances ca. 0.4 and 0.8 Å, respectively, larger than those estimated in SMD. The C_{trz}-I···M and I···M···I angles in both solvent models are comparable. The E^2 values for the Ag·**8·XB$_3$'** complex are also comparable in both versions of SMD, whilst for the Cu(I) complex, the average E^2 value is higher in SMD18. On the other hand, the longer Tl(I)···I distances in SMD18 yielded lower E^2 values in Tl·**8·XB$_3$'** (Table 11) and Tl·**3·I** (Table 12).

Regardless of the SMD solvation model, the E^2 values for the M···I interactions pale in comparison to the M···Te values assessed in the M·**8·ChB$_3$'** complexes, namely with Ag(I) and Cu(I) (Table 9). Therefore, the discussion presented in the Main Text is based on the SMD solvation model results.

Table 11 | Selected distances (Å) and angles (°), together with NBO analysis (E^2, kcal mol^{-1}), of the M···I interactions in the Cu(I), Ag(I) and Tl(I) cation complexes of **8·XB$_3$'**, in the SMD and SMD18 solvation models.

Solvent	Metal	Component	Halogen	Dimensions			NBO analysis	
				M···I	C_{trz}-I···M	I···M···I[a]	Interaction	E^2
SMD	Cu(I)	Macrocycle	I1	2.69	100.6	101.5;	$n_I \rightarrow LV_{Cu+}$	34.0
			I2	2.71	102.2	116.7;	$n_I \rightarrow LV_{Cu+}$	35.3
		Axle	I3	2.60	108.2	134.5	$n_I \rightarrow LV_{Cu+}$	43.2
		Average		*2.66*	*103.7*	*117.6*	*Average*	*37.5*
	Ag(I)	Macrocycle	I1	2.93	100.5	97.5;	$n_I \rightarrow LV_{Ag+}$	31.2
			I2	2.82	99.3	113.4;	$n_I \rightarrow LV_{Ag+}$	41.6
		Axle	I3	2.74	105.6	140.2	$n_I \rightarrow LV_{Ag+}$	50.3
		Average		*2.83*	*101.8*	*117.0*	*Average*	*41.0*
	Tl(I)	Macrocycle	I1	3.87	90.6	161.9;	$n_I \rightarrow LV_{Tl+}$	13.7
			I2	3.86	86.3	66.7;	$n_I \rightarrow LV_{Tl+}$	13.7
		Axle	I3	3.84	141.1	113.8	$n_I \rightarrow LV_{Tl+}$	14.5
		Average		*3.86*	*106.0*	*114.1*	*Average*	*14.0*
		C_{ar}	-	3.30; 3.43			$\Omega_{C=C} \rightarrow LV_{Tl+}$	11.2
		Average		3.36			*Average*	1.9
SMD18	Cu(I)	Macrocycle	I1	2.78	99.1	97.4;	$n_I \rightarrow LV_{Cu+}$	36.1
			I2	2.68	98.7	108.2;	$n_I \rightarrow LV_{Cu+}$	44.4
		Axle	I3	2.60	106.2	137.6	$n_I \rightarrow LV_{Cu+}$	54.3
		Average		*2.69*	*101.3*	*114.4*	*Average*	*44.9*
	Ag(I)	Macrocycle	I1	2.97	102.0	95.8;	$n_I \rightarrow LV_{Ag+}$	31.7
			I2	2.87	100.7	113.1;	$n_I \rightarrow LV_{Ag+}$	40.9
		Axle	I3	2.76	104.2	146.0	$n_I \rightarrow LV_{Ag+}$	53.1
		Average		*2.87*	*102.3*	*118.3*	*Average*	*41.9*
	Tl(I)	Macrocycle	I1	4.30	88.7	151.9;	$n_I \rightarrow LV_{Tl+}$	8.9
			I2	4.23	84.4	64.1;	$n_I \rightarrow LV_{Tl+}$	9.0
		Axle	I3	4.26	139.1	109.7	$n_I \rightarrow LV_{Tl+}$	9.6
		Average		*4.26*	*104.1*	*108.6*	*Average*	*9.2*
		C_{ar}	-	3.32; 3.62			$\Omega_{C=C} \rightarrow LV_{Tl+}$	9.0
		Average		3.47			*Average*	1.5

[a] The angles subtended at the metal centre are given in the following order: I1···M···I2, I1···M···I3, and I2···M···I3.

Table 12 | Selected bond distances and angles (distances in Å and angles in °), together with NBO analysis (E^2, kcal mol^{-1}), for the Tl(I)···I interactions, in the Tl(I) complexes of **3·I**, in the SMD and SMD18 solvation models.

Solvent	Halogen	Bonding dimensions			NBO analysis	
		Tl(I)···I	C_{trz}-I···Tl(I)	I···Tl(I)···I	Interaction	E^2
SMD	I1	3.73	90.7	67.4	$n_I \rightarrow LV_{Tl+}$	8.9
	I2	3.84	89.3		$n_I \rightarrow LV_{Tl+}$	8.4
	C_{ar}	3.26; 3.75	-	-	$\Omega_{C=C} \rightarrow LV_{Tl+}$	6.6
SMD18	I1	4.47	83.0	55.7	$n_I \rightarrow LV_{Tl+}$	5.1
	I2	4.61	79.4		$n_I \rightarrow LV_{Tl+}$	4.5
	C_{ar}	3.32; 3.41			$\Omega_{C=C} \rightarrow LV_{Tl+}$	6.4

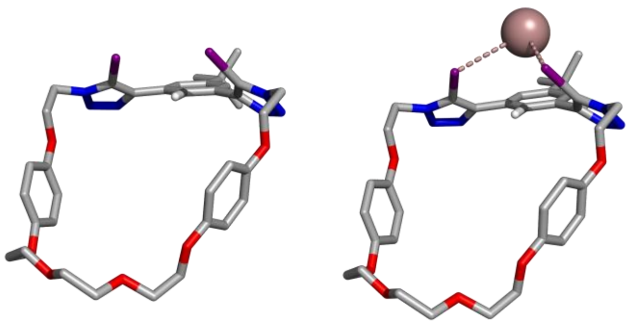

Fig. 111 | PBE0-D3(BJ)/def2-TZVP/acetonitrile-SMD optimised structures of free **3·I** (left) and Tl(I)·**3·I** (right), in the *endo*/*endo* binding geometry. The bonding interactions are represented as dashed lines, regardless of their nature.

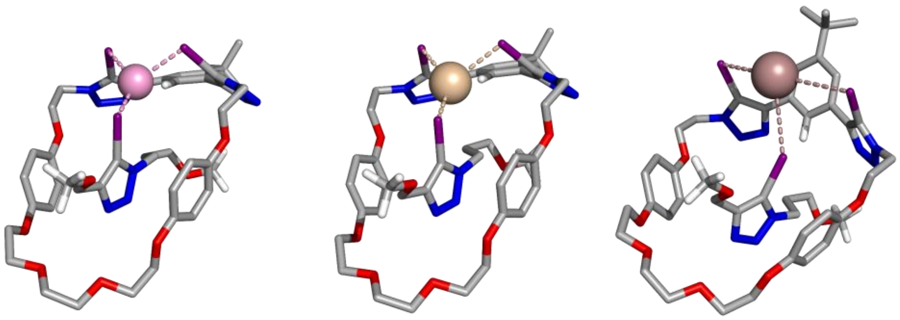

Fig. 112 | PBE0-D3(BJ)/def2-TZVP/acetonitrile-SMD optimised structures of **8·XB$_3$'** metal complexes of Cu(I) (left), Ag(I) (centre), and Tl(I) (right), in the *endo*/*endo* binding geometry. The bonding interactions are represented as dashed lines, regardless of their nature.

Metal complexes of XB-based receptor 11·I'

Likewise for Cu(I)·**3·Te^{Me}**, the binding modes with Cu(I) endotopically coordinated to **11·I'** in a linear or trigonal coordination fashion were evaluated by DFT calculations undertaken in dichloromethane and acetone. These binding modes are illustrated in Fig. 113 with the optimised structures in the SMD solvation model of acetone, together with the free macrocycle model. The Cu(I)·**11·I'** complex presents two equal strength Cu(I)···I interactions with an average distance of 2.60 Å and an I···Cu(I)···I angle of 146.6°. The optimised geometry in the less polar dichloromethane displays similar coordination parameter values (Table 13). Despite the dielectric constants' difference between solvents, the RMSD between computed structures is only 0.14 Å. In the computed structures for the Cu(I) trigonal complex of **11·I'**, the two Cu(I)···I synergetic interactions are similar, with average distances of 2.68 Å in both solvents

and being longer than in the linear geometry. Accordingly, the average E^2 values for the Cu(I)···I interactions decrease, showing how the Lewis binding ability of **11·I’** binding iodine sites is affected by the acetonitrile ligand (Table 13).

The optimised geometries in SMD18 present longer Cu(I)···I interactions. The RMSD values to the SMD optimised structures are 0.21 Å (acetone) and 0.24 Å (dichloromethane) in the linear geometry, and 0.11 Å (acetone) and 0.37 Å (dichloromethane) in the Cu(I)·**11·I’** trigonal complex.

Noteworthy, the E^2 values for the **3·Te^Me** complexes are higher than for **11·I’**, regardless of the solvation models and different solvent media with different dielectric constants (dichloromethane = 8.93 and acetone = 20.49 vs. acetonitrile = 35.69), and coordination geometry (Table 10 versus Table 13**Error! Reference source not found.**). In other words, despite the XB-based binding units having a lower Lewis binding ability than the ChB-based ones, **11·I’** should be able to endotopically recognise Cu(I) in solution, as suggested by the experimental spectroscopic data presented below.

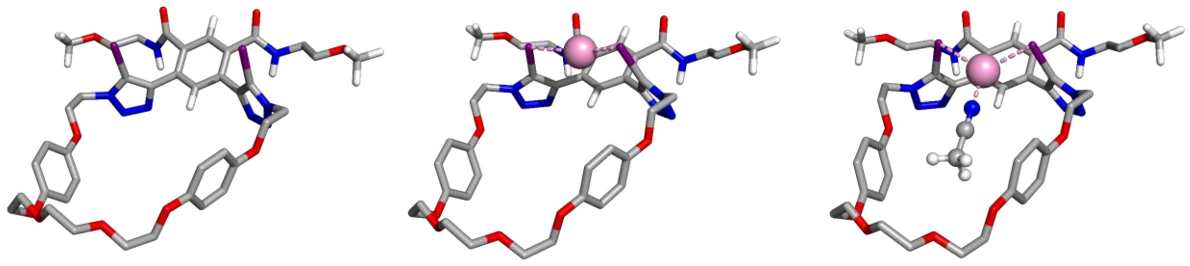

Fig. 113 | PBE0-D3(BJ)/def2-TZVP/acetone-SMD optimised structure of free **11·I’** (left), Cu(I)**·11·I’** in a linear geometry (centre) and Cu(I)**·11·I’** in a trigonal geometry with an acetonitrile molecule (right).

Table 13 | Selected bond distances and angles (distances in Å and angles in °), together with NBO analysis (E^2, kcal mol^{-1}) of the M···I/N interactions in the Cu(I) cation complexes of **11·I'**, in two binding modes and two solvation models.

Binding mode	Solvation model	Solvent	Iodine/ Acetonitrile	Bonding dimensions			NBO analysis	
				M···I/N	C_{trz}-I···M	I···M···I/N[a]	Interaction	E^2
Linear	SMD	Dichloromethane	I1	2.61	92.1	146.6	$n_I \rightarrow LV_{Cu+}$	41.3
			I2	2.59	92.9		$n_I \rightarrow LV_{Cu+}$	42.6
		Acetone	I1	2.61	92.8	150.4	$n_I \rightarrow LV_{Cu+}$	39.2
			I2	2.62	94.3		$n_I \rightarrow LV_{Cu+}$	38.8
	SMD18	Dichloromethane	I1	2.67	99.1	171.3	$n_I \rightarrow LV_{Cu+}$	37.6
			I2	2.68	99.4		$n_I \rightarrow LV_{Cu+}$	36.9
		Acetone	I1	2.68	98.3	171.3	$n_I \rightarrow LV_{Cu+}$	33.3
			I2	2.73	99.0		$n_I \rightarrow LV_{Cu+}$	31.4
Trigonal	SMD	Dichloromethane	I1	2.65	91.9	119.3;	$n_I \rightarrow LV_{Cu+}$	42.3
			I2	2.71	89.6	129.2;	$n_I \rightarrow LV_{Cu+}$	33.7
			N_{ACN}	1.93	-	110.7	-	-
		Acetone	I1	2.64	90.6	122.9;	$n_I \rightarrow LV_{Cu+}$	40.1
			I2	2.72	89.7	125.3;	$n_I \rightarrow LV_{Cu+}$	32.2
			N_{ACN}	1.94	-	110.6	-	-
	SMD18	Dichloromethane	I1	2.69	94.2	107.8;	$n_I \rightarrow LV_{Cu+}$	43.4
			I2	2.71	91.9	132.8;	$n_I \rightarrow LV_{Cu+}$	34.4
			N_{ACN}	1.92	-	119.5	-	-
		Acetone	I1	2.68	91.2	114.4;	$n_I \rightarrow LV_{Cu+}$	42.1
			I2	2.83	91.7	134.0;	$n_I \rightarrow LV_{Cu+}$	29.1
			N_{ACN}	1.92	-	111.6	-	-

[a] The angles subtended at the metal centre are given in the following order: I1···M···I2, I1···M···N_{ACN}, and I2···M···N_{ACN}.

Distribution of electrostatic potential on ChB and XB triazole-based binding units

MEP of the 1,4-dimethyl-5-(methyltellanyl)-1*H*-1,2,3-triazole motif displays two V_S values of -6.6 and -8.7 kcal mol^{-1}, positioned almost perpendicular to the C_{trz}-Te and C_{Me}-Te bonds (see Fig. 114). The 5-iodo-1,4-dimethyl-1*H*-1,2,3-triazole motif also presents two perpendicular V_S points of -1.0 kcal mol^{-1}.

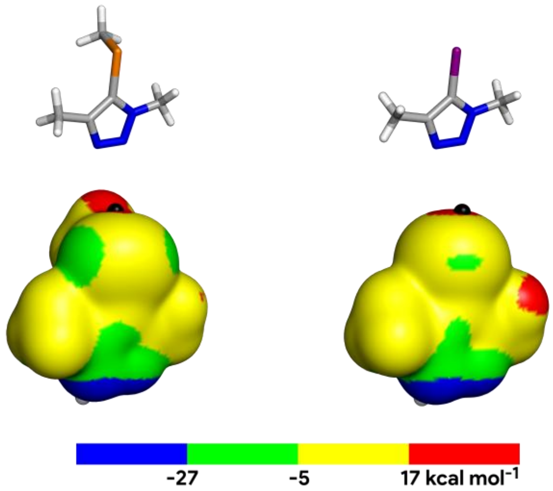

Fig. 114 | DFT optimised structures of 1,4-dimethyl-5-(methyltellanyl)-1*H*-1,2,3-triazole (left) and 5-iodo-1,4-dimethyl-1*H*-1,2,3-triazole (right) fragments in the gas-phase, at the PBE0-D3(BJ)/def2-TZVP theory level, together with the corresponding MEPs (0.001 e $Bohr^{-3}$). The most positive and most negative values per triazole binding motif are identified with black and white hemispheres, respectively.

Natural Population Analysis charges and Fuzzy Bond Orders

In addition to the E^2 energies, the charge transfers derived from the Te···X^-, M···I and M···Te interactions were also characterized through the evaluation of the NPA charges of the TeMe and I binding units in **8·ChB$_3$'** and **8·XB$_3$'**, in the free and host-guest complexes. The NPA charges of the binding units were averaged and are presented in Table 14, together with the NPA charge of the metal cation or anion guests. The changes of the NPA charges derived from the Te···X^-, M···Te, and M···I interactions are presented in Fig. 115. The Te···X^- interactions lead to slight variations in the NPA charges on both the anions and TeMe binding units, which behave as Lewis acids, resulting in slightly charge depleted anions. The Me···I and Me···Te interactions lead to a decrease of the metal centres' charges, while the charges of the TeMe and I binding units indicate a more pronounced charge redistribution on the host upon metal coordination. The NPA charges of the complexed metals are inversely correlated with the corresponding E^2 values ($R^2 \geq 0.95$).

Table 14 | NPA charges per binding unit and average NPA charges assessed in the DFT optimised structures of free and complexed **8·ChB$_3$'** and **8·XB$_3$'**, together with the NPA charges* of the guest ions.

Theory level	Host	Guest	Te1/I1	Te2/I2	Te3/I3	Average NPA	X^-/M
M06-2X/Def2-TZVP(D)/acetone-CPCM	**8·ChB$_3$'**	Free	0.51	0.52	0.51	0.51	-
		Cl^-	0.56	0.55	0.57	0.56	-0.91
		Br^-	0.55	0.55	0.56	0.55	-0.91
		I^-	0.54	0.54	0.55	0.54	-0.90
M06-2X/Def2-TZVP(D)/acetone-SMD18	**8·ChB$_3$'**	Free	0.53	0.53	0.54	0.53	-
		Cl^-	0.56	0.56	0.57	0.56	-0.91
		Br^-	0.56	0.55	0.56	0.56	-0.91
		I^-	0.55	0.54	0.55	0.55	-0.90
PBE0-D3(BJ)/Def2-TZVP/acetonitrile-SMD	**8·ChB$_3$'**	Free	0.51	0.51	0.51	0.51	-
		Cu(I)	0.59	0.60	0.59	0.59	0.57
		Ag(I)	0.60	0.61	0.63	0.61	0.50
		Tl(I)	0.54	0.54	0.55	0.55	0.74
PBE0-D3(BJ)/Def2-TZVP/acetonitrile-SMD	**8·XB$_3$'**	Free	0.27	0.26	0.24	0.26	-
		Cu(I)	0.31	0.31	0.31	0.31	0.74
		Ag(I)	0.31	0.33	0.33	0.32	0.69
		Tl(I)	0.29	0.29	0.27	0.29	0.84
PBE0-D3(BJ)/Def2-TZVP/acetonitrile-SMD18	**8·XB$_3$'**	Free	0.23	0.23	0.22	0.23	-
		Cu(I)	0.26	0.28	0.30	0.28	0.66
		Ag(I)	0.26	0.28	0.30	0.28	0.66
		Tl(I)	0.25	0.25	0.24	0.25	0.89

* As multiples of *e*.

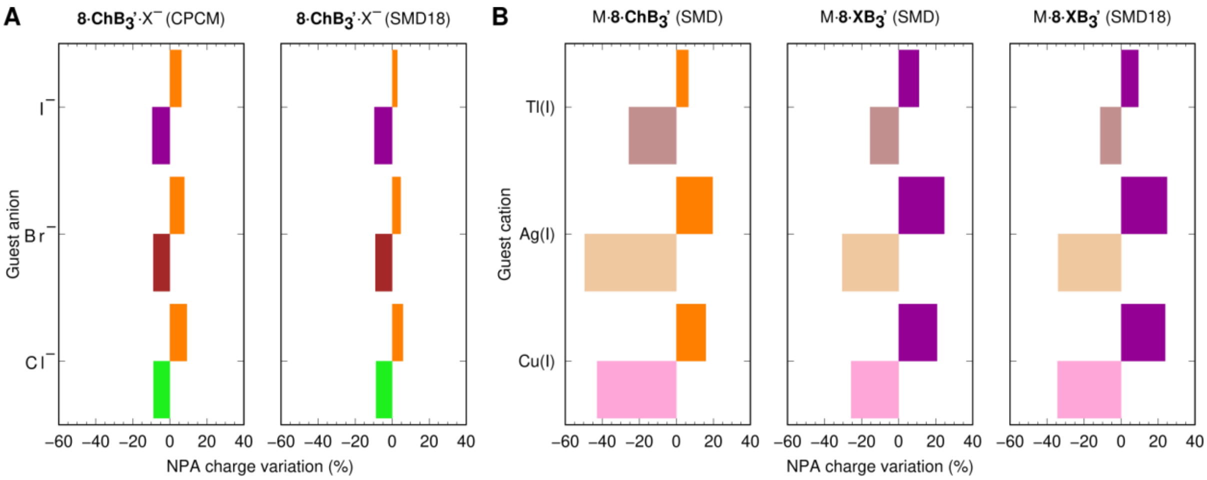

Fig. 115 | A: Variation (%) of the average NPA charges on the binding units of **8·ChB$_3$'** (orange bars, CPCM or SMD18 solvation models), together with the variation on the guest anions (Cl^-, green bars; Br^-, brown bars; and I^- purple bars); **B**: Variation (%) of the average NPA charges on the binding units of **8·ChB$_3$'** (orange bars, SMD solvation model) or **8·XB$_3$'** (purple bars, SMD or SMD18 solvation models), together with the variation on the guest cations (Cu(I), lavender pink bars; Ag(I), desert sand bars; and Tl(I), rosy brown bars). The variation of the NPA charges was calculated as $NPA_{variation}(\%) = \frac{NPA_{complex} - NPA_{free}}{NPA_{free}} \times 100$, where $NPA_{complex}$ and NPA_{free} respectively correspond to the average NPA charges of the Te/I binding units in the complexes and in the free receptors or to the NPA charges of the ions in the bound and unbound states (-1 for the halides and +1 for the metal cations).

FBO analysis was carried out in the halide associations of **8·ChB₃'** (Research Article Fig. 4b), the metal complexes of **8·ChB₃'** (Research Article Fig. 4c) and in the *endo*/*endo* conformations of the metal complexes of **8·XB₃'** (Fig. 112, left), affording the values listed in Table 15 and discussed in the Main Text.

Table 15 | FBO per binding unit and average FBO for the Te···X⁻ and M···Te (**8·ChB₃'**) and M···I (**8·XB₃'**) interactions in the DFT optimised structures.

Theory level	Host	Guest	Te1/I1	Te2/I2	Te3/I3	Average FBO
M06-2X/Def2-TZVP(D)/acetone-CPCM	**8·ChB₃'**	Cl^-	0.41	0.27	0.31	0.33
		Br^-	0.40	0.33	0.28	0.34
		I^-	0.39	0.35	0.26	0.33
M06-2X/Def2-TZVP(D)/acetone-SMD18	**8·ChB₃'**	Cl^-	0.42	0.28	0.30	0.33
		Br^-	0.41	0.33	0.27	0.34
		I^-	0.38	0.37	0.25	0.34
PBE0-D3(BJ)/Def2-TZVP/acetonitrile-SMD	**8·ChB₃'**	Cu(I)	0.98	1.01	0.98	0.99
		Ag(I)	1.02	1.03	1.06	1.04
		Tl(I)	0.40	0.32	0.41	0.38
PBE0-D3(BJ)/Def2-TZVP/acetonitrile-SMD	**8·XB₃'**	Cu(I)	0.85	0.84	0.99	0.90
		Ag(I)	0.81	0.94	1.06	0.94
		Tl(I)	0.27	0.29	0.25	0.27
PBE0-D3(BJ)/Def2-TZVP/acetonitrile-SMD18	**8·XB₃'**	Cu(I)	0.80	0.91	1.04	0.92
		Ag(I)	0.81	0.92	1.08	0.94
		Tl(I)	0.15	0.18	0.15	0.16

The NPA and FBO analyses were extended to the DFT optimised structures of the free and Cu(I) complexes of **3·Te^{Me}** and **11·I'** (Table 16). In line with the insights for the **8·ChB₃'** and **8·XB₃'**, a more pronounced charge transfer occurs in the Cu(I) complexes of **3·Te^{Me}** than in the complexes of **11·I'**, regardless of the solvation conditions and binding geometry. In agreement, the FBO parameters indicate that the M···Te interactions are also slightly stronger than the M···I ones.

Table 16 | NPA charges per binding unit and average NPA charges assessed in the DFT optimised structures of free and complexed **3·Te^{Me}** and **11·I'**, together with the NPA charges of the guest metal cations, along with the FBO per binding unit and average FBO for the M···Te and M···I interactions in the DFT optimised structures, in the SMD and SMD18 solvation models, in the corresponding implicit solvents.

Theory level	Host	Guest	Binding Geometry	NPA charges*				FBO		
				Te1/I1	Te2/I2	Average	M	Te1/I1	Te2/I2	Average
PBE0-D3(BJ)/Def2-TZVP/ acetonitrile-SMD	**3·Te^{Me}**	Free	-	0.50	0.51	0.50	-	-	-	-
		Cu(I)	Linear	0.62	0.62	0.62	0.64	1.09	1.08	1.08
			Trigonal	0.59	0.59	0.59	0.70	1.03	1.00	1.02
PBE0-D3(BJ)/Def2-TZVP/ acetone-SMD	**11·I'**	Free	-	0.27	0.27	0.27	-	-	-	-
		Cu(I)	Linear	0.33	0.33	0.33	0.81	1.00	1.00	1.00
			Trigonal	0.33	0.31	0.32	0.81	0.95	0.86	0.91
PBE0-D3(BJ)/Def2-TZVP/ dichloromethane-SMD	**11·I'**	Free	-	0.26	0.26	0.26	-	-	-	-
		Cu(I)	Linear	0.33	0.33	0.33	0.79	1.00	1.03	1.02
			Trigonal	0.32	0.31	0.32	0.79	0.95	0.87	0.91
PBE0-D3(BJ)/Def2-TZVP/ acetone-SMD18	**11·I'**	Free	-	0.24	0.24	0.24	-	-	-	-
		Cu(I)	Linear	0.27	0.27	0.27	0.80	0.97	0.92	0.94
			Trigonal	0.29	0.27	0.28	0.77	0.95	0.79	0.87
PBE0-D3(BJ)/Def2-TZVP/ dichloromethane-SMD18	**11·I'**	Free	-	0.24	0.24	0.24	-	-	-	-
		Cu(I)	Linear	0.28	0.28	0.28	0.77	0.99	0.97	0.98
			Trigonal	0.29	0.28	0.28	0.75	0.93	0.90	0.92

* As multiples of *e*.

S6 Further Evidence for Iodine···Cation Interaction

To provide further experimental evidence to elucidate the cation binding modes, we have conducted a further series of ^{1}H NMR titration experiments of [2]rotaxanes **8·XB$_3$/ChB$_3$/HB$_3$** with Cu(I) and Ag(I) cations in the non-competitive CD_2Cl_2, which is less coordinating than CD_3CN utilised in cation association constants determination. This was expected to provide more definitive structural features regarding the rotaxane cation binding conformation in the resulting spectra. A ^{1}H NMR titration of **8·XB$_3$** with $[Cu(CH_3CN)_4]PF_6$ is shown as representative in Fig. 116.

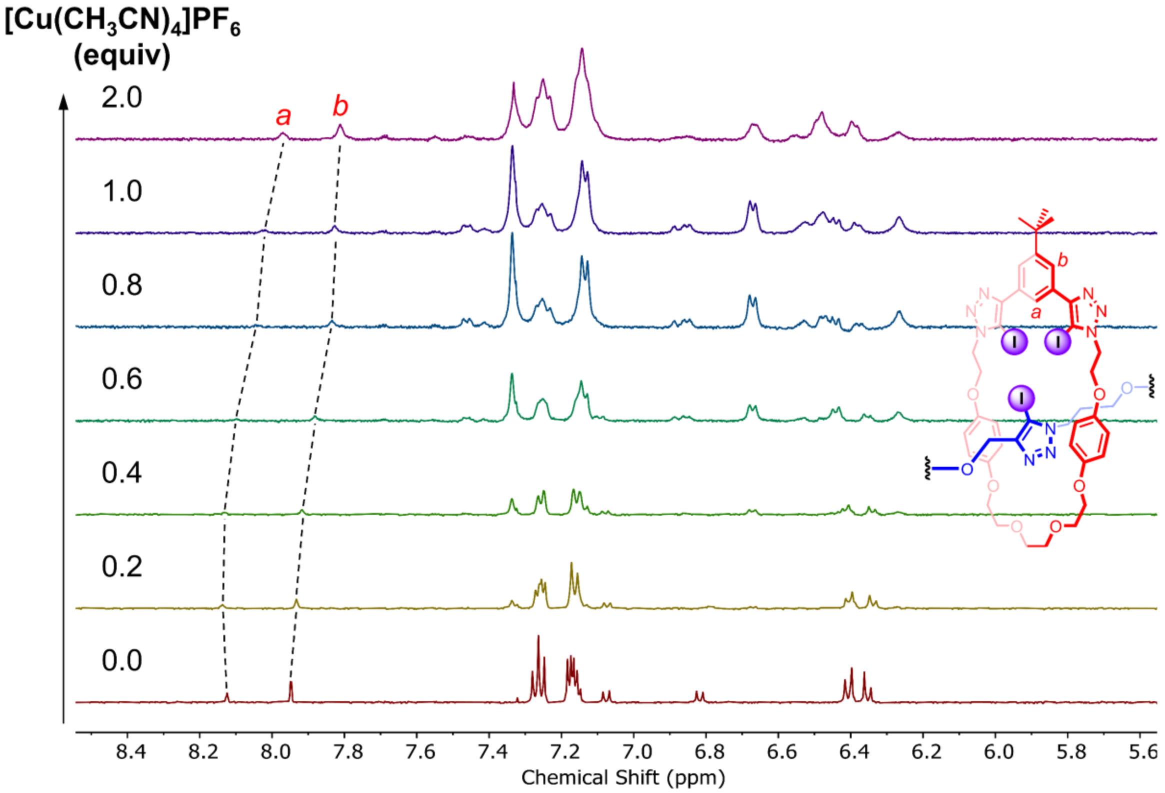

Fig. 116 | ^{1}H NMR titration spectra of XB [2]rotaxane **8·XB$_3$** upon the stepwise addition of up to 2.0 equivalent of $[Cu(CH_3CN)_4]PF_6$ in CD_2Cl_2 ([**8·XB$_3$**] = 1.0 mM, 500 MHz, 298 K).

In all cases, similar to ^{1}H NMR titrations conducted in CD_3CN, significant perturbations to rotaxane cavity protons were observed (Fig. 120–125). Importantly, in the presence of one equivalent of Cu(I) or Ag(I), the symmetry of the ^{1}H NMR spectra is retained for all three rotaxane hosts. For ChB [2]rotaxane **8·ChB$_3$**, this can be explained by the formation of three convergent Te···cation interactions, which is supported by the pronounced shifts to $TeCH_3$ signals. In the case of **8·HB$_3$**, cation binding is mediated by all three triazole nitrogens decorating the binding pocket, which induces significant perturbations to the triazole proton

peaks. However, for XB [2]rotaxane **8·XB$_3$**, the titration spectra observed are consistent with cation binding through either three iodine or three nitrogen donors from the iodo-triazole motifs in a symmetric fashion. Therefore, despite our best efforts, it was still not possible to distinguish between these two binding modes via ^{1}H NMR titration experiments.

Therefore, attention was turned to elucidate the cation binding conformation by UV-Vis absorption spectroscopy. It has been well-documented that direct triazole N-coordination to cations induces significant changes to the triazole absorption peaks at ca. 290 nm due to perturbation of the HOMO-LUMO energy gaps of the heterocycle.[48,49] To investigate the triazole-N contribution to cation binding, UV-Vis absorption titration experiments of **8·XB$_3$/ChB$_3$/HB$_3$** with [Cu(CH_3CN)$_4$]PF_6 were conducted in CH_2Cl_2. As shown in Fig. 117, negligible optical changes were observed with **8·ChB$_3$**, consistent with Cu(I) binding via the three ChB tellurium donor atoms. In contrast, drastic perturbation to triazole absorption peak at 290 nm was observed for **8·HB$_3$**, with a clear isosbestic point at 273 nm, arising from triazole-N mediated cation chelation. Pleasingly, for **8·XB$_3$**, no such perturbation was observed, strongly suggesting cation binding was mediated by the proposed Lewis basic iodine donors, and highlighting the similarity with the Lewis basic tellurium donors metal cation binding mode of **8·ChB$_3$**. This postulated three iodine donor cation binding mode of **8·XB$_3$** was also corroborated by computational DFT calculations.

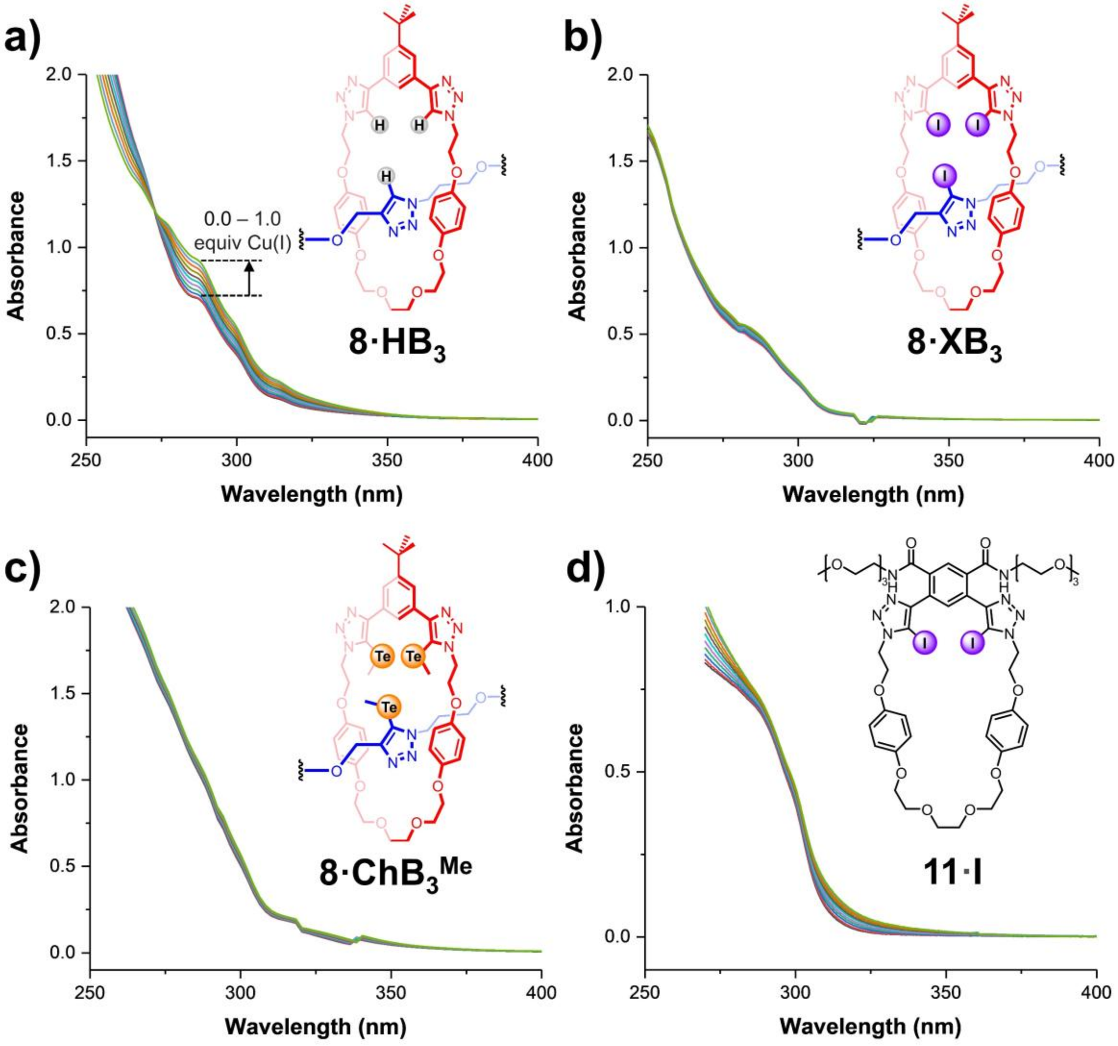

Fig. 117 | UV-Vis absorption titration spectra of [2]rotaxanes a) **8·HB$_3$**, b) **8·XB$_3$**, c) **8·ChB$_3^{Me}$**, and macrocycle d) **11·I** upon the addition of 1.0 equivalent of [Cu(CH_3CN)$_4$]PF_6 ([receptor] = 50 mM, *T* = 298 K, CH_2Cl_2).

To further demonstrate experimentally the involvement of amphoteric iodine in cation association, we designed and synthesised a new XB macrocycle **11·I** (Fig. 126), in which the central phenyl ring is appended with tri(ethylene glycol) (TEG) groups *ortho* to each of the iodo-triazoles. The positioning of the amide-linked TEG chains restricts the free rotation of the iodo-triazole groups by preventing the bulky iodine atoms from passing through, and negates the ability of the iodo-triazole nitrogen to interact with cations in an endotopic coordinating fashion.[6] To confirm this, titration of **11·I** with Cu(I) cation was conducted by ^{1}H NMR in CD_2Cl_2 (Fig. 118) and UV-Vis absorption in CH_2Cl_2 (Fig. 117d). Similar to **8·XB$_3$** and **8·ChB$_3$**, symmetry in ^{1}H NMR spectrum was retained upon the addition of one equivalent of Cu(I), whilst negligible

optical changes to the triazole absorption peaks were observed, providing further experimental evidence for the iodine-mediated cation binding mode.

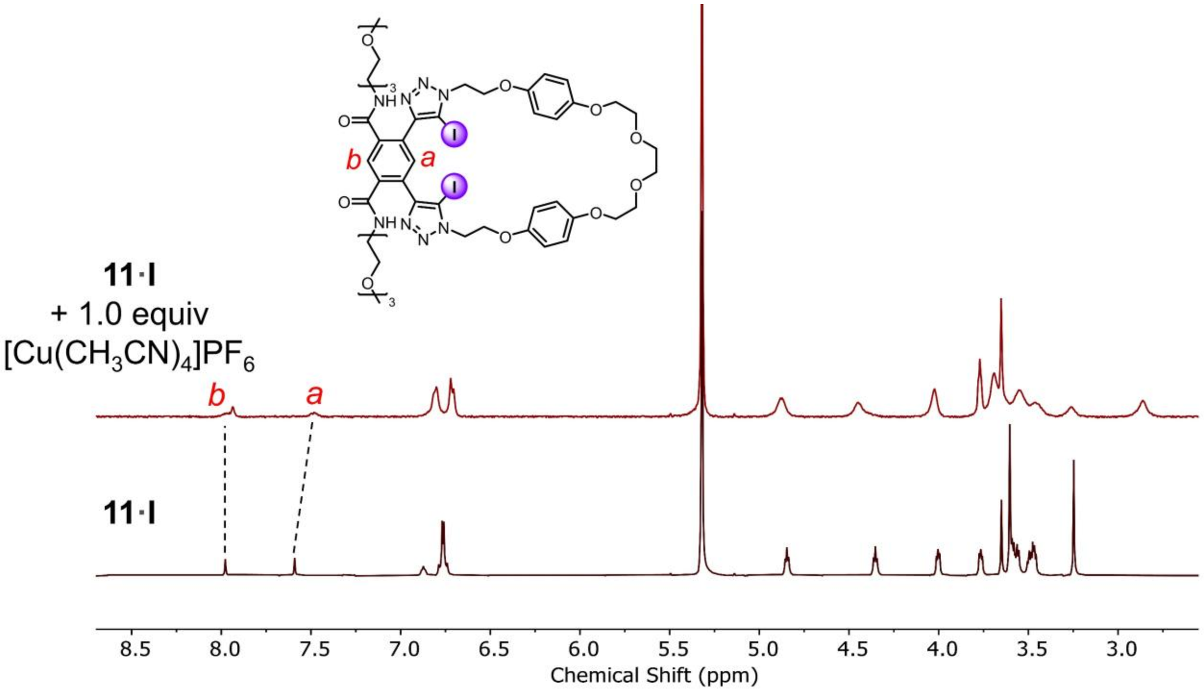

Fig. 118 | ^{1}H NMR titration spectra of XB macrocycle **11·I** upon the addition of 1.0 equivalent of $[Cu(CH_3CN)_4]PF_6$ in CD_2Cl_2 ([**11·I**] = 1.0 mM, 500 MHz, 298 K).

After establishing the capability of TEG-appended XB macrocycle **11·I** in binding Cu(I) cation in a bidentate endotopic fashion, its efficacy in facilitating active-metal template (AMT) [2]rotaxane synthesis was evaluated. Following the standard AMT protocol (Supplementary Section 1), [2]rotaxane **12·XB$_2$HB** was synthesised from XB macrocycle **11·I**, stopper azide **7** and stopper proto-alkyne **4** in 10% yield (Fig. 119). It is noteworthy this is the first example of exclusively employing iodine atom-mediated macrocycle Cu(I) binding in promoting a CuAAC-AMT rotaxane mechanical bond forming reaction.

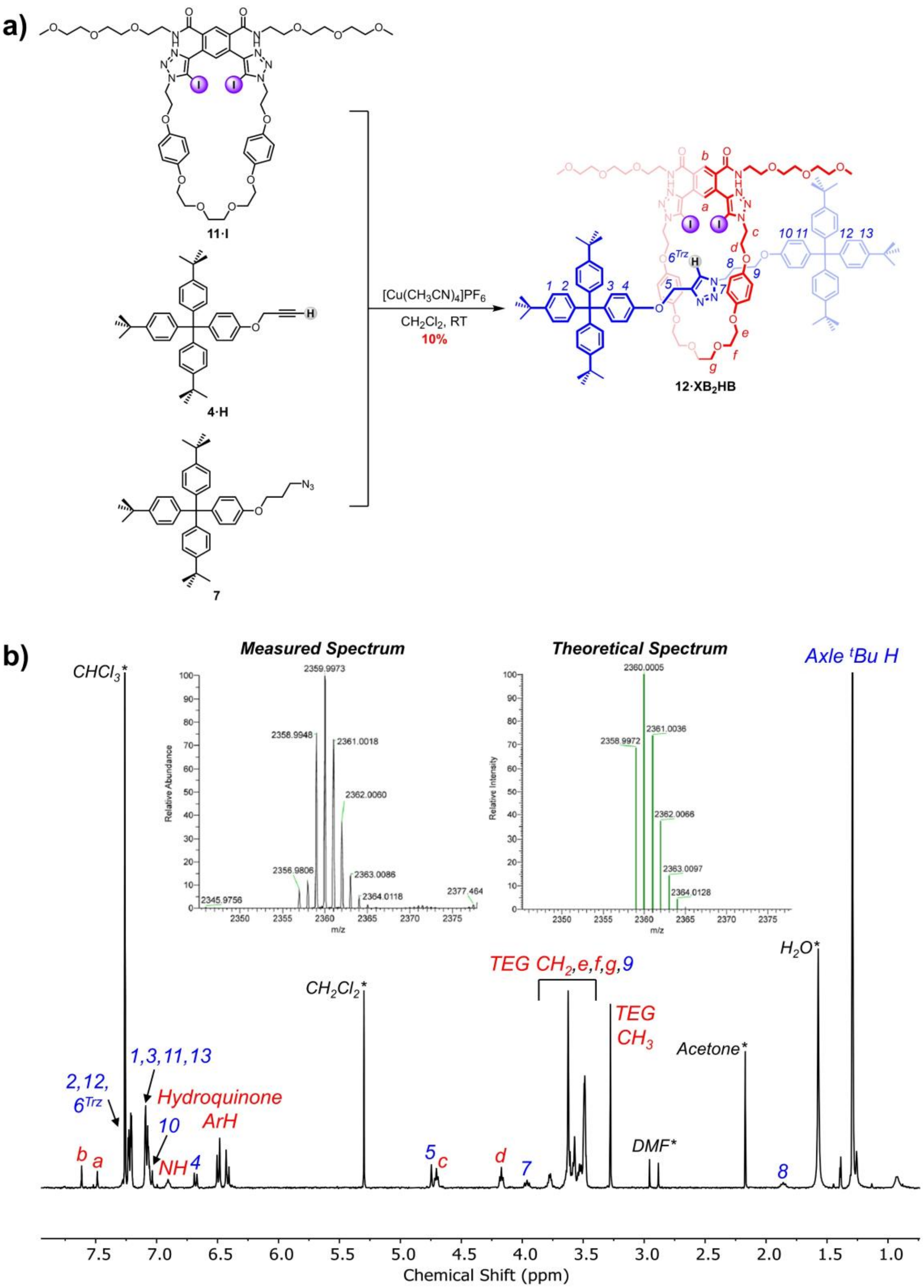

Fig. 119 | a) Active-metal template synthesis of XB [2]rotaxane **12·XB$_2$HB** via iodine-mediated Cu(I) binding, and its b) ^{1}H NMR (500 MHz, 298 K, CDCl$_3$) and high-resolution ESI mass spectra.

^{1}H NMR Cation Titration Spectra

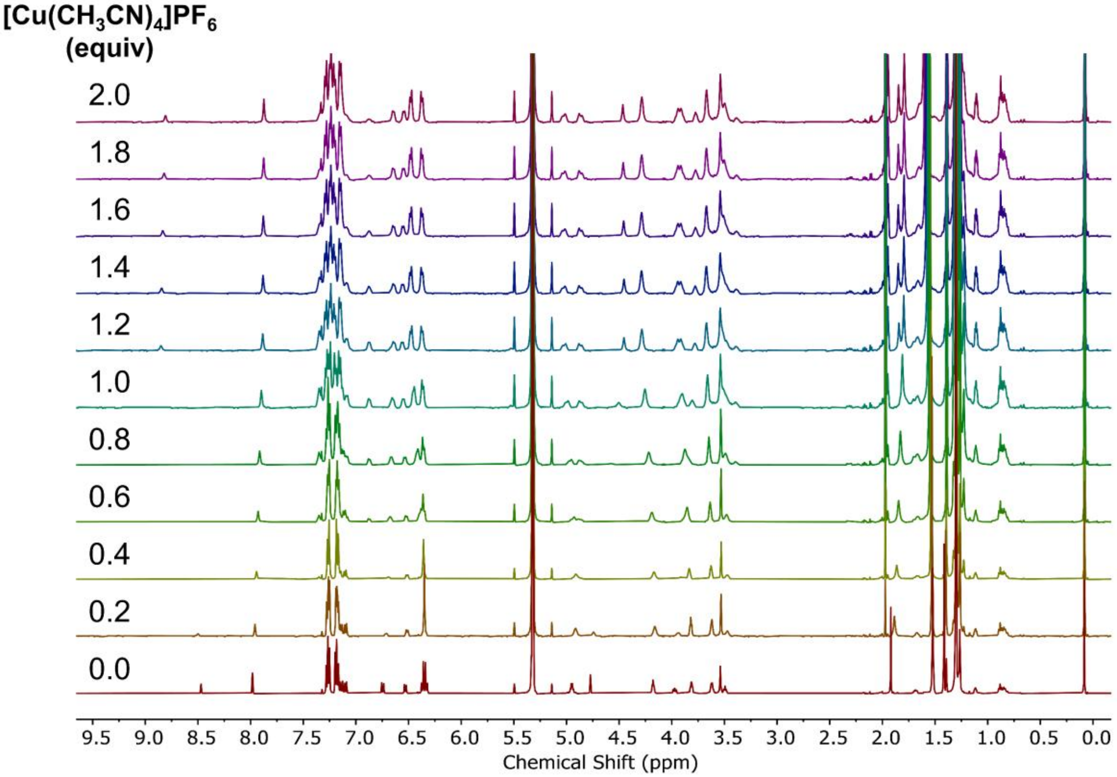

Fig. 120 | ^{1}H NMR titration spectra of **8·ChB$_3$** upon addition of 2.0 equivalents of $[Cu(CH_3CN)_4]PF_6$ ([**8·ChB$_3$**] = 1.0 mM, 500 MHz, 298 K, CD_2Cl_2).

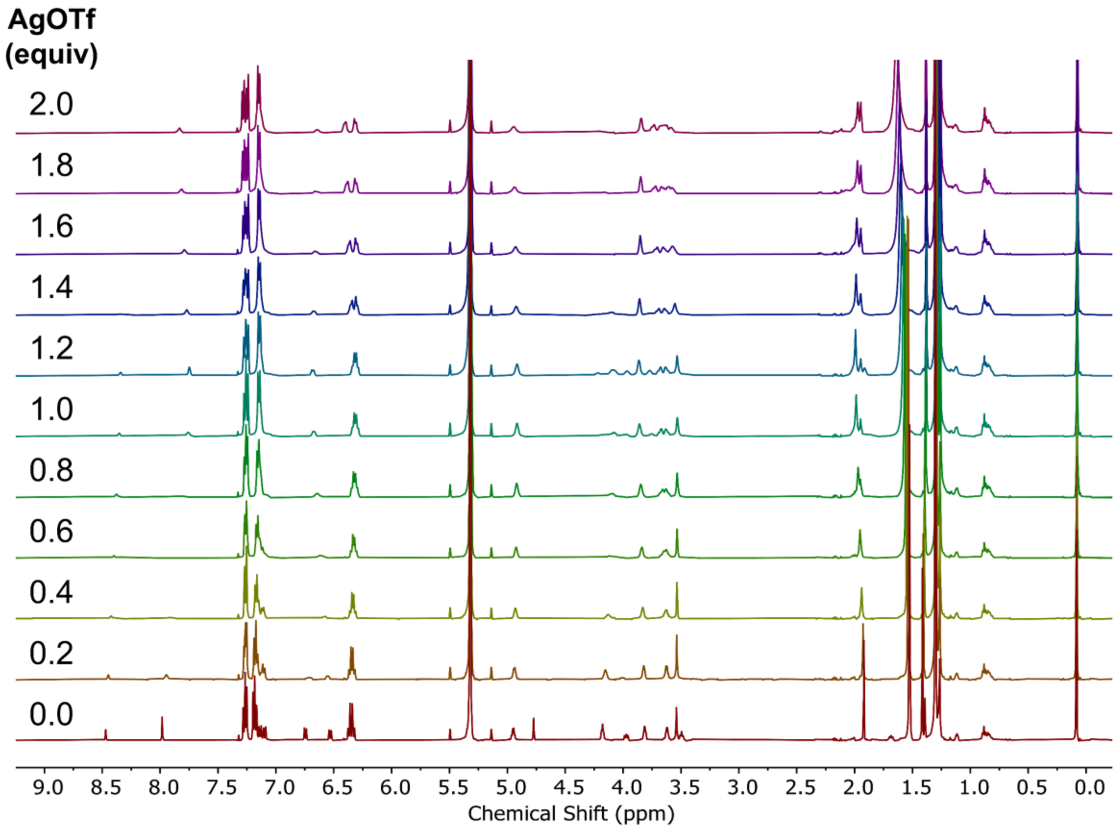

Fig. 121 | ^{1}H NMR titration spectra of **8·ChB$_3$** upon addition of 2.0 equivalents of AgOTf ([**8·ChB$_3$**] = 1.0 mM, 500 MHz, 298 K, CD_2Cl_2).

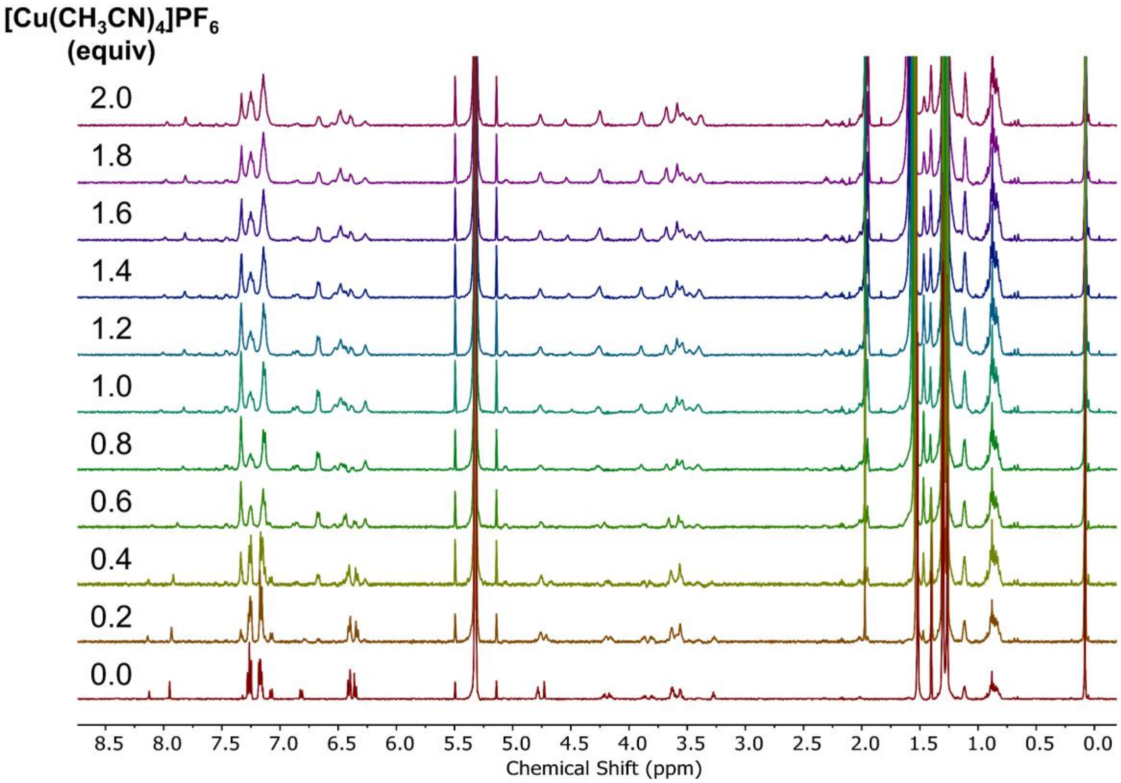

Fig. 122 | ^{1}H NMR titration spectra of **8·XB$_3$** upon addition of 2.0 equivalents of $[Cu(CH_3CN)_4]PF_6$ ([**8·XB$_3$**] = 1.0 mM, 500 MHz, 298 K, CD_2Cl_2).

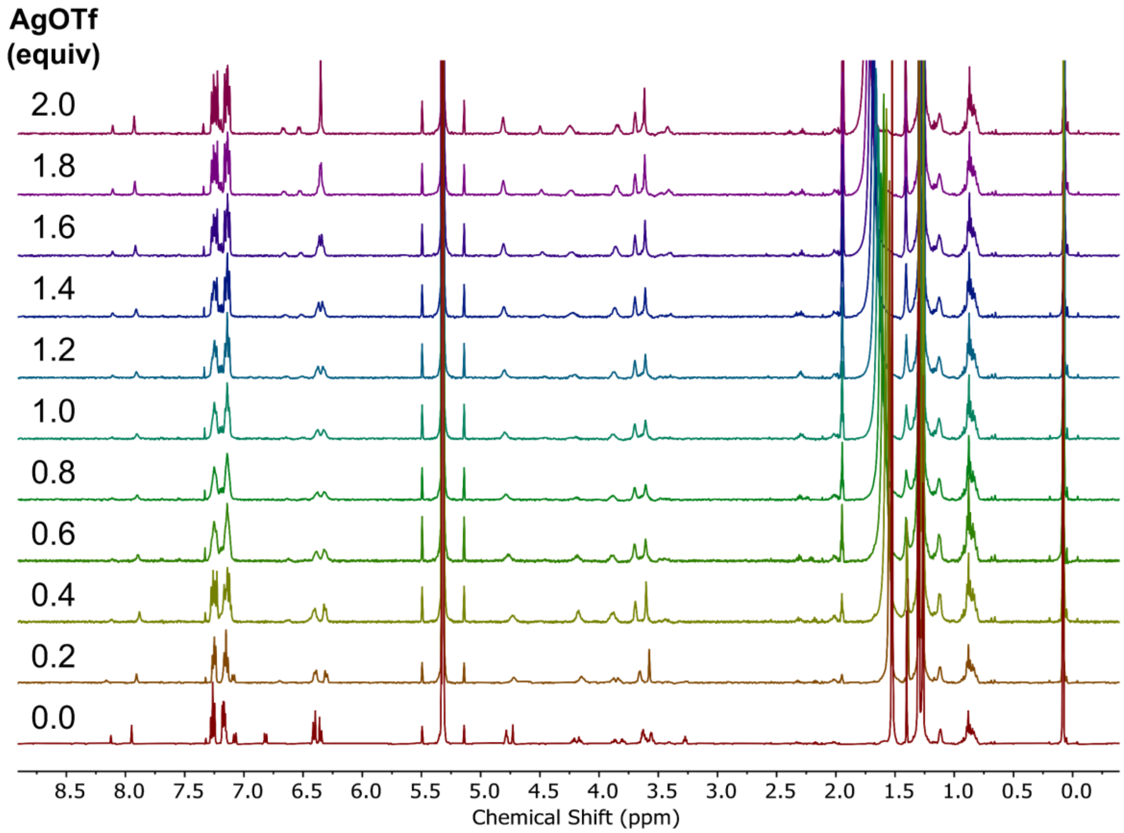

Fig. 123 | ^{1}H NMR titration spectra of **8·XB$_3$** upon addition of 2.0 equivalents of AgOTf ([**8·XB$_3$**] = 1.0 mM, 500 MHz, 298 K, CD_2Cl_2).

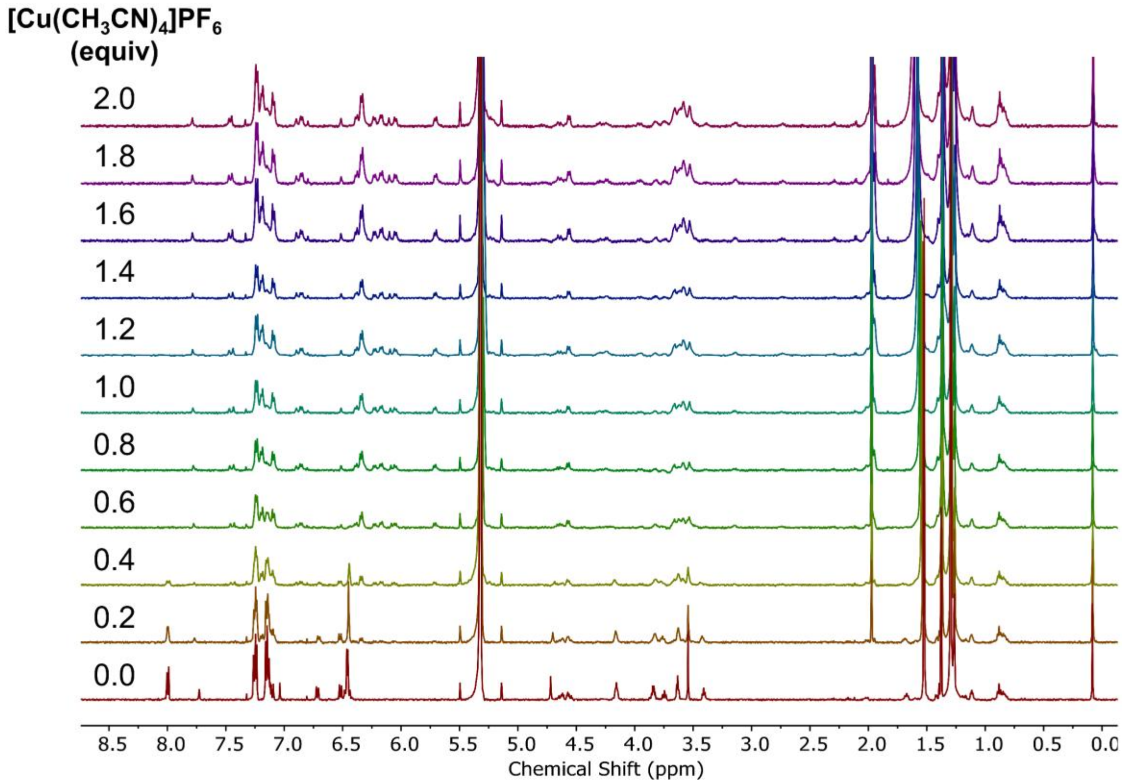

Fig. 124 | ^{1}H NMR titration spectra of **8·HB$_3$** upon addition of 2.0 equivalents of $[Cu(CH_3CN)_4]PF_6$ ([**8·HB$_3$**] = 1.0 mM, 500 MHz, 298 K, CD_2Cl_2).

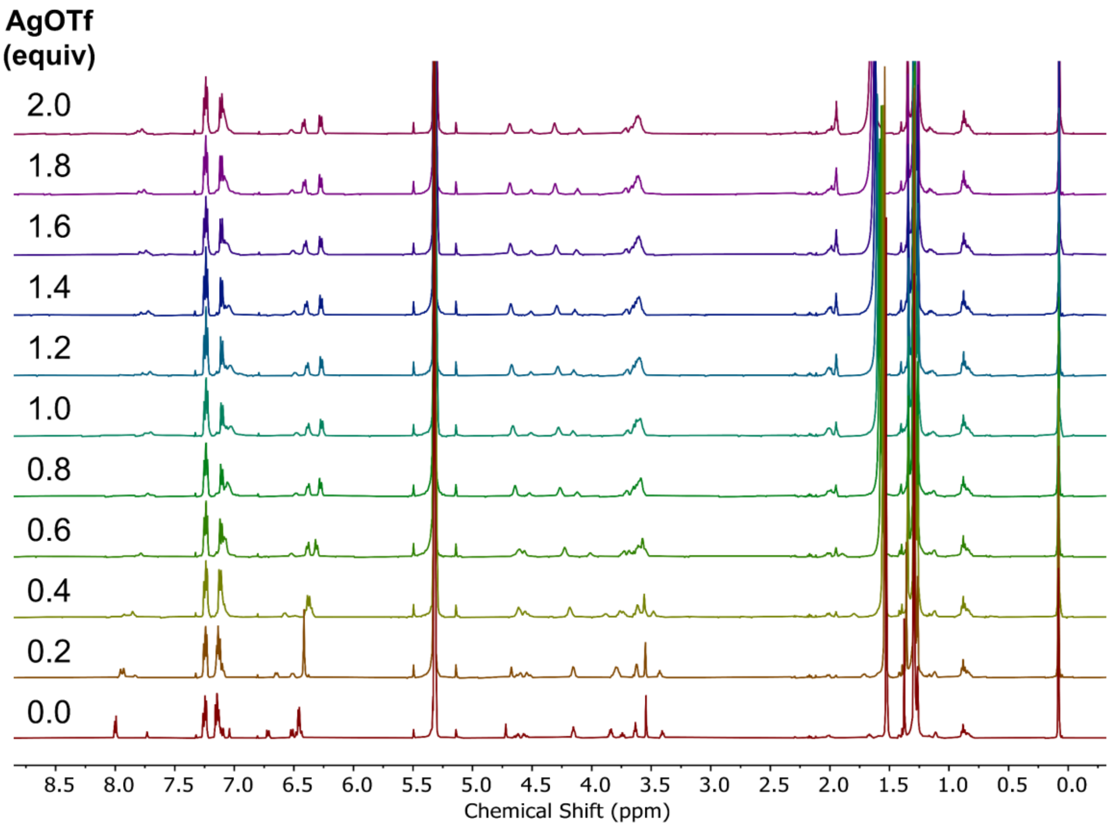

Fig. 125 | ^{1}H NMR titration spectra of **8·HB$_3$** upon addition of 2.0 equivalents of AgOTf ([**8·HB$_3$**] = 1.0 mM, 500 MHz, 298 K, CD_2Cl_2).

Synthesis of Tri(ethylene glycol)-Appended XB Macrocycle

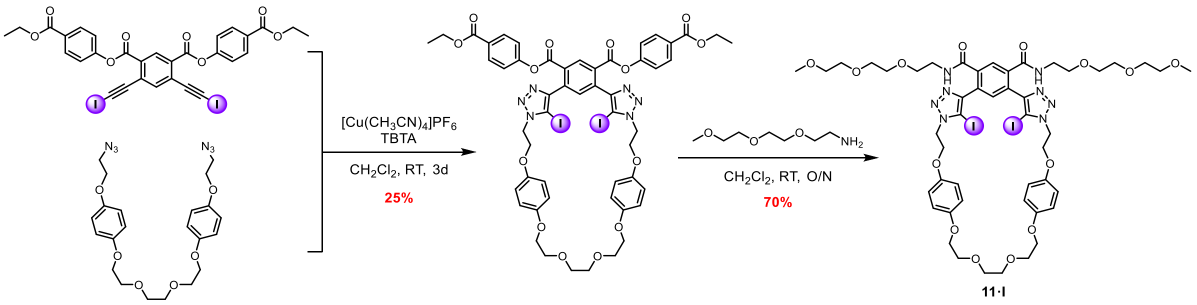

Fig. 126 | Synthesis of tri(ethylene glycol)-appended XB macrocycle **11·I**.

S7 Acknowledgement

We dedicate the publication to Professor Jean-Pierre Sauvage on the occasion of his 80th birthday. Y. C. T. thanks the Croucher Foundation for a scholarship, and his fiancée Jessica Chung for her steadfast love, support and encouragement. A. D. thanks EPSRC for studentship funding (Grant reference number EP/N509711/1). The theoretical studies were developed within the scope of the project CICECO-Aveiro Institute of Materials, UIDB/50011/2020, UIDP/50011/2020 & LA/P/0006/2020, financed by national funds through the FCT/MCTES (PIDDAC).

S8 References

1. Hein, J. E., Krasnova, L. B., Iwasaki, M., & Fokin, V. V. Cu-catalyzed azide-alkyne cycloaddition: Preparation of tris((1-benzyl-1H-1,2,3-triazolyl)methyl)amine. *Org. Synth.* **88**, 238–246 (2011).
2. Tse, Y. C., Docker, A., Zhang, Z., & Beer, P. D. Lithium halide ion-pair recognition with halogen bonding and chalcogen bonding heteroditopic macrocycles. *Chem. Commun.* **57**, 4950–4953 (2021).
3. Lim, J. Y., Marques, I., Thompson, A. L., Christensen, K. E., Félix, V., & Beer, P. D. Chalcogen bonding macrocycles and [2]rotaxanes for anion recognition. *J. Am. Chem. Soc.* **139**, 3122–3133 (2017).
4. Kilah, N. L., Wise, M. D., Serpell, C. J., Thompson, A. L., White, N. G., Christensen, K. E., & Beer, P. D. Enhancement of anion recognition exhibited by a halogen-bonding rotaxane host system. *J. Am. Chem. Soc.* **132**, 11893–11895 (2010).
5. Lim, J. Y. C., Bunchuay, T., Ayme, J.-F., & Beer, P. D. Strong and selective halide anion binding by neutral halogen-bonding [2]rotaxanes in wet organic solvents. *Chem. Eur. J.* **23**, 4700–4707 (2017).
6. Borissov, A., Marques, I., Lim, J. Y., Félix, V., Smith, M. D., & Beer, P. D. Anion recognition in water by charge-neutral halogen and chalcogen bonding foldamer receptors. *J. Am. Chem. Soc.* **141**, 4119–4129 (2019).
7. Docker, A., Bunchuay, T., Ahrens, M. J., Martinez-Martinez, A. J., & Beer, P. D. Chalcogen bonding ion-pair cryptand host discrimination of potassium halide salts. *Chem. Eur. J.* **27**, 7837–7841 (2021).

8. Docker, A., Guthrie, C. C., Kuhn, H., & Beer, P. D. Modulating chalcogen bonding and halogen bonding sigma-hole donor atom potency and selectivity for halide anion recognition. *Angew. Chem. Int. Ed.* **60**, 21973–21978 (2024).
9. LoCoco, M. D., Zhang, X., & Jordan, R. F. Chelate-controlled synthesis of racemic ansa-zirconocenes. *J. Am. Chem. Soc.* **126**, 15231–15244 (2004).
10. Stefani, H. A., Silva, N. C. S., Vasconcelos, S. N. S., Manarin, F., & Souza, F. B. Functionalization of 5-telluro-1,2,3-triazoles: Te/Li exchange and Suzuki–Miyaura cross-coupling reaction. *Tetrahedron Lett.* **54**, 2809–2812 (2013).
11. Supramolecular.org - Binding constant calculators | Supramolecular. http://app.supramolecular.org/bindfit/.
12. Thordarson, P. Determining association constants from titration experiments in supramolecular chemistry. *Chem. Soc. Rev.* **40**, 1305–1323 (2011).
13. Hibbert, D. B. & Thordarson, P. The death of the Job plot, transparency, open science and online tools, uncertainty estimation methods and other developments in supramolecular chemistry data analysis. *Chem. Commun.* **52**, 12792–12805 (2016).
14. Groom, C. R., Bruno, I. J., Lightfoot, M. P., & Ward, S. C. The Cambridge Structural Database. *Acta Crystallogr. Sect. B Struct. Sci. Cryst. Eng. Mater.* **72**, 171–179 (2016).
15. Kilah, N. L., Wise, M. D., Serpell, C. J., Thompson, A. L., White, N. G., Christensen, K. E., & Beer, P. D. Crystallographic implications for the design of halogen bonding anion receptors. *Cryst. Growth Des.* **11**, 4565–4571 (2011).
16. Bunchuay, T., Docker, A., Martinez-Martinez, A. J., & Beer, P. D. A potent halogen-bonding donor motif for anion recognition and anion template mechanical bond synthesis. *Angew. Chem. Int. Ed.* **58**, 13823–13827 (2019).
17. Frisch, M. J., Trucks, G. W., Schlegel, H. B., Scuseria, G. E., Robb, M. A., Cheeseman, J. R., Scalmani, G. , Barone, V. , Petersson, G. A., Nakatsuji, H., Li, X., Caricato, M., Marenich, A. V., Bloino, J., Janesko, B. G., Gomperts, R., Mennucci, B., Hratchian, H. P., Ortiz, J. V., Izmaylov, A. F., Sonnenberg, J. L., Williams-Young, D., Ding, F., Lipparini, F., Egidi, F., Goings, J., Peng, B., Petrone, A., Henderson, T., Ranasinghe, D., Zakrzewski, V. G., Gao, J., Rega, N., Zheng, G., Liang, W., Hada, M., Ehara, M., Toyota, K., Fukuda, R., Hasegawa, J., Ishida, M., Nakajima, T., Honda, Y., Kitao, O., Nakai, H., Vreven, T., Throssell, K., Montgomery, J. A., Jr., Peralta, J. E., Ogliaro, F., Bearpark, M. J., Heyd, J. J., Brothers, E. N., Kudin, K. N., Staroverov, V. N., Keith, T. A., Kobayashi, R., Normand, J., Raghavachari, K., Rendell, A. P., Burant, J. C., Iyengar, S. S., Tomasi, J., Cossi, M., Millam, J. M., Klene, M., Adamo, C., Cammi, R.,

Ochterski, R. L., Martin, J. W., Morokuma, K., Farkas, O., Foresman, J. B., & Fox, D. J. Gaussian 16, Revision C.01. *Gaussian, Inc., Wallingford CT* (2019).

18. Adamo, C. & Barone, V. Toward reliable density functional methods without adjustable parameters: The PBE0 model. *J. Chem. Phys.* **110**, 6158–6170 (1999).
19. Grimme, S., Antony, J., Ehrlich, S., & Krieg, H. A consistent and accurate ab initio parametrization of density functional dispersion correction (DFT-D) for the 94 elements H-Pu. *J. Chem. Phys.* **132**, 154104 (2010).
20. Grimme, S., Ehrlich, S., & Goerigk, L. Effect of the damping function in dispersion corrected density functional theory. *J. Comput. Chem.* **32**, 1456–1465 (2011).
21. Weigend, F. & Ahlrichs, R. Balanced basis sets of split valence, triple zeta valence and quadruple zeta valence quality for H to Rn: Design and assessment of accuracy. *Phys. Chem. Chem. Phys.* **7**, 3297–3305 (2005).
22. Andrae, D., Häussermann, U., Dolg, M., Stoll, H., & Preuß, H. Energy-adjusted ab initio pseudopotentials for the second and third row transition elements. *Theor. Chim. Acta* **77**, 123–141 (1990).
23. Metz, B., Schweizer, M., Stoll, H., Dolg, M., & Liu, W. A small-core multiconfiguration Dirac-Hartree-Fock-adjusted pseudopotential for Tl: Application to TlX (X = F, Cl, Br, I). *Theor. Chem. Acc.* **104**, 22–28 (2000).
24. Lu, T. & Chen, F. Multiwfn: A multifunctional wavefunction analyzer. *J. Comput. Chem.* **33**, 580–592 (2012).
25. Lu, T. & Chen, F. Quantitative analysis of molecular surface based on improved Marching Tetrahedra algorithm. *J. Mol. Graph. Model.* **38**, 314–323 (2012).
26. Barone, V. & Cossi, M. Quantum calculation of molecular energies and energy gradients in solution by a conductor solvent model. *J. Phys. Chem. A* **102**, 1995–2001 (1998).
27. Cossi, M., Rega, N., Scalmani, G., & Barone, V. Energies, structures, and electronic properties of molecules in solution with the C-PCM solvation model. *J. Comput. Chem.* **24**, 669–681 (2003).
28. Zhao, Y. & Truhlar, D. G. The M06 suite of density functionals for main group thermochemistry, thermochemical kinetics, noncovalent interactions, excited states, and transition elements: Two new functionals and systematic testing of four M06-class functionals and 12 other functionals. *Theor. Chem. Acc.* **120**, 215–241 (2008).
29. Zhao, Y. & Truhlar, D. G. The M06 suite of density functionals for main group thermochemistry, thermochemical kinetics, noncovalent interactions, excited states,

and transition elements: Two new functionals and systematic testing of four M06 functionals and 12 other functionals. *Theor. Chem. Acc.* **119**, 525–525 (2008).

30. Peterson, K. A., Figgen, D., Goll, E., Stoll, H., & Dolg, M. Systematically convergent basis sets with relativistic pseudopotentials. II. Small-core pseudopotentials and correlation consistent basis sets for the post-d group 16–18 elements. *J. Chem. Phys.* **119**, 11113–11123 (2003).
31. Rappoport, D. & Furche, F. Property-optimized Gaussian basis sets for molecular response calculations. *J. Chem. Phys.* **133**, 134105 (2010).
32. Glendening, E. D., Landis, C. R., & Weinhold, F. Natural bond orbital methods. *WIREs Comput. Mol. Sci.* **2**, 1–42 (2012).
33. Glendening, E. D., Landis, C. R., & Weinhold, F. NBO 7.0: New vistas in localized and delocalized chemical bonding theory. *J. Comput. Chem.* **40**, 1410–1423 (2019).
34. Reed, A. E., Weinstock, R. B., & Weinhold, F. Natural population analysis. *J. Chem. Phys.* **83**, 735–746 (1985).
35. Mayer, I. & Salvador, P. Overlap populations, bond orders and valences for ‘fuzzy’ atoms. *Chem. Phys. Lett.* **383**, 368–375 (2004).
36. Docker, A., Marques, I., Kuhn, H., Zhang, Z., Félix, V., & Beer, P. D. Selective potassium chloride recognition, sensing, extraction, and transport using a chalcogen-bonding heteroditopic receptor. *J. Am. Chem. Soc.* **144**, 14778–14789 (2022).
37. Engelage, E., Schulz, N., Heinen, F., Huber, S. M., Truhlar, D. G., & Cramer, C. J. Refined SMD parameters for bromine and iodine accurately model halogen-bonding interactions in solution. *Chem. Eur. J.* **24**, 15983–15987 (2018).
38. Akbaba, S., Steinke, T., Vogel, L., Engelage, E., Erdelyi, M., & Huber, S. M. Elucidating the binding mode of sulfur- and selenium-based cationic chalcogen-bond donors. *Chem. Eur. J.* **30**, e202400608 (2024).
39. Marenich, A. V., Cramer, C. J., & Truhlar, D. G. Universal solvation model based on solute electron density and on a continuum model of the solvent defined by the bulk dielectric constant and atomic surface tensions. *J. Phys. Chem. B* **113**, 6378–6396 (2009).
40. Scalmani, G. & Frisch, M. J. Continuous surface charge polarizable continuum models of solvation. I. General formalism. *J. Chem. Phys.* **132**, 114110 (2010).
41. Ji, G. et al. Direct copolymerization of ethylene with protic comonomers enabled by multinuclear Ni catalysts. *Nat. Commun.* **12**, 6283 (2021).

42. Wu, L., Sheong, F. K., & Lin, Z. DFT studies on copper-catalyzed dearomatization of pyridine. *ACS Catal.* **10**, 9585–9593 (2020).
43. Gallegos, F. E., Meneses, L. M., Cuesta, S. A., Santos, J. C., Arias, J., Carrillo, P., & Pilaquinga, F. Computational modeling of the interaction of silver clusters with carbohydrates. *ACS Omega* **7**, 4750–4756 (2022).
44. Kircheva, N. & Dudev, T. Gallium as an antibacterial agent: A DFT/SMD study of the Ga^{3+}/Fe^{3+} competition for binding bacterial siderophores. *Inorg. Chem.* **59**, 6242–6254 (2020).
45. Morris, L. J., Ghana, P., Rajeshkumar, T., Carpentier, A., Maron, L., & Okuda, J. A Brønsted acidic gallium hydride: Facile interconversion of NNNN-macrocycle supported $[Ga\ I]^{+}$ and $[Ga\ III\ H]^{2+}$. *Angew. Chem. Int. Ed.* **61**, e202114629 (2022).
46. Lin, T.-P. & Gabbaï, F. P. Two-electron redox chemistry at the dinuclear core of a TePt platform: Chlorine photoreductive elimination and isolation of a $Te^{5+}Pt^{5+}$ complex. *J. Am. Chem. Soc.* **134**, 12230–12238 (2012).
47. Freindorf, M., Yannacone, S., Oliveira, V., Verma, N., & Kraka, E. Halogen bonding involving I_2 and d^8 transition-metal pincer complexes. *Crystals* **11**, 373 (2021).
48. Fleischel, O., Wu, N., & Petitjean, A. Click-triazole: Coordination of 2-(1,2,3-triazol-4-yl)-pyridine to cations of traditional tetrahedral geometry (Cu(i), Ag(i)). *Chem. Commun.* **46**, 8454 (2010).
49. Monkowius, U., Ritter, S., König, B., Zabel, M., & Yersin, H. Synthesis, characterisation and ligand properties of novel Bi-1,2,3-triazole ligands. *Eur. J. Inorg. Chem.* **2007**, 4597–4606 (2007).
